# Single-atom-engineered perovskite enables near-theoretical-rate hydroxyl radical electrogeneration

**DOI:** 10.1038/s41467-026-75926-5

**Published:** 2026-07-24

**Authors:** Yaobin Wang, Chaoyue Xie, Ruiqing Zhao, Zhiyuan Su, Hongmei Li, Hang Zhang, Bo Li, Changhui Zhou, Yongyang Chen, Zeyu Du, Jinhua Li, Yunfei Bu, Jing Bai, Baoxue Zhou, Emiliano Cortés, Min Liu

**Affiliations:** 1 State Key Laboratory of Green Papermaking and Resource Recycling, School of Environmental Science and Engineering, Shanghai Jiao Tong University, Shanghai, China; 2 Hunan Joint International Research Center for Carbon Dioxide Resource Utilization, School of Physics, Central South University, Changsha, China; 3 School of Metallurgy and Environment, Central South University, Changsha, China; 4 Nanoinstitut München, Fakultät für Physik, Ludwig-Maximilians-Universität München, München, Germany; 5 School of Science, STEM College, RMIT University, Melbourne, VIC Australia; 6 UNIST-NUIST Energy and Environment Jointed Lab (UNNU), School of Environment Science and Technology, Nanjing University of Information Science and Technology, No.219, Nanjing, China

**Keywords:** Electrocatalysis, Energy, Electrocatalysis

## Abstract

Electrochemical advanced oxidation that directly activates O_2_ through the oxygen reduction reaction (ORR) to generate hydroxyl radicals (•OH) offers a sustainable strategy for degrading persistent organic pollutants. However, prevailing approaches typically rely on a stepwise process involving the 2e⁻ ORR to produce H_2_O_2_ followed by 1e⁻ activation. High barriers associated with intermediate desorption and inter-site transfer consequently limit the •OH yield. Here, we construct a single-active-site architecture in the perovskite oxide Pr_1.0_Sr_1.0_Fe_0.5_Zn_0.25_Mo_0.25_O_4-δ_ (PSFZM) that enables a direct three-electron ORR pathway for efficient •OH generation. The Zn^δ^⁺ single active center selectively stabilizes *OOH and *H_2_O_2_ through weak orbital interactions, while an adjacent Mo atom polarizes the O atoms of adsorbed H_2_O_2_, promoting cleavage of the peroxide bond at the active site. This strategy avoids intermediate desorption and migration, enabling continuous proton-coupled electron transfer. The catalyst achieves a •OH production rate of 821 μmol h⁻^1^ and an O_2_ utilization of 37.7%, metrics competitive with previously reported systems. In a membrane-free flow cell that uses gaseous O_2_ directly, the •OH generation efficiency reaches 64.7%. By combining atomic-level catalyst design with reactor engineering, this work establishes a scalable platform for sustainable wastewater treatment.

## Abstract (web summary)

Electrochemical O2 activation enables in situ hydroxyl radical (•OH) production, offering a sustainable route for pollutant degradation. Here, the authors report a single-atom engineering strategy that delivers highly competitive •OH yields through a consecutive 3e⁻ oxygen reduction reaction.

## Introduction

The global freshwater crisis is intensifying, coupled with rapid urbanization and a surge in emerging organic pollutants (EOPs) from key industries such as papermaking, electronics, and pharmaceuticals, necessitating the development of advanced and sustainable wastewater treatment technologies^1–3^. Among numerous methods, electrochemical advanced oxidation processes (EAOPs) are especially attractive as they directly activate O_2_ via the oxygen reduction reaction (ORR) to produce hydroxyl radicals (•OH), enabling reagent-free degradation of recalcitrant contaminants^4–7^. Compared to conventional Fenton-based processes, EAOPs avoid inherent limitations such as strict pH requirements, iron-sludge formation, and continuous chemical consumption^8–10^. However, on most heterogeneous catalysts, the ORR pathway toward •OH generation proceeds through a sequential 2e⁻ transfer followed with 1e^−^ activation steps: O_2_ is first reduced to *OOH and subsequently to *H_2_O_2_, followed by O-O bond cleavage to produce •OH^11–15^. The differences in bond polarity and dissociation energetics between *OOH and *H_2_O_2_ present a significant mechanistic challenge, making the simultaneous stabilization and efficient conversion of these intermediates inherently difficult^16–21^.

**Fig. 1:**
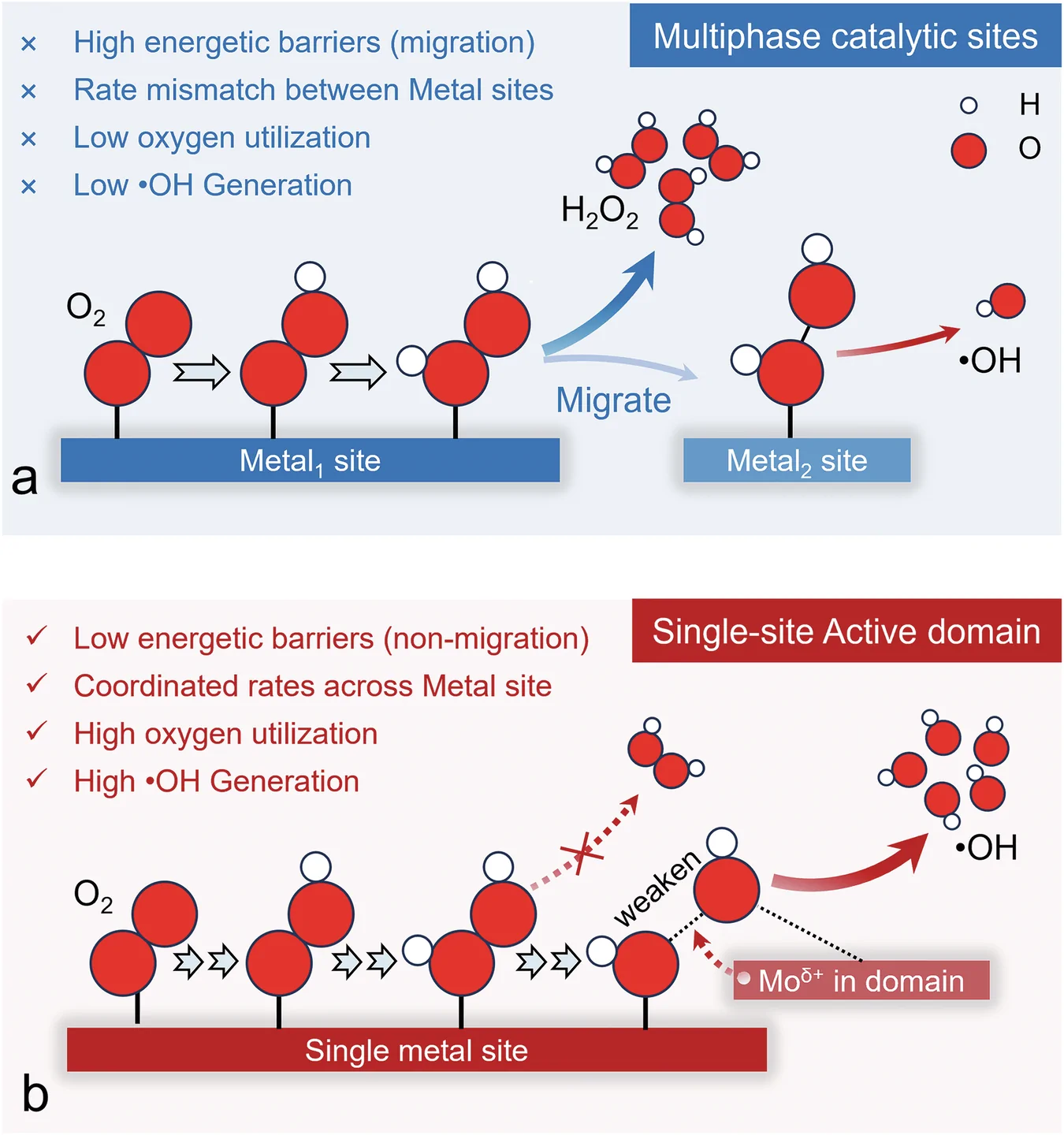
Intermediate recognition strategy promotes •OH generation. **a** The evolution of O_2_ activation in a classic multiphase catalytic system. Long pathway resulting from the migration of intermediates from the Metal_1_ site to the Metal_2_ site. **b** The evolution of O_2_ activation in the single-site active domain catalytic system. The intermediate completes the adsorption, protonation and O–O bond cleavage process in the domain.

Although prior studies have explored heterogeneous core-shell catalysts and diatomic catalysts to decouple the 2e^−^ ORR step (H_2_O_2_ generation) and 1e^−^ *H_2_O_2_ activation (•OH formation), these stepwise and spatially separated pathways suffer from several intrinsic kinetic bottlenecks: (i) high energetic barriers associated with intermediate desorption and migration between distinct active sites^22–24^, (ii) asynchronous reaction rates between the 2e^−^ ORR and 1e^−^ *H_2_O_2_ activation steps^25,26^, and (iii) inefficient oxygen utilization due to intermediates exiting reactive zones before complete conversion (Fig. 1a)^27–32^.

To overcome these limitations, we propose a single-site active domain strategy, wherein cooperative multi-metal catalysis is confined to a single crystallographic domain. This enables the ORR to proceed through three-step proton-coupled electron transfer (PCET) at a single catalytic site, eliminating the need for the traditional (2e^−^ + 1e^−^) cascade mechanism (Fig. 1b and Supplementary Fig. 1). A designed perovskite catalyst, PSFZM (Pr_1_Sr_1_Fe_0.5_Zn_0.25_Mo_0.25_O_4-δ_), was synthesized, featuring a Zn^δ+^ single-site that stabilizes *OOH and *H_2_O_2_ via weak orbital interactions, while adjacent Mo atoms cooperatively polarize the O atoms of adsorbed H_2_O_2_, promoting controlled HO-OH cleavage at a single site and enabling low-barrier conversion to •OH. As a result, PSFZM achieves a Faradaic efficiency (FE) of 28.7% for the 3e^−^ ORR pathway toward •OH, with an O_2_ utilization rate of 37.7% and a radical generation rate of 821 μmol h^−^^1^. These metrics compare favorably with those of reported catalysts based on decoupled 2e^−^ ORR and subsequent 1e^−^ H_2_O_2_ activation processes. Furthermore, when deployed in a membrane-free cylindrical electrolyzer integrating 3e^−^ ORR at the cathode and 1e^−^ water oxidation at the anode, the system delivers an overall •OH generation efficiency of 64.7%, supported by a stable gas-liquid-solid triple-phase interface and highly efficient reactant mass transfer.

## Results

### Synthesis and structural characterization of the PSFZM active domains

**Fig. 2:**
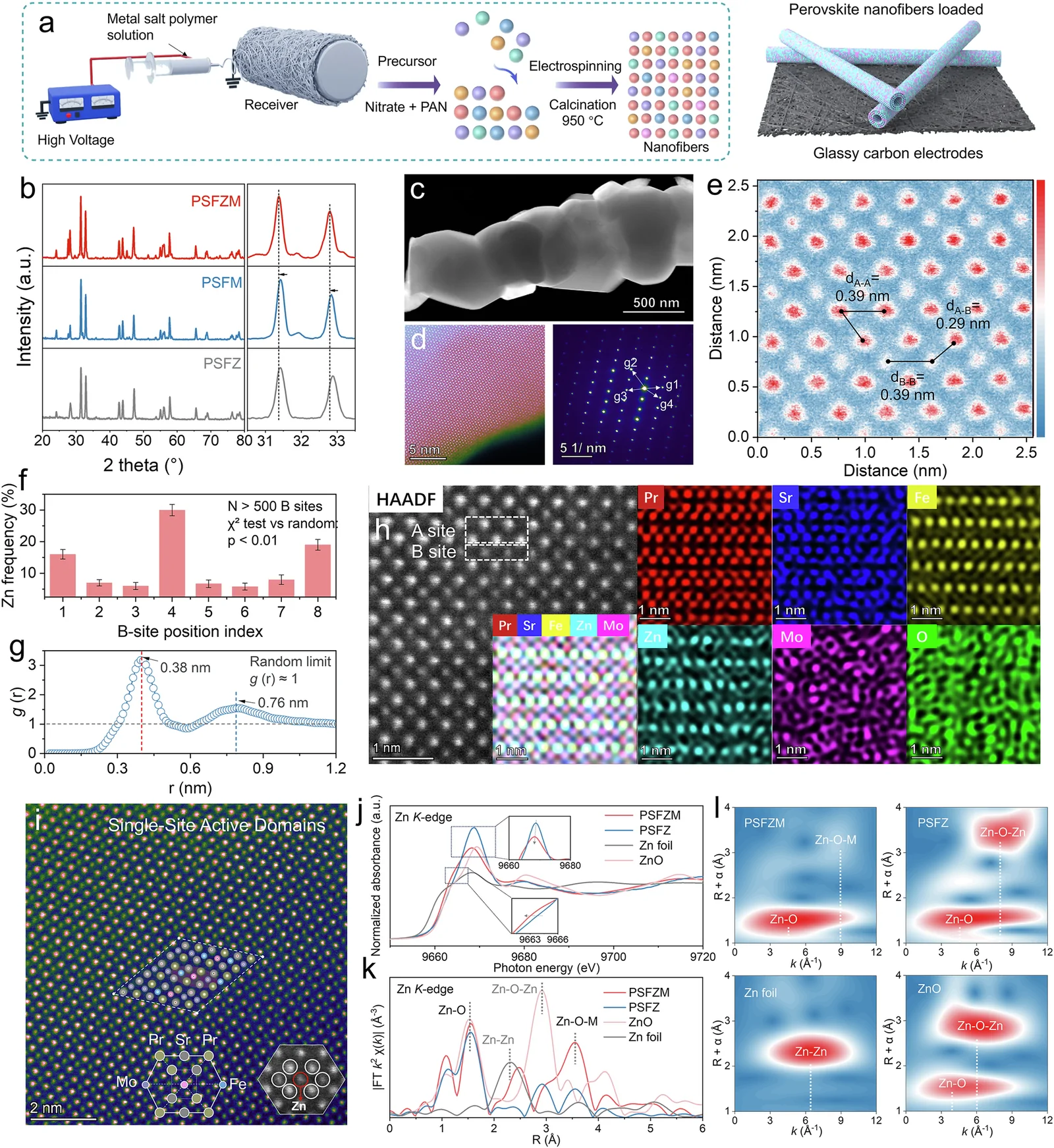
Atomic structure characterization of the active domains for PSFZM. **a** Schematic diagram showing the preparation of PSFZM electrodes by the electrospinning method. Colors represent: Pr (red), Sr (purple), Fe (yellow), Zn (green), Mo (blue). **b** XRD patterns of PSFZM, PSFZ and PSFM. **c** SEM image of PSFZM. **d** HAADF-STEM image. The inset shows the corresponding selected area electron diffraction (SAED) pattern of PSFZM. **e** Atomic spacing diagram based on HAADF-STEM. **f** Periodicity analysis of Zn occupation on the B-site sublattice. Error bars represent the standard deviation of Zn occupation frequencies obtained from *n* = 3 independent HAADF-STEM images/regions. **g** Radial distribution function g(r) of Zn-rich B-site columns. **h** HAADF-STEM image and the corresponding elemental maps for PSFZM. **i** The elemental intensity maps obtained from HAADF-STEM conversion show that elements with larger ionic radii are A-site elements, while those with smaller ionic radii are B-site elements. The inset shows an overlaid comparison between the atomic contrast observed by STEM and the corresponding atomic model, while the lower-left inset presents the atomic distribution and simulated image within a unit active domain. **j** Zn *K*-edge XANES spectra of PSFZM, PSFZ, Zn foil, ZnO. **k** Zn *K*-edge *k*^*3*^-weighted FT-EXAFS spectra and **l** WT-EXAFS spectra of PSFZM, PSFZ, Zn foil, ZnO. Source data for this figure are provided as a Source data file.

To materialize the proposed single-site active domain strategy, PSFZM, along with control samples of PSFZ (Pr_1_Sr_1_Fe_0.75_Zn_0.25_O_4-δ_) and PSFM (Pr_1_Sr_1_Fe_0.75_Mo_0.25_O_4-δ_) were synthesized via electrospinning (Fig. 2a). Rietveld-refined X-ray Diffraction (XRD) patterns confirm that PSFM, PSFZ, and PSFZM retain the tetragonal Ruddlesden-Popper structure with the I4/mmm space group (Supplementary Figs. 2–4 and Supplementary Table 1). PSFZM exhibits the largest unit-cell volume and the most pronounced elongation of axial B–O bonds, accompanied by left-shifted diffraction peaks at ~32° and ~34° (Fig. 2b). These crystallographic features demonstrate that Zn/Mo co-doping induces lattice expansion and BO_6_ octahedral distortion, supporting the formation of mixed-metal B–O active domains distinct from the single-metal local environments. Field-emission scanning electron microscopy (FE-SEM) and high-angle annular dark-field scanning transmission electron microscopy (HAADF-STEM) images reveal that PSFZM forms a highly crystalline porous nanofiber network structure (Fig. 2c and Supplementary Figs. 5–7c). Aberration-corrected high-resolution transmission electron microscopy (AC-HRTEM) images (Fig. 2d) together with the atomic spacing map (Fig. 2e) further disclose an orthogonally distorted tetragonal lattice within PSFZM. Quantitative statistical analysis of more than 500 B-site atomic columns indicates that the distribution of Zn sites in PSFZM is non-random (Supplementary Figs. 8 and 9), showing a significant deviation from the random occupancy model according to the *χ*^2^ test (*p* < 0.01; Fig. 2f). The radial distribution function decays toward the random-limit value of g(r) ≈ 1 (Fig. 2g), indicating the presence of a localized, zinc-enriched ordered structure. Atomic-scale elemental mapping and STEM-energy dispersive X-ray spectroscopy (EDS) line-scan analysis further confirms this conclusion, showing that Pr and Sr atoms are uniformly distributed at the A sites, whereas Zn atoms are periodically enriched at the B sites near the center of the distorted tetragonal unit cells (Fig. 2h, i and Supplementary Figs. 7d, 10–11). Collectively, these results provide compelling evidence for the formation of Zn-centered active-domain units.

The local coordination environment was probed by X-ray absorption fine structure (XAFS) spectroscopy. The Zn K-edge XANES spectrum of PSFZM exhibits an absorption edge positioned between those of Zn foil and ZnO, indicating a reduced Zn valence state (Zn^δ⁺^) relative to PSFZ (Fig. 2j and Supplementary Fig. 12). Consistently, the Zn–O coordination number decreases from 4.3 ± 1.0 in PSFZ to 3.7 ± 0.6 in PSFZM (Supplementary Table 2), corroborating this observation. Fourier-transformed EXAFS (FT-EXAFS) analyses further reveal a pronounced peak at ~2.48 Å in PSFZM, located between the Zn–Zn scattering of Zn foil (2.24 Å) and the Zn–O–Zn scattering of ZnO (2.98 Å) (Supplementary Fig. 13). This intermediate position indicates that the Zn–O–Zn motif in PSFZM does not arise from a continuous Zn–O–Zn–O aggregated network, but rather reflects a second-nearest-neighbor geometric correlation between Zn and the surrounding oxygen polyhedra. More importantly, PSFZM features a distinct Zn–O–M coordination shell with a bond length of 3.88 ± 0.01 Å, and the spectrum can be reproduced without invoking any direct Zn-Zn scattering contribution. This scattering path is absent in both PSFZ and the Zn reference samples (Fig. 2k), pointing to a stronger Zn-centered local ordering tendency in PSFZM^33^. Wavelet-transformed (WT) EXAFS contour plots (Fig. 2l) resolve the scattering contributions in the (k, R) plane and further substantiate this conclusion: PSFZM displays a low-k intensity maximum corresponding to Zn–O backscattering, together with a discrete high-k lobe at *k* ≈ 9 Å^–1^ that is attributed to the heavier second-shell Zn–O–M path. Combined with the X-ray photoelectron spectroscopy (XPS) results, this direct structural evidence confirms the successful construction of atomic-scale Zn–O–M coupling within the perovskite matrix (Supplementary Figs. 14–18). Taken together, these structural analyses verify that periodically modulated active domains have been successfully established in PSFZM.

### Electrode kinetics of the three-electron oxygen reduction reaction

**Fig. 3:**
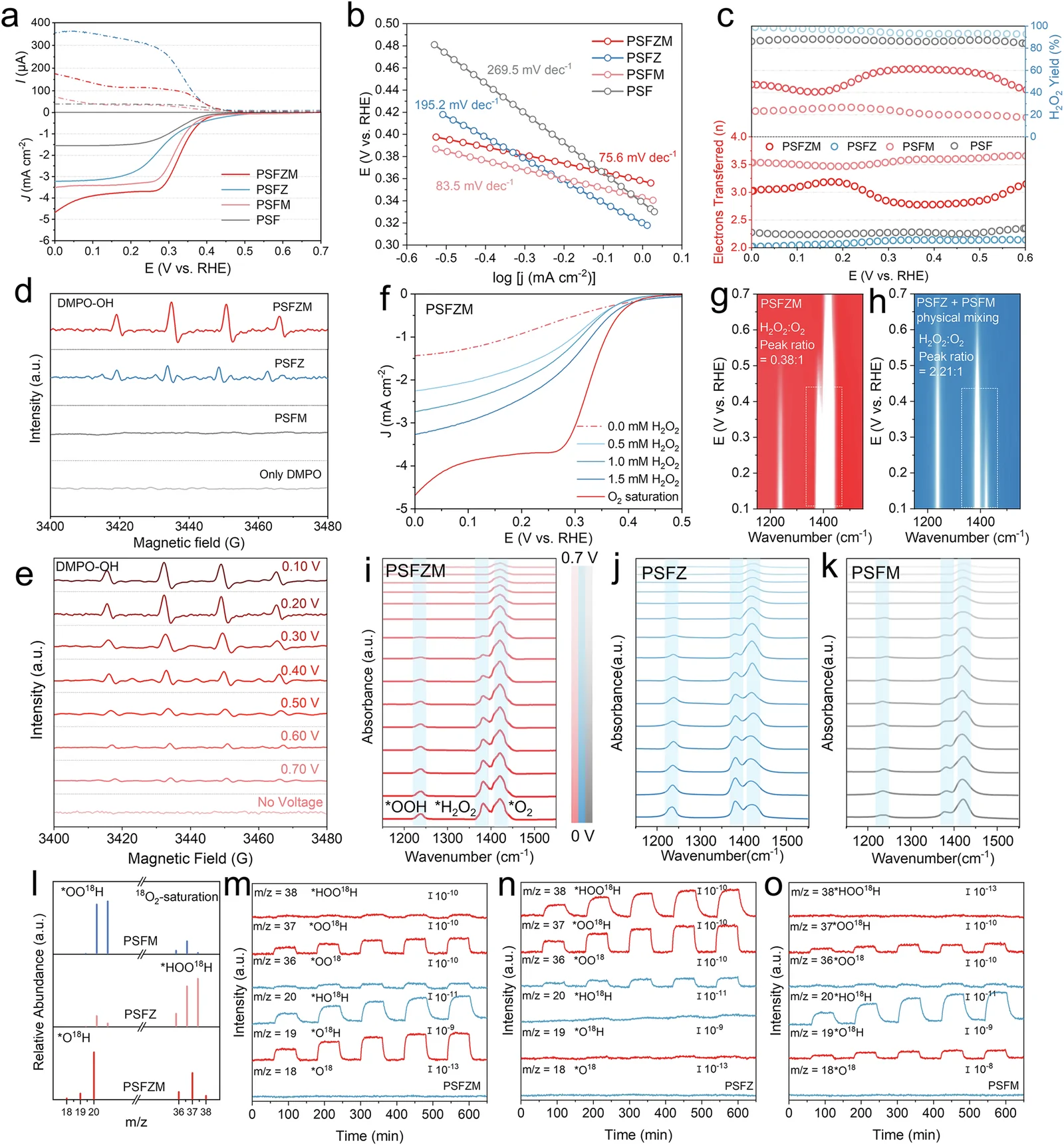
Qualitative analysis of ORR selectivity and O_2_ conversion to •OH. **a** RRDE polarization profiles for oxygen reduction reaction. Note: The solid lines are the disk current density corresponding to oxygen reduction and the dotted lines are the ring current density corresponding to H_2_O_2_ oxidation, non-iR corrected. The measurements were conducted in O_2_-saturated 1.0 M Na_2_SO_4_ at room temperature, with a pH = 7.8 and a catalyst loading of 0.202 mg cm^−^^2^. **b** The corresponding Tafel plots. **c** H_2_O_2_ selectivity and number of transferred electrons (*n*) calculated from RRDE polarization curves. **d** EPR spectra of DMPO−•OH adducts for samples from different catalyst-catalyzed electro-Fenton reactions. **e** DMPO−•OH adduct EPR spectra of ORR electrochemistry over PSFZM catalyst collected in an incremental voltage configuration. **f** LSV with different concentrations of H_2_O_2_ raw materials in the O_2_-free configuration and the O_2_-filled configuration. Presented are the potential-dependent in situ FTIR contour plots of **g** PSFZM and the **h** PSFZ + PSFM physical mixture, acquired in an O_2_-saturated electrolyte. The ORR electrochemical in-situ FTIR spectra of **i** PSFZM, **j** PSFZ, and **k** PSFM catalyst collected in an incremental voltage configuration. **l** Representative mass spectra of PSFZM, PSFZ, and PSFM, recorded under conditions saturated with ^18^O_2_, reveal distinct ^18^O-labeled oxygen reduction products. Time-resolved DEMS traces of selected isotope-labeled fragments, including peroxide/hydroperoxide-related channels and hydroxyl/water-related channels, during intermittent electrolysis over **m** PSFZM, **n** PSFZ, and **o** PSFM. Source data for this figure are provided as a Source data file.

Next, we evaluated the electrocatalytic ORR kinetics of the catalyst in O_2_/N_2_-saturated 1 M Na_2_SO_4_ (Supplementary Fig. 19). Prior to conducting ORR measurements, the collection efficiency of the rotating ring-disk electrode (RRDE) was experimentally calibrated (collection efficiency of *N* = 34.8%, Supplementary Figs. 20 and 21)^34^. RRDE measurements revealed markedly accelerated ORR kinetics for PSFZM (Fig. 3a, b) and delivered a current density of 4.2 mA cm^−^^2^ at 0.07–0.10 V (vs. RHE), approaching the theoretical diffusion-limitation current for 3e^−^ ORR pathway. The electron-transfer number (*n* ≈ 3.0) in the potential range of 0.20–0.60 V confirmed the predominance of the selective 3e^−^ ORR route. In contrast, PSFZ followed a canonical 2e^−^ pathway (*n* ≈ 2) with nearly 100% H_2_O_2_ selectivity, while PSFM exhibited 4e^−^ pathway characteristics (*n* ≈ 3.5) (Fig. 3c). Koutecky-Levich analysis and electrochemically active surface area (ECSA) further validated the high 3e^−^ ORR selectivity and electrochemical activity of PSFZM, with Levich constants of 8.17 and 8.59 mA^−^^1^ cm^2^ s^1/2^ at 0.2 and 0.3 V (vs. RHE), respectively (Supplementary Figs. 22 and 23). Durability tests further confirmed the stable performance of PSFZM, with its crystal structure largely maintained after electrolysis (Supplementary Figs. 24–26).

Electron spin resonance (ESR) spectroscopy confirmed that PSFZM predominantly generates •OH during electrocatalysis, with a peak intensity much higher than that of the other samples (Fig. 3d and Supplementary Fig. 27). Quasi-operando ESR measurements showed that the signal intensity increased with increasing cathodic bias and reached a maximum at 0.2 V versus RHE (Fig. 3e). This directly indicates that •OH originates from the ORR process and is closely correlated with the electrolytic cell voltage. A control experiment in which H_2_O_2_ was added to the electrolyte showed that the cathodic current increased linearly with H_2_O_2_ concentration, confirming that surface-adsorbed H_2_O_2_ is the direct precursor for •OH generation and that PSFZM mediates a confined, domain-level sequential process (Fig. 3f).

To further substantiate this mechanism, in-situ Fourier-transform infrared spectroscopy (FT-IR) was used to clarify the evolution of reaction intermediates (Fig. 3g–k). With increasing cathodic bias, the *OOH and *H_2_O_2_ signals on PSFZ were markedly enhanced, indicating continuous H_2_O_2_ accumulation without subsequent activation (Fig. 3j). By contrast, PSFM exhibited only a weak *OOH signal and almost no detectable H_2_O_2_ feature, consistent with a four-electron (4e⁻) ORR pathway (Fig. 3k). In sharp contrast, PSFZM displayed an O−O stretching band of adsorbed O_2_ at ~1424 cm^−1^; as the potential shifted negatively, characteristic *OOH-related bands subsequently emerged at ~1241 and ~1389 cm^−1^ (Fig. 3i). Notably, the *OOH and *H_2_O_2_ species reached a dynamic formation–consumption equilibrium near 0.2 V. To more clearly distinguish continuous ORR evolution from spatially separated tandem evolution, PSFZM was compared with a physical mixture of PSFZ and PSFM. For PSFZM, the relative intensity ratio of the *H_2_O_2_-related band to the O_2_/*OOH-related band was only 0.38:1, indicating that peroxide did not accumulate as the dominant surface species (Fig. 3g). The nearly six-fold enhancement of the peroxide-related signal in the PSFZ + PSFM physical mixture indicates substantial H_2_O_2_ accumulation, consistent with a conventional decoupled 2e^−^ + 1e^−^ tandem process (Fig. 3h and Supplementary Fig. 28). These results strongly support a confined active-domain-mediated continuous conversion pathway of O_2_ → *OOH → *H_2_O_2_ → •OH, in which the B-site Zn centers serve as the key sites for direct O₂ activation during the 3e⁻ ORR process.

To resolve the operative ORR pathway on PSFZM, we performed isotope-resolved DEMS in ^18^O_2_-saturated electrolyte. The relative mass spectral abundance distribution (Fig. 3l) shows that PSFM exhibits strong responses only at m/z = 18–20, corresponding to a typical 4e^−^ ORR pathway. In contrast, PSFZ displays synchronized signals at m/z = 37 and 38, while remaining silent at m/z = 19 and 20, indicating the direct desorption of *OOH as H_2_O_2_. Time-resolved DEMS measurements (Fig. 3m–o) further reveal that PSFZM exhibits synchronized and reversible responses across all four channels (m/z = 37, 38, 19, and 20) during each potential step. Compared with PSFZ, the H_2_O_2_ signal (m/z = 38) is markedly suppressed, whereas the *OH/ H_2_O signals (m/z = 19/20) are simultaneously enhanced. In a conventional tandem mechanism involving H_2_O_2_ desorption–diffusion–re-adsorption, the m/z = 38 signal must precede the m/z = 19/20 signals and exhibit distinct relaxation times. Therefore, the strictly synchronized responses observed for PSFZM can only originate from the continuous reduction of surface-confined *H_2_O_2_ intermediates at the same active site.

### Quantifying reactive oxygen species (ROS) generation and unveiling the single-site activation mechanism

**Fig. 4:**
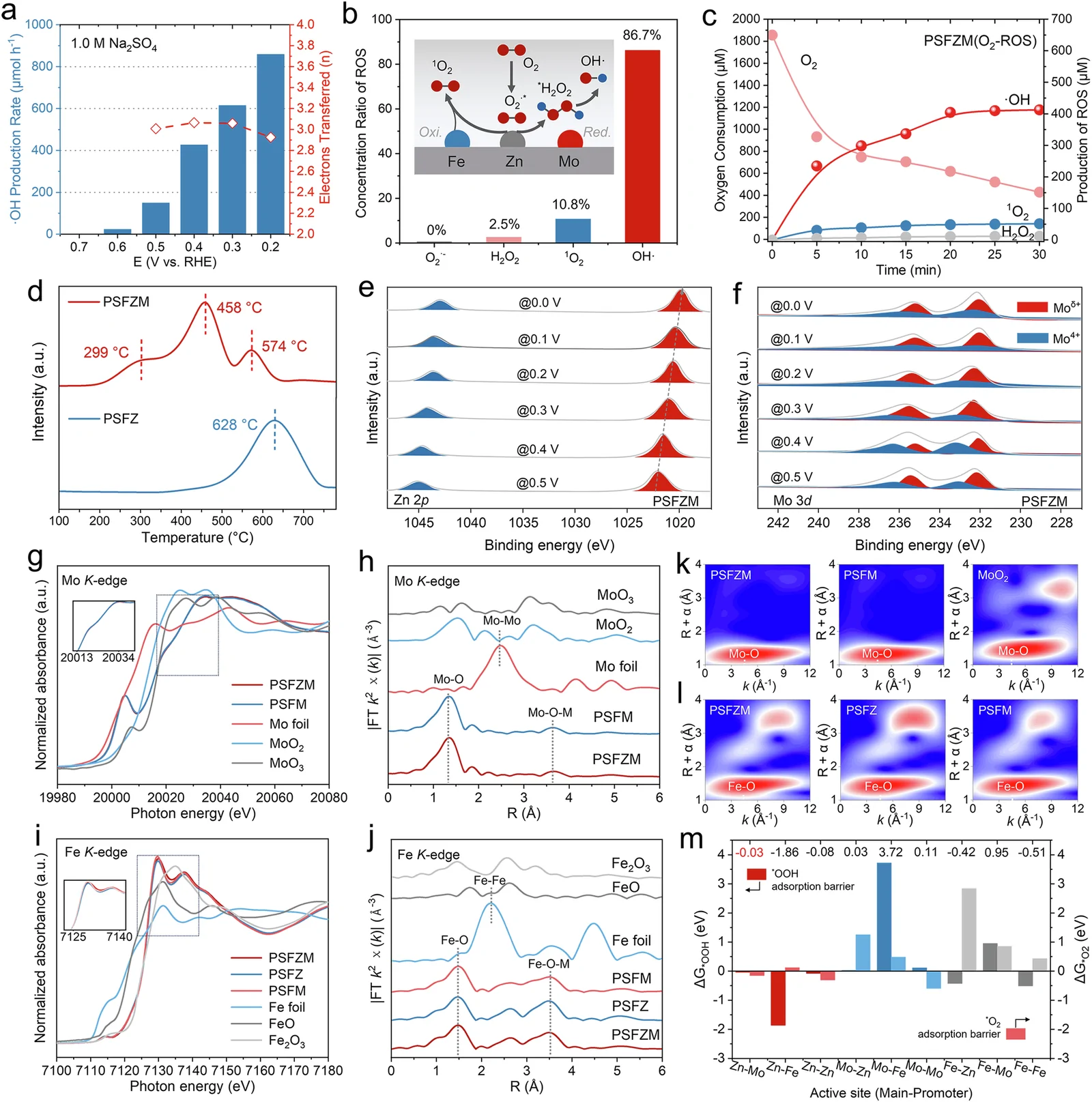
Mechanism of dual co-catalytic sites promoting ROS formation. **a** Benzoic acid fluorescence spectrophotometry, used to detect the amount of •OH free radicals generated. •OH yield at different oxygen reduction voltages. **b** The proportion of ROS generated by PSFZM through 3e^−^ ORR, and the inset shows the single-site active domain mechanism. Colors represent: O (deep red), H (deep blue) **c** Oxygen utilization during 30-min continuous testing. Quantitative determination of the consumed dissolved O_2_ and generated ROS. **d** O_2_-TPD profiles of PSFZM and PSFZ. In-situ XPS spectra of **e** Zn 2*p* and **f** Mo 3*d* in electrochemistry. The shaded areas represent the background-subtracted fitted components of the Zn 2*p* core-level spectra, where the red and blue regions correspond to the Zn 2*p*_*3/2*_ and Zn 2*p*_*1/2*_ peaks, respectively. The integrated areas reflect the relative Zn-related signal intensity under different applied potentials. **g** Mo *K*-edge XANES spectra of PSFZM, PSFM, Mo foil, MoO_2_ and MoO_3_. **h** Mo *K*-edge *k*^*3*^-weighted FT-EXAFS spectra. **i** Fe *K*-edge XANES spectra of PSFZM, PSFZ, PSFM, Fe foil, FeO and Fe_2_O_3_. **j** Fe *K*-edge *k*^*3*^-weighted FT-EXAFS spectra. WT-EXAFS spectra of **k** Mo *K*-edge and **l** Fe *K*-edge. **m** Comparison of free energies of adsorption of intermediate *OOH and O_2_ at B sites in PSFZM. Source data for this figure are provided as a Source data file.

After establishing the pathway, we quantified the efficiency of the ROS generation process (Supplementary Figs. 29 and 30). Using benzoic acid fluorescence probing, PSFZM achieved a high •OH production rate of 821 μmol h^−^^1^ under steady-state electrolysis (Fig. 4a, Supplementary Table 3), which is 2.01 times and 4.56 times higher than that of PSFZ (Supplementary Fig. 31) and PSFM (Supplementary Fig. 32), respectively. Using norfloxacin (NOR) as a model persistent antibiotic, a comprehensive analysis of ROS distribution through free radical trapping revealed that the proportion of •OH was 86.7%, with minor yields of ^1^O_2_ (10.8%) and H_2_O_2_ (2.5%) (Supplementary Figs. 33 and 34), leading to an O_2_-to-ROS conversion efficiency of 37.7% (Fig. 4b, c). This high efficiency is attributed to optimized O_2_ binding, as evidenced by O_2_ temperature-programmed desorption (O_2_-TPD) and electrochemical oxygen insertion/extraction studies (Fig. 4d and Supplementary Fig. 35).

To identify the active site responsible for O_2_ activation, we conducted operando XPS. The Zn 2*p* spectra exhibited a significant negative shift and increased intensity upon applying cathodic potential, indicating that the Zn^δ+^ site serves as the electron-donating center during O_2_ activation (Fig. 4e). In contrast, the Mo 3*d* spectra remained largely unchanged (Fig. 4f), suggesting that Mo primarily plays a structural and electronic modulation role.

Combining the Mo *K*-edge and Fe *K*-edge XANES spectra (Fig. 4g–j and Supplementary Figs. 36–39) with the WT-EXAFS spectra (Fig. 4k, l) revealed that the chemical states and bond lengths of Mo–O and Fe–O in PSFZM are similar to those observed in PSFZ and PSFM. The bond lengths were determined to be 1.77 ± 0.01 Å for Mo-O and 1.95 ± 0.02 Å for Fe–O, indicating that the chemical states of Mo and Fe are unaffected by the special distorted Ruddlesden-Popper structure (Supplementary Tables 4, 5, Supplementary Figs. 40 and 41). The adsorption characteristics and intermediate evolution processes in PSFZM align with those in PSFZ and PSFM. This further supports the conclusion that Zn is the primary adsorption site in PSFZM, dominating the continuous activation of O_2_ at this site.

To clarify the roles of *O_2_ and *OOH at the Zn single-site, we enumerated all possible adsorption configurations based on density functional theory calculations (DFT) (Fig. 4m). The results show that the Zn sites adsorb O_2_, with the most favorable reaction pathway appearing when Zn is coordinated with Mo (Zn–O–Mo). This finding indicates that the initial evolution of O_2_ occurs at the Zn sites, in agreement with the in situ XPS results.

### Elucidation of the activity of high-yield •OH sources

To elucidate the source of the high •OH yield exhibited by PSFZM in the 3e^−^ ORR, we systematically calculated the Gibbs free energy (Δ*G*) of all fundamental steps in the 3e^−^ ORR reaction pathway (Supplementary Fig. 42). We considered a series of local structural motifs, including Zn–O–Fe, Zn–O–Mo, and Zn–O–Zn site arrangements (Supplementary Figs. 43–49), to resolve the evolution of oxygen intermediates and their associated free energy changes in PSFZM and its control samples (PSFZ, PSFM, and PSF).

**Fig. 5:**
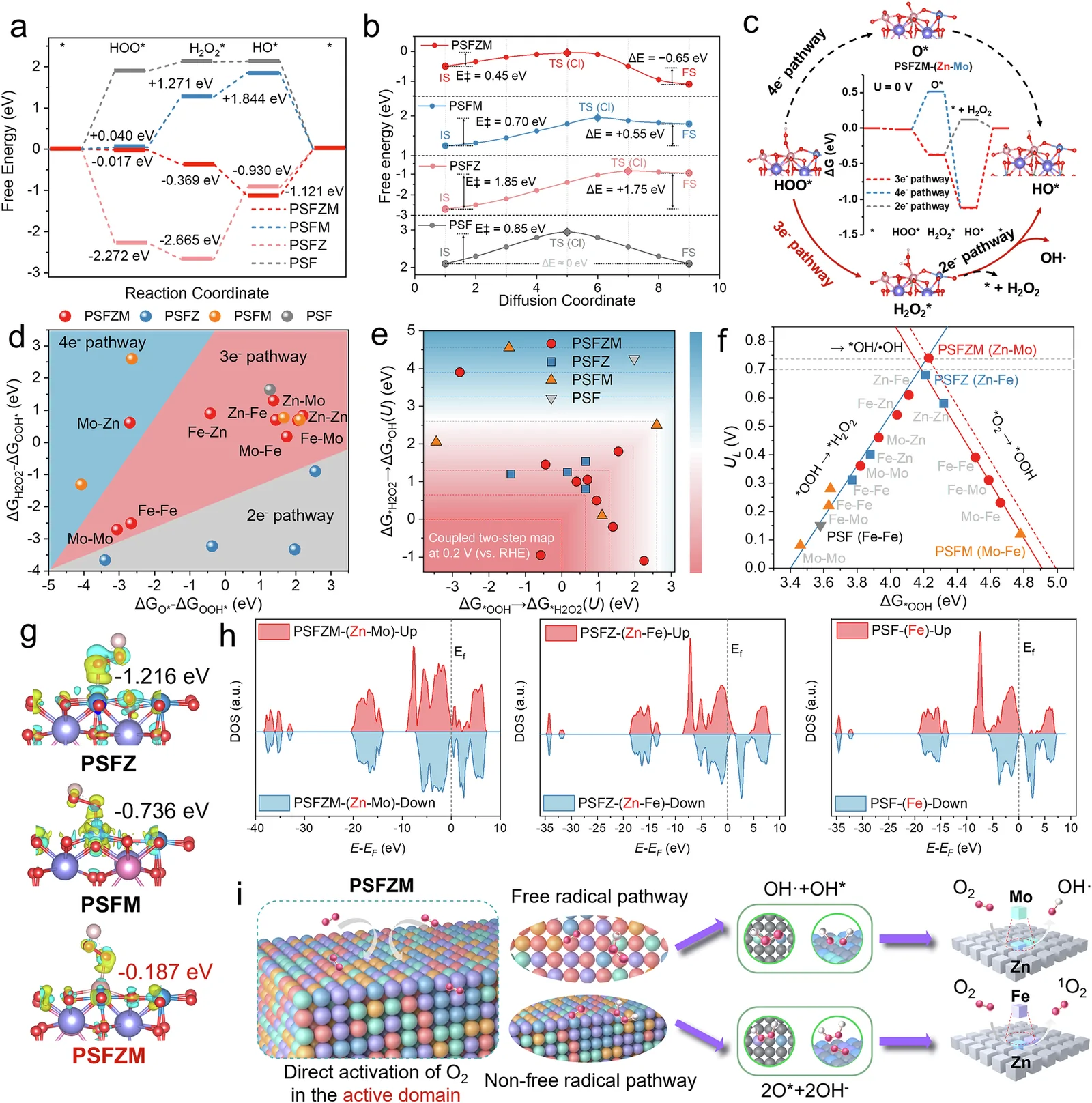
DFT calculations involving each reaction site. **a** Gibbs free energy of the whole reaction pathway for the generation of •OH via three-electron ORR for all samples, and the detailed data are listed in Supplementary Data 1. **b** CI-NEB energy profiles for the rate-determining elementary step on the four candidate catalysts. **c** Structural models and corresponding free energy comparisons of the 3e⁻ ORR and 4e⁻ ORR pathways on the PSFZM catalyst. Colors represent: Pr (gray), Sr (yellow), Fe (purple), Zn (blue), Mo (pink), O (red), H (white). The relevant structures are listed in Supplementary Data 2. **d** Energy differences between reaction intermediates at equilibrium potential. The blue, red, and gray regions indicate active sites for the thermodynamically driven 4e^−^, 3e^−^, and 2e^−^ ORR pathways, respectively. **e** Two-step coupled selectivity map at 0.2 V (vs. RHE). **f** Scaling-relation analysis of intermediate adsorption free energies on dual-atom sites. **g** Side view of the charge density difference for H_2_O_2_ adsorbed on PSFZ/PSFM/PSFZM, the cyan region represents charge depletion, and the yellow region indicates charge accumulation. Colors represent: Pr (gray), Sr (yellow), Fe (purple), Zn (blue), Mo (pink), O (red), H (white). **h** Calculated density of states (DOS) analysis for PSF/PSFZ/PSFZM after H_2_O_2_ adsorption. **i** Schematic diagram of the reaction pathway of O_2_ generating ROS through free radical and non-free radical at the active domain of PSFZM. Colors represent: Pr (pink), Sr (purple), Fe (yellow), Zn (green), Mo (blue), O (red), H (white). Source data for this figure are provided as a Source data file.

We selected the Gibbs free energy of the most favorable active site in each sample for a full-process comparison, and the free-energy landscape constructed for the optimal Zn–O–Mo configuration (Fig. 5a) reveals a four-step reaction mechanism for the 3e⁻ ORR:

Step I: Zn* + O_2_ + H^+^ + e^−^ → Zn-OOH

1 $$({\rm\Delta }G=0.01\mathrm{eV},{{\rm{O}}}_{2}\,\mathrm{adsorption}\,\mathrm{and}\,\mathrm{activation})$$

Step II: Zn-OOH + H^+^ + e^−^ → Zn-H_2_O_2_

2 $$(\Delta {\rm{G}}=-0.38{\rm{eV}},\,*{\rm{Hydrogenation}}\,{\rm{to}}\,{{\rm{H}}}_{2}{{\rm{O}}}_{2})$$

3 $$({\rm\Delta }G=-0.38\,\mathrm{eV},\,*\mathrm{Hydrogenation}\,\mathrm{to}\,{{\rm{H}}}_{2}{{\rm{O}}}_{2})$$

Step III: Zn-HOOH-Mo + e^−^ → Zn-OH + OH^−^

4 $$(\Delta {\rm{G}}=-0.75{\rm{eV}},{{\rm{H}}}_{2}{{\rm{O}}}_{2}\,{\rm{cleavage}})$$

Step IV: Zn-OH → Zn + •OH

5 $$({\rm\Delta }G=-1.12\,\mathrm{eV},\,{\rm\bullet }\mathrm{OH\; liberation})$$

Density functional theory (DFT) calculations show that H_2_O_2_ desorption from the Zn site is an uphill process, that is, a non-spontaneous step with Δ*G* = +0.52 eV. This feature suppresses H_2_O_2_ release and thereby enhances the selectivity toward the 3e^−^ ORR pathway. Among all examined structural motifs, the Zn–O–Mo unit in the material constructs a confined microenvironment that optimizes the orientation of reaction intermediates and their desorption kinetics (Supplementary Figs. 50–65).

We further performed climbing-image nudged elastic band (CI-NEB) calculations to locate the initial state (IS), transition state (TS), and final state (FS) of each model, thereby evaluating the kinetic accessibility of the key O–O bond activation step. The transition-state profiles show that PSFZM exhibits the lowest energy barrier for converting surface-adsorbed H_2_O_2_ into •OH-related intermediates. In contrast, PSFZ, PSFM, and PSF encounter higher kinetic barriers or energetically less favorable final states (Fig. 5b). This result indicates that the enhanced •OH yield on PSFZM does not arise solely from the thermodynamic stabilization of peroxide species, but also from the simultaneous reduction of the kinetic barrier for intradomain peroxide activation. Meanwhile, the cleavage of *H_2_O_2_, identified as the rate-determining step (RDS), is a downhill process on PSFZM, whereas it is uphill on PSFM (0.75 eV), PSFZ (1.73 eV), and PSF (0.01 eV). Therefore, the specific Zn–O–Mo configuration lowers the critical energy barrier, accelerates electron transfer, and rationalizes the experimentally observed high •OH generation rate (Fig. 5c).

Conventional descriptors, such as ΔG_*OOH_, often fail to accurately capture the energetics of downstream product formation. Therefore, we introduced ΔG_*H2O2_–ΔG_*OOH_ and ΔG_*O_–ΔG_*OOH_ as more predictive descriptors for reaction selectivity (Fig. 5d). Mechanistic mapping shows that the PSFZM catalytic site is located in a specific region that favors the formation of H_2_O_2_ and •OH while effectively suppressing excessive reduction toward the *O intermediate. A coupled two-step descriptor map at 0.2 V versus RHE further shows that the Zn–O–Mo sites of PSFZM occupy the optimal region where peroxide formation and peroxide activation are balanced (Fig. 5e). In contrast, PSFZ stabilizes peroxide intermediates but lacks sufficient activation capability, leading to H₂O₂ accumulation, while PSFM promotes stronger O–O activation but does not efficiently retain the peroxide intermediate. In Fig. 5f, the two volcano branches describe the thermodynamic limitations associated with *H_2_O_2_ and *OOH formation, respectively. The volcano apex approaches the equilibrium potential of the O_2_/H_2_O_2_ couple (U*°* = 0.70 V vs. RHE), defining the minimum theoretical overpotential imposed by the scaling relationship. The Zn–O–Mo motif in PSFZM lies near this optimum and gives the highest limiting potential among all examined motifs (ΔG_*OOH_ ≈ 4.28 eV, U_*L*_ ≈ 0.749 V).This position indicates that the Zn–O–Mo active domain effectively balances *OOH stabilization with *H_2_O_2_ activation, thereby mitigating the conventional scaling constraint and promoting the confined conversion of surface-retained *H_2_O_2_ toward •OH.

Charge-density difference and DOS analyses further corroborate these findings (Supplementary Figs. 66–68). The Zn-O-Mo domain exhibits a smooth *OOH interaction energy (–0.187 eV), facilitating •OH desorption and preventing over-oxidation to *O (Fig. 5g). Meanwhile, Mo incorporation significantly upshifts the Zn *d*-band center (from –5.693 eV in PSF to –3.028 eV in PSFZM), enabling balanced intermediate adsorption and faster ORR kinetics (Fig. 5h). In addition, we also performed calculations on non-radical pathway and found a small amount of ^1^O_2_ product in the product detection (Fig. 5i and Supplementary Figs. 69, 70). In summary, the Zn-O-Mo configuration in PSFZM has the optimal energy potential for balancing H_2_O_2_ formation and decomposition into •OH, indicating that this single-site active-domain strategy can effectively avoid the energy consumption caused by intermediate migration and rate mismatch in the conventional 2e^–^ ORR with 1e^–^ H_2_O_2_ tandem mechanism.

### Practical decontamination performance and system engineering

The ultimate test of our catalyst lies in its practical efficacy. Degradation experiments showed that PSFZM achieved an 88.6% NOR removal rate in 30 min and a 63.4% total organic carbon (TOC) mineralization rate in 100 min, which are 2.1 times and 4.1 times higher than those of PSFZ and PSFM, respectively (Supplementary Figs. 71–75). This rapid degradation performance highlights the effectiveness of continuously generated •OH radicals in deep oxidation. Furthermore, the system exhibited broad-spectrum activity against various pollutants, including tetracycline (TC), phenol, bisphenol A (BPA), and rhodamine B (RhB), demonstrating its versatile decontamination capabilities (Supplementary Fig. 76). Notably, PSFZM also demonstrated robust stability over multiple cycles, proving its potential for practical applications (Supplementary Figs. 77 and 78). The influence of common inorganic ions and a wide pH range on degradation performance was negligible, indicating the strong adaptability and mechanistic robustness (Supplementary Figs. 79–85).

To elucidate the degradation mechanism, liquid chromatography-mass spectrometry (LC-MS) analysis was performed. After 5 min of electrolysis, •OH radicals initiated the cleavage of the piperazine ring, followed by oxidation and demethylation reactions, generating an intermediate product with m/z = 251, which was ultimately mineralized into inorganic products (Supplementary Fig. 86). This confirms the complete degradation of NOR via the ROS pathway mediated by the PSFZM catalyst.

**Fig. 6:**
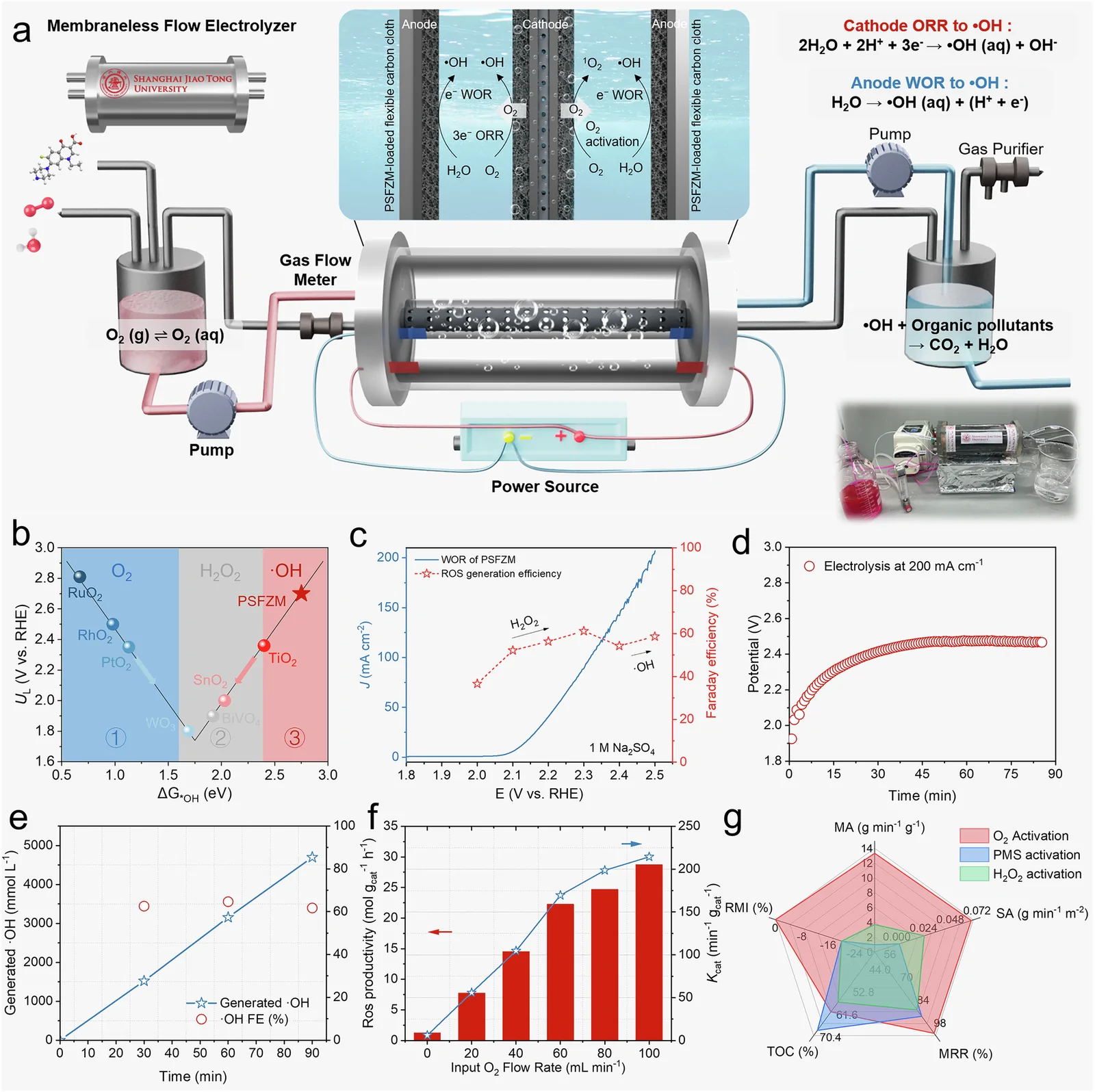
Flowing membraneless bifunctional electrode electrolyzer performance. **a** Schematic diagram of the actual operation of the cylindrical membraneless electrolyzer supported by PSFZM electrodes. Colors represent: C (gray), H (white), O (red), N (blue), F (green). **b** Volcano plot of the theoretical adsorption free energies of *OH (ΔG_*OH_) for the products synthesis on different materials through WOR. **c** Polarization I-V curves of PSFZM (WOR) measured in O_2_-saturated 1.0 M Na_2_SO_4_, and the corresponding FE profiles obtained by the titration method. **d** The stability profile for PSFZM||PSFZM full cell (*E*_cell_) measured at 200 mA cm^−2^. **e** The •OH yield and FE in full cell under PSFZM || PSFZM configuration. **f** ROS generation rate and first-order kinetics of RhB degradation under different input O_2_ flow rates. **g** The radar chart was established to compare with the H_2_O_2_ activation and PMS activation systems. Source data for this figure are provided as a Source data file.

To transcend the limitations of conventional batch reactors, we engineered a membrane-free cylindrical electrolyzer that integrates the PSFZM cathode (3e⁻ ORR) with an identical PSFZM anode (for synergistic oxidation) (Fig. 6a). We determined the •OH generation efficiency of the e^−^ water oxidation reaction (WOR) through DFT calculations and electrochemical testing of the anode (Fig. 6b–d). This design creates a stable gas-liquid-solid triple-phase interface, enabling direct utilization of gaseous O_2_ and eliminating mass-transfer bottlenecks that typically hinder reactor efficiency (Supplementary Figs. 87–89). In this setup, the 3e^−^ ORR || e^−^ WOR full-cell system delivered a peak FE of 64.7% for •OH generation (Fig. 6e).

Applied to RhB degradation, the system exhibited rapid pollutant removal across a range of flow rates, with a degradation rate of 169.5 min^−^^1^ g_cat_^−1^ (Supplementary Fig. 90). Enhanced oxygen flow was directly correlated with increased •OH output and pollutant degradation, underscoring the importance of O_2_ mass transfer in enhancing ORR activity (Fig. 6f). A comparative radar plot (Fig. 6g) benchmarked PSFZM against conventional H_2_O_2_ and peroxymonosulfate (PMS) activation systems. PSFZM showed competitive values in mass activity (3.62 times and 13.56 times higher, respectively), surface activity (2.72 times and 3.14 times), TOC removal efficiency, and raw material economy (Supplementary Table 6)^35–37^. Furthermore, PSFZM exhibited a metal retention rate of 99.9%, ensuring long-term environmental compatibility and electrode reusability.

Finally, to evaluate the oxygen conversion efficiency, we compared the ROS generation rates across different systems. The PSFZM electrode achieved an ROS production rate of 28.8 mol g_cat_^−1^ h^−1^ in a membrane-free cylindrical electrolytic cell, demonstrating efficient ROS generation under the present operating conditions^38–40^. At high operating current densities (200 mA cm⁻^2^), the PSFZM-based electrolyzer showed competitive performance among reported ORR-driven oxygen activation systems, establishing it as a scalable, energy-efficient, and environmentally sustainable solution for advanced wastewater remediation (Supplementary Fig. 91 and Supplementary Table 7).

## Discussion

In summary, we demonstrate the potential of a single-site active domain strategy in perovskite oxide catalysts to enable efficient and direct 3e⁻ ORR for continuous and sustainable •OH generation. The PSFZM catalyst, with its rationally designed Zn^δ⁺^ and Mo centers, allows for efficient oxygen activation and transformation, bypassing the need for intermediate desorption and migration, and overcoming the kinetic bottlenecks of conventional systems. This approach enables efficient •OH generation while improving the O_2_-to-ROS conversion efficiency under the present electrochemical oxidation conditions. As a result, the PSFZM electrode achieves a •OH generation rate of 821 μmol h^−1^. Benchmarking against representative electrochemical O_2_-activation systems further highlights the competitive ROS production performance of PSFZM. Moreover, by integrating this catalyst into a membrane-free flow electrolyzer, we demonstrate its scalability and performance in a real-world setting, achieving a •OH generation efficiency and rate of 64.7% and 28.8 mol g_cat_^−1^ h^−1^, respectively, at a high current density of ~200 mA cm^−2^. The findings suggest that this integrated combination of atomic-level catalyst design and reactor engineering holds significant promise for transforming the future of wastewater treatment, offering a pathway for the sustainable remediation of complex organic pollutants in water.

## Methods

### Materials synthesis

PSFZM perovskite samples were prepared by electrospinning. The analytical grade starting material is used directly without any additional purification. Therefore, stoichiometric amounts of Pr(NO_3_)_3_·4H_2_O (AR, Sinopharm Group Chemical Reagent Co., Ltd) (0.399 g), Sr(NO_3_)_2_ (AR, Sinopharm Group Chemical Reagent Co., Ltd) (0.212 g), Fe(NO_3_)_3_·9H_2_O (AR, Sinopharm Group Chemical Reagent Co., Ltd) (0.202 g), Zn(NO_3_)_2_·6H_2_O (AR, Sinopharm Group Chemical Reagent Co., Ltd) (0.074 g), and (NH_4_)_6_Mo_7_O_24_·4H_2_O (AR, Sinopharm Group Chemical Reagent Co., Ltd) (0.044 g) were dissolved in 12 mL of N, N-dimethylformamide (DMF) (RG, Adamas). Then, 1 g polyacrylonitrile (PAN) (AR, Adamas) was gradually added to the precursor solution after all metal nitrates were dissolved by immersion in a water bath at 80 °C. The mixture was stirred at a controlled temperature of 80 °C for 20 h to ensure complete dissolution and homogenization of the nitrate mixture and PAN. The prepared viscous precursor gel was filled into a plastic syringe with a stainless-steel needle. A plastic syringe was fixed to an electrospinning machine, and the high voltage power supply was adjusted to ensure that the fibers were formed by electrostatic force and deposited on the tin foil of a synchronously rotating metal drum. Finally, after calcination in air at 1000 °C for 24 h, Pr_1.0_Sr_1.0_Fe_0.5_ Zn_0.25_Mo_0.25_O_4-δ_ catalysts were obtained, including Pr_1.0_Sr_1.0_Fe_1.0_O_4-δ_ with different precursors, Pr_1.0_Sr_1.0_Fe_0.75_Zn_0.25_O_4-δ_ and Pr_1.0_Sr_1.0_Fe_0.75_Mo_0.25_O_4-δ_ nanofibers.

### Physical characterizations

Phase identification was performed by XRD on a Bruker D8 Advance diffractometer using Cu Kα radiation. The diffraction patterns were collected from 20 to 80° at a scan rate of 2° min^–1^ to assess the crystallinity and phase purity of the perovskite samples. The morphology of the catalysts was examined by SEM (Thermo Fisher Scientific). TEM and HRTEM images were acquired on a JEOL JEM-2100F microscope to resolve the nanoscale morphology and lattice fringes. Atomic-resolution aberration-corrected TEM/STEM imaging was carried out on a Spectra 300 field-emission aberration-corrected transmission electron microscope (Thermo Fisher Scientific) at the Zhangjiang Public Instrument Platform of Shanghai Jiao Tong University, Shanghai, China. Elemental distributions were obtained by EDS. XPS measurements were conducted on a Kratos AXIS UltraDLD-600 W spectrometer with Al Kα radiation (1486.6 eV), and all binding energies were calibrated against the adventitious carbon C 1*s* peak at 284.8 eV. EPR spectra were recorded using a Bruker EPR A300 spectrometer, whereas solution-phase spin-trapping measurements were performed on a Bruker Micro-ESR system. O_2_-TPD profiles were collected using a TP-5080 chemisorption system.

### Figure preparation

The schematic illustrations were created by the authors using Maxon Cinema 4D under an institutional software license. All graphical elements were originally generated for this work, and no third-party copyrighted templates, stock images, or externally sourced graphical assets were used.

### In-situ FTIR spectrometry

The in-situ FTIR measurements were conducted using a combination of the TENSOR II FTIR spectrophotometer and a CHI 760 electrochemical workstation. The array electrodes obtained were employed directly as the working electrode, with Hg/HgO serving as the reference electrode and a graphite rod as the counter electrode. In-situ FTIR spectra were collected under various electrolyte configurations during LSV or potentiation tests.

### Rotating ring disk electrode (RRDE) testing

Half-cell measurements were performed in a standard three-electrode configuration (AUT87986, Metrohm Autolab B.V., Netherlands) with a graphite rod as the counter electrode and Hg/HgO (CH Instruments) as the reference electrode. Catalyst inks were prepared by dispersing 10 mg of catalyst and 3 mg of acetylene carbon black (conductive additive) in 1 mL of binder solution (ethanol: isopropyl alcohol: 5 wt% Nafion solution (Sigma-Aldrich) = 5:5:1, volumetric ratio), followed by tip sonication. For electrochemical testing, 5 µL of each ink was drop-coated onto a glassy carbon disk electrode and evaluated in an O_2_-saturated 1 M Na_2_SO_4_ (RG, Adamas) solution (pH = 7.8 ± 0.2, electrolytes were prepared within two weeks prior to use and stored at room temperature, 25 °C) at 10 mV s⁻^1^. Reversible hydrogen electrode (RHE) calibration was performed at 1 mV s^−^^1^ in an Ar-saturated aqueous solution. The number of electrons transferred (n) and peroxide yield (%) were calculated by the followed equations:

6 $$n=4\times \,\frac{{I}_{d}}{{I}_{d}+{I}_{r}/N}$$

7 $${{H}_{2}O}_{2}=200\times \,\frac{{I}_{r}/N}{{I}_{d}+{I}_{r}/N}$$

where *I*_*d*_ and *I*_*r*_ are the disk current and ring current, respectively, and *N* is the collection efficiency.

The theoretical diffusion-limited current density for a formal 3e^−^ ORR process was calculated using the Levich equation:

8 $${j}_{{\rm{L}}}=0.620{nF}{D}_{{{\rm{O}}}_{2}}^{2/3}{\nu }^{-1/6}{C}_{{{\rm{O}}}_{2}}{\omega }^{1/2}$$

where ${j}_{{\rm{L}}}$ is the diffusion-limited current density, n is the number of electrons transferred per O_2_ molecule, F is the Faraday constant, ${D}_{{{\rm{O}}}_{2}}$ is the diffusion coefficient of dissolved oxygen, ν is the kinematic viscosity of the electrolyte, ${C}_{{{\rm{O}}}_{2}}$ is the dissolved oxygen concentration, and ω is the angular rotation rate. For the RRDE measurement at 1600 rpm,

9 $$\omega=2\pi \times \frac{1600}{60}=167.6\,{\rm{rad}}\,{{\rm{s}}}^{-1}$$

Using the transport parameters for O_2_-saturated 1.0 M Na_2_SO_4_ under our experimental conditions, the calculated theoretical diffusion-limited current density for a 3e^−^ ORR process is:

10 $${j}_{{\rm{L}},3{{\rm{e}}}^{-}}=4.5\,{\rm{mA}}\,{\rm{c}}{{\rm{m}}}^{-2}$$

The measured disk current density of PSFZM at 0.07–0.10 V versus RHE was 4.2 ± 0.2 mA cm⁻^2^, corresponding to approximately 93 ± 5% of the calculated 3e^−^ diffusion-limited current. Therefore, the phrase “approaching the theoretical limit” refers specifically to the calculated Levich mass-transfer limit under the defined RRDE conditions, not to an absolute theoretical limit for •OH generation.

Unless otherwise stated, the reported optimal values correspond to the best-performing result obtained under the optimized experimental conditions. Independent measurements were performed to confirm reproducibility, and the number of measurements is indicated in the corresponding figure captions or “Methods” section. When applicable, representative optimal values are shown together with the mean ± standard deviation to avoid selective reporting.

All potentials were converted to the RHE scale. A Hg/HgO reference electrode with 1.0 M KOH as the internal filling solution was used. The conversion was performed according to:

11 $${{\rm{E}}}_{{\rm{RHE}}}={{\rm{E}}}_{{\rm{Hg}}/{\rm{HgO}}}+0.098+0.0591\,\times {\rm{pH}}$$

Because the electrolyte used in this work was O_2_-saturated 1.0 M Na_2_SO_4_ with a measured pH of 7.8 at room temperature (25 °C), the conversion factor was calculated to be 0.559 V. Therefore, all potentials measured versus Hg/HgO were converted to the RHE scale using:

12 $${{\rm{E}}}_{{\rm{RHE}}}={{\rm{E}}}_{{\rm{Hg}}/{\rm{HgO}}}+0.559\,{\rm{V}}$$

The Hg/HgO reference electrode was calibrated before electrochemical measurements using a H_2_-saturated electrolyte and a clean Pt electrode. The calibration was repeated at least three times, and the electrode drift before and after the measurements was less than 2 mV.

### ECSA estimation

The ECSA was estimated from the electrochemical C_dl_. Cyclic voltammetry was performed in a non-faradaic potential region in 1.0 M Na_2_SO_4_ electrolyte at room temperature (25 °C), with a catalyst loading of 0.202 mg cm^−^^2^. For PSFZM, CV curves were collected at scan rates of 5, 10, 15, 20, 30, 50 and 100 mV s⁻^1^. The capacitive current density was calculated from the difference between the anodic and cathodic current densities at a fixed potential:

13 $$\Delta j=\frac{{j}_{a}-{j}_{c}}{2}$$

where ${j}_{a}$ and ${j}_{c}$ are the anodic and cathodic current densities, respectively. The C_dl_ value was determined from the slope of the linear fitting between Δj and the scan rate:

14 $$\Delta j={C}_{{dl}}\times v$$

where v is the scan rate. The ECSA was then calculated according to:

15 $${\rm{ECSA}}=\frac{{C}_{{dl}}}{{C}_{s}}$$

where ${C}_{s}$ is the specific capacitance of a smooth surface and was taken as 40 μF cm^−^^2^.

### ROS determination and quantification

The electrochemical testing to identify the accumulated amount of ROS was conducted by typical H-type cell configuration by using a Nafion membrane (Nafion 211, FuelCell Store) as a separator. Nafion 211 membrane (thickness: 25.4 μm) was used as the proton-exchange membrane. Prior to use, the membrane was rinsed with deionized water, hydrated in deionized water at 80 °C for 1 h, treated in 0.5 M H_2_SO_4_ at 80 °C for 1 h, and then thoroughly rinsed with deionized water until the rinsing solution reached neutral pH. The pretreated membrane was stored in deionized water before cell assembly.

#### •OH production and quantification

The content of •OH generated during ORR was detected by the photoluminescence steady-State fluorescence spectroscopy (PL) signal of hydroxybenzoic acid (hydroxycoumarin), obtained from the capture of •OH by benzoic acid, which indicates the concentration of •OH. The details are as follows: 100 mL H_2_O containing 100 mg benzoic acid (40 mg coumarin) was mixed; the suspension was adjusted to pH 3.5. Then, at different time intervals, a 3-mL solution was extracted and measured by PL spectroscopy to calculate the contents of •OH (excitation wavelength: 300 nm of hydroxybenzoic acid and 332 nm of hydroxycoumarin).

#### Calibration method for the determination of •OH concentration by fluorescence method of benzoic acid and coumarin

For BA-based •OH quantification, the electrolyte contained BA under the same pH, ionic strength, and electrolysis conditions as those used for ORR measurements. Aliquots were collected at fixed time intervals, filtered, and analyzed by steady-state fluorescence spectroscopy. External calibration curves were established using standard hydroxybenzoic acid solutions prepared in the same electrolyte matrix. The fluorescence intensity was corrected by subtracting the blank signal obtained without applied bias or without catalyst. The apparent •OH concentration was obtained from the calibration curve and converted into the amount of •OH according to

16 $${n}_{\bullet {\rm{OH}}}={C}_{\bullet {\rm{OH}}}V$$

Where *C*_•OH_ is the calibrated apparent •OH-equivalent concentration and *V* is the electrolyte volume. The •OH production rate was calculated from the slope of *n*_•OH_ versus electrolysis time.

For CM-based validation, standard 7-hydroxycoumarin solutions were used to construct calibration curves under the same electrolyte conditions. Because the 7-hydroxycoumarin product can be further oxidized in strongly oxidative systems, CM-derived values were used only as an independent verification of the trend rather than as the primary basis for Faradaic-efficiency calculation.

#### H_2_O_2_ production and quantification

The amount of H_2_O_2_ formed during electrolysis was determined using a Ce(IV) sulfate colorimetric assay. Briefly, aliquots of the electrolyte were mixed with an acidic Ce(IV) sulfate solution, in which H_2_O_2_ reduces Ce^4+^ to Ce^3+^ and is simultaneously oxidized to O_2_. The consumption of Ce^4+^ was quantified from the decrease in absorbance at 316 nm using ultraviolet-visible spectroscopy.

17 $$2C{e}^{4+}+{{\rm{H}}}_{2}{{\rm{O}}}_{2}\to 2C{e}^{3+}+2{{\rm{H}}}^{+}+{{\rm{O}}}_{2}$$

On the basis of the 2:1 stoichiometric ratio between Ce^4+^ and H_2_O_2_, the H_2_O_2_ concentration was calculated as:

18 $${{\rm{C}}}_{{\rm{H}}2{\rm{O}}2}=\frac{{\Delta {\rm{C}}}_{{\rm{Ce}}4+}}{2}$$

where ${\Delta {\rm{C}}}_{{\rm{Ce}}4+}$ denotes the concentration of Ce^4+^ consumed after reaction with H_2_O_2_.

The Ce(IV) working solution was freshly prepared by dissolving 33.2 mg of Ce(SO_4_)_2_ in 100 mL of 0.5 M H_2_SO_4_, giving a Ce^4+^ concentration of 1.0 mM. Calibration was performed by adding standard H_2_O_2_ solutions with known concentrations to the Ce(IV) reagent under identical conditions, followed by recording the absorbance at 316 nm. The calibration curve was constructed within the linear response range of approximately 0.1–0.8 mM Ce^4+^. Electrolyte samples collected after electrolysis were diluted when necessary to fall within this range, and their H_2_O_2_ concentrations were obtained from the corresponding absorbance change.

#### ^1^O_2_ production and quantification

The generation rate of ^1^O_2_ during electrochemical O_2_ activation is determined by FFA (*k*^1^O_2_, FFA = 1.2 × 10^8 ^M^−1^s^−1^). We assume that the physical quenching of ^1^O_2_ by FFA is negligible, and the steady-state concentration of ^1^O_2_ can be calculated by the following equation:

19 $$\frac{{\rm{d}}\left[{\rm{FFA}}\right]}{{\rm{d}}t}=-{k}_{{{\rm{O}}}_{2,{\rm{FFA}}}}{\left[{{}^{1}{\rm{O}}}_{2}\right]}_{{\rm{ss}}}\left[{\rm{FFA}}\right]$$

20 $${\left[{{}^{1}{\rm{O}}}_{2}\right]}_{{\rm{ss}}}=\frac{{k}_{{\rm{FFA}}}}{{k}_{{{\rm{O}}}_{2,{\rm{FFA}}}}}$$

Accordingly, the formation rate of ^1^O_2_ in the electrocatalytic oxygen reduction process was calculated by the following equation:

21 $${R}_{1{\rm{O}}2,{form}}={\left[{{}^{1}{\rm{O}}}_{2}\right]}_{0}\left({k}_{d}+{k}_{1O2,\mathrm{FFA}}\left[\mathrm{FFA}\right]\right)$$

where *k*_d_ = 2.7 × 10^4 ^s^−1^ is the physical quenching rate constant by solvent (D_2_O/H_2_O = 0.95/0.05, *k*_d, H2O_ = 2.5 × 10^5 ^s^−1^ and *k*_d, D2O_ = 1.6 × 10^4 ^s^−1^).

### Calculation of apparent Faraday efficiency

The apparent Faradaic efficiency for cathodic •OH generation through the formal 3e^−^ ORR pathway was calculated as

22 $$F{E}_{\bullet \mathrm{OH}}=\frac{3F{n}_{\bullet \mathrm{OH}}}{Q}\times 100\%$$

where *F* is the Faraday constant, *n*_•OH_ is the amount of •OH determined from the BA calibration, and $Q=\int I{dt}$ is the total cathodic charge passed during electrolysis. For H_2_O_2_, the corresponding Faradaic efficiency was calculated as

23 $${\mathrm{FE}}_{{{\rm{H}}}_{2}{{\rm{O}}}_{2}}=\frac{2F{n}_{{{\rm{H}}}_{2}{{\rm{O}}}_{2}}2F{n}_{{{\rm{H}}}_{2}{{\rm{O}}}_{2}}}{Q}\times 100\%$$

For the paired full-cell test, the reported value is defined as an overall electron-to-•OH generation efficiency rather than a single-electrode Faradaic efficiency, because •OH can be generated through cathodic ORR and anodic water oxidation.

The oxygen-utilization efficiency was calculated from the oxygen balance:

24 $${\eta }_{{{\rm{O}}}_{2}\to {\rm{ROS}}}=\frac{{n}_{{{\rm{O}}}_{2},{\rm{ROS}}}}{{n}_{{{\rm{O}}}_{2},{\rm{consumed}}}}\times 100\%$$

Here, *n*_O2, consumed_ was determined from the decrease in dissolved O_2_ after blank correction, and *n*_O2, ROS_ was obtained by summing the O_2_-equivalent molar amounts of detected •OH, H_2_O_2_ and ^1^O_2_. The ROS selectivity was calculated as

25 $${S}_{i}=\frac{{n}_{{{\rm{O}}}_{2},i}}{\sum {n}_{{{\rm{O}}}_{2},i}\sum {n}_{{{\rm{O}}}_{2},i}}\times 100\%$$

Calibration curves were repeated at least three times. The limit of detection and limit of quantification were calculated as $3{\sigma }_{{\rm{blank}}}/k$ and $10{\sigma }_{{\rm{blank}}}/k$, respectively, where k is the slope of the calibration curve. Errors are reported as standard deviations from independent experiments, and propagated uncertainties were used for calculated efficiencies.

### DFT calculation method

Spin-polarized density functional theory (DFT) calculations were performed using the Vienna Ab Initio Simulation Package (VASP), employing the projector-augmented wave (PAW) method^41,42^. Electron exchange-correlation effects were treated within the generalized gradient approximation (GGA) using the Perdew-Burke-Ernzerhof (PBE) functional^43^. To account for strong electron correlations in Fe 3*d*-orbitals, the GGA + *U* approach was applied with an effective Hubbard parameter of *U* = 5.3 eV. A plane-wave energy cutoff of 520 eV was used, and Brillouin zone integration was carried out with a 5 × 5 × 1 Monkhorst-Pack *k*-point grid, corresponding to a spacing of 0.036 Å^−^^1^ for perovskite surface calculations^44^. To simulate aqueous environments, implicit solvation effects were incorporated via the VASP sol method, which accounts for water interactions with surfaces and adsorbed intermediates. Structural optimizations were conducted until atomic forces fell below 0.02 eV Å^−1^, with energy convergence set to 10^−^^6^ eV per atom. A vacuum layer of ~20 Å along the *c*-axis was included to minimize periodic boundary interactions^45,46^.

We note that DFT, MD, and FEM models necessarily idealize the catalyst structure and simplify the solvent, applied potential, and dynamic surface reconstruction that occur under catalytic conditions; accordingly, the calculated energetics and field distributions are used to rationalize the experimental trends rather than to provide a quantitatively exact description of the active site.

### Electrode preparation for membraneless electrolyzer

For PSFZM (3e^−^ ORR) || PSFZM (e^−^ WOR) testing, catalyst ink was prepared by drop coating by dispersing 100 mg of catalyst and 30 mg of carbon black in 100 mL of solvent (50:50 = ethanol: isopropanol, volume ratio), using 5% Nafion solution as a binder (600 μL). The ink was evenly sprayed onto commercial carbon cloth (AvCarb P50, FuelCell Store) with a spray gun and used as the electrode after drying with a loading density of 0.1 mg cm^−2^. We used a circulation pump and an electrolyte reservoir to supply the electrolyte liquid to the membrane-free electrolytic cell (volume capacity: 6 L).

### Determination of intermediate products

Norfloxacin concentration was determined by UV-visible spectrophotometry with a maximum absorption wavelength of *λ* = 278 nm. Liquid chromatography-mass spectrometry (LC-MS, Bruker) was used to detect the intermediate products in the degradation process of norfloxacin, mainly using an SB-C18 reversed-phase column (5 μm × 4.6 mm × 250 mm), a methanol:water gradient elution mobile phase, an injection volume of 5 μL, a flow rate of 0.25 mL min^−1^, and an electrospray ionization (ESI) source of positive ion scanning.

### Degradation performance and stability test method

The target simulated wastewater is antibiotics (tetracycline, TC; norfloxacin, NOR), phenols (phenol, Phenol; bisphenol A, BPA), and dyes (rhodamine B, RhB). Take 20 mg L^−1^ simulated wastewater mother liquor to quantitatively prepare a certain concentration of Na_2_SO_4_ electrolyte. The experiment is usually carried out at room temperature (25 °C). The specific experimental steps are as follows: the prepared ink is evenly coated on 1 × 1 cm^2^ carbon paper, and after drying, it is used as a working electrode, and the reaction time is controlled by chronoamperometry. Samples are taken at regular time intervals and filtered with a 0.45 μm filter membrane. Usually, 1.0 mL of electrolyte solution is measured in a liquid phase test bottle, and 0.5 mL of methanol is added to terminate the reaction; 1.5 mL of solution is measured in a cuvette, and the absorbance is quickly tested with a UV-visible spectrophotometer (UV-6100, Mapada). The C/C_0_-t curve is drawn according to the simulated wastewater concentration at different time points in the degradation reaction, and the degradation rate is calculated by the corresponding kinetics to determine the activation performance of O_2_ activation to produce ROS. In the cyclic stability test, the catalyst samples are reused many times. Generally, after the reaction is completed, the used catalyst is washed several times with deionized water and ethanol, and then the recovered catalyst sample is placed in a 60 °C constant temperature drying oven for 24 h for use.

### X-ray absorption spectroscopy measurements

X-ray absorption fine structure (XAFS) measurements were carried out at the Taiwan Photon Source (TPS) of the National Synchrotron Radiation Research Center (NSRRC, Hsinchu, Taiwan) using the TPS 44 A quick-scanning X-ray absorption spectroscopy (QXAFS) beamline. The synchrotron radiation was generated from a 3.0 GeV storage ring operated at a typical ring current of ~400 mA. The incident X-ray beam was monochromatized using a water-cooled Si(111) double-crystal monochromator, and the beam was further collimated and focused by Rh-coated mirrors.

At the sample position, the X-ray beam size was approximately 1–2 mm in the horizontal direction and 0.3–0.5 mm in the vertical direction, ensuring a high signal-to-noise ratio and sufficient energy resolution for XAFS measurements. X-ray absorption near-edge structure (XANES) and extended X-ray absorption fine structure (EXAFS) spectra at the corresponding absorption edges were collected.

Depending on the concentration of the target element, XAFS spectra were recorded in either transmission mode or fluorescence mode using ionization chambers or a solid-state fluorescence detector, respectively. The energy calibration was performed by simultaneously measuring the corresponding metal foil standards, with the first inflection point of the metal K-edge defined as the reference energy.

To assist in the determination of the oxidation state and local coordination environment of the target element, several reference compounds were measured under identical experimental conditions, including metal foils, oxide references, and coordination compounds. All measurements were conducted at room temperature (25 °C). The XAFS data were processed and analyzed using the Athena and Artemis software packages, including background subtraction, normalization, Fourier transformation, and EXAFS fitting.

### Electrochemical in situ X-ray photoelectron spectroscopy

Electrochemical in situ X-ray photoelectron spectroscopy (XPS) measurements were performed using a customized electrochemical cell integrated into an ultra-high-vacuum (UHV) XPS system. The electrochemical cell was designed with an X-ray transparent window and an electron-permeable interface, enabling real-time monitoring of surface chemical states under electrochemical control. The working electrode consisted of the PSFZM sample deposited on a conductive substrate, while a Pt wire and an Hg/HgO electrode were used as the counter and reference electrodes, respectively. All potentials reported in this work were converted to the reversible hydrogen electrode (RHE) scale unless otherwise stated.

XPS spectra were collected using monochromatic Al Kα radiation (hν = 1486.6 eV). The binding energy scale was calibrated using the C 1*s* peak at 284.8 eV. During the in-situ measurements, the applied potential was stepwise controlled in the range of 0–0.5 V, and XPS spectra were recorded after stabilization at each potential to ensure electrochemical equilibrium. Core-level spectra of Zn 2*p*, Mo 3*d*, O 1*s*, and other relevant regions were systematically collected to track the evolution of oxidation states and surface chemical environments.

To minimize beam-induced damage and electrolyte evaporation effects, the X-ray flux and acquisition time were carefully optimized. All spectra were processed using standard background subtraction and peak fitting procedures, and the relative chemical states were quantified based on integrated peak areas corrected by atomic sensitivity factors.

### Calibration of the RRDE collection efficiency (N)

The collection efficiency N of the RRDE was experimentally calibrated using the [Fe(CN)_6_]^3–^/^4–^ redox couple in N_2_-saturated electrolyte, following the protocol described previously¹. The calibration electrolyte consisted of 10 mM K_3_[Fe(CN)_6_] (≥99.0%, Sigma-Aldrich) prepared with ultrapure water (18.2 MΩ cm, Milli-Q), and was deaerated by bubbling high-purity N_2_ (≥99.999%) for at least 30 min prior to and throughout the measurements.

A three-electrode configuration was used, comprising a glassy carbon disk–platinum ring RRDE (disk diameter 4.0 mm; ring inner/outer diameter 5.0/7.0 mm; Pine Research) as the working electrode, a platinum wire as the counter electrode, and a saturated Hg/HgO electrode as the reference. Prior to each measurement, the glassy carbon disk was polished sequentially with 1.0, 0.3, and 0.05 μm alumina slurries (Buehler) to a mirror finish, followed by ultrasonication in ultrapure water.

Measurements were performed at five rotation rates (*ω* = 400, 800, 1200, 1600 and 2000 rpm). The electrode was preconditioned with five cyclic voltammetric scans at each rotation rate to ensure a stable interface, and the limiting plateau currents for both the disk and ring were extracted for analysis.

The collection efficiency was calculated as:

26 $${\rm{N}}=-{{\rm{I}}}_{{\rm{ring}}}\,/\,{{\rm{I}}}_{{\rm{disk}}}$$

where *I*_ring_ is the limiting oxidation current at the ring and *I*_disk_ is the limiting reduction current at the disk.

### DEMS measurements

DEMS measurements were carried out in a sealed electrochemical cell using 1 M Na_2_SO_4_ as the electrolyte under an O_2_ atmosphere. Before the measurement, the electrolyte was purged with O_2_ to establish an O_2_-saturated environment, and a continuous O_2_ flow was maintained during the test. A microporous membrane was placed between the electrochemical compartment and the mass-spectrometric inlet to separate the liquid electrolyte from the gas-analysis line. The membrane was supplied by Linglu, with a nominal pore size of ≤20 nm and a thickness of 40 μm. According to the supplier specification, the pore-size distribution was mainly below 20 nm. The pore-size distribution was not independently characterized in this work; therefore, the reported pore size and pore-size distribution refer to the nominal values provided by the manufacturer.

During DEMS testing, the electrochemical measurements and mass-spectrometric signal acquisition were performed simultaneously. The selected mass-to-charge signals were continuously recorded during potential-controlled electrolysis to monitor the evolution and consumption of gas-phase. The raw ion-current signals were corrected by subtracting the background signal obtained under open-circuit conditions or from the blank electrolyte. Unless otherwise stated, the DEMS signals are presented as relative ion-current intensities. When quantitative amounts were calculated, calibration was performed using standard gases or species with known concentrations. Photographs of the DEMS setup and the electrochemical cell are provided in Supplementary Fig. 92.

## Supplementary information

Supplementary Information

Description of Additional Supplementary Files

Supplementary Data 1

Supplementary Data 2

Transparent Peer Review file

## Source data

Source Data

## Data Availability

The datasets analyzed and generated during the current study are included in the paper. Source data are provided with this paper.
