# Supplementary material for: Single-atom-engineered perovskite enables near-theoretical-rate hydroxyl radical electrogeneration
Source: Nat Commun. 2026 Jul 24;17:9026. doi: 10.1038/s41467-026-75926-5 (PMC13507266; doi:10.1038/s41467-026-75926-5)
Supplement: Supplementary file 1 [file 41467_2026_75926_MOESM1_ESM.pdf]

Supplementary Information

Single-atom-engineered perovskite enables near-theoretical-rate hydroxyl radical electrogeneration

Yaobin Wang[1], Chaoyue Xie[1], Ruiqing Zhao[1], Zhiyuan Su[1], Hongmei Li[2], Hang Zhang[2,3,4], Bo Li[5], Changhui Zhou[1], Yongyang Chen[1], Zeyu Du[1], Jinhua Li[1], Yunfei Bu[6], Jing Bai[1,*], Baoxue Zhou[1,*], Emiliano Cortés[4,*], Min Liu[2,3,*]

[1] State Key Laboratory of Green Papermaking and Resource Recycling, School of Environmental Science and Engineering, Shanghai Jiao Tong University, Shanghai 200240, China

[2] Hunan Joint International Research Center for Carbon Dioxide Resource Utilization, School of Physics, Central South University, Changsha 410083, China

[3] School of Metallurgy and Environment, Central South University, Changsha, 410083, China

[4] Nanoinstitut München, Fakultät für Physik, Ludwig-Maximilians-Universität München, München, Germany

[5] School of Science, STEM College, RMIT University, Melbourne, VIC 3000, Australia

[6] UNIST-NUlST Energy and Environment jointed Lab (UNNU), School of Environment science and technology, Nanjing University of Information Science and Technology, No.219, Ningliu Road, Nanjing, 210044 P.R. China

Table of Contents

Supplementary Figure 1. 5

Supplementary Figure 2. 6

Supplementary Figure 3. 6

Supplementary Figure 4. 7

Supplementary Figure 5. 8

Supplementary Figure 6. 9

Supplementary Figure 7. 10

Supplementary Figure 8. 11

Supplementary Figure 9. 12

Supplementary Figure 10. 13

Supplementary Figure 11. 14

Supplementary Figure 12. 15

Supplementary Figure 13. 16

Supplementary Figure 14. 17

Supplementary Figure 15. 18

Supplementary Figure 16. 19

Supplementary Figure 17. 20

Supplementary Figure 18. 21

Supplementary Figure 19. 22

Supplementary Figure 20. 23

Supplementary Figure 21. 23

Supplementary Figure 22. 24

Supplementary Figure 23. 24

Supplementary Figure 24. 25

Supplementary Figure 25. 26

Supplementary Figure 26. 27

Supplementary Figure 27. 28

Supplementary Figure 28. 29

Supplementary Figure 29. 30
Supplementary Figure 30. 31
Supplementary Figure 31. 32
Supplementary Figure 32. 33
Supplementary Figure 33. 34
Supplementary Figure 34. 34
Supplementary Figure 35. 35
Supplementary Figure 36. 36
Supplementary Figure 37. 37
Supplementary Figure 38. 38
Supplementary Figure 39. 39
Supplementary Figure 40. 40
Supplementary Figure 41. 41
Supplementary Figure 42. 42
Supplementary Figure 43. 43
Supplementary Figure 44. 44
Supplementary Figure 45. 45
Supplementary Figure 46. 46
Supplementary Figure 47. 47
Supplementary Figure 48. 48
Supplementary Figure 49. 49
Supplementary Figure 50. 50
Supplementary Figure 51. 51
Supplementary Figure 52. 52
Supplementary Figure 53. 53
Supplementary Figure 54. 54
Supplementary Figure 55. 55
Supplementary Figure 56. 56
Supplementary Figure 57. 57
Supplementary Figure 58. 58

Supplementary Figure 59. 59
Supplementary Figure 60. 60
Supplementary Figure 61. 61
Supplementary Figure 62. 62
Supplementary Figure 63. 63
Supplementary Figure 64. 64
Supplementary Figure 65. 65
Supplementary Figure 66. 66
Supplementary Figure 67. 67
Supplementary Figure 68. 68
Supplementary Figure 69. 69
Supplementary Figure 70. 70
Supplementary Figure 71. 71
Supplementary Figure 72. 71
Supplementary Figure 73. 72
Supplementary Figure 74. 73
Supplementary Figure 75. 74
Supplementary Figure 76. 75
Supplementary Figure 77. 76
Supplementary Figure 78. 77
Supplementary Figure 79. 78
Supplementary Figure 80. 79
Supplementary Figure 81. 80
Supplementary Figure 82. 81
Supplementary Figure 83. 82
Supplementary Figure 84. 83
Supplementary Figure 85. 84
Supplementary Figure 86. 85
Supplementary Figure 87. 86
Supplementary Figure 88. 87

Supplementary Figure 89. 88
Supplementary Figure 90. 89
Supplementary Figure 91. 90
Supplementary Figure 92. 91
Supplementary Table 1. 92
Supplementary Table 2. 93
Supplementary Table 3. 94
Supplementary Table 4. 95
Supplementary Table 5. 96
Supplementary Table 6. 97
Supplementary Table 7. 98
References 99

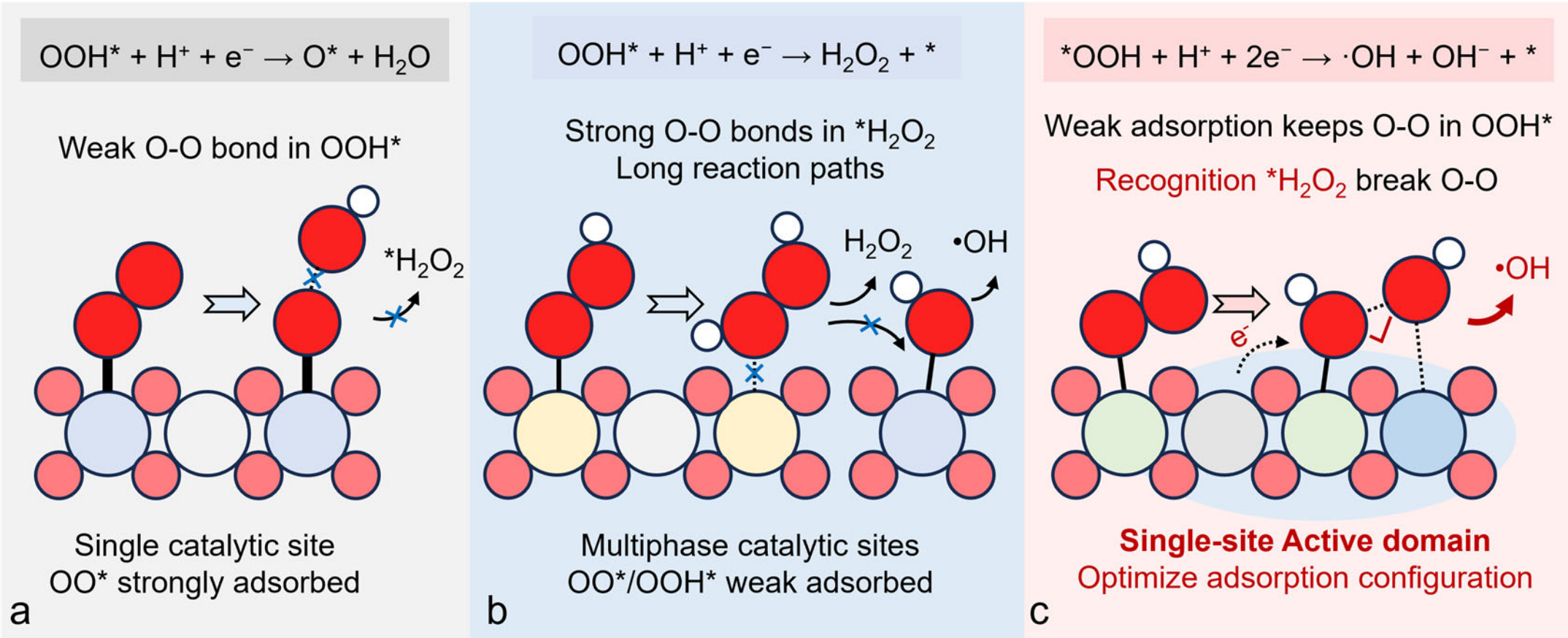

Supplementary Fig. 1 Intermediate recognition strategy promotes •OH generation. **a** Schematic diagram of the O-O bond breaking in *OOH caused by strong adsorption of O_2 at a single catalytic site. This process follows the four-electron oxygen reduction pathway. **b** Schematic diagram of the O-O bond retention in *H_2O_2 caused by weak adsorption of heterogeneous catalytic sites. This process follows the two-electron oxygen reduction pathway. **c** Active domain-single site recognizes oxygen reduction intermediates to maintain/break the O-O bond. This process follows the three-electron oxygen reduction pathway. Colors represent: transition metal elements (light blue/light yellow), lanthanide metal elements (grey), Zn (green), Fe (dark gray), Mo (blue), O (red), H (white).

Moreover, the similar binding characteristics of *O_2 and *OOH often lead to a proportional scaling relationship, where strong *OOH binding enhances O_2 activation but suppresses H_2O_2 evolution (**Supplementary Fig. 1**). Conversely, weak *OOH adsorption may allow O-O retention in *H_2O_2 but necessitates desorption and transfer to external sites for activation into •OH.

To overcome these challenges, we propose a single-site active domain strategy, wherein co-catalytic elements embedded within a perovskite oxide lattice modulate the adsorption configurations and reactivities of key intermediates (*OOH and *H_2O_2) at a unified site.

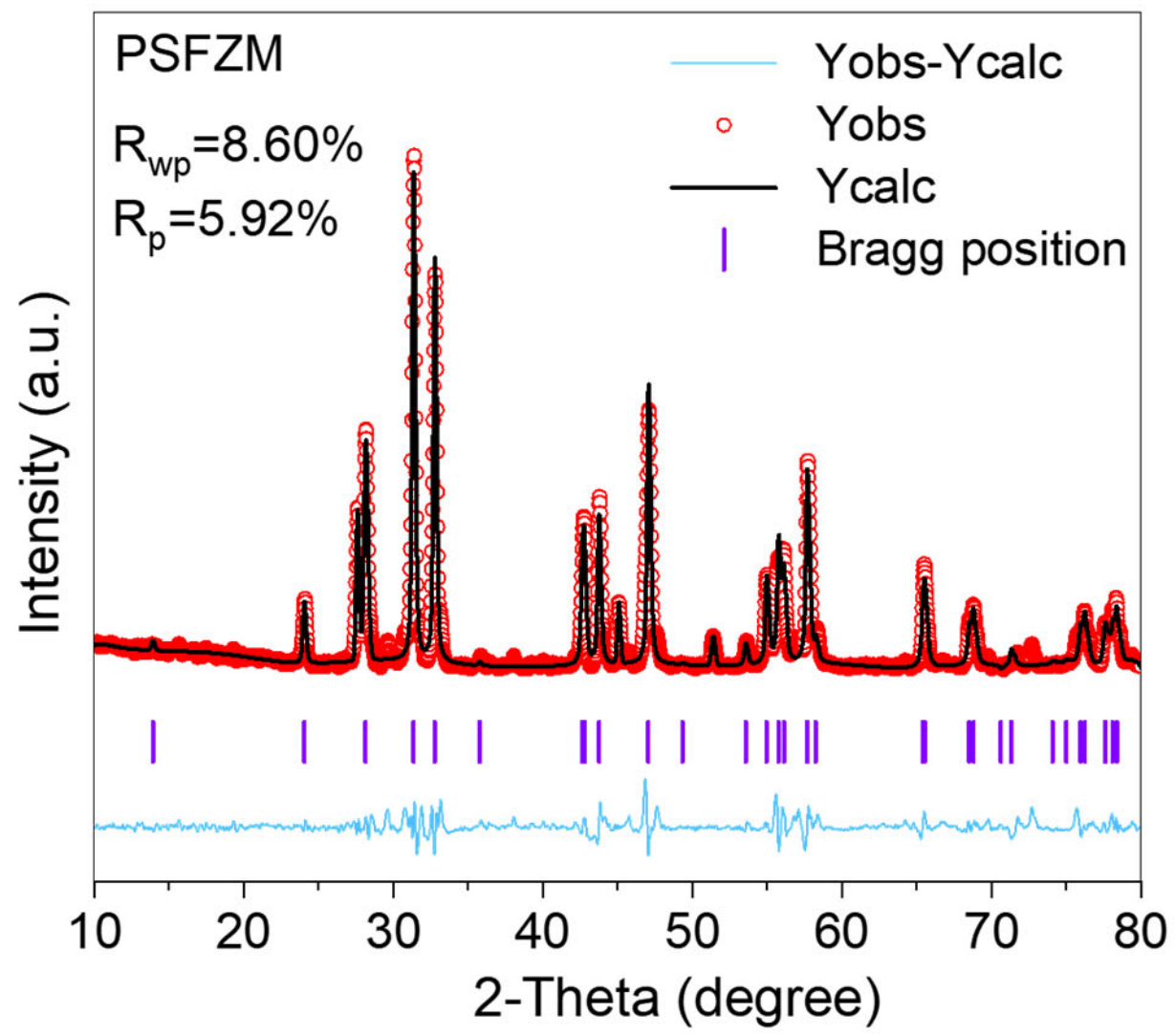

Supplementary Fig. 2. XRD Rietveld refinement of PSFZM. Source data for Supplementary Fig. 2 are provided as a Source Data file.

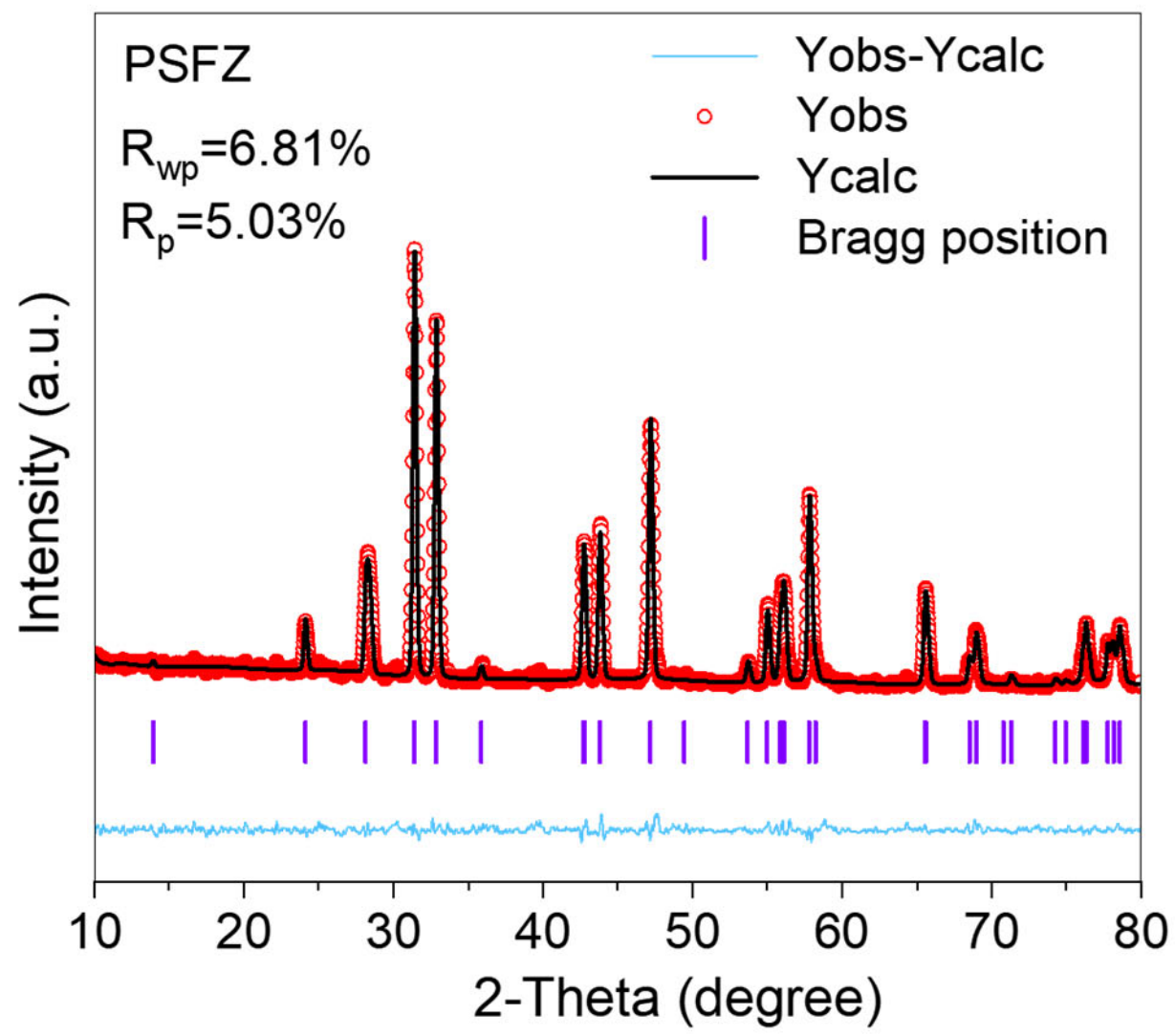

Supplementary Fig. 3. XRD Rietveld refinement of PSFZ. Source data for Supplementary Fig. 3 are provided as a Source Data file.

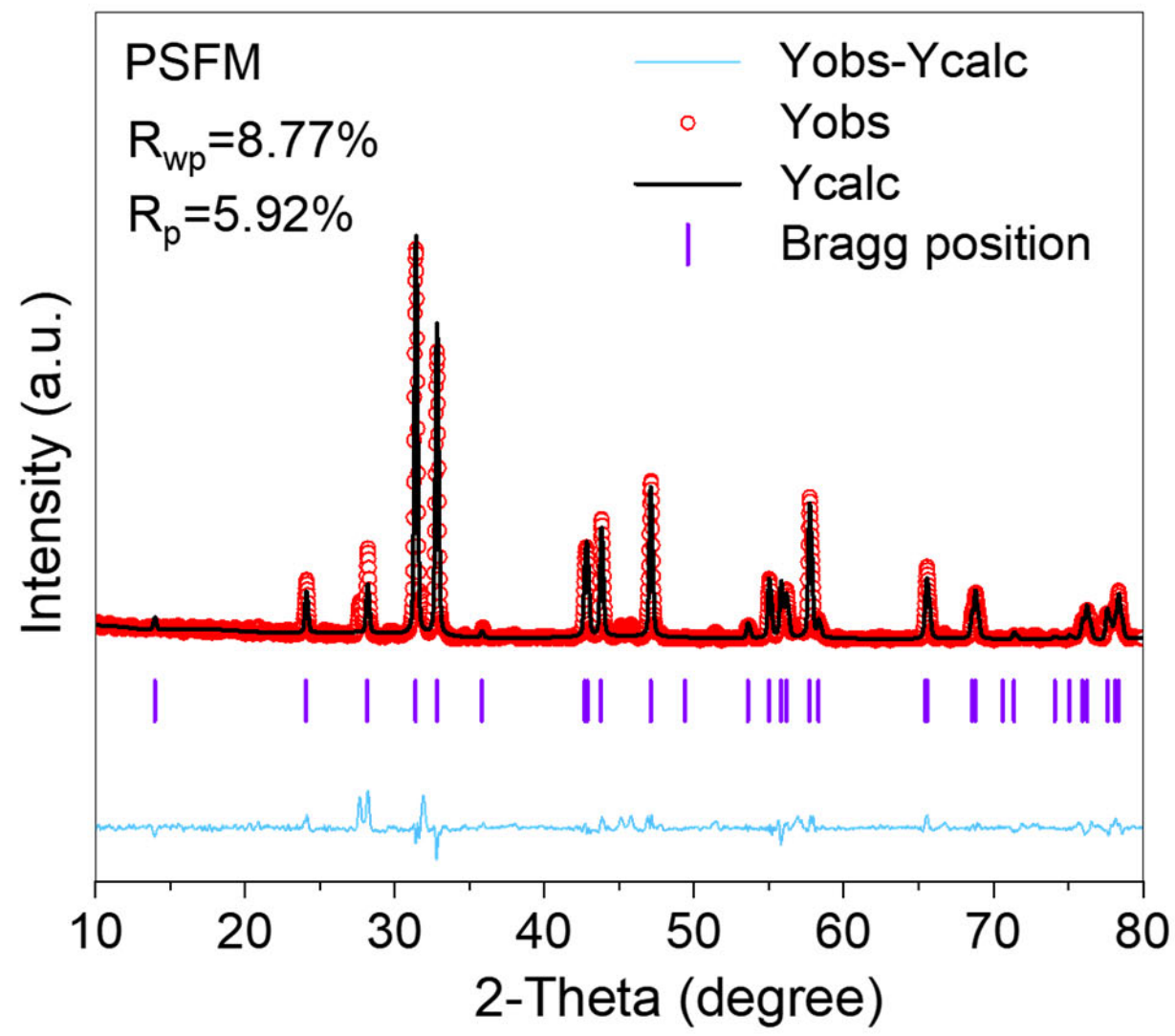

Supplementary Fig. 4. XRD Rietveld refinement of PSFM. Source data for Supplementary Fig. 4 are provided as a Source Data file.

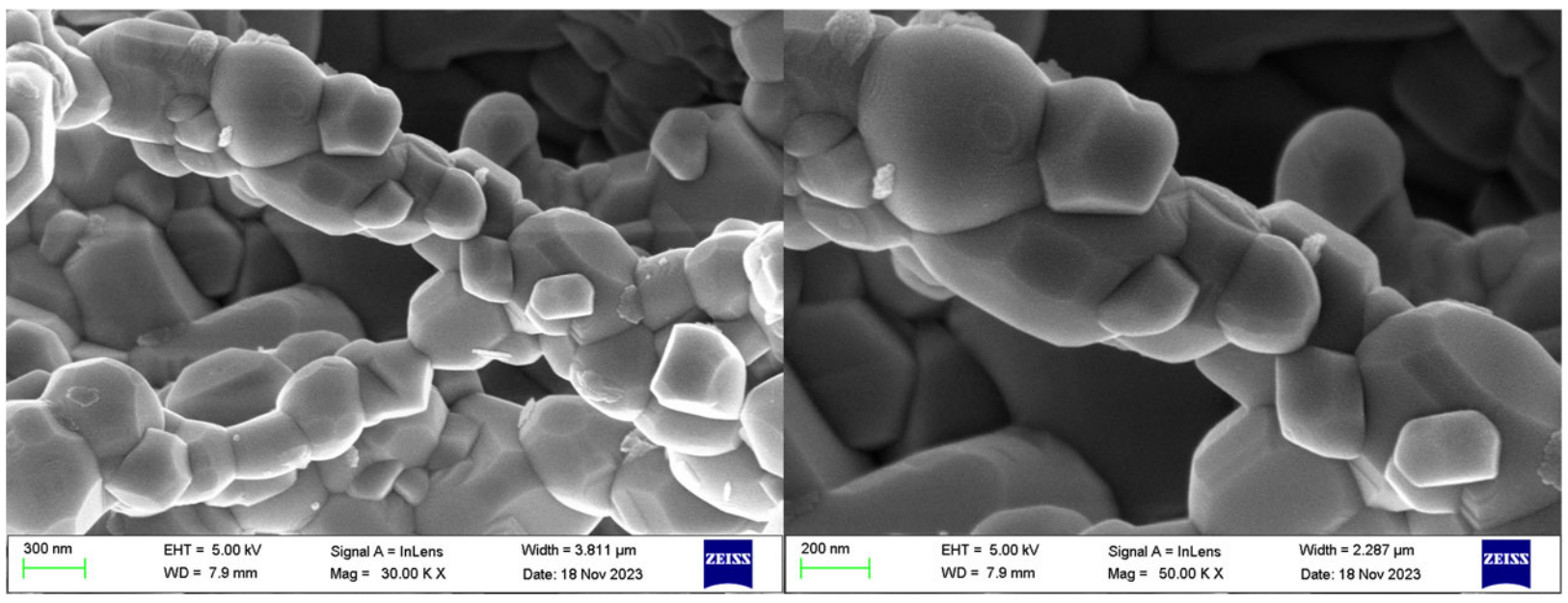

Supplementary Fig. 5. SEM image of PSFZM.

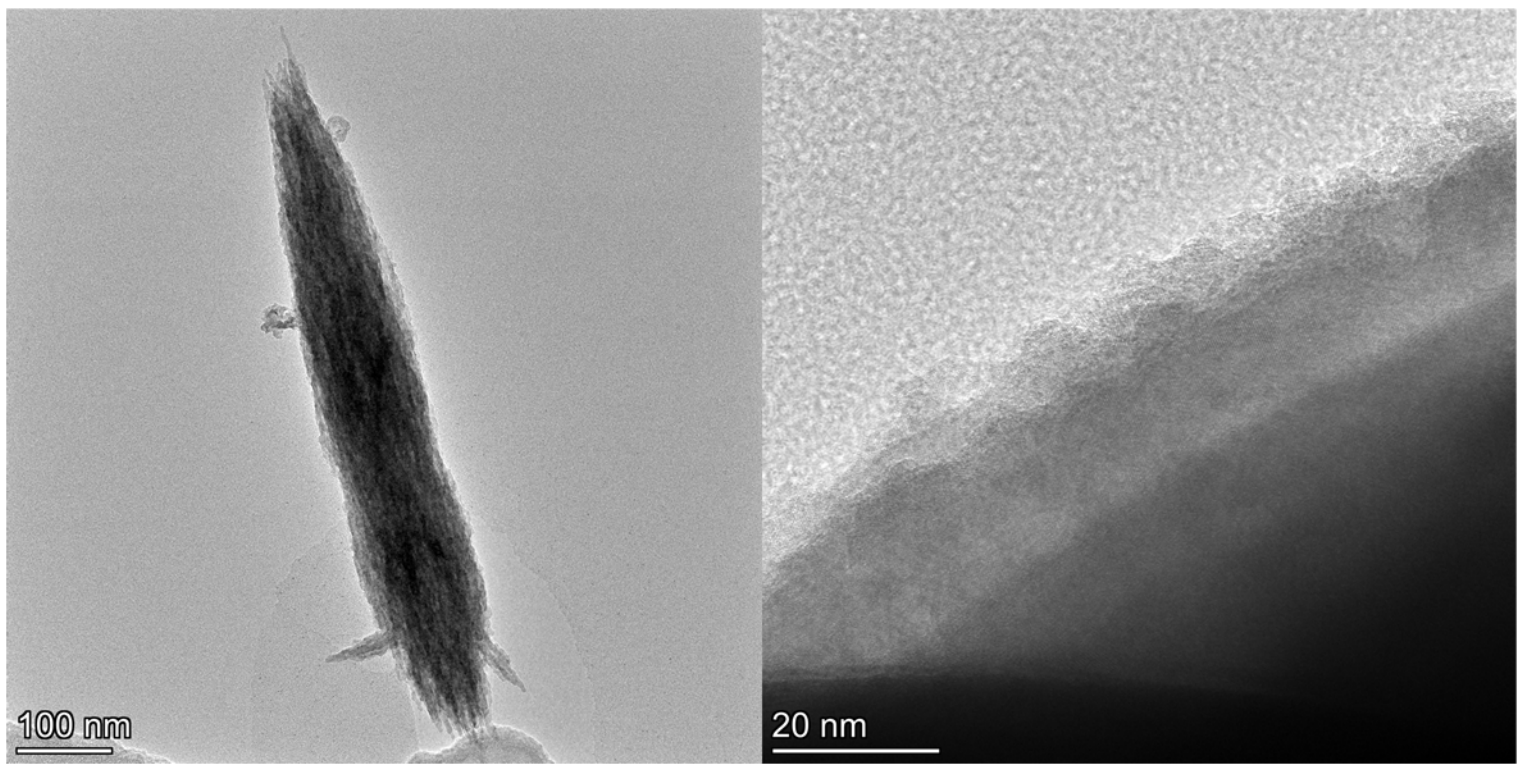

Supplementary Fig. 6 TEM image of PSFZM.

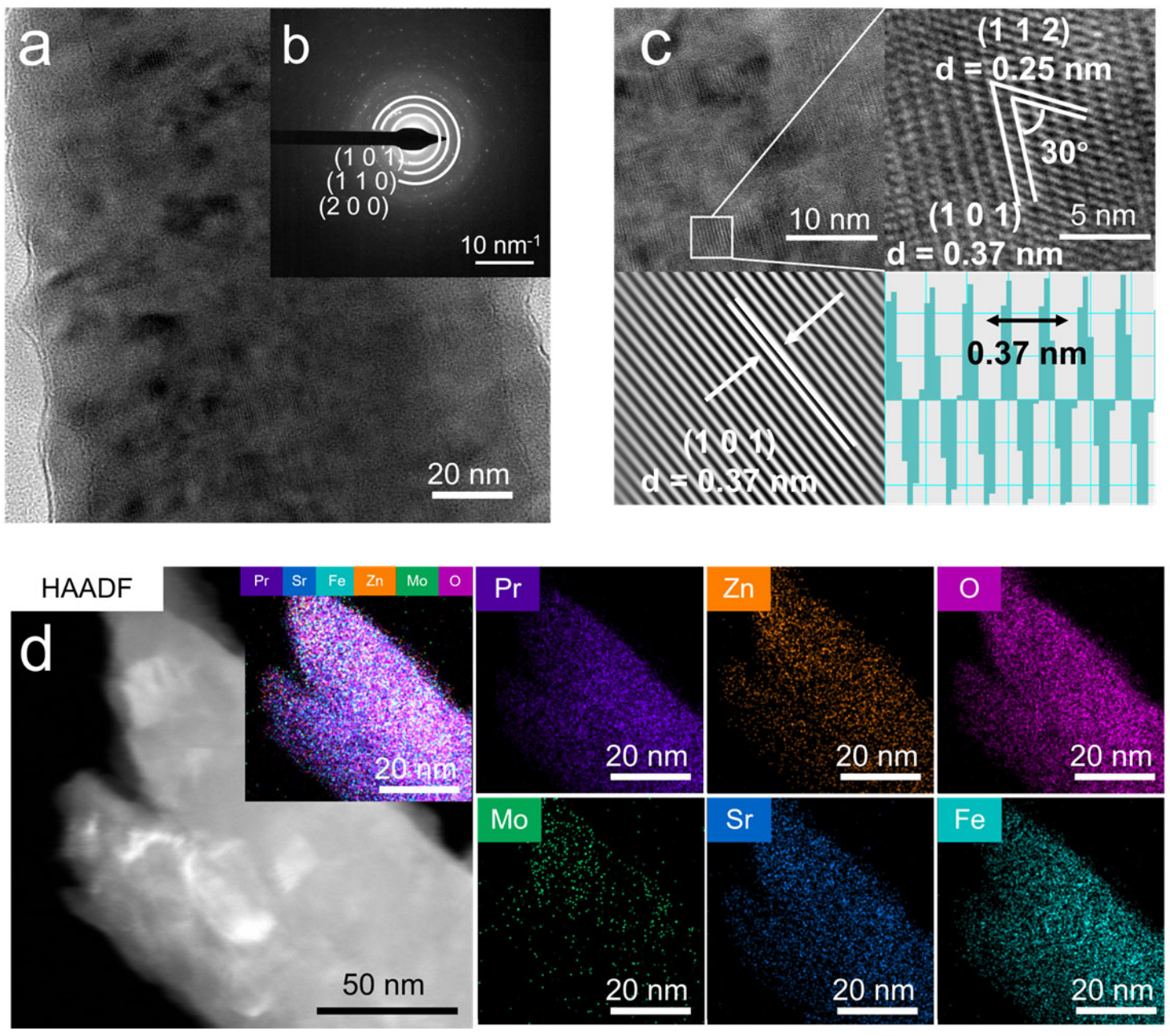

Supplementary Fig. 7. a TEM image and **b** the corresponding selected area electron diffraction (SAED) pattern of PSFZM. **c** HR-TEM image of PSFZM. The inset shows the FFT image and lattice spacing distribution. **d** HAADF-TEM image and the corresponding elemental maps for PSFZM.

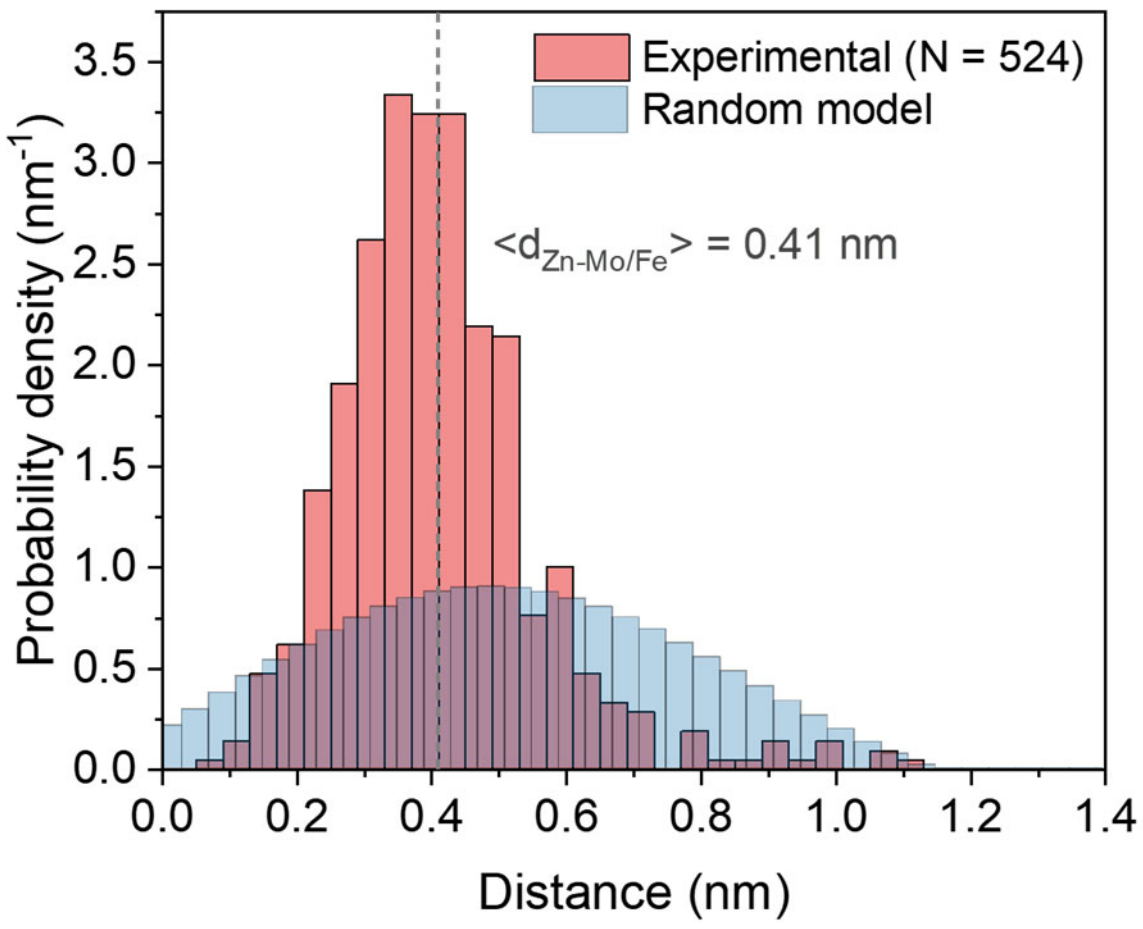

Supplementary Fig. 8 Statistical distribution of Zn–Mo/Fe neighbouring distances in PSFZM. Source data for Supplementary Fig. 8 are provided as a Source Data file.

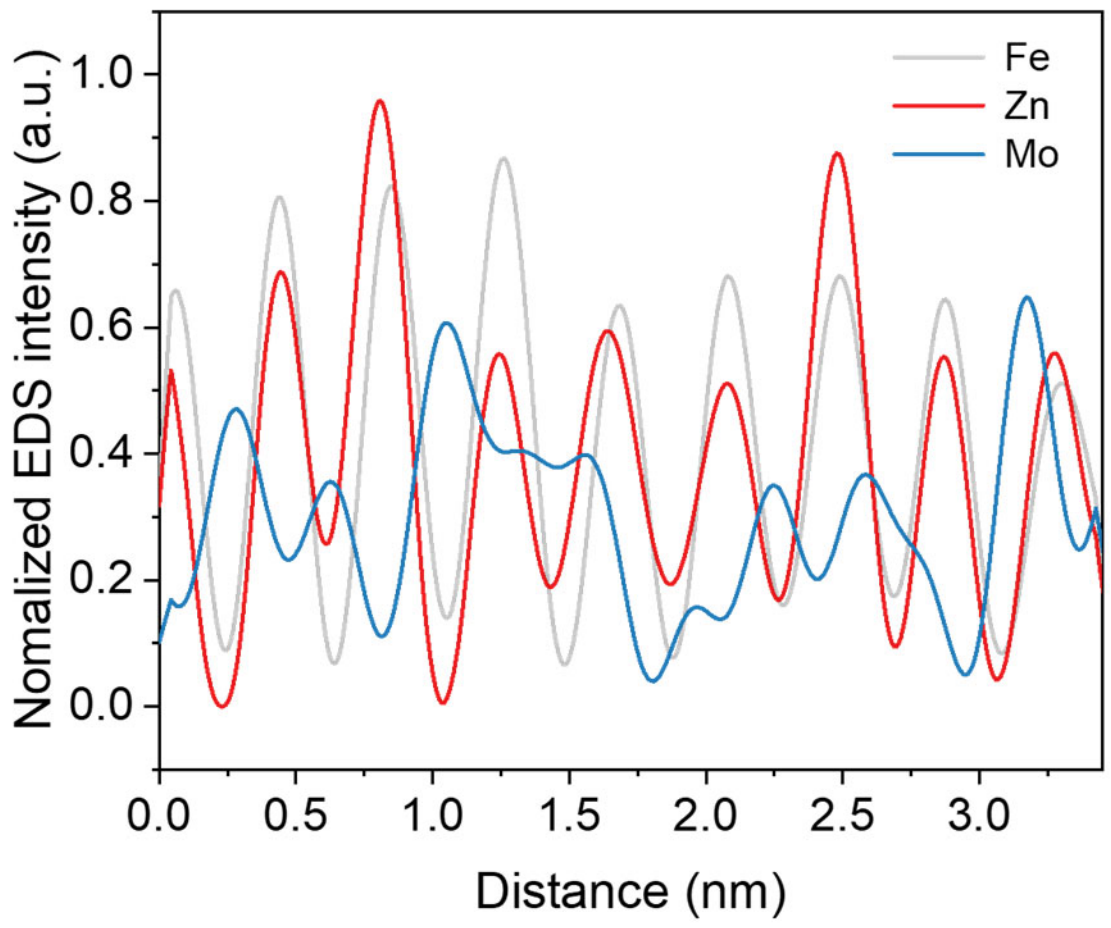

Supplementary Fig. 9 STEM-EDS line-scan analysis of Fe, Zn and Mo distributions in PSFZM. Source data for Supplementary Fig. 9 are provided as a Source Data file.

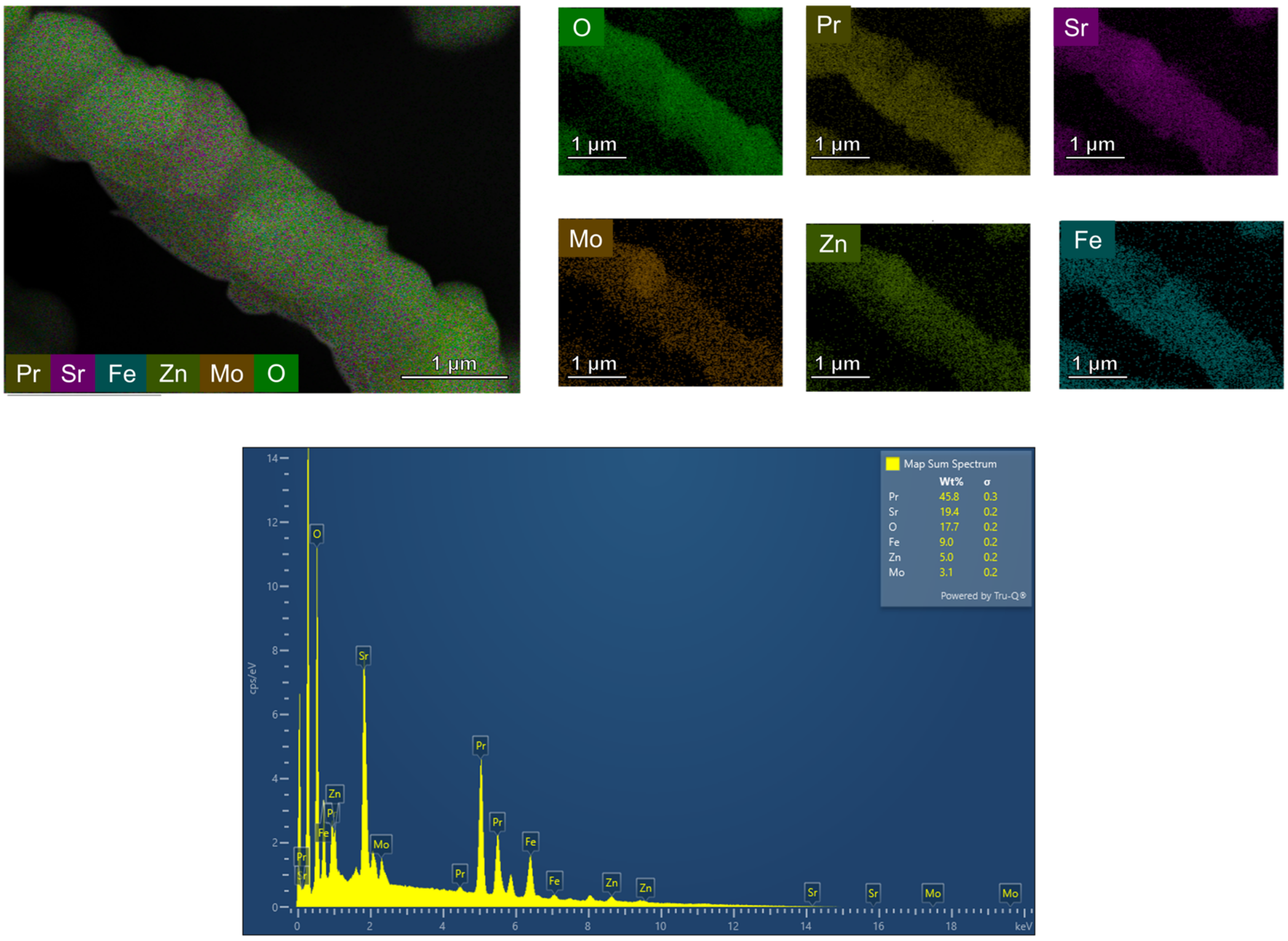

Supplementary Fig. 10 SEM elemental mapping and map sum spectrum of PSFZM.

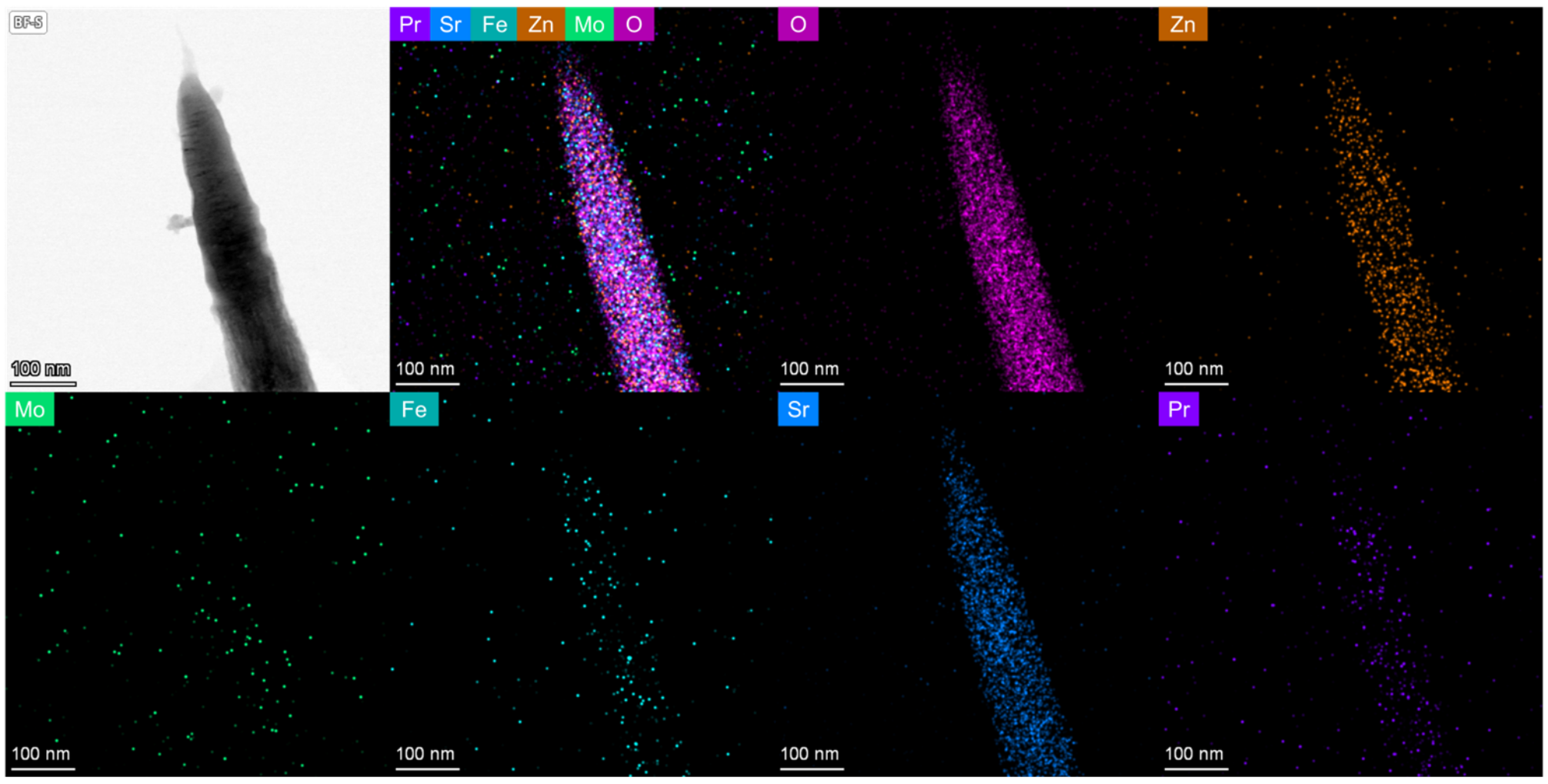

Supplementary Fig. 11 HAADF-TEM images and elemental mapping of PSFZM.

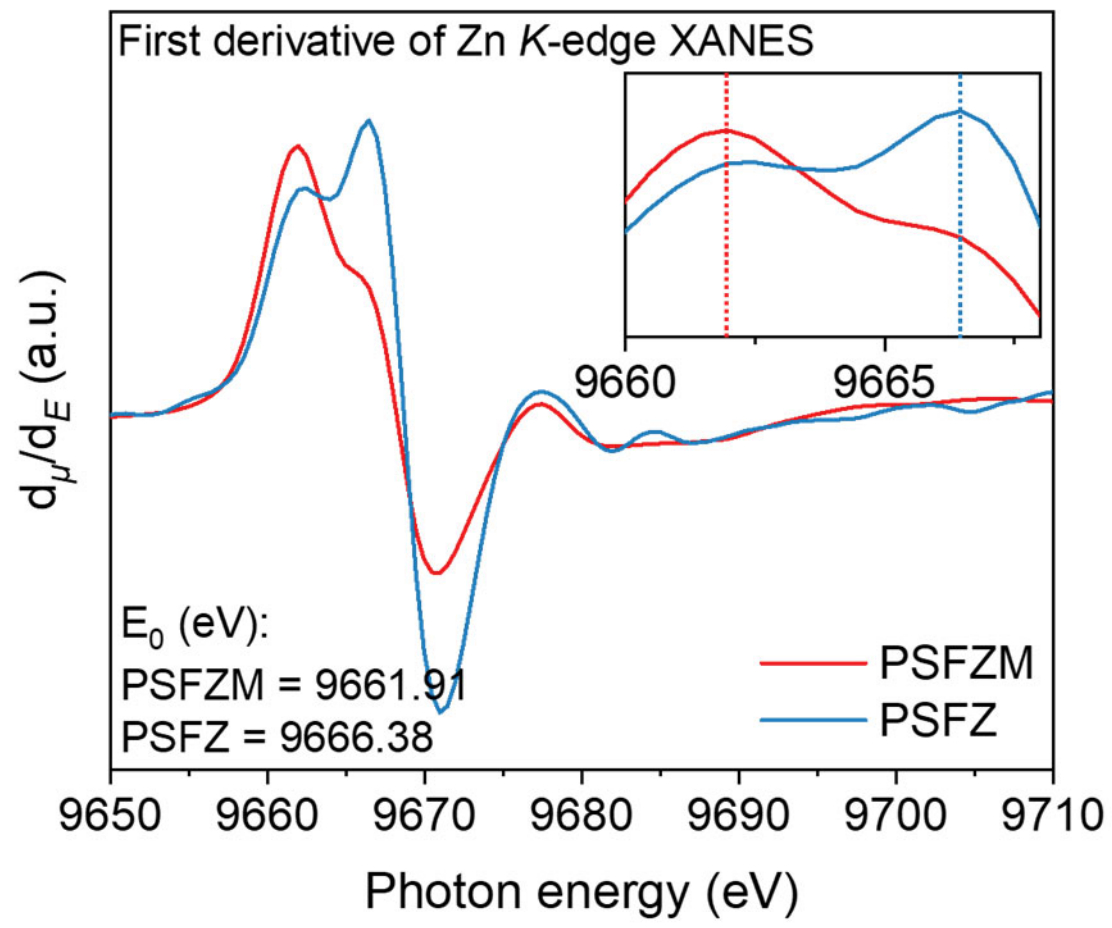

Supplementary Fig. 12 First-derivative Zn *K*-edge XANES spectra of PSFZM and PSFZ. Source data for Supplementary Fig. 12 are provided as a Source Data file.

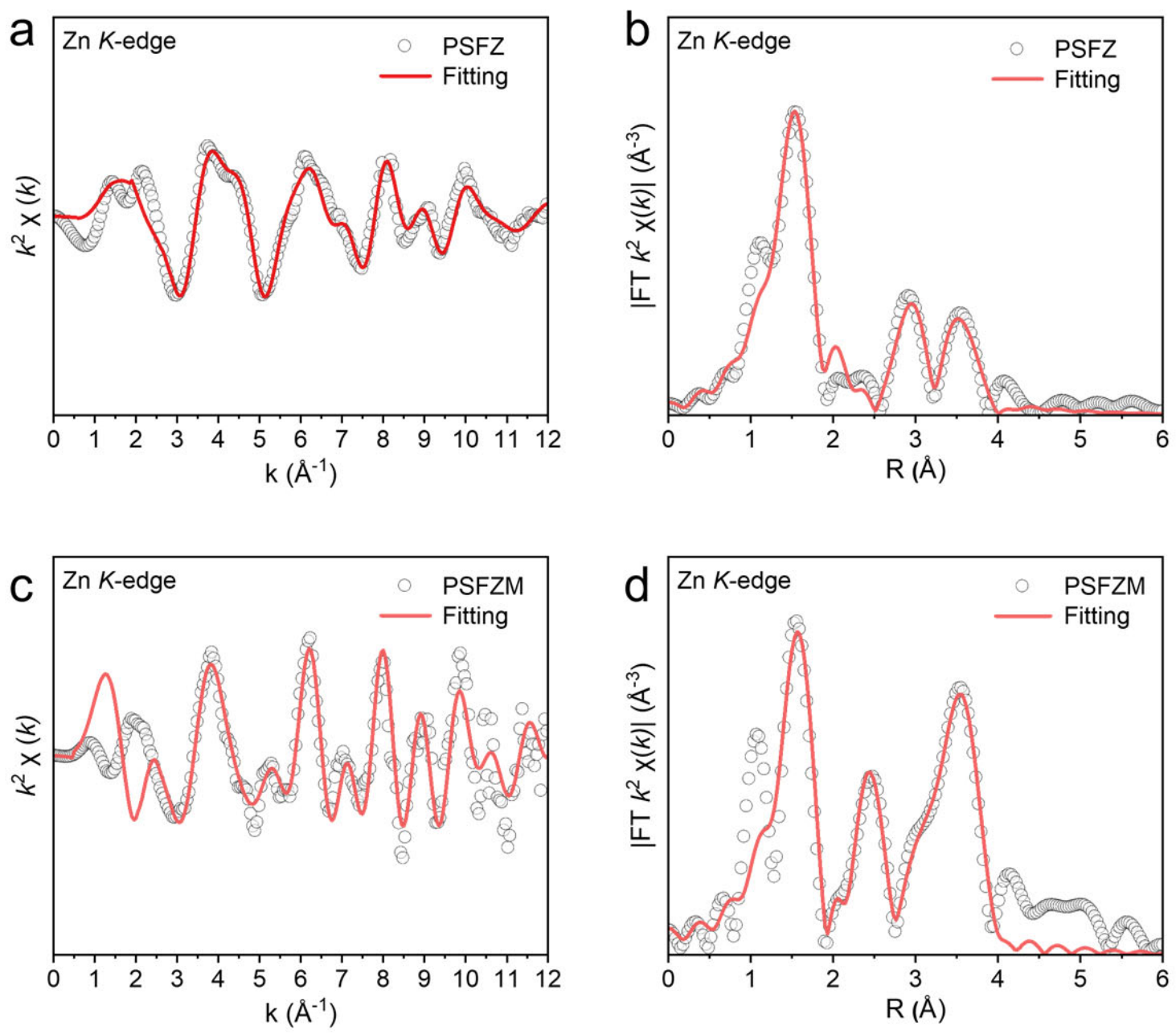

Supplementary Fig. 13 Fitting of the Zn *K*-edge EXAFS K spectra for **a** PSFZ and **c** PSFZM. Fitting of the Zn *K*-edge EXAFS R space fitting result for **b** PSFZ and **d** PSFZM. Source data for Supplementary Fig. 13 are provided as a Source Data file.

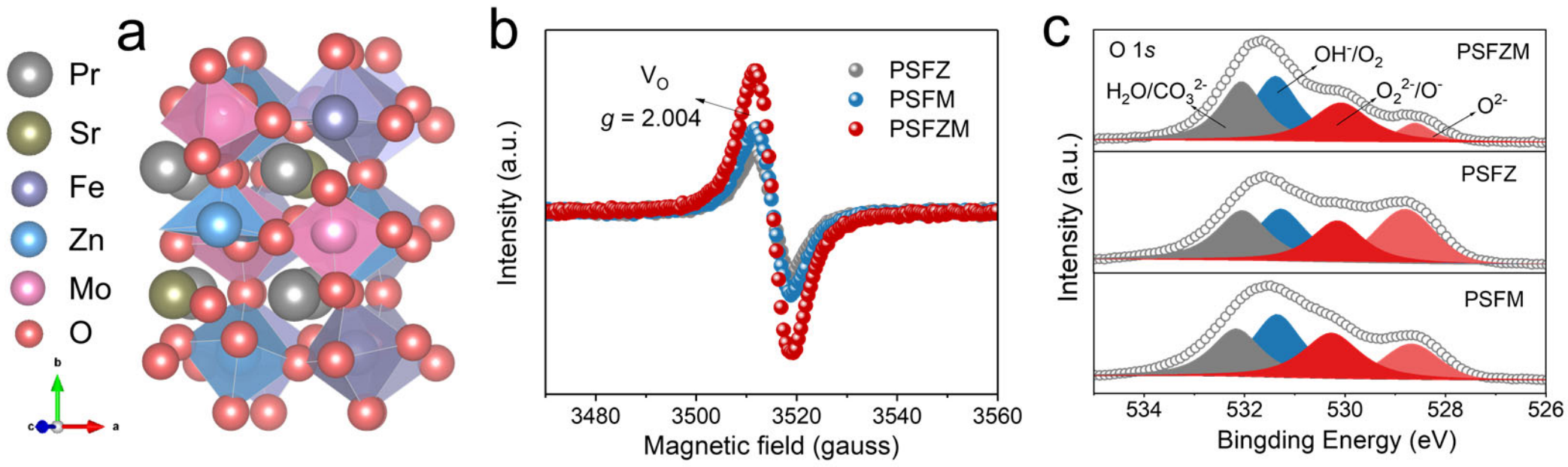

Supplementary Fig. 14 a Schematic presentation of PSFZM perovskite crystal structure. **b** The low-temperature solid EPR results of PSFZM, PSFZ and PSFM. **c** XPS depth profile analysis in O 1*s* region for PSFZM, PSFZ and PSFM. The shaded peaks denote the fitted O 1*s* components corresponding to lattice oxygen, oxygen-vacancy-related defective oxygen, and surface adsorbed oxygen/hydroxyl species. Source data for Supplementary Fig. 14 are provided as a Source Data file.

To investigate oxygen vacancies (O_v) concentrations, we employed electron spin resonance (ESR) spectroscopy (**Supplementary Figs. 14b**). The results reveal a stronger ESR signal for PSFZM compared to PSFZ and PSFM, confirming increased O_v formation due to Mo incorporation. Further X-ray photoelectron spectroscopy (XPS) provided insights into surface and bulk chemical states.

The O 1*s* high-resolution XPS spectra (**Supplementary Figs. 14c**) for PSFZM, PSFZ, and PSFM, revealed multiple components: lattice oxygen (O_L, ~528.6 eV), O_v (~530.2 eV), surface adsorbed oxygen species (O_{ads}, ~531.5 eV), and molecular water (O_w, ~532.4 eV). Quantitative deconvolution showed that PSFZM had the highest O_v/O_{total} ratio among all samples, indicating that high-valent Mo incorporation facilitates lattice distortion and O_v formation.

These O_v are crucial for catalytic activity. As previously reported, internal O_v enhance the peroxo-intermediate (*OOH) binding capacity at Zn sites while suppressing undesired decomposition pathways to *O and OH^-. This synergy is critical for facilitating efficient three-electron ORR to •OH.

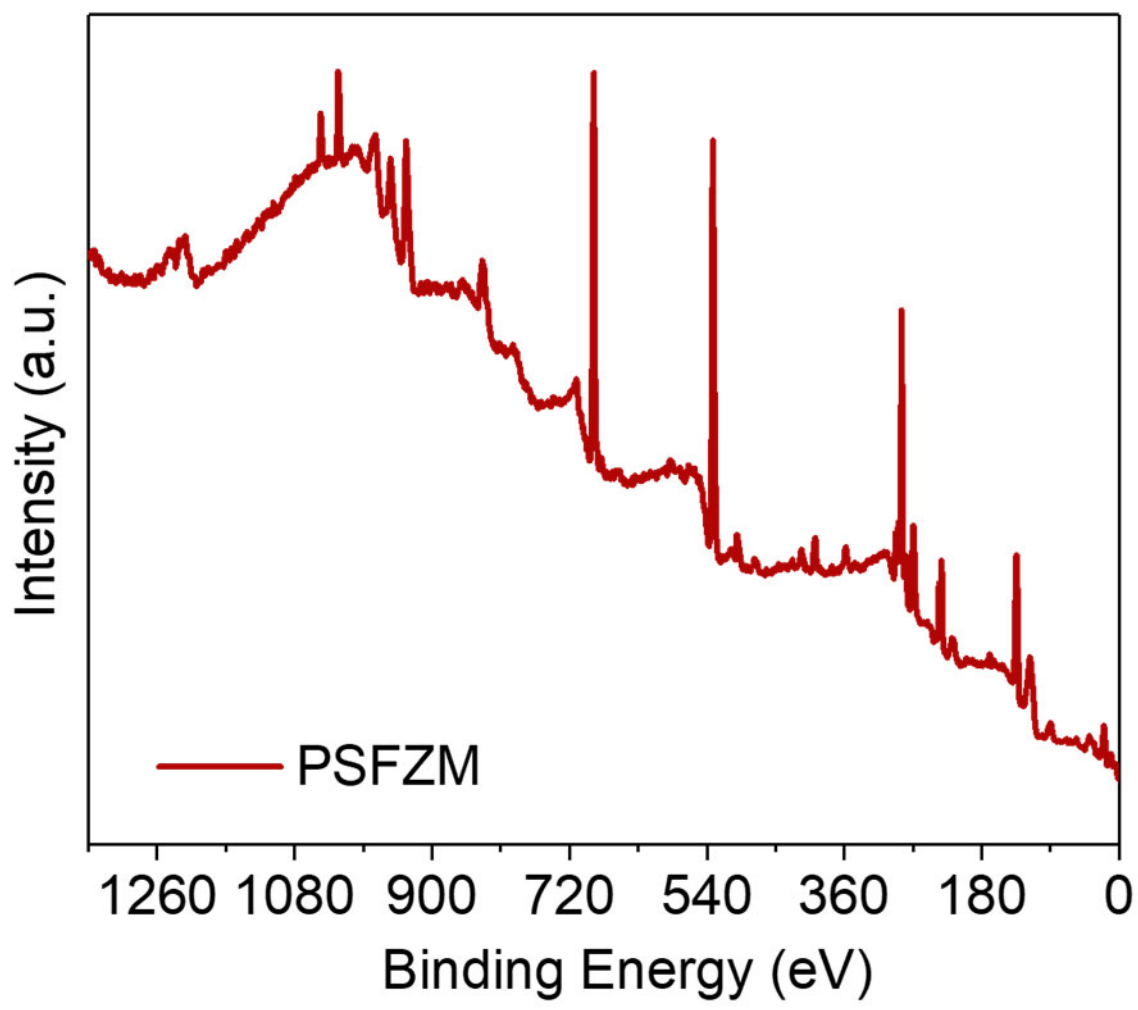

Supplementary Fig. 15 Surface XPS full survey scan of PSFZM oxide. Source data for Supplementary Fig. 15 are provided as a Source Data file.

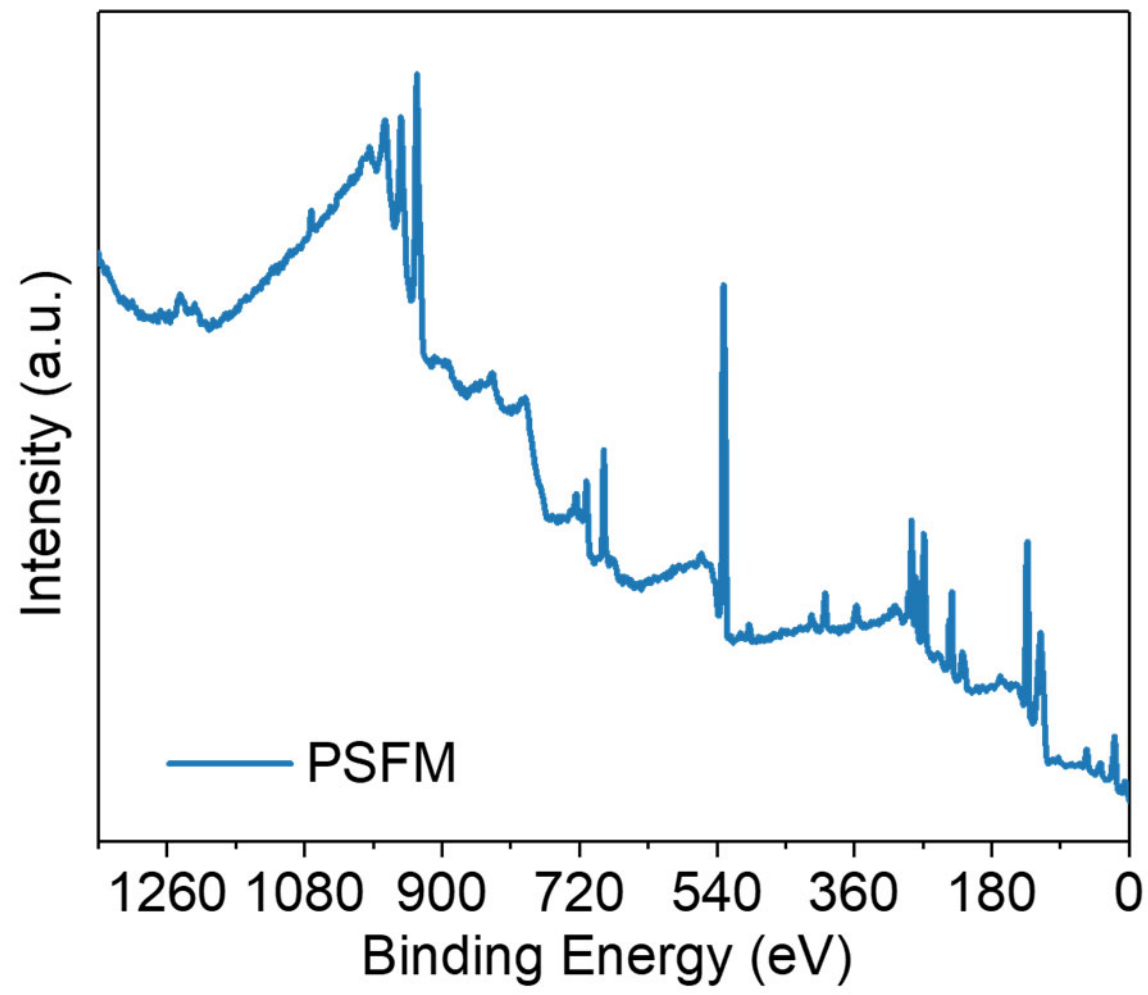

Supplementary Fig. 16 Surface XPS full survey scan of PSFM oxide. Source data for Supplementary Fig. 16 are provided as a Source Data file.

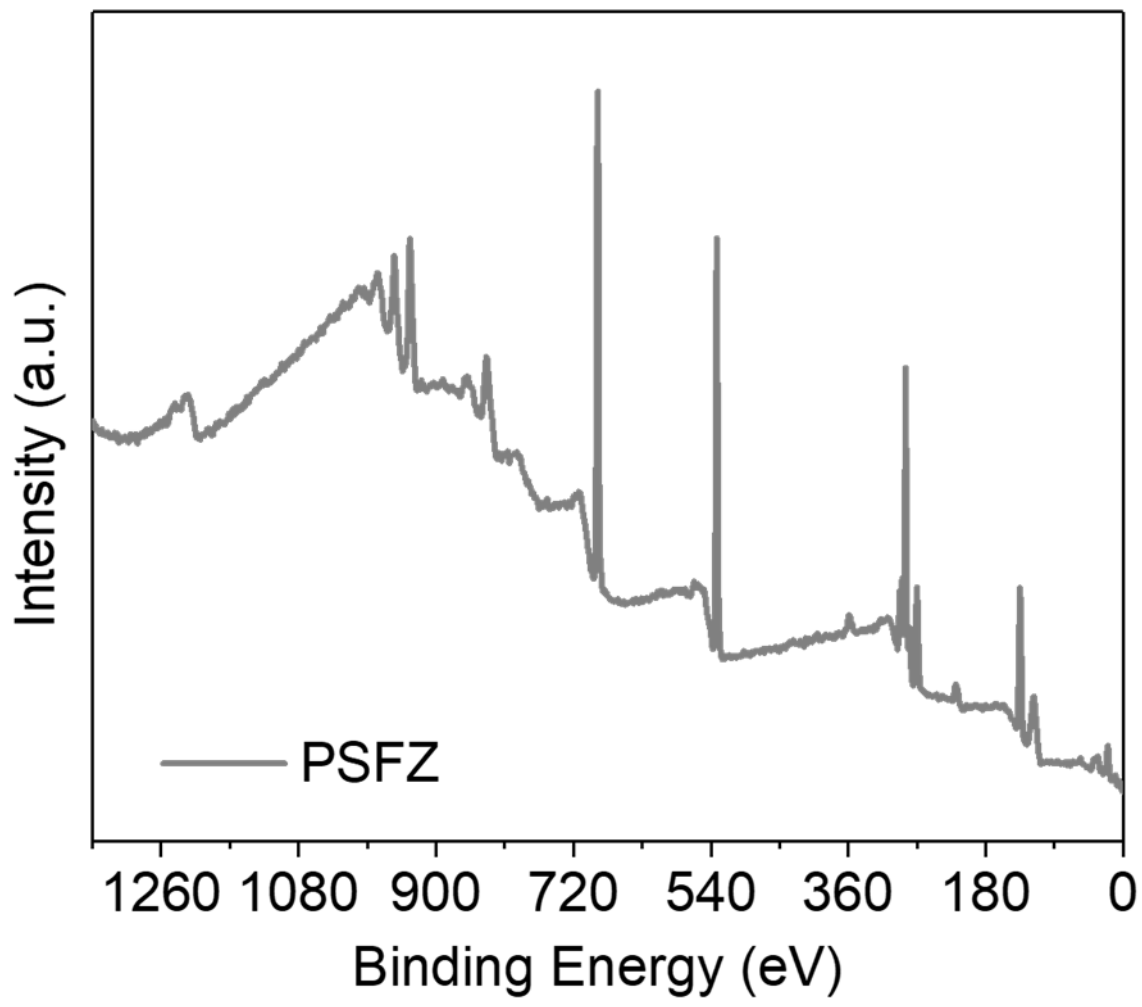

Supplementary Fig. 17 Surface XPS full survey scan of PSFZ oxide. Source data for Supplementary Fig. 17 are provided as a Source Data file.

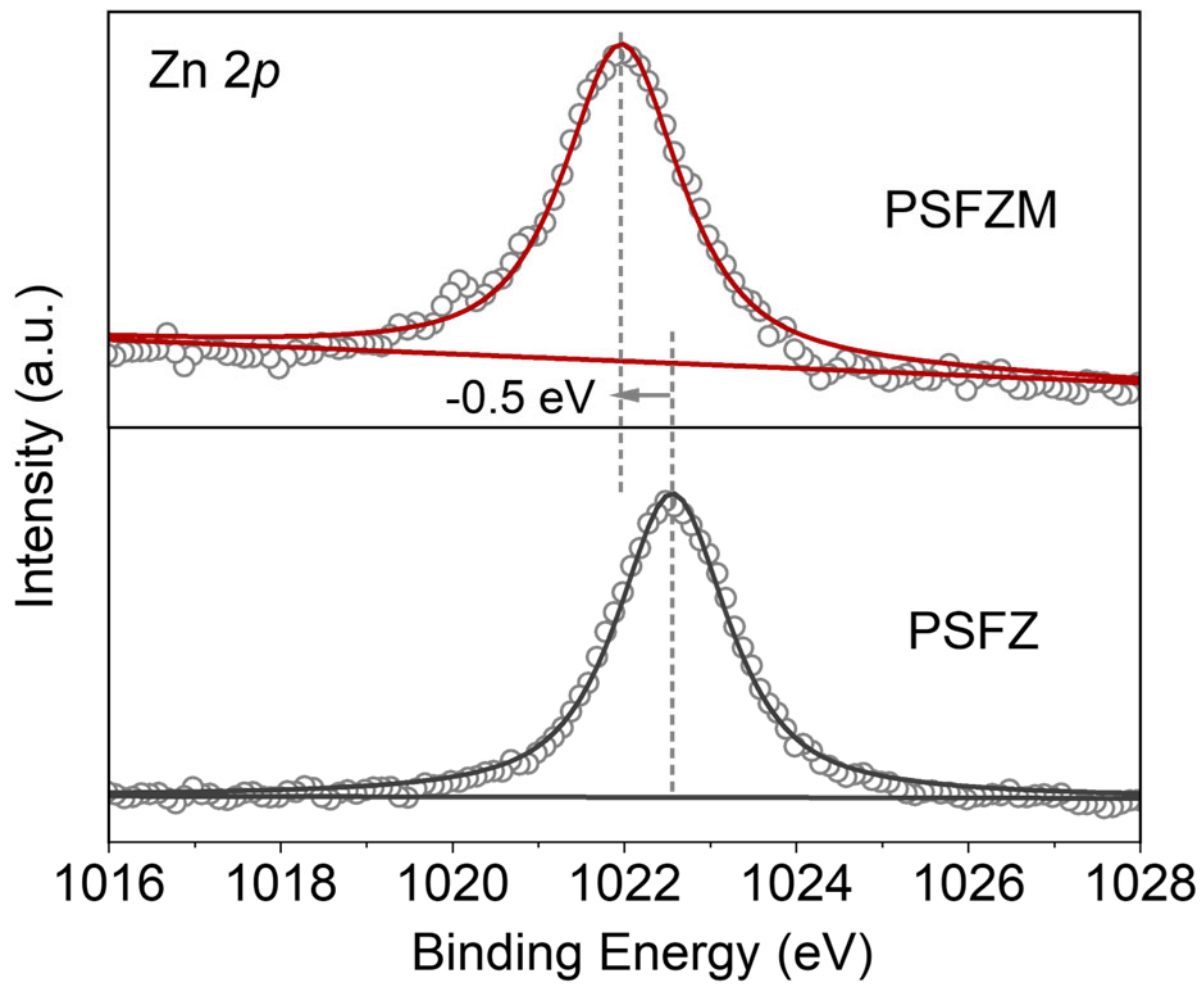

Supplementary Fig. 18 High-resolution surface XPS scan of Zn 2*p* of PSFZM and PSFZ oxide. Source data for Supplementary Fig. 18 are provided as a Source Data file.

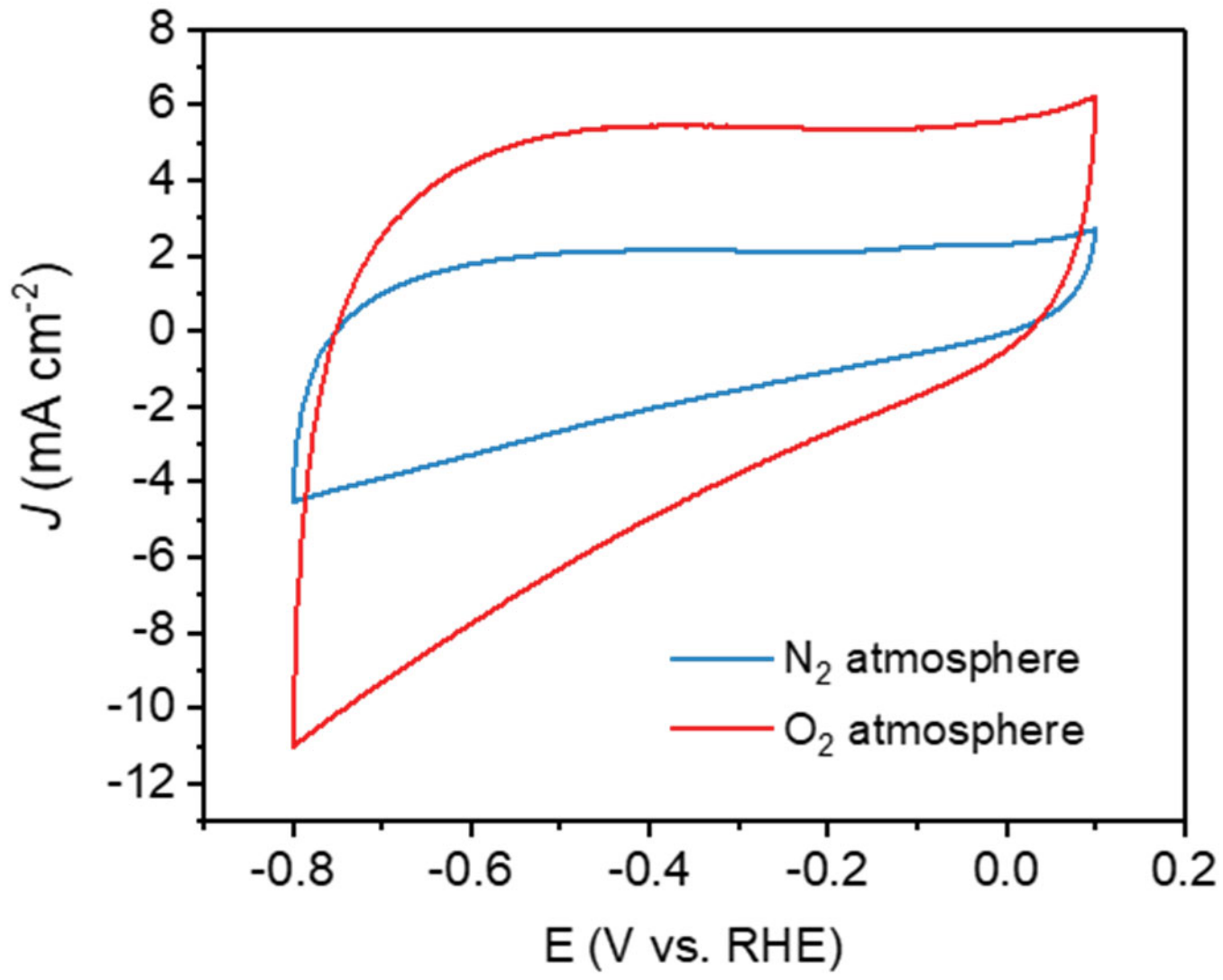

Supplementary Fig. 19 CV curves in the potential range of 0.1 V - -0.8 V (vs. RHE) under N_2 and O_2 atmospheres, respectively. Source data for Supplementary Fig. 19 are provided as a Source Data file.

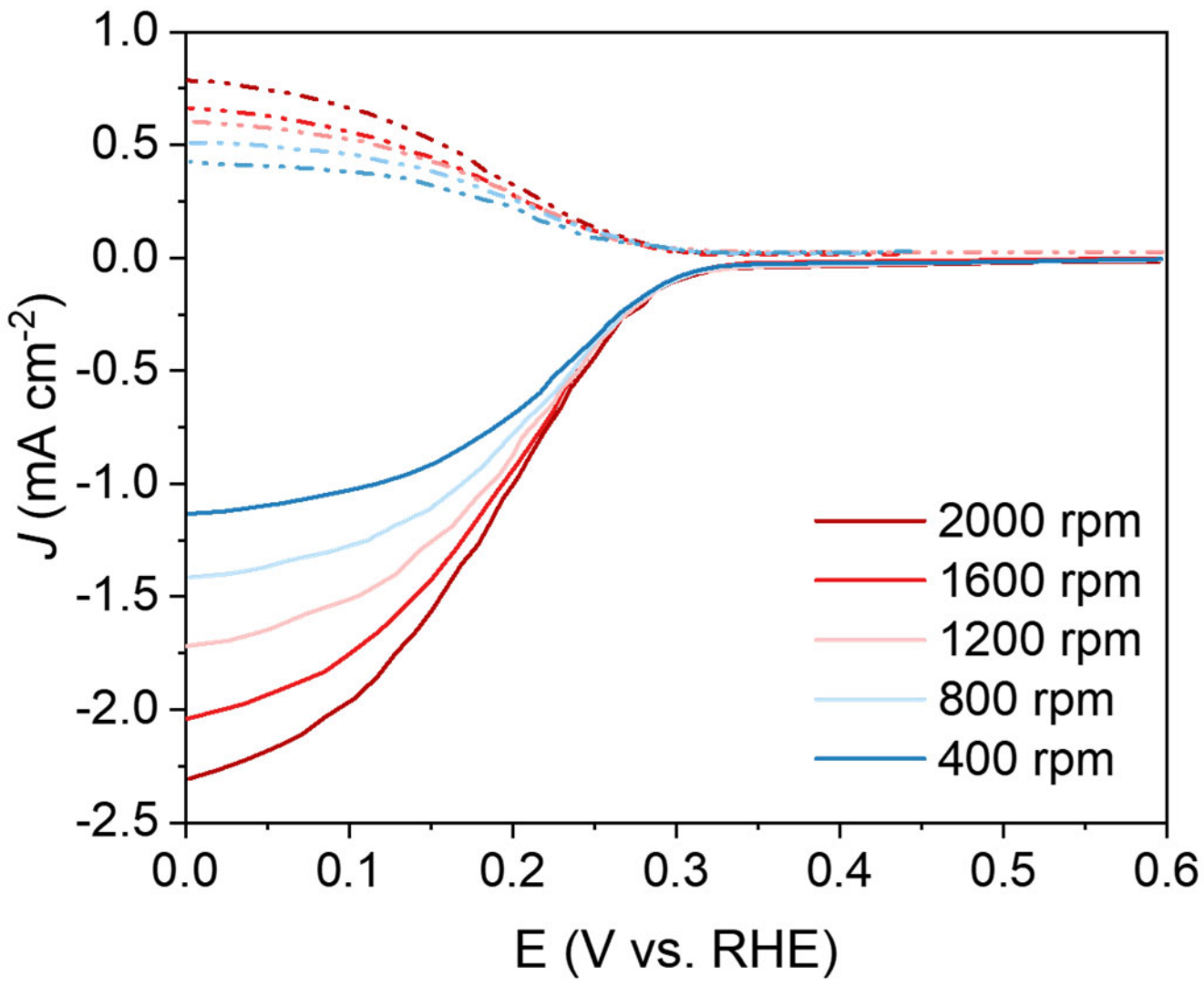

Supplementary Fig. 20 LSV curves in 10 mM $K_3[Fe(CN)_6]$ at different rotation speeds. Note: The solid lines are the disk current density corresponding to oxygen reduction and the dotted lines are the ring current density corresponding to H_2O_2 oxidation. Source data for Supplementary Fig. 20 are provided as a Source Data file.

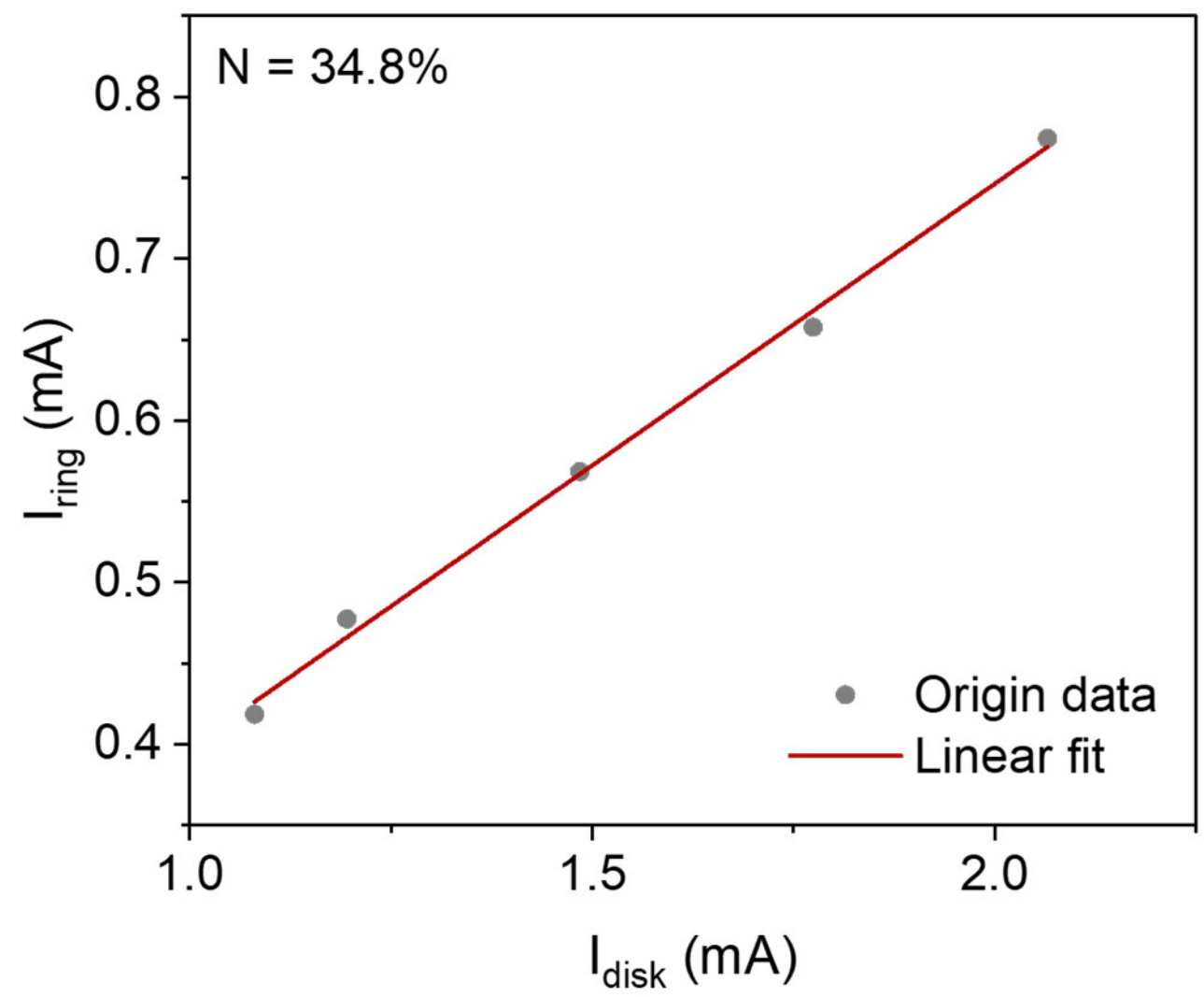

Supplementary Fig. 21 The collection efficiency (N) of RRDE, which was determined to be 34.8%. Source data for Supplementary Fig. 21 are provided as a Source Data file.

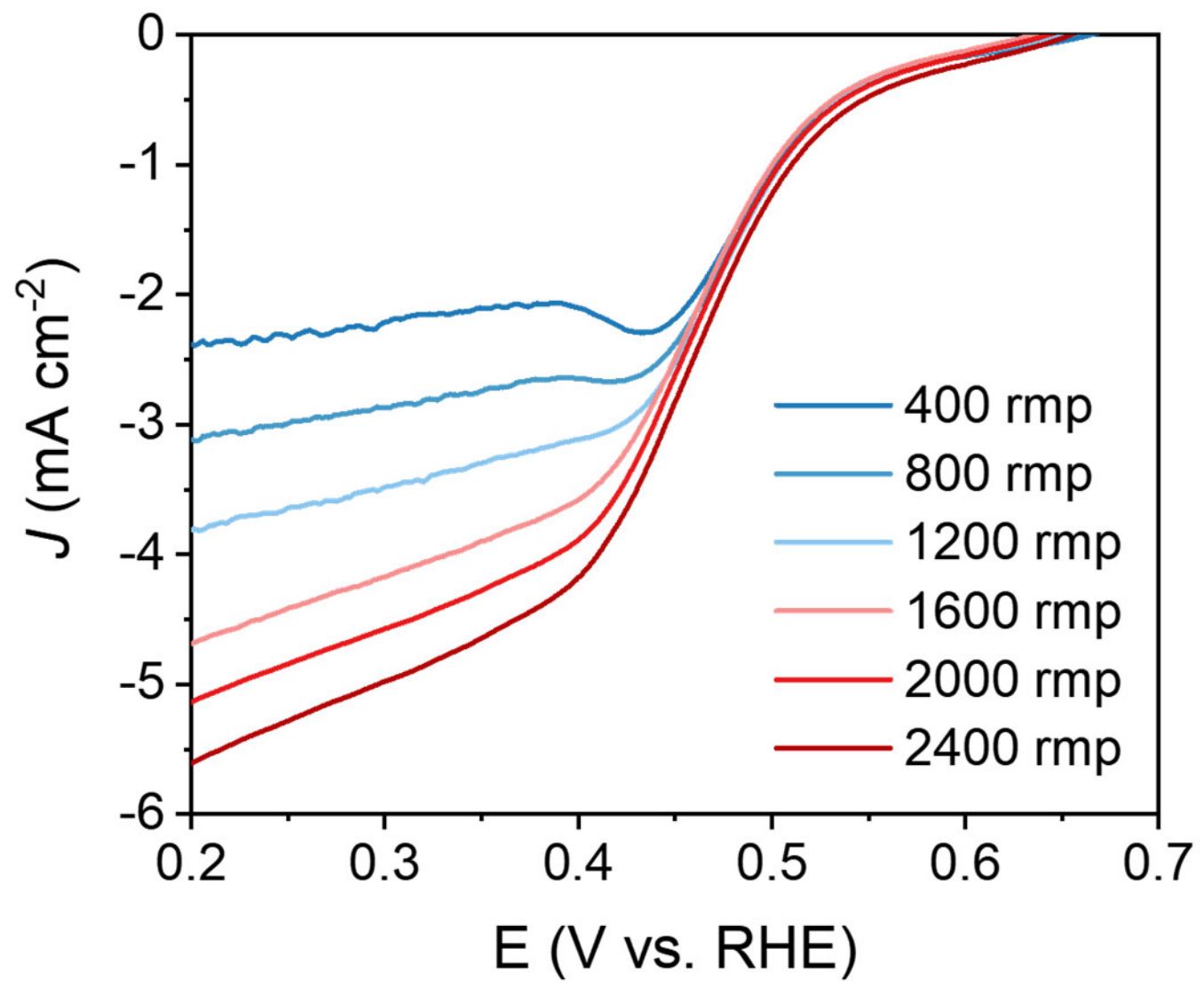

Supplementary Fig. 22 Oxygen reduction reaction profiles at 400 to 2400 rpm in O_2-saturated 1.0 M Na_2SO_4 for PSFZM. Source data for Supplementary Fig. 22 are provided as a Source Data file.

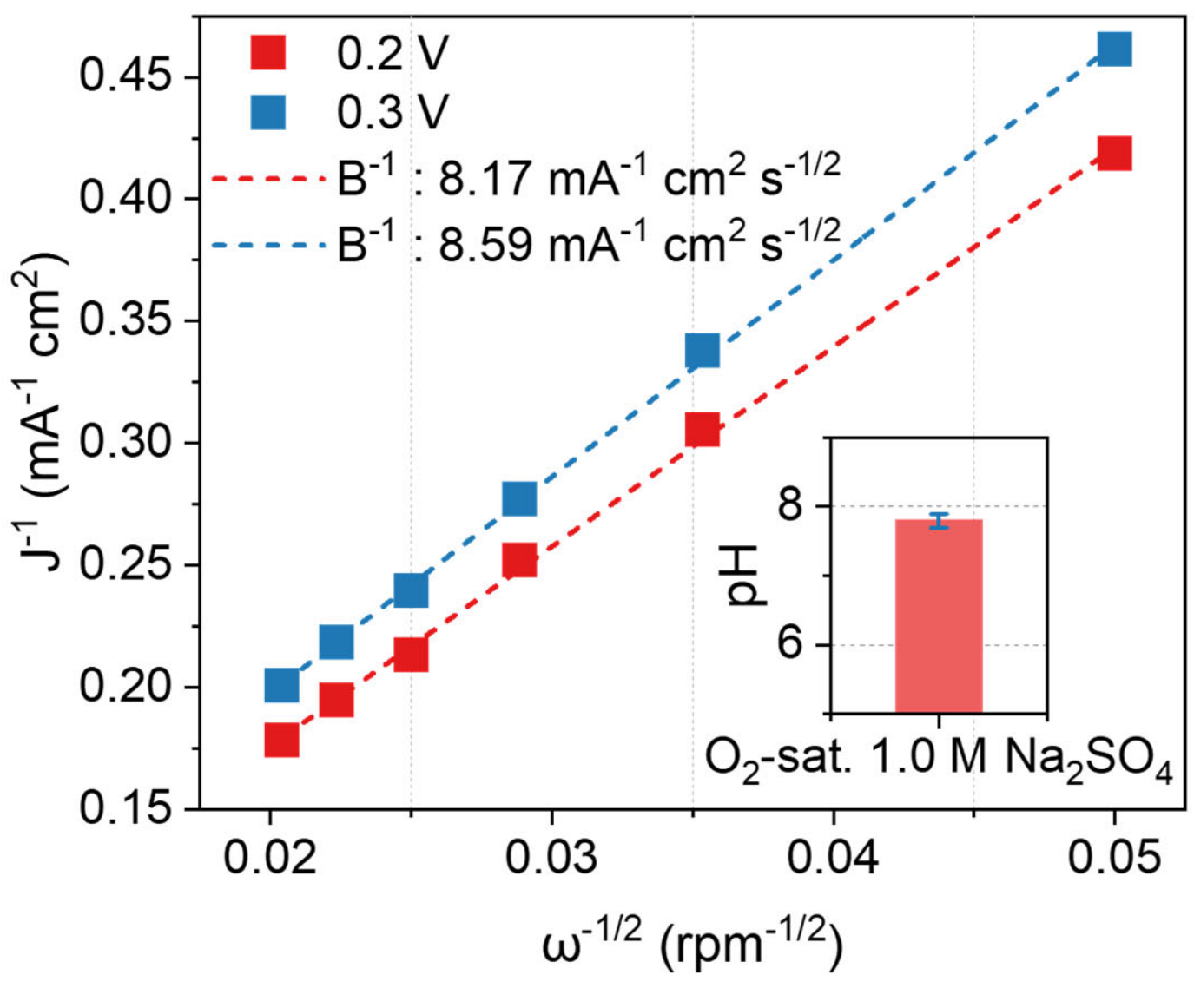

Supplementary Fig. 23 The corresponding Koutecky-Levich slope analysis for PSFZM. Source data for Supplementary Fig. 23 are provided as a Source Data file. The inset shows the pH measurement of the electrolyte.

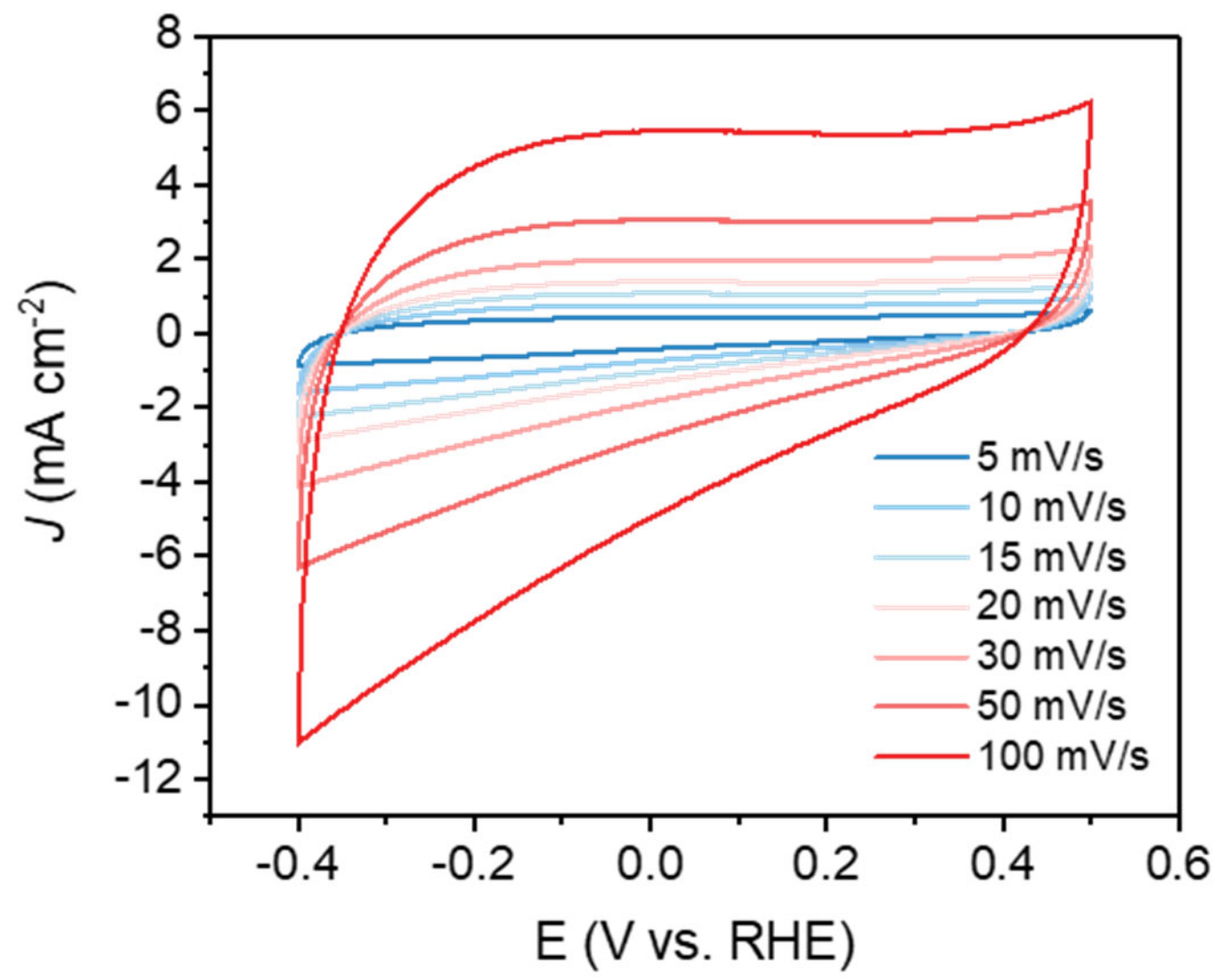

Supplementary Fig. 24 ECSA estimation is determined from C_{dl}. C_{dl} obtained by CV method is expected to be linearly proportional to ECSA. Scan rates in PSFZM are 5, 10, 15, 20, 30, 50 and 100 mV s^{-1}. Source data for Supplementary Fig. 24 are provided as a Source Data file.

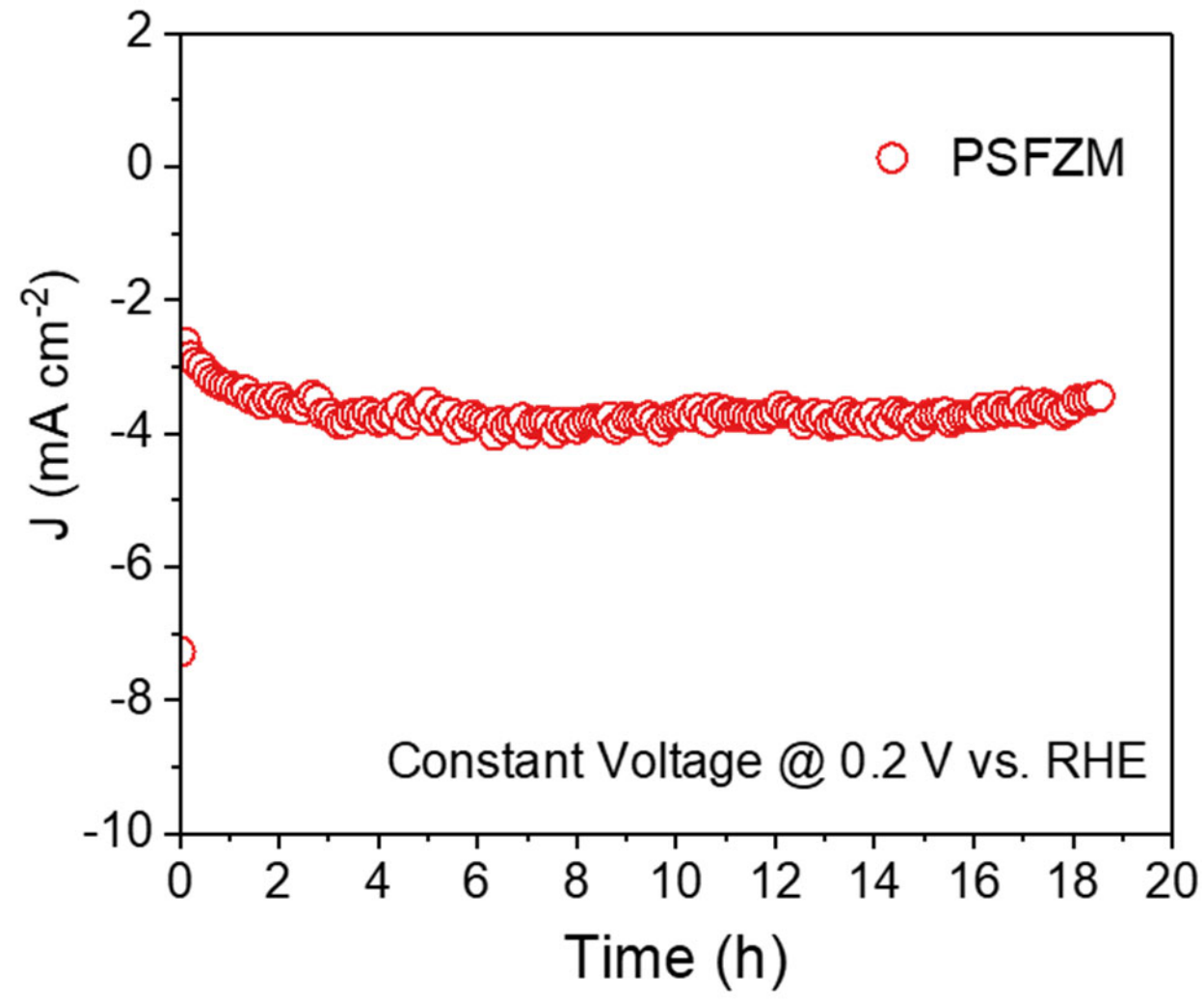

Supplementary Fig. 25 I-t stability curve of PSFZM at 0.2 V vs. RHE voltage. Source data for Supplementary Fig. 25 are provided as a Source Data file.

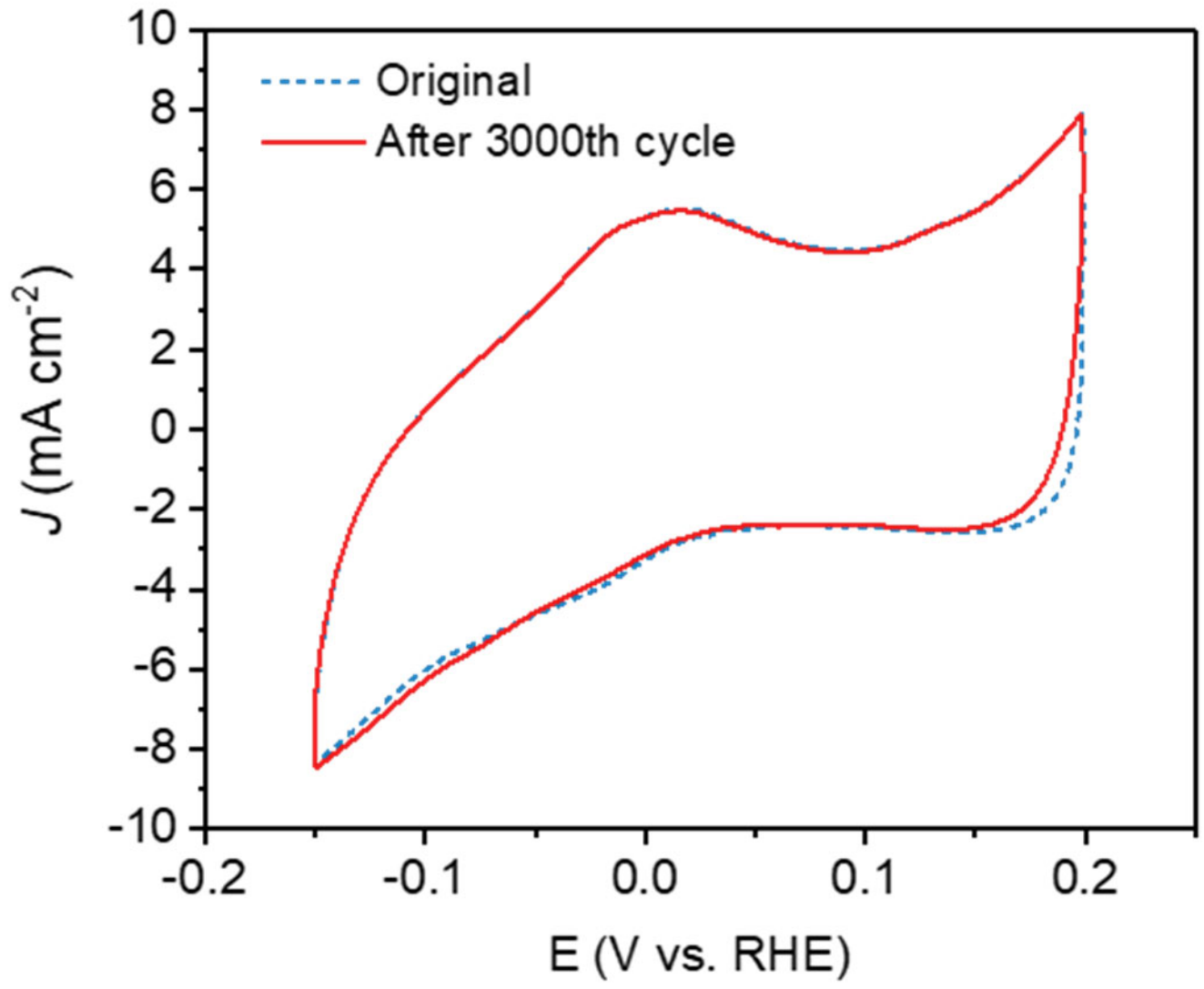

Supplementary Fig. 26 Comparison of CV curves of the sample after 3000th accelerated cycle with that of the initial sample. Source data for Supplementary Fig. 26 are provided as a Source Data file.

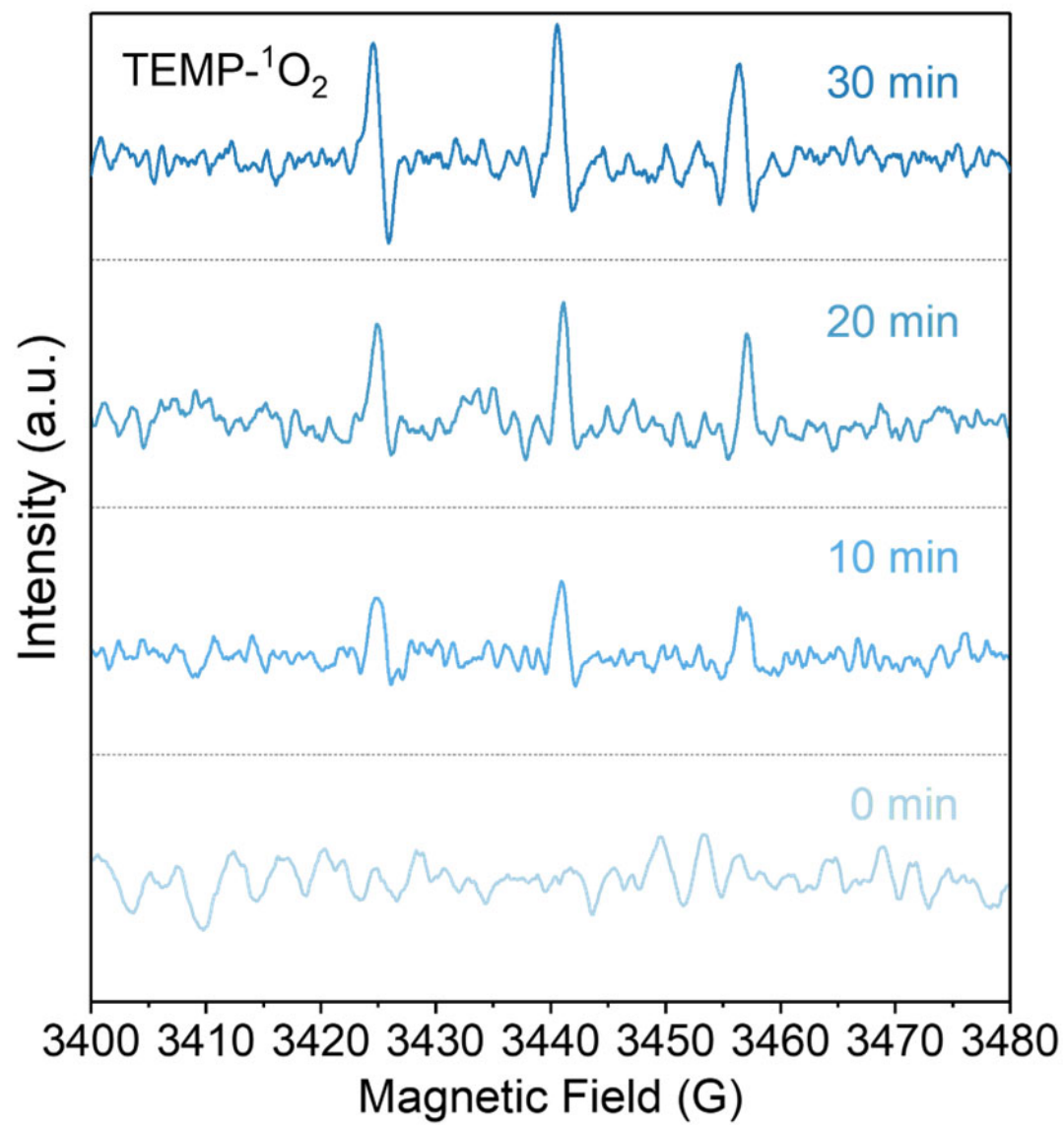

Supplementary Fig. 27 TEMP−$^1O_2$ adduct EPR spectra of ORR electrochemistry of PSFZM catalyst collected in incremental time configuration. Source data for Supplementary Fig. 27 are provided as a Source Data file.

To assess the dynamic changes of reactive oxygen species (ROS) under operating conditions, we performed time-dependent electron spin resonance (ESR) measurements (**Supplementary Fig. 27**). singlet oxygen ($^1O_2$) generation was confirmed using 2,2,6,6-tetramethyl-4-piperidinol (TEMP) as a trapping agent, producing a characteristic 1:1:1 triplet signal (αN = 16.9 G, g = 2.0054), which stabilized after 30 minutes of electrolysis.

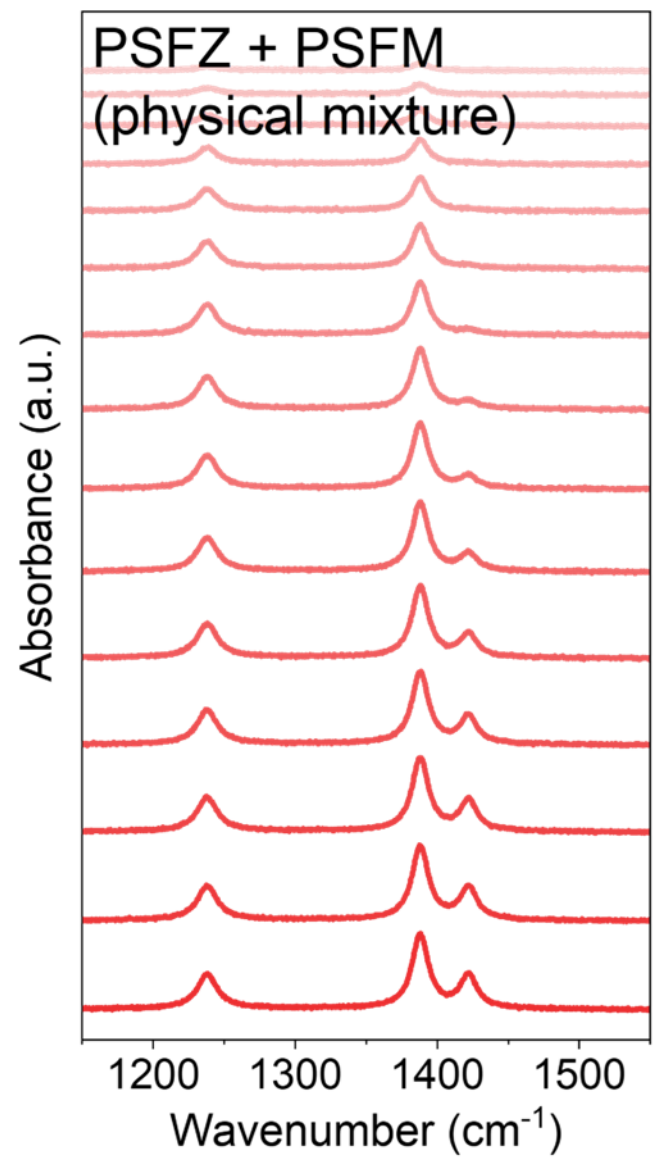

Supplementary Fig. 28 The ORR electrochemical in-situ FTIR spectra of PSFZ + PSFM physical mixture catalyst collected in an incremental voltage configuration. Source data for Supplementary Fig. 28 are provided as a Source Data file.

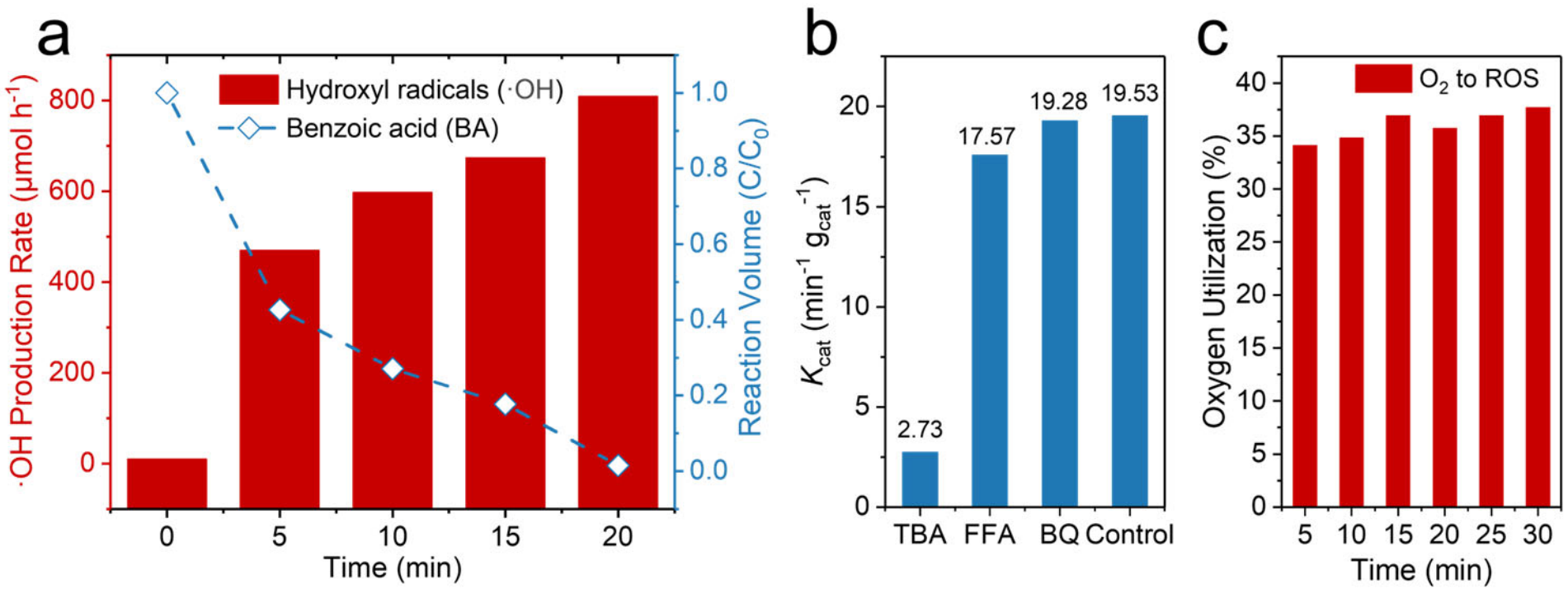

Supplementary Fig. 29 a Benzoic acid fluorescence spectrophotometry, used to detect the amount of •OH free radicals generated. **b** Effects of different free radical scavengers (30 mM BQ, 10 mM TBA and 10 mM FFA) on NOR degradation. **c** Oxygen utilization during 30-min continuous testing. Source data for Supplementary Fig. 29 are provided as a Source Data file.

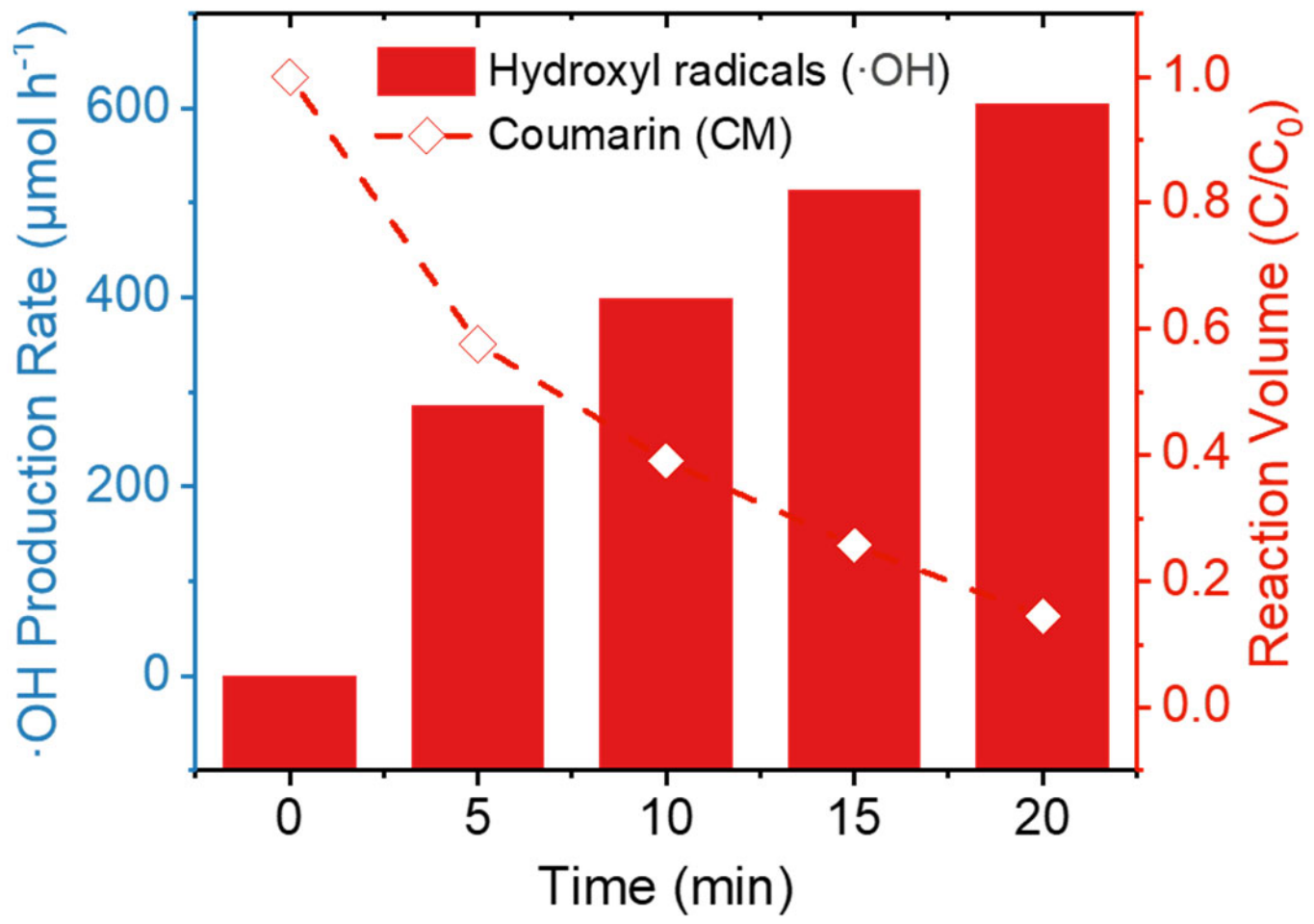

Supplementary Fig. 30 The PSFZM electrode was used to detect the generation of •OH radicals using coumarin fluorescence spectrophotometry after 20 min ORR process. Source data for Supplementary Fig. 30 are provided as a Source Data file.

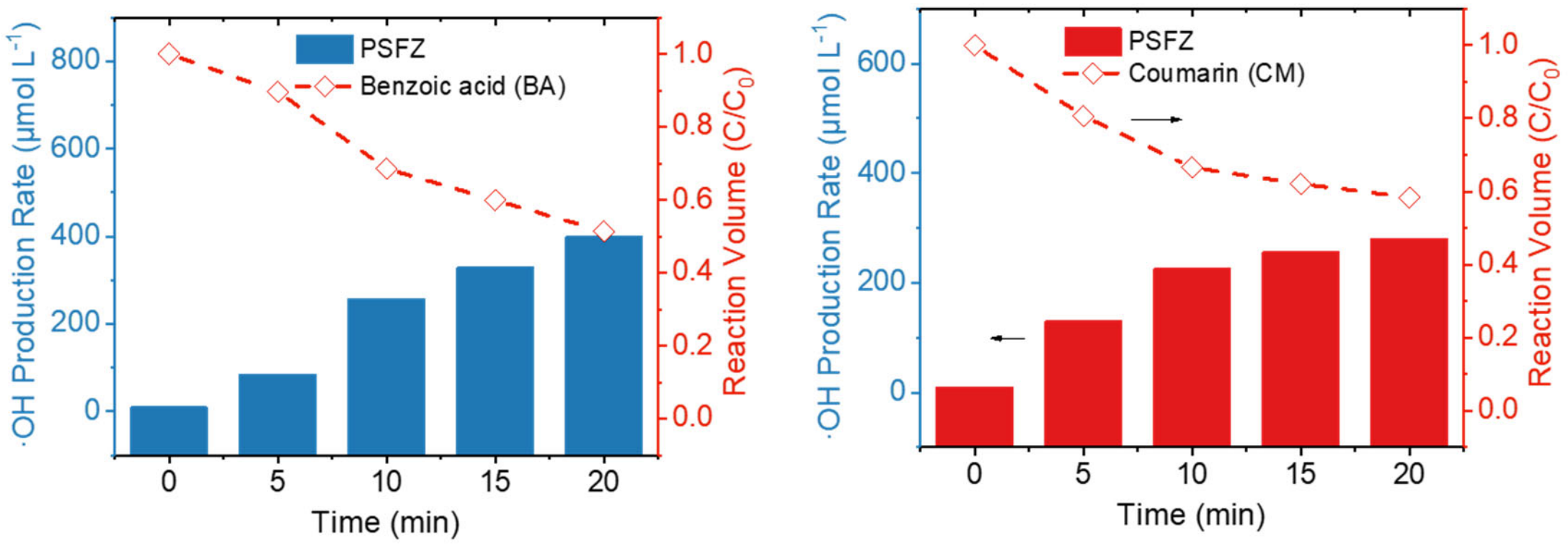

Supplementary Fig. 31 The PSFZ electrode was used to detect the generation of •OH radicals after 20 min ORR process using benzoic acid and coumarin fluorescence spectrophotometry, respectively. Source data for Supplementary Fig. 31 are provided as a Source Data file.

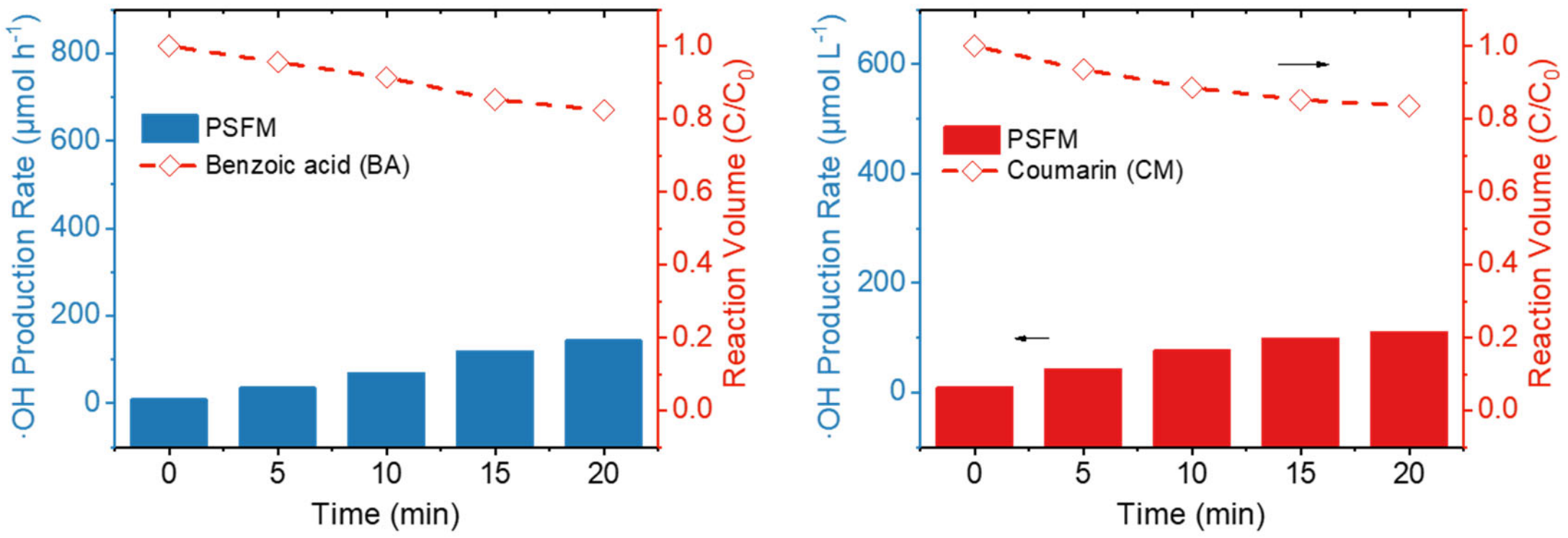

Supplementary Fig. 32 The PSFM electrode was used to detect the generation of •OH radicals after 20 min ORR process using benzoic acid and coumarin fluorescence spectrophotometry, respectively. Source data for Supplementary Fig. 32 are provided as a Source Data file.

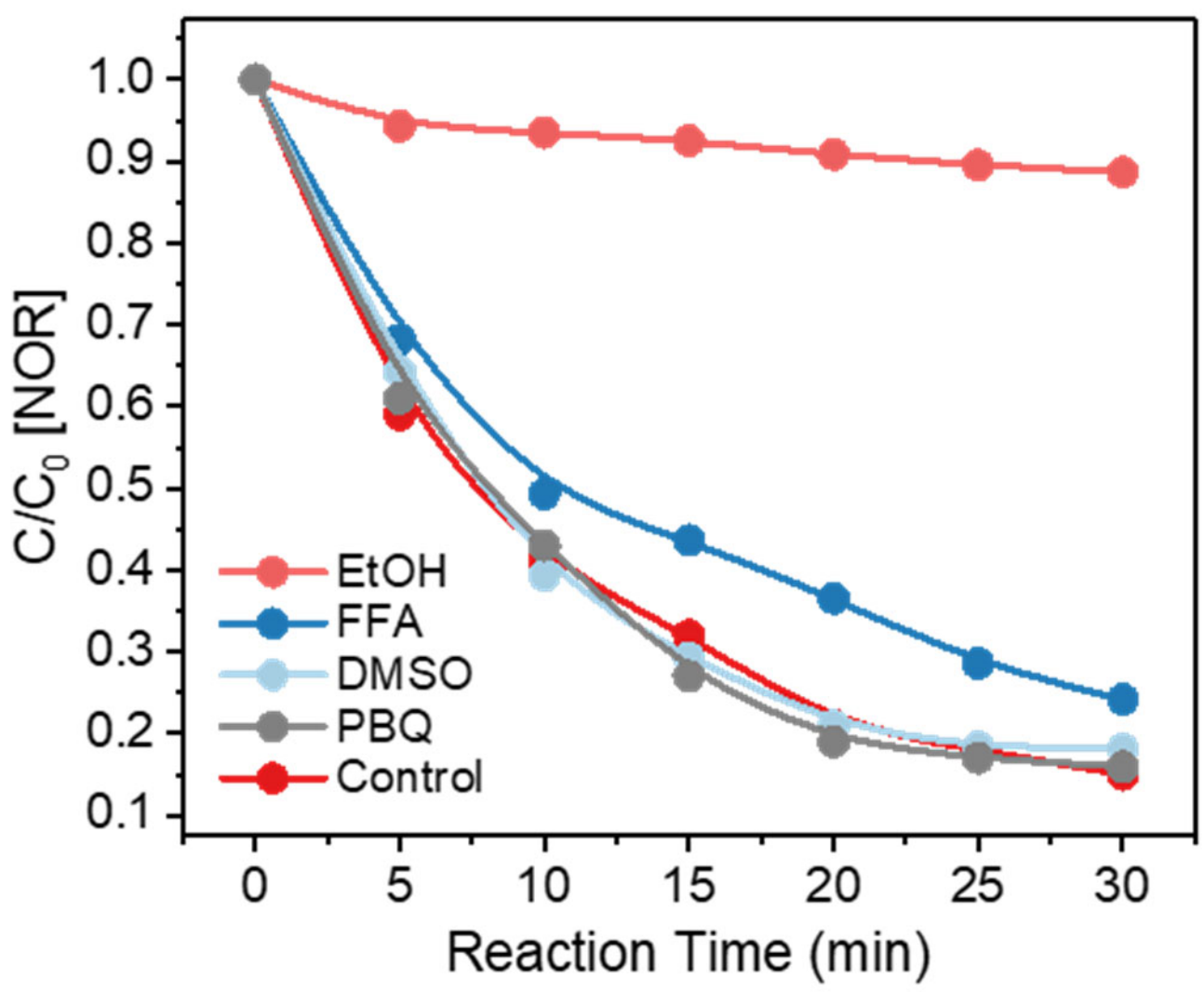

Supplementary Fig. 33 In the PSFZM system, the effects of different free radical scavengers (30 mM EtOH, 10 mM PBQ, 10 mM DMSO and 10 mM FFA) on the degradation of norfloxacin were investigated. Source data for Supplementary Fig. 33 are provided as a Source Data file.

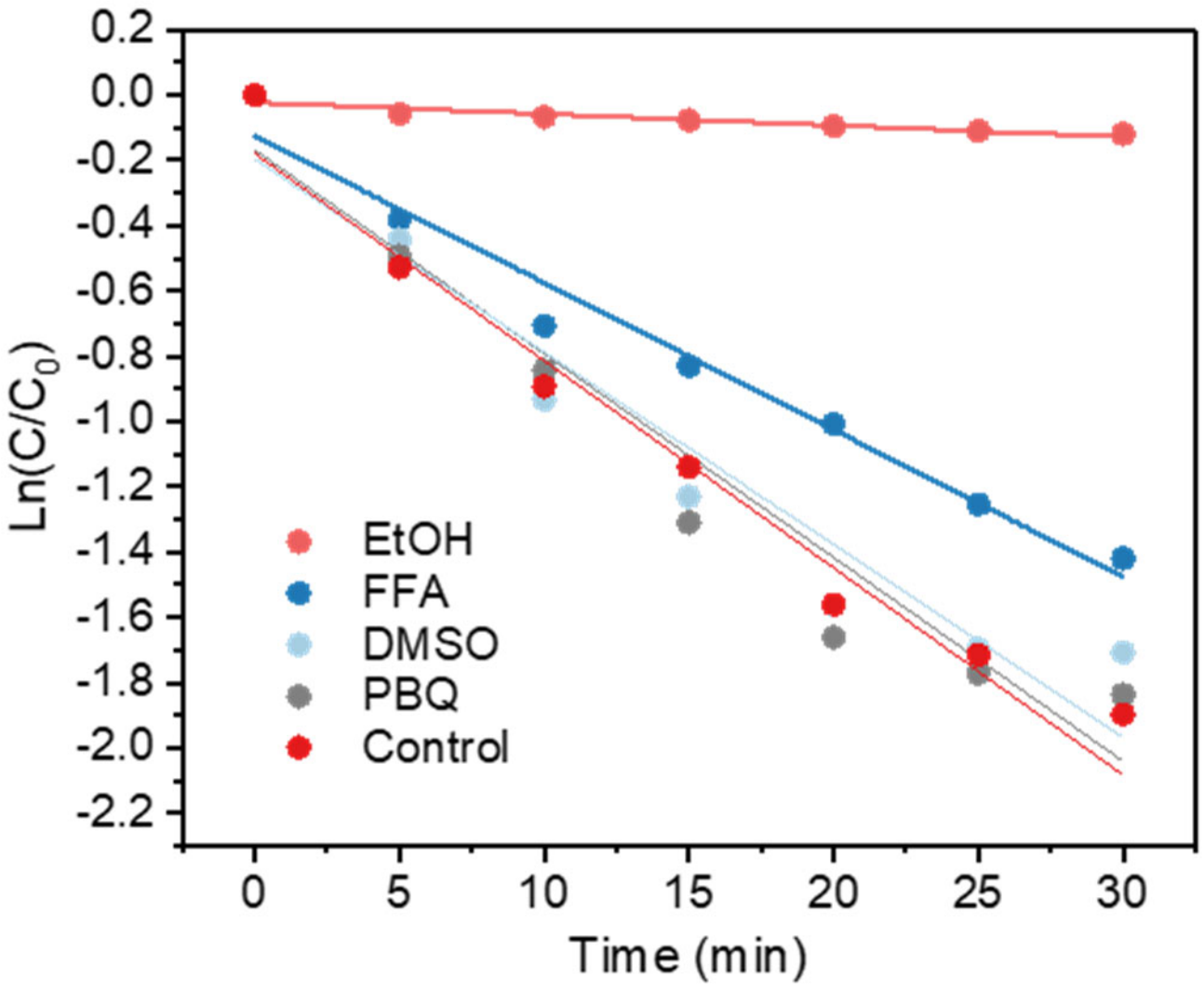

Supplementary Fig. 34 The first-order kinetics of norfloxacin degradation by different free radical scavengers were investigated in the PSFZM system. Source data for Supplementary Fig. 34 are provided as a Source Data file.

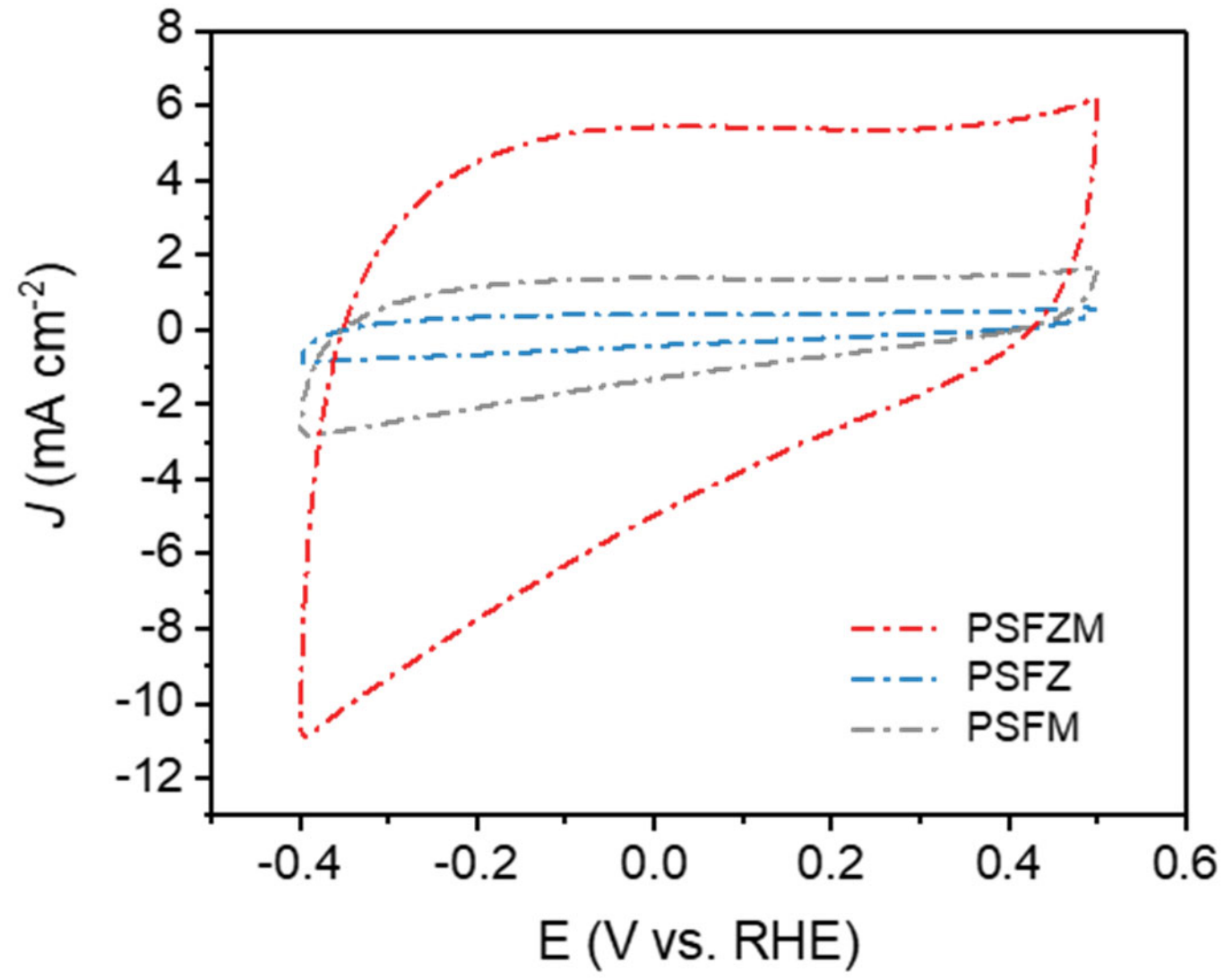

Supplementary Fig. 35 CV curves of PSFZM, PSFZ, and PSFM in Ar-saturated 1 M KOH, where the redox peaks indicate electrochemical oxygen insertion/deinsertion. Source data for Supplementary Fig. 35 are provided as a Source Data file.

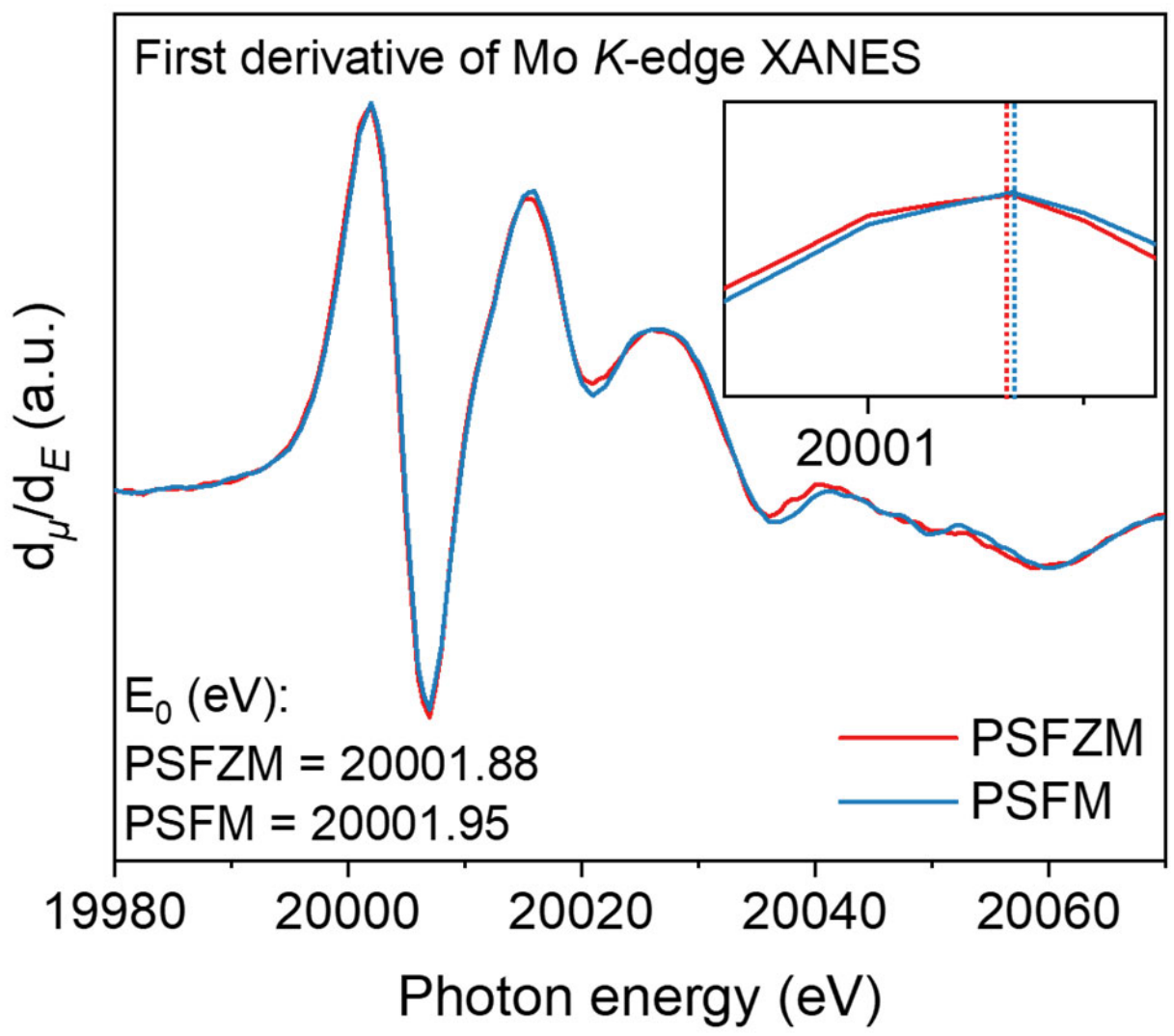

Supplementary Fig. 36 First-derivative Mo *K*-edge XANES spectra of PSFZM and PSFM. Source data for Supplementary Fig. 36 are provided as a Source Data file.

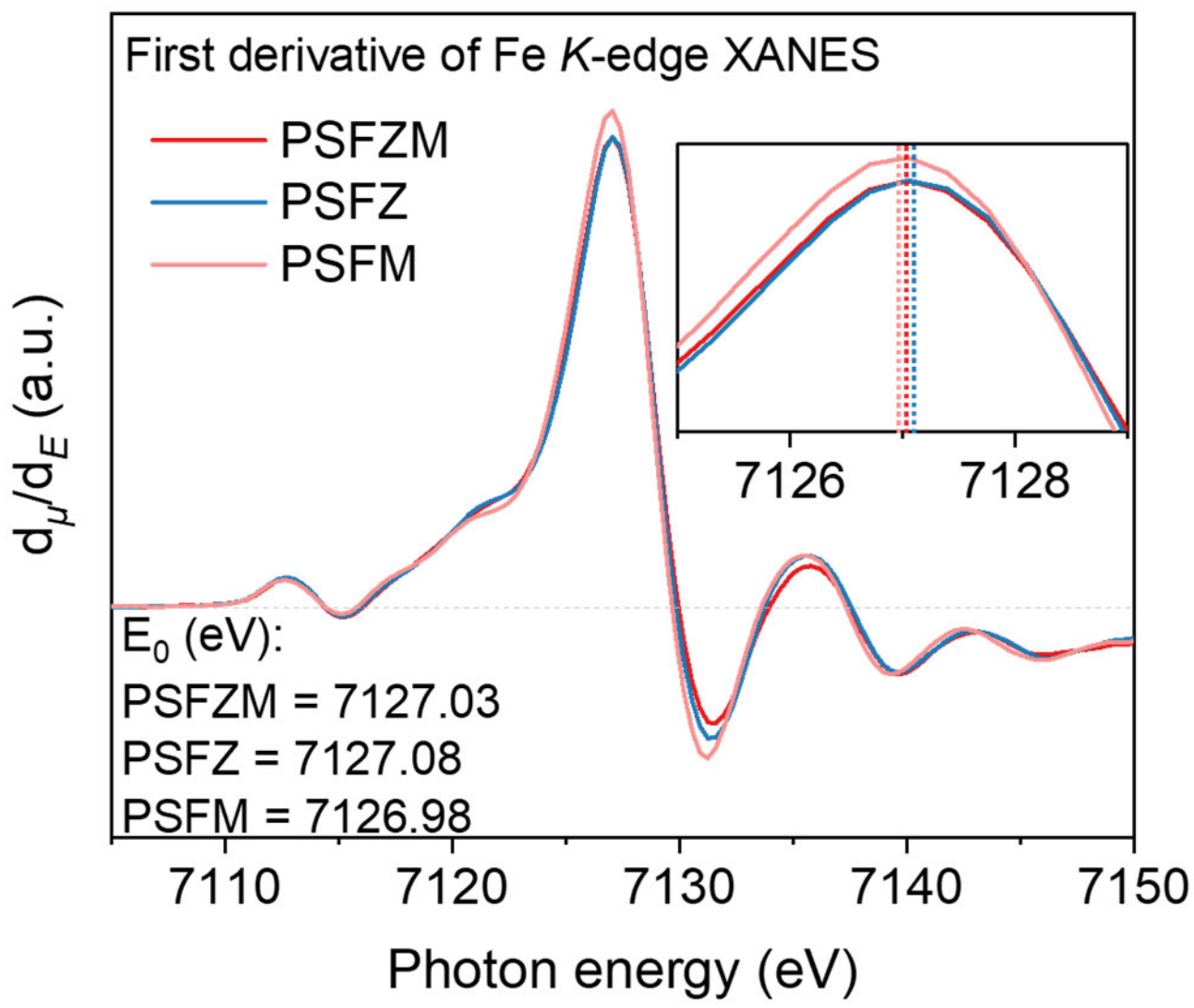

Supplementary Fig. 37 First-derivative Fe *K*-edge XANES spectra of PSFZM, PSFZ and PSFM. Source data for Supplementary Fig. 37 are provided as a Source Data file.

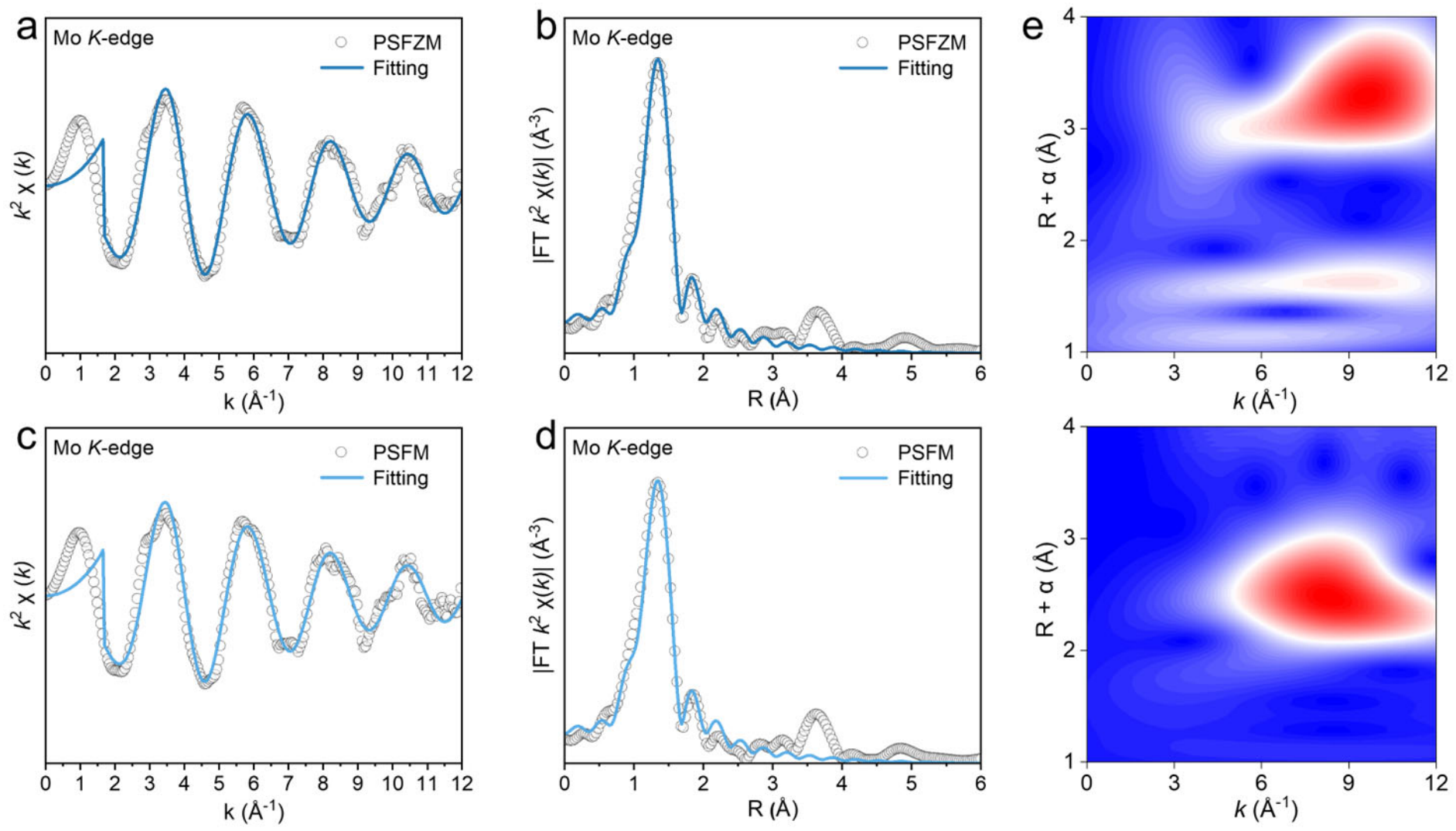

Supplementary Fig. 38 Fitting of the Mo *K*-edge EXAFS K spectra for **a** PSFZM and **c** PSFM. Fitting of the Mo *K*-edge EXAFS R space fitting result for **b** PSFZM and **d** PSFM. **e** WT-EXAFS spectrum of MoO_3 and Mo foil. Source data for Supplementary Fig. 38 are provided as a Source Data file.

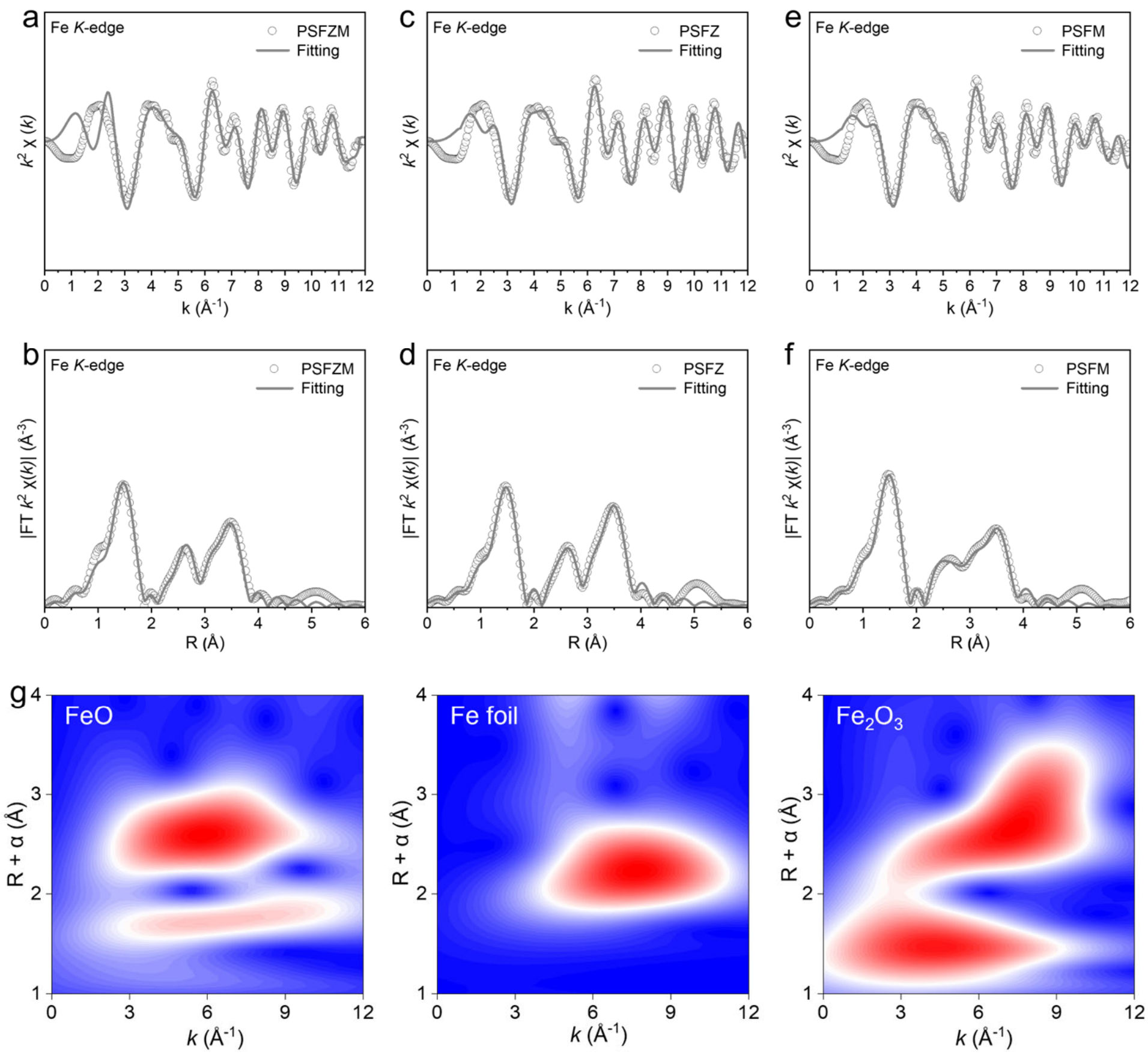

Supplementary Fig. 39 Fitting of the Fe *K*-edge EXAFS K spectra for **a** PSFZM, **c** PSFZ and **e** PSFM. Fitting of the Fe *K*-edge EXAFS R space fitting result for **b** PSFZM, **d** PSFZ and **f** PSFM. **g** WT-EXAFS spectrum of FeO, Fe foil and Fe_2O_3. Source data for Supplementary Fig. 39 are provided as a Source Data file.

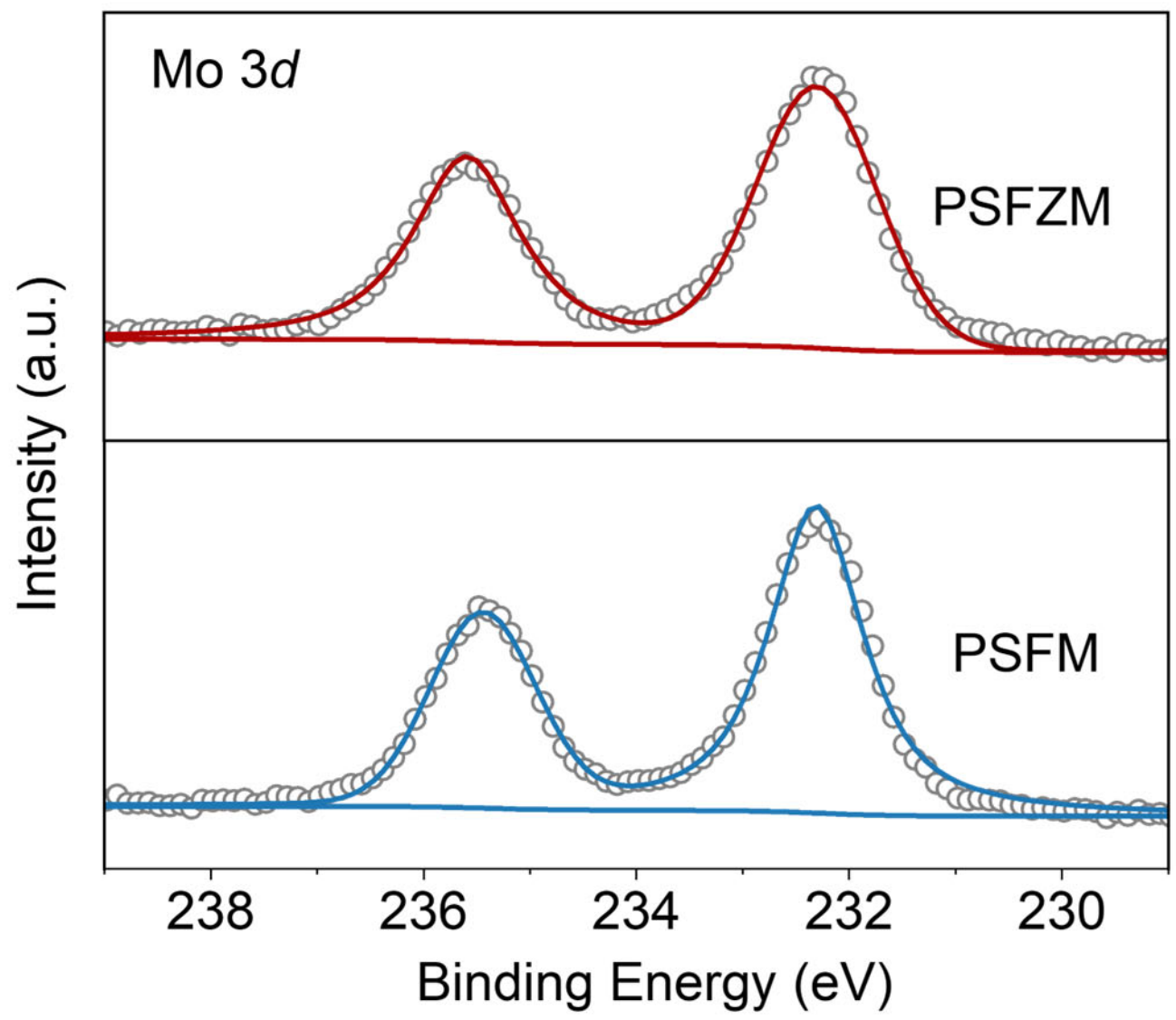

Supplementary Fig. 40 High-resolution surface XPS scan of Mo 3*d* of PSFZM and PSFM oxide. Source data for Supplementary Fig. 40 are provided as a Source Data file.

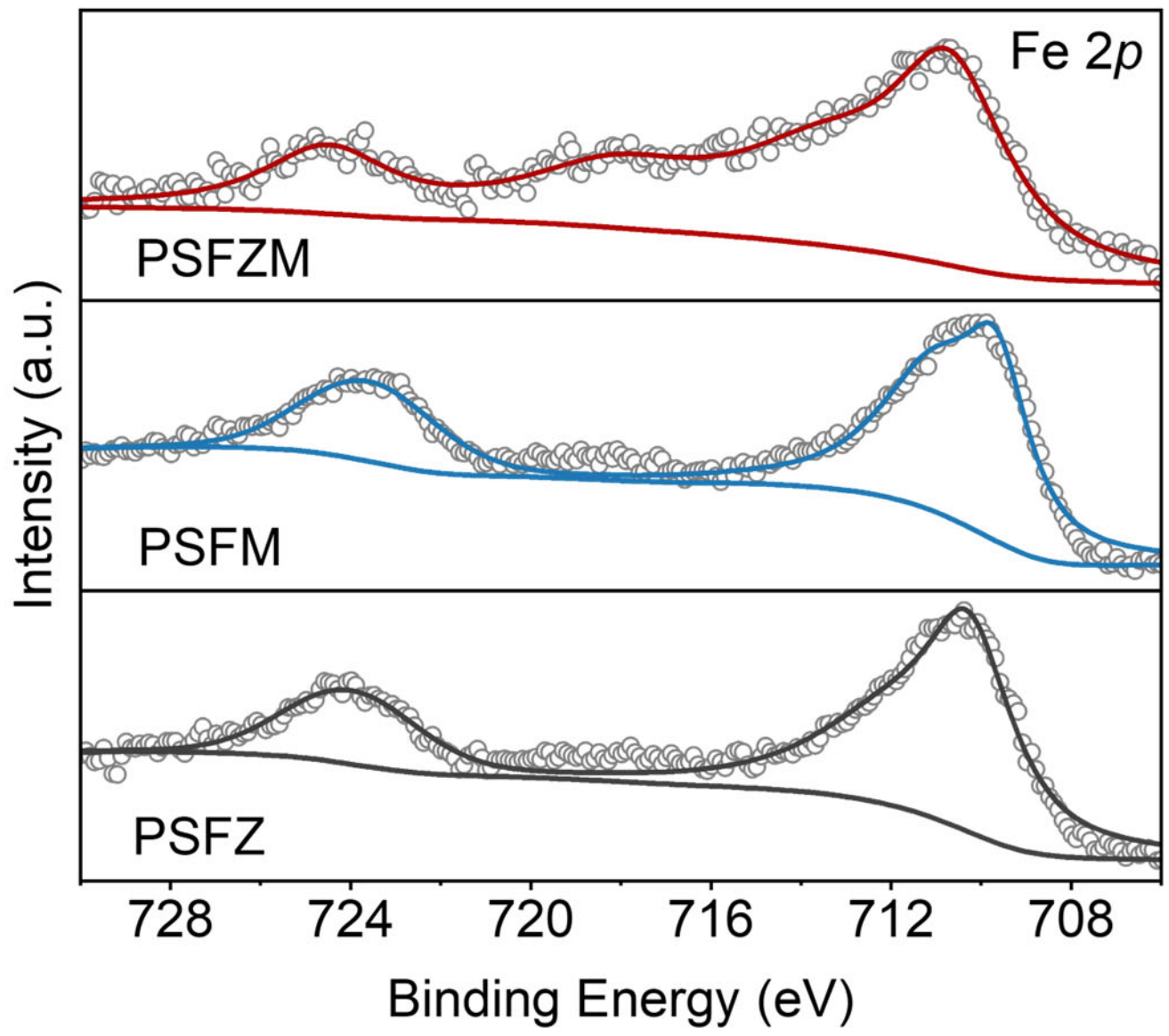

Supplementary Fig. 41 High-resolution surface XPS scan of Fe 2*p* of PSFZM, PSFM and PSFZ oxide. Source data for Supplementary Fig. 41 are provided as a Source Data file.

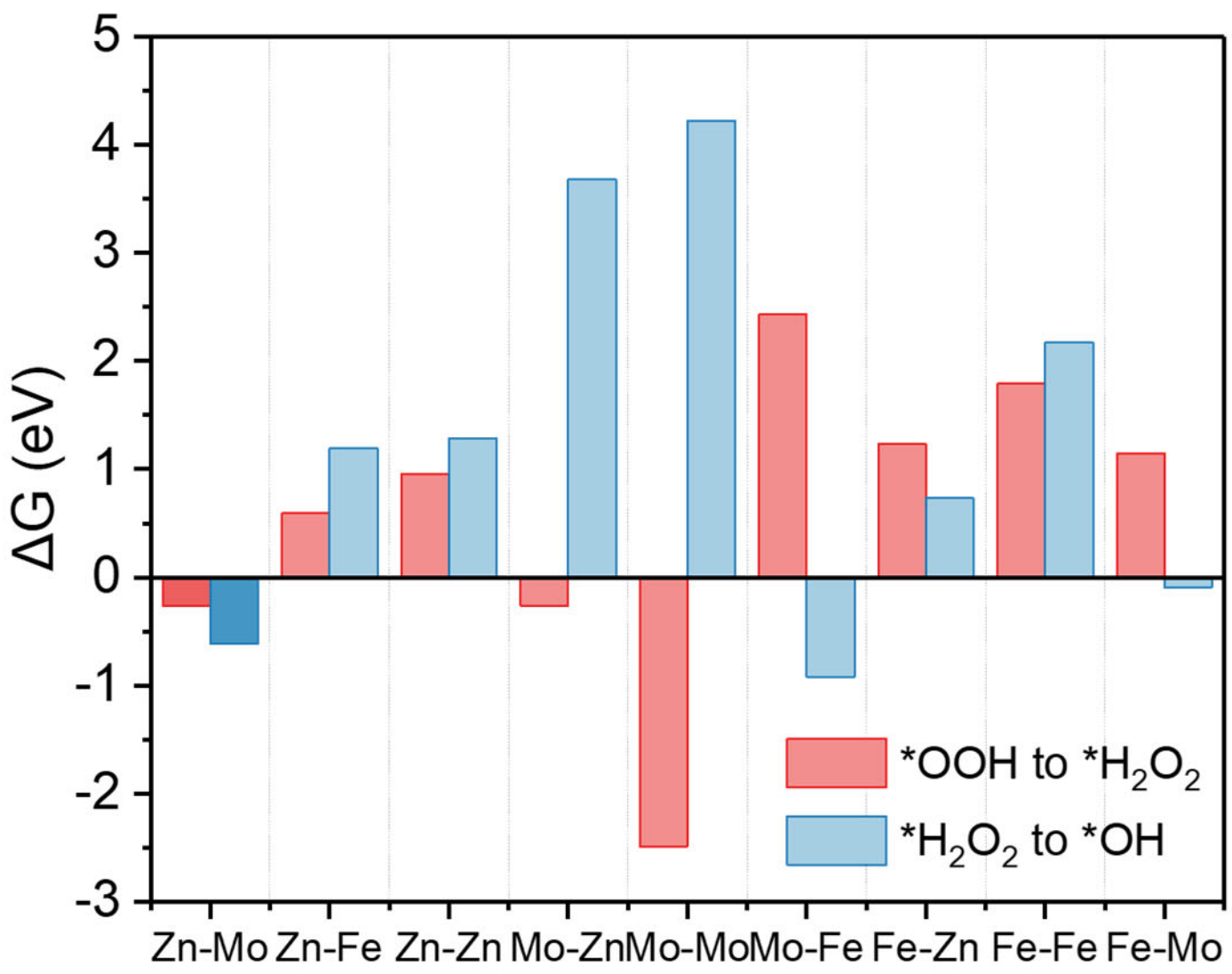

Supplementary Fig. 42 Gibbs free energy changes for the conversion of *OOH to *H_2O_2 and *H_2O_2 to •OH intermediates at the active sites with different coordination situations at the B site of PSFZM perovskite oxides. Source data for Supplementary Fig. 42 are provided as a Source Data file.

As shown in **Supplementary Fig. 42**, the Zn-Mo dual-site configuration in PSFZM exhibits an optimal balance between *OOH adsorption and H_2O_2 decomposition, enabling spontaneous •OH generation. Mechanistically, O_2 first adsorbs onto the Zn site to form *OOH, which is then stabilized via electronic interaction with a neighboring Mo site. This heteronuclear Zn-O-Mo bridge disrupts the symmetric electron cloud distribution, alters *H_2O_2 adsorption geometry, and facilitates O–O bond cleavage to yield •OH.

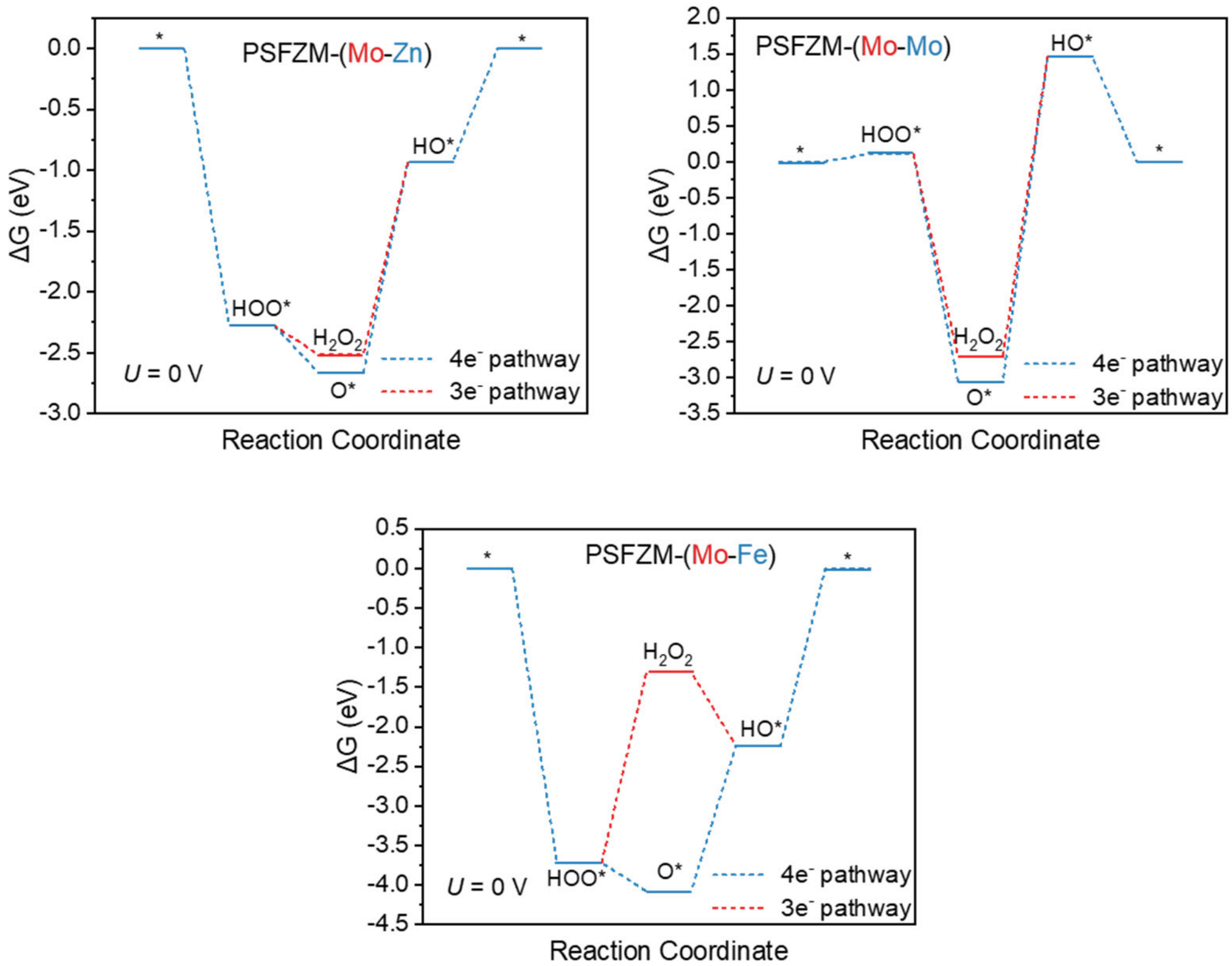

Supplementary Fig. 43 Complete Gibbs free energy process of $3e^-$ and $4e^-$ ORR pathway at Mo site of PSFZM. Source data for Supplementary Fig. 43 are provided as a Source Data file.

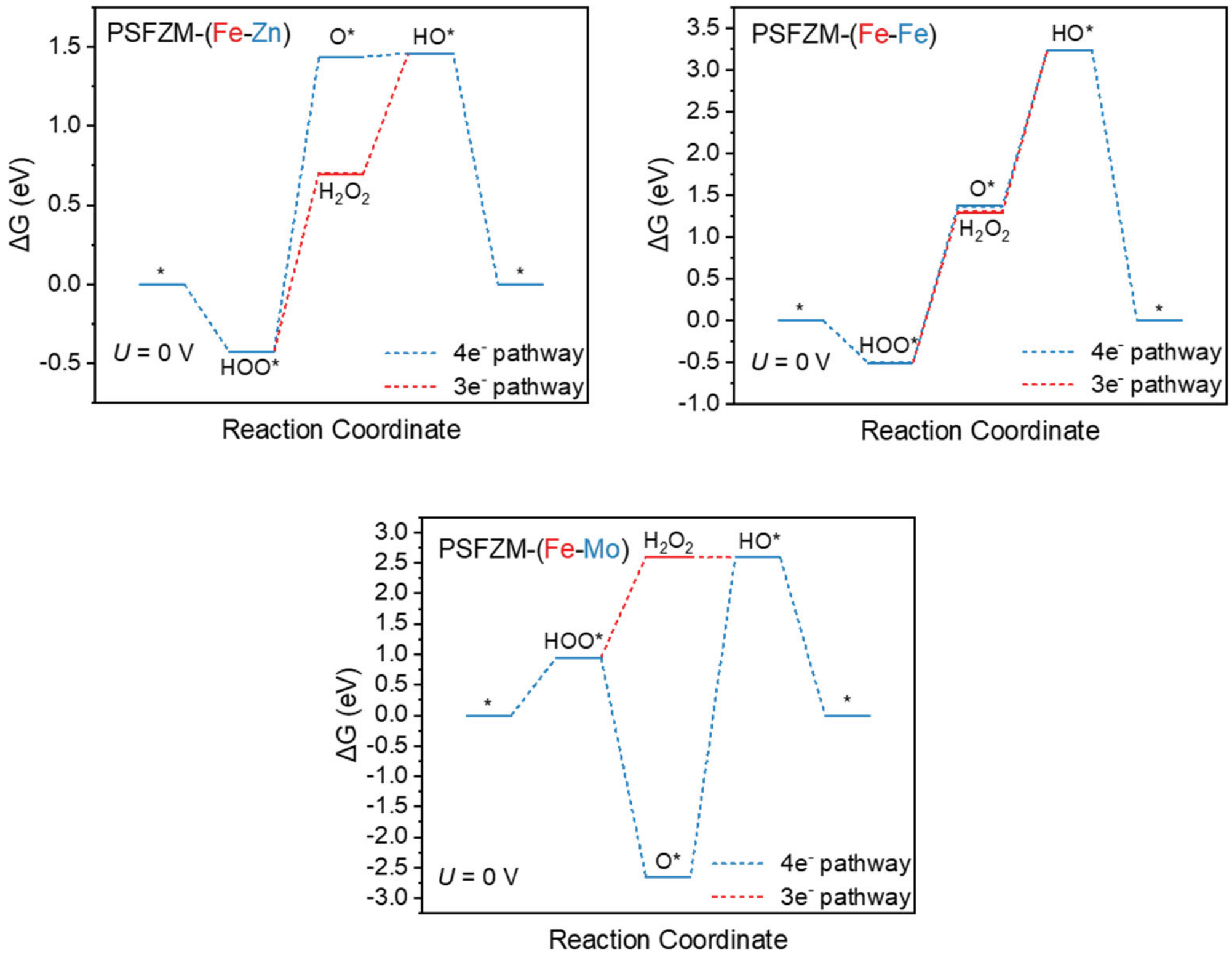

Supplementary Fig. 44 Complete Gibbs free energy process of $3e^-$ and $4e^-$ ORR pathway at Fe site of PSFZM. Source data for Supplementary Fig. 44 are provided as a Source Data file.

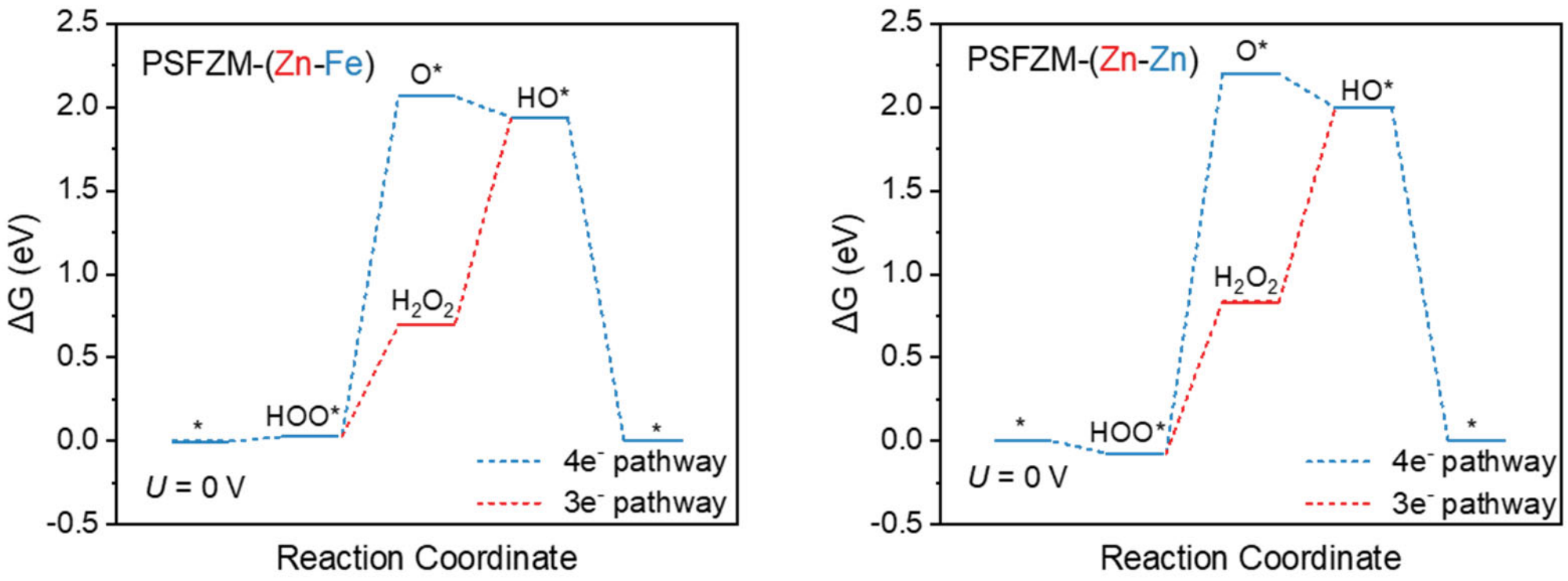

Supplementary Fig. 45 Complete Gibbs free energy process of 3e$^-$ and 4e$^-$ ORR pathway at Zn site of PSFZM. Source data for Supplementary Fig. 45 are provided as a Source Data file.

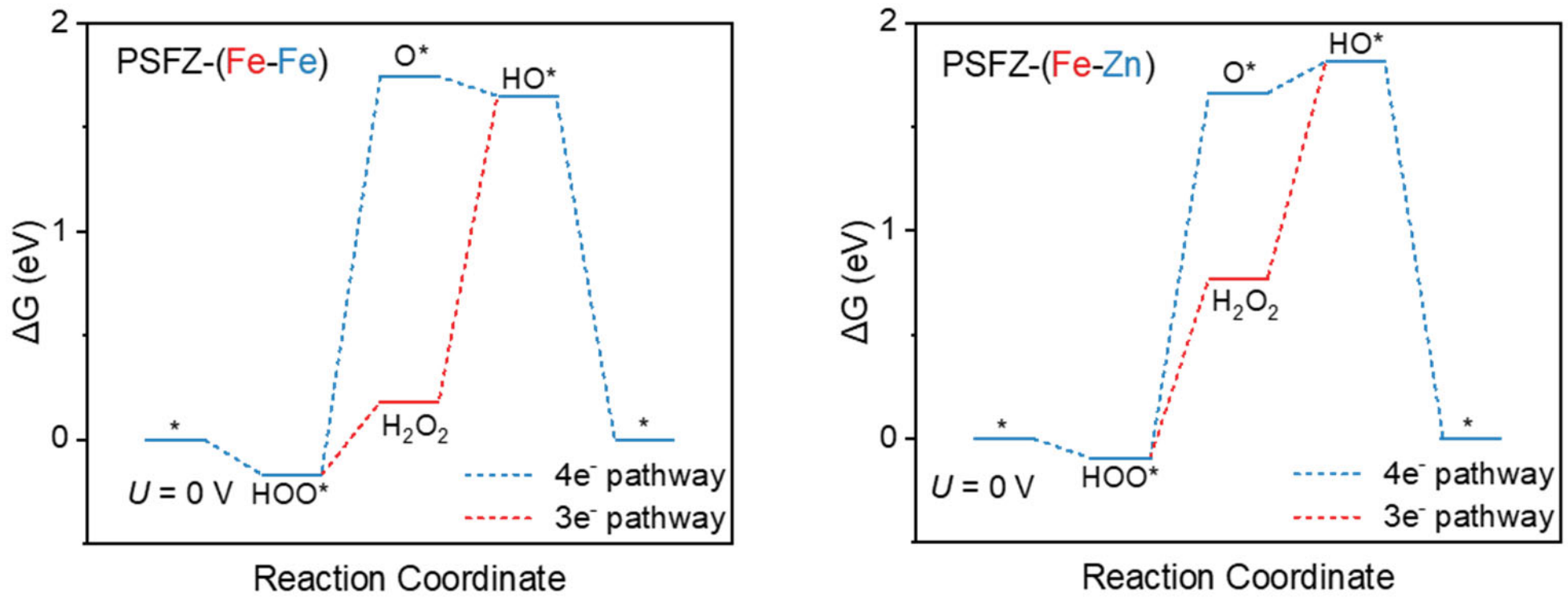

Supplementary Fig. 46 Complete Gibbs free energy process of 3e$^-$ and 4e$^-$ ORR pathway at Fe site of PSFZ. Source data for Supplementary Fig. 46 are provided as a Source Data file.

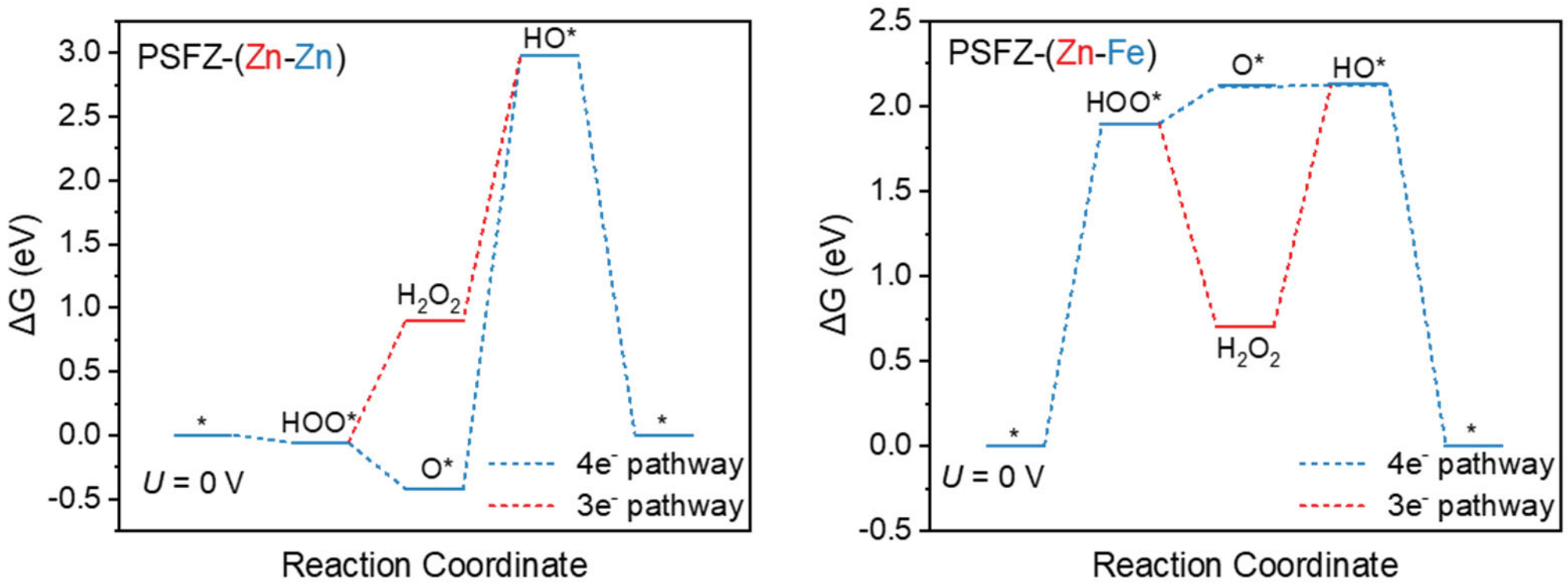

Supplementary Fig. 47 Complete Gibbs free energy process of 3e$^-$ and 4e$^-$ ORR pathway at Zn site of PSFZ. Source data for Supplementary Fig. 47 are provided as a Source Data file.

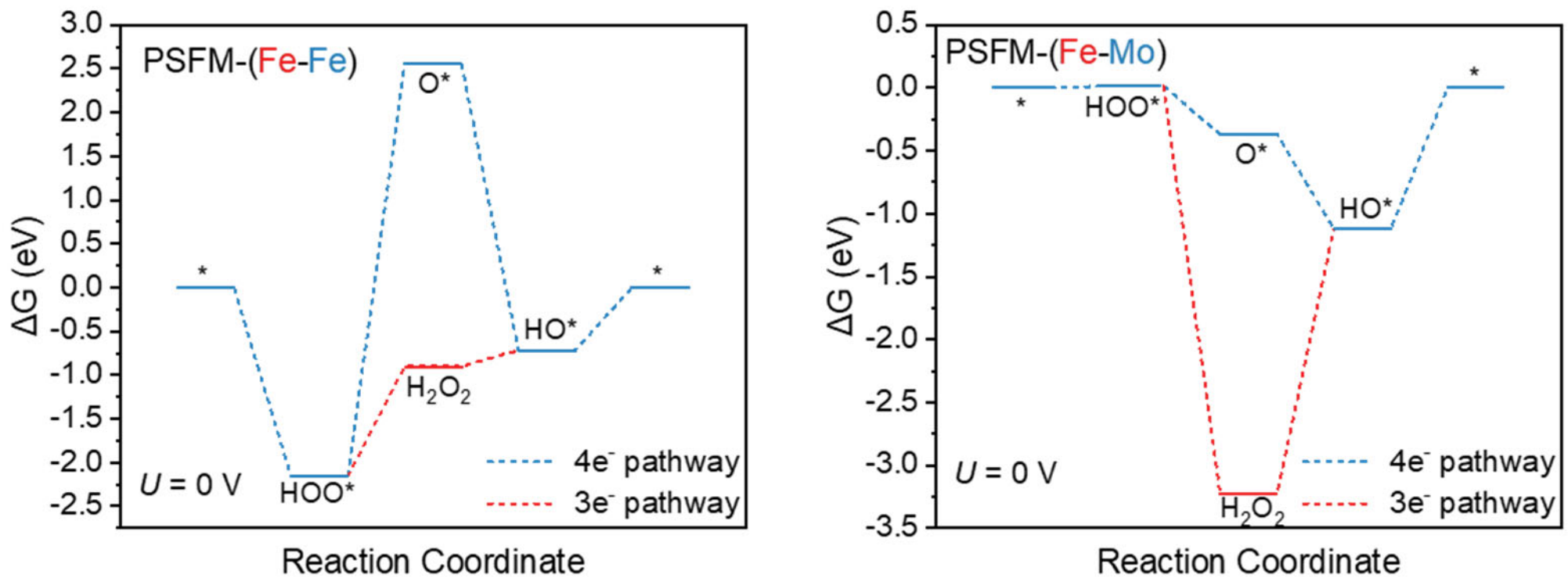

Supplementary Fig. 48 Complete Gibbs free energy process of $3e^-$ and $4e^-$ ORR pathway at Fe site of PSFM. Source data for Supplementary Fig. 48 are provided as a Source Data file.

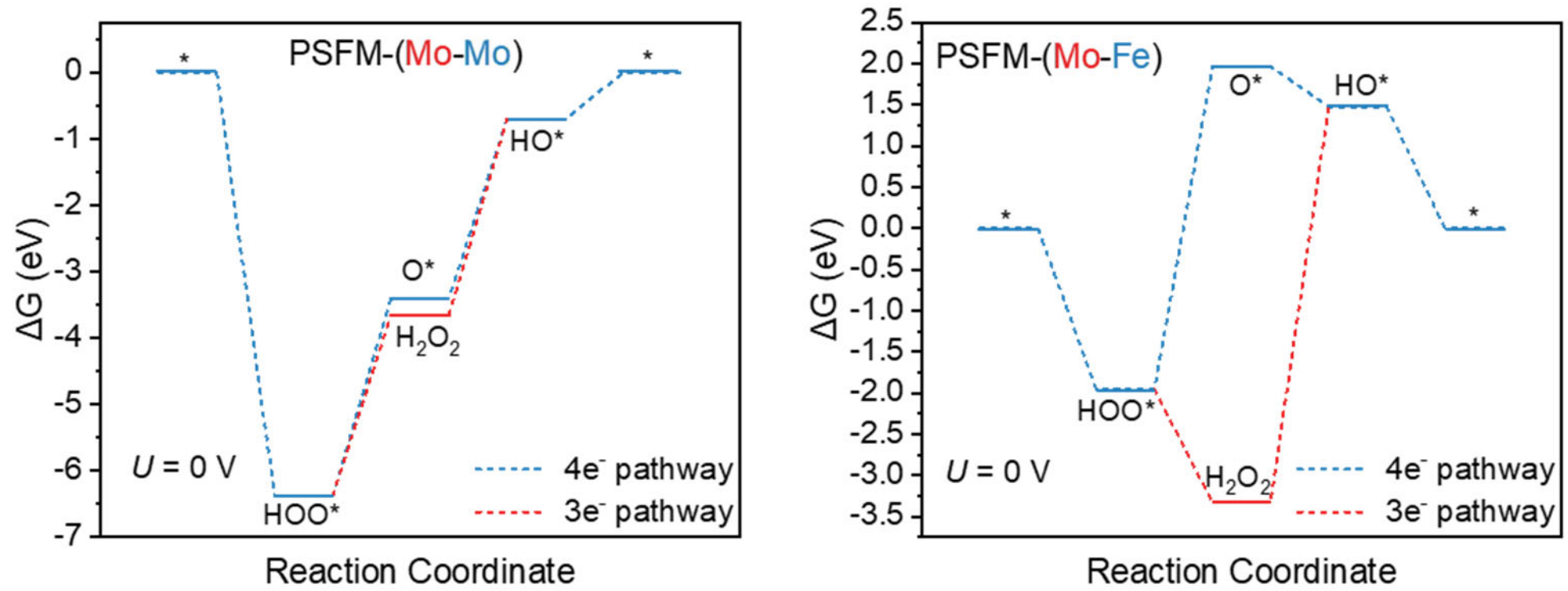

Supplementary Fig. 49 Complete Gibbs free energy process of $3e^-$ and $4e^-$ ORR pathway at Mo site of PSFM. Source data for Supplementary Fig. 49 are provided as a Source Data file.

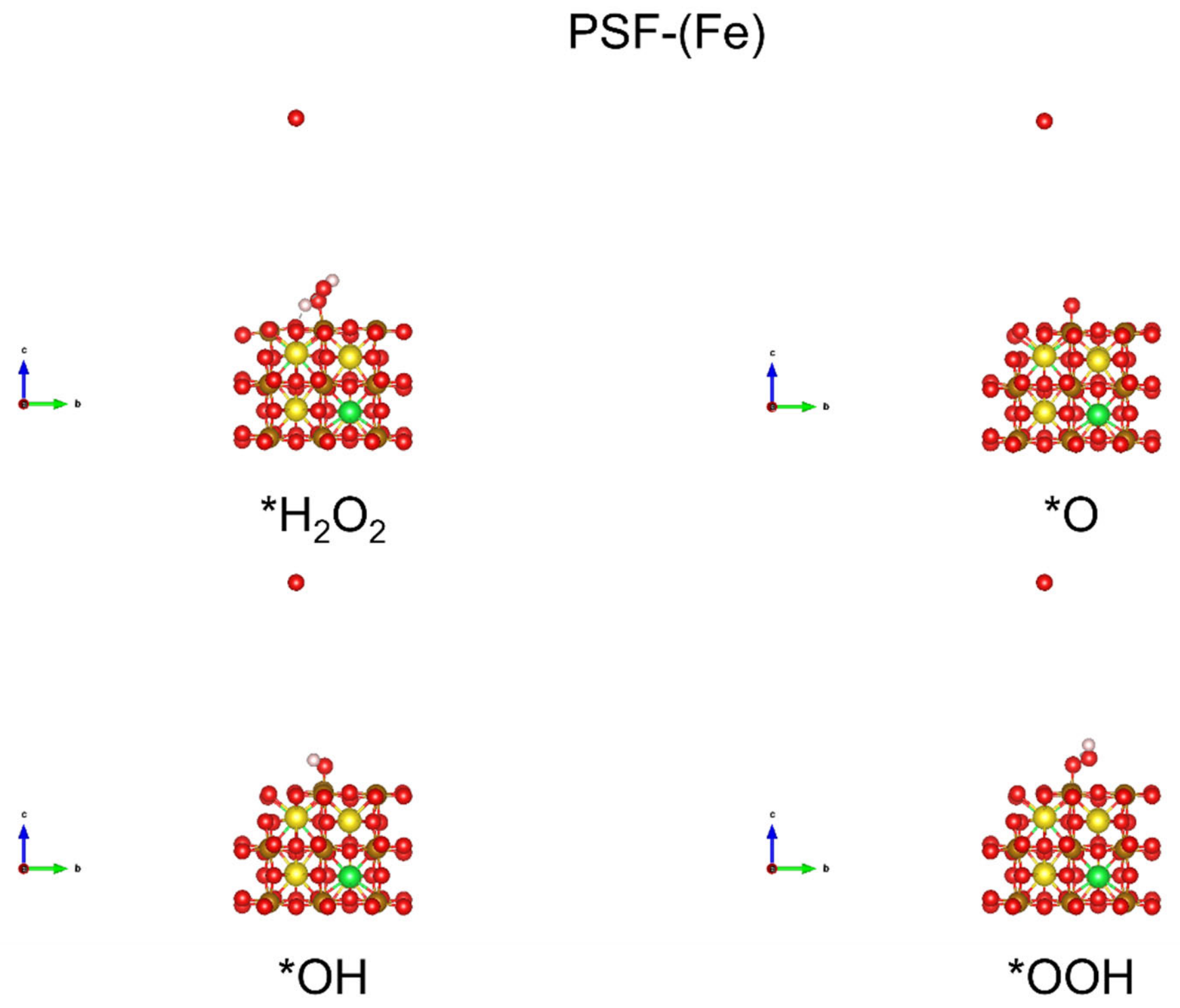

Supplementary Fig. 50 Side-view structure of the adsorbed intermediates $*H_2O_2$, *O, *OH, and *OOH on the PSF- (Fe) site. Colors represent: Pr (yellow), Sr (green), Fe (brown), Zn (grey), Mo (pink), O (red), H (white).

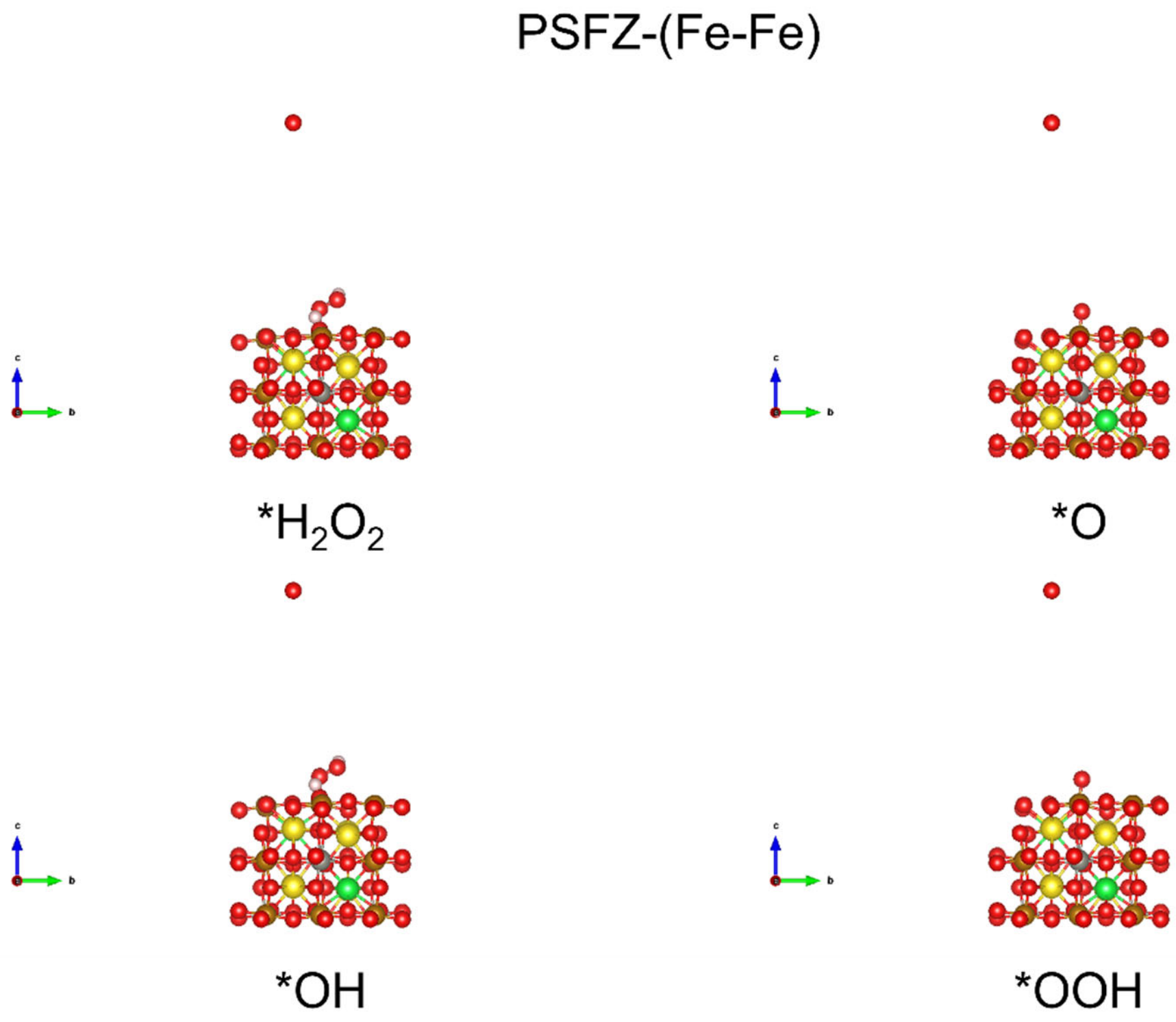

Supplementary Fig. 51 Side-view structure of the adsorbed intermediates $*H_2O_2$, *O, *OH, and *OOH on the PSFZ- (Fe-Fe) site. Colors represent: Pr (yellow), Sr (green), Fe (brown), Zn (grey), Mo (pink), O (red), H (white).

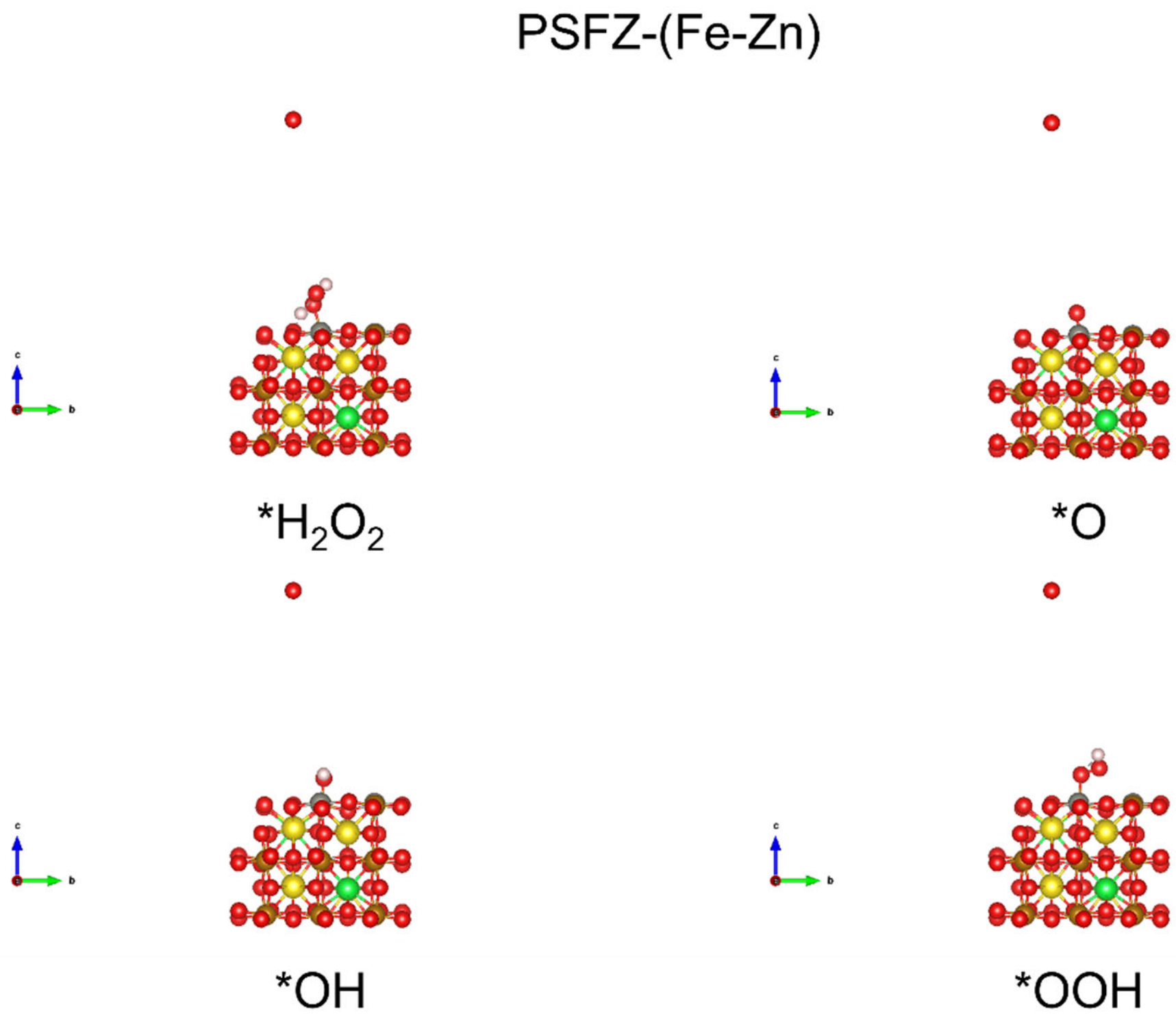

Supplementary Fig. 52 Side-view structure of the adsorbed intermediates *H_2O_2, *O, *OH, and *OOH on the PSFZ- (Fe-Zn) site. Colors represent: Pr (yellow), Sr (green), Fe (brown), Zn (grey), Mo (pink), O (red), H (white).

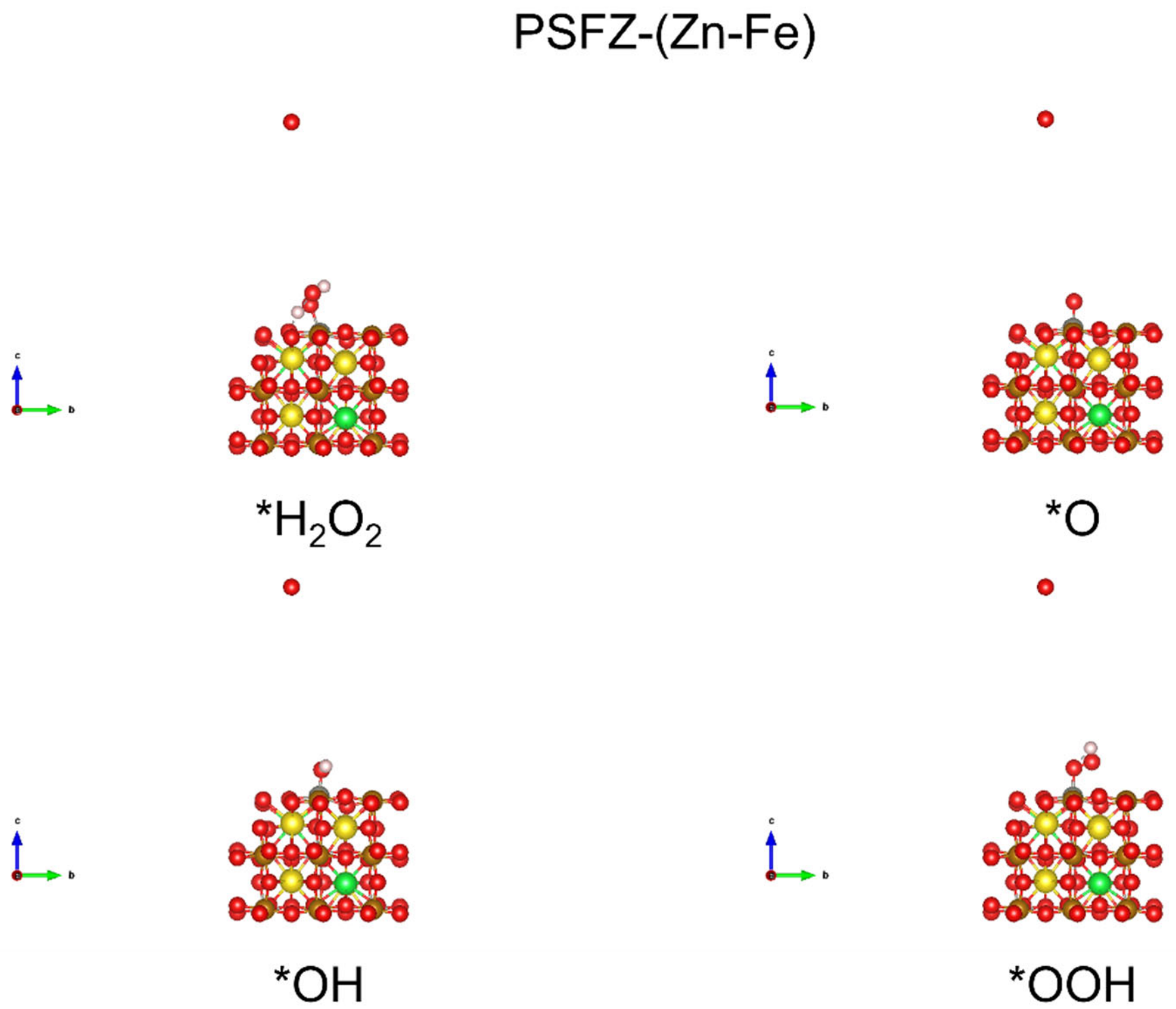

Supplementary Fig. 53 Side-view structure of the adsorbed intermediates *H_2O_2, *O, *OH, and *OOH on the PSFZ- (Zn-Fe) site. Colors represent: Pr (yellow), Sr (green), Fe (brown), Zn (grey), Mo (pink), O (red), H (white).

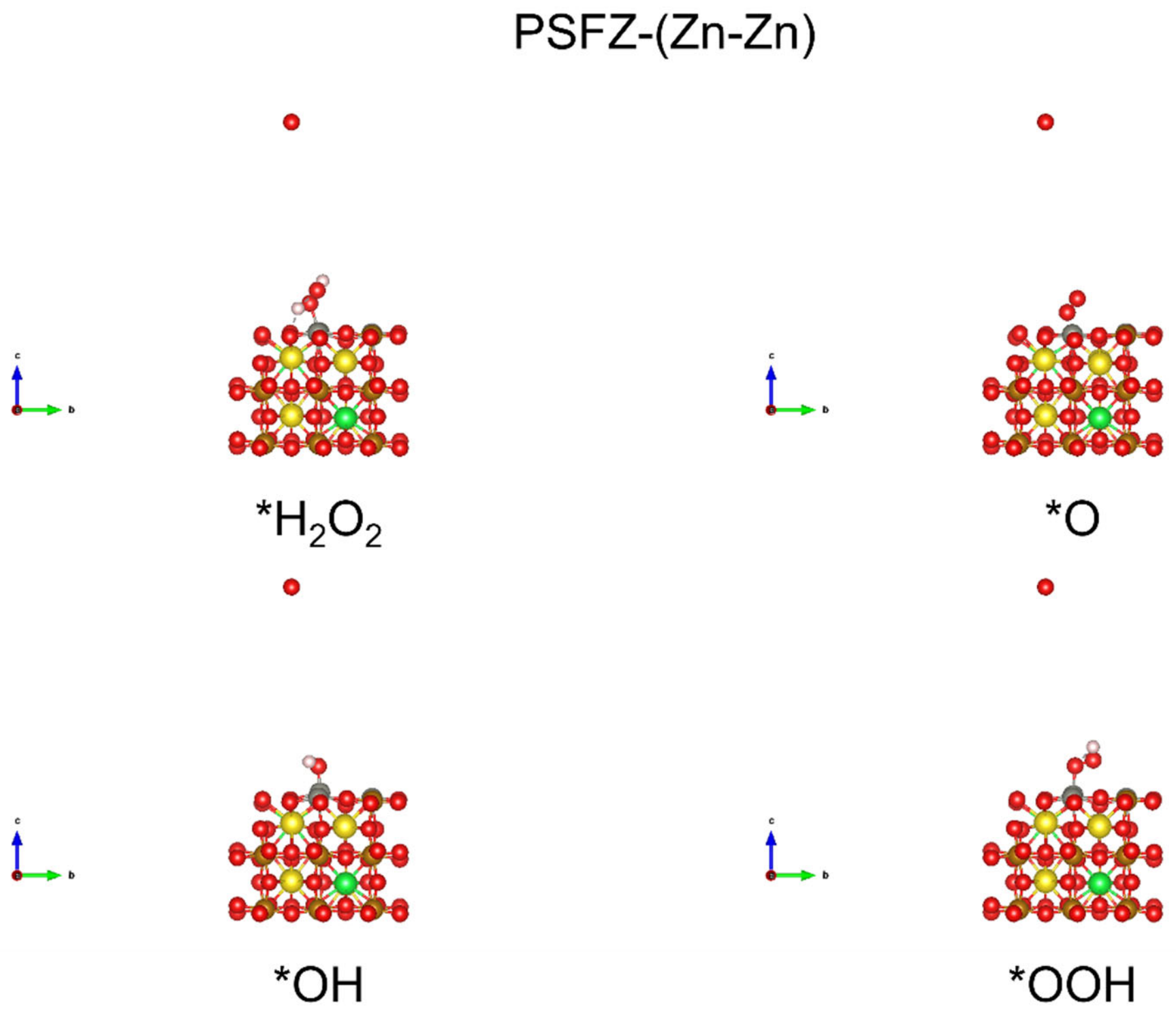

Supplementary Fig. 54 Side-view structure of the adsorbed intermediates *H_2O_2, *O, *OH, and *OOH on the PSFZ- (Zn-Zn) site. Colors represent: Pr (yellow), Sr (green), Fe (brown), Zn (grey), Mo (pink), O (red), H (white).

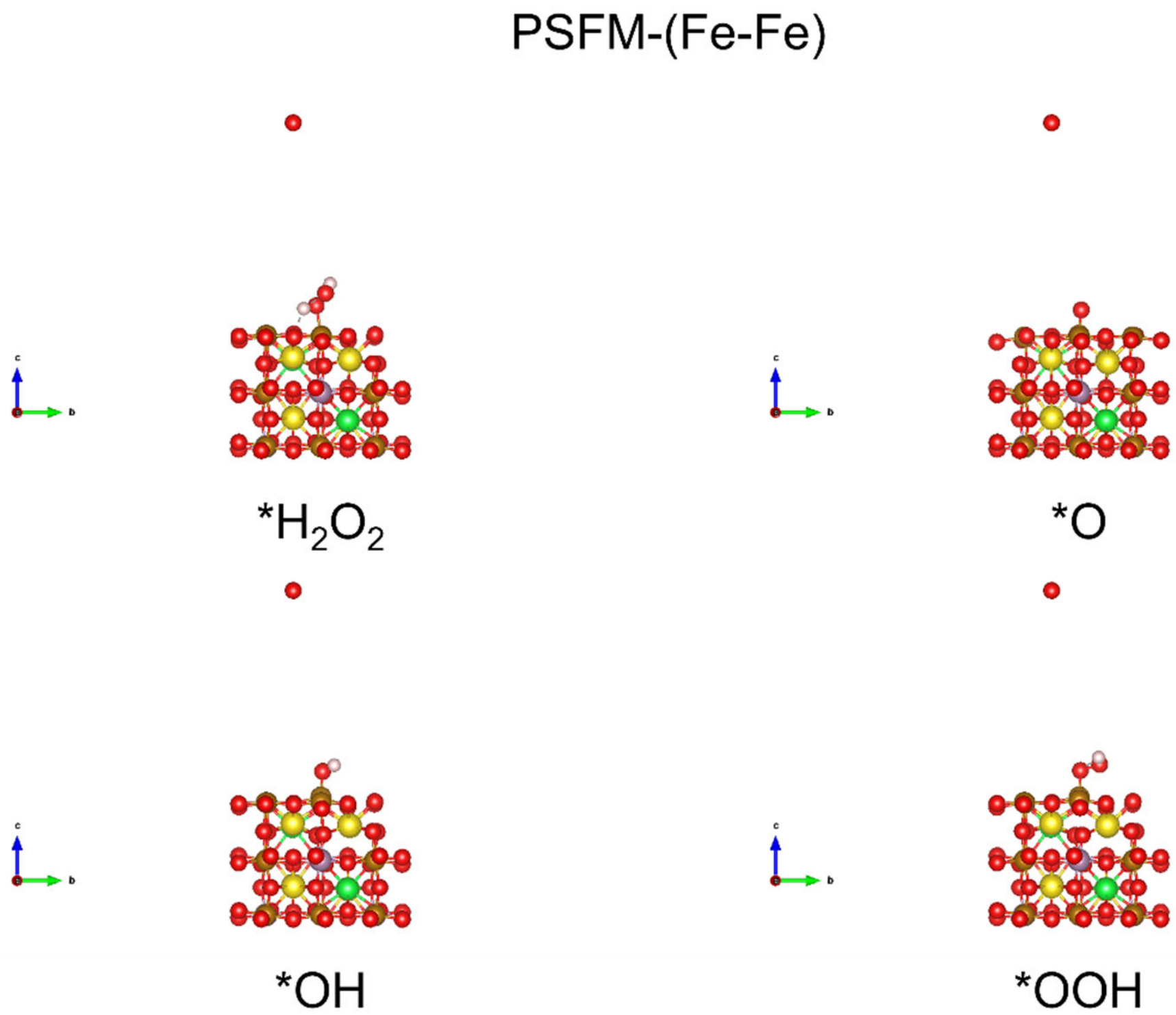

Supplementary Fig. 55 Side-view structure of the adsorbed intermediates *H_2O_2, *O, *OH, and *OOH on the PSFM- (Fe-Fe) site. Colors represent: Pr (yellow), Sr (green), Fe (brown), Zn (grey), Mo (pink), O (red), H (white).

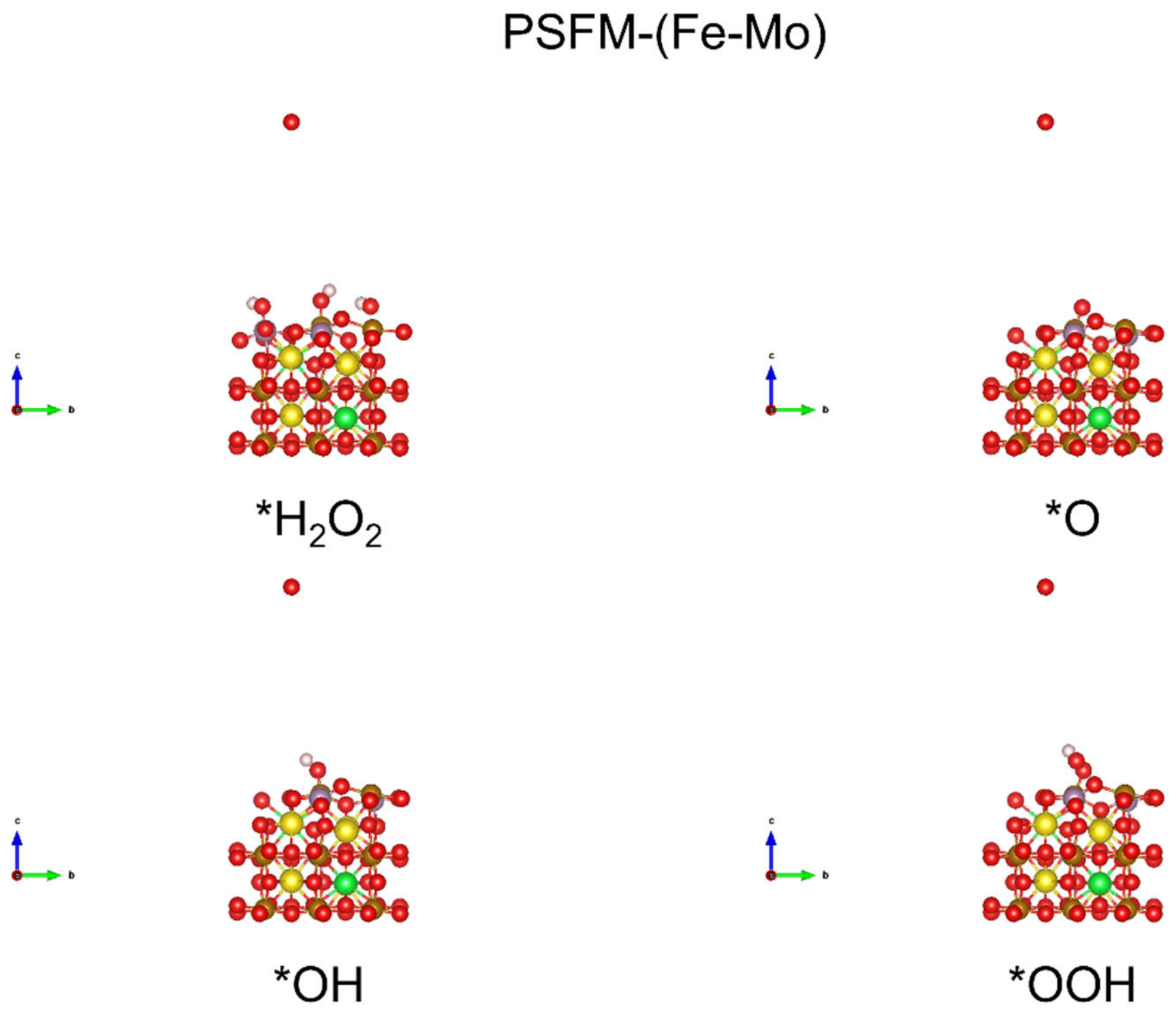

Supplementary Fig. 56 Side-view structure of the adsorbed intermediates *H_2O_2, *O, *OH, and *OOH on the PSFM- (Fe-Mo) site. Colors represent: Pr (yellow), Sr (green), Fe (brown), Zn (grey), Mo (pink), O (red), H (white).

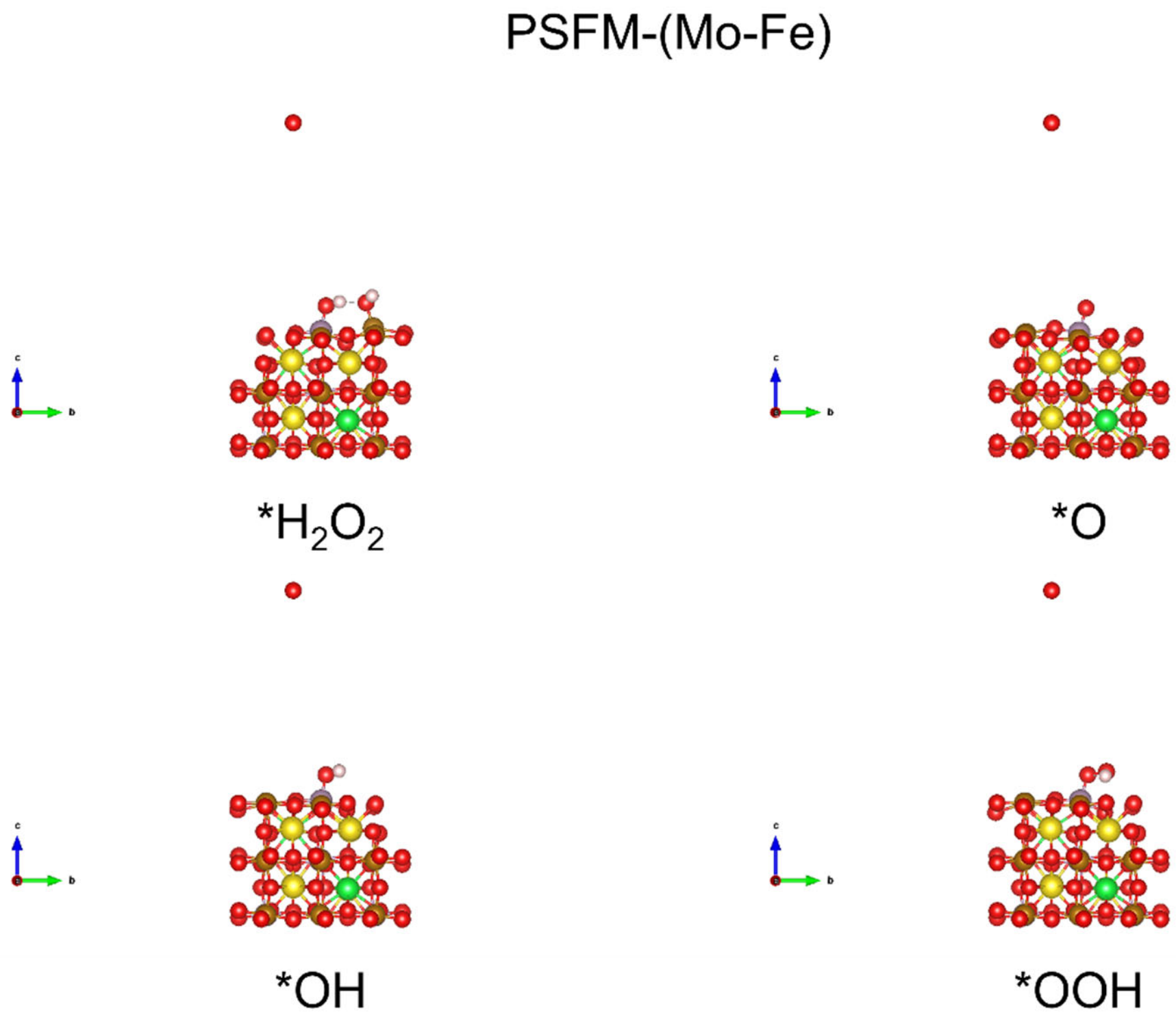

Supplementary Fig. 57 Side-view structure of the adsorbed intermediates *H_2O_2, *O, *OH, and *OOH on the PSFM- (Mo-Fe) site. Colors represent: Pr (yellow), Sr (green), Fe (brown), Zn (grey), Mo (pink), O (red), H (white).

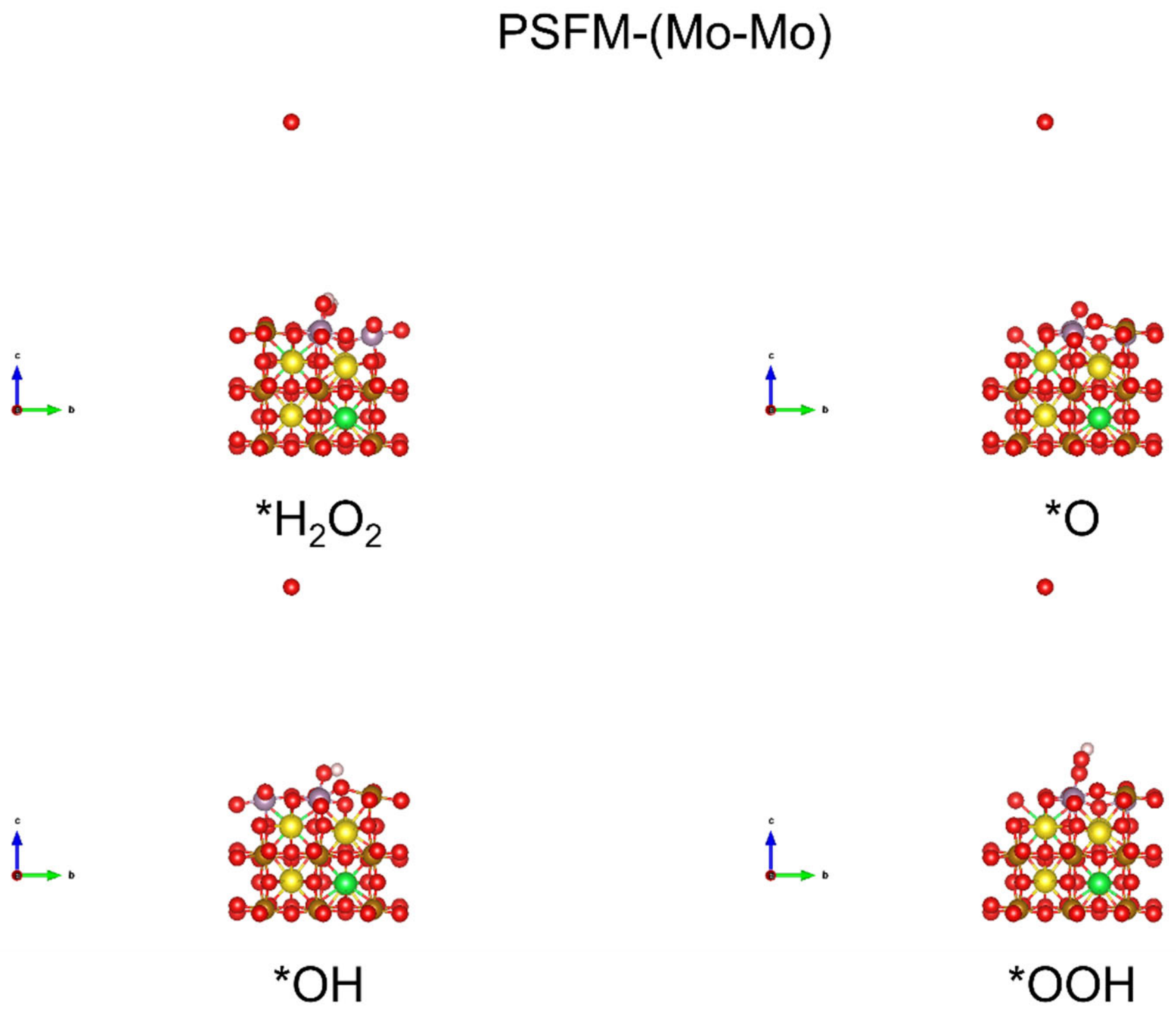

Supplementary Fig. 58 Side-view structure of the adsorbed intermediates *H_2O_2, *O, *OH, and *OOH on the PSFM- (Mo-Mo) site. Colors represent: Pr (yellow), Sr (green), Fe (brown), Zn (grey), Mo (pink), O (red), H (white).

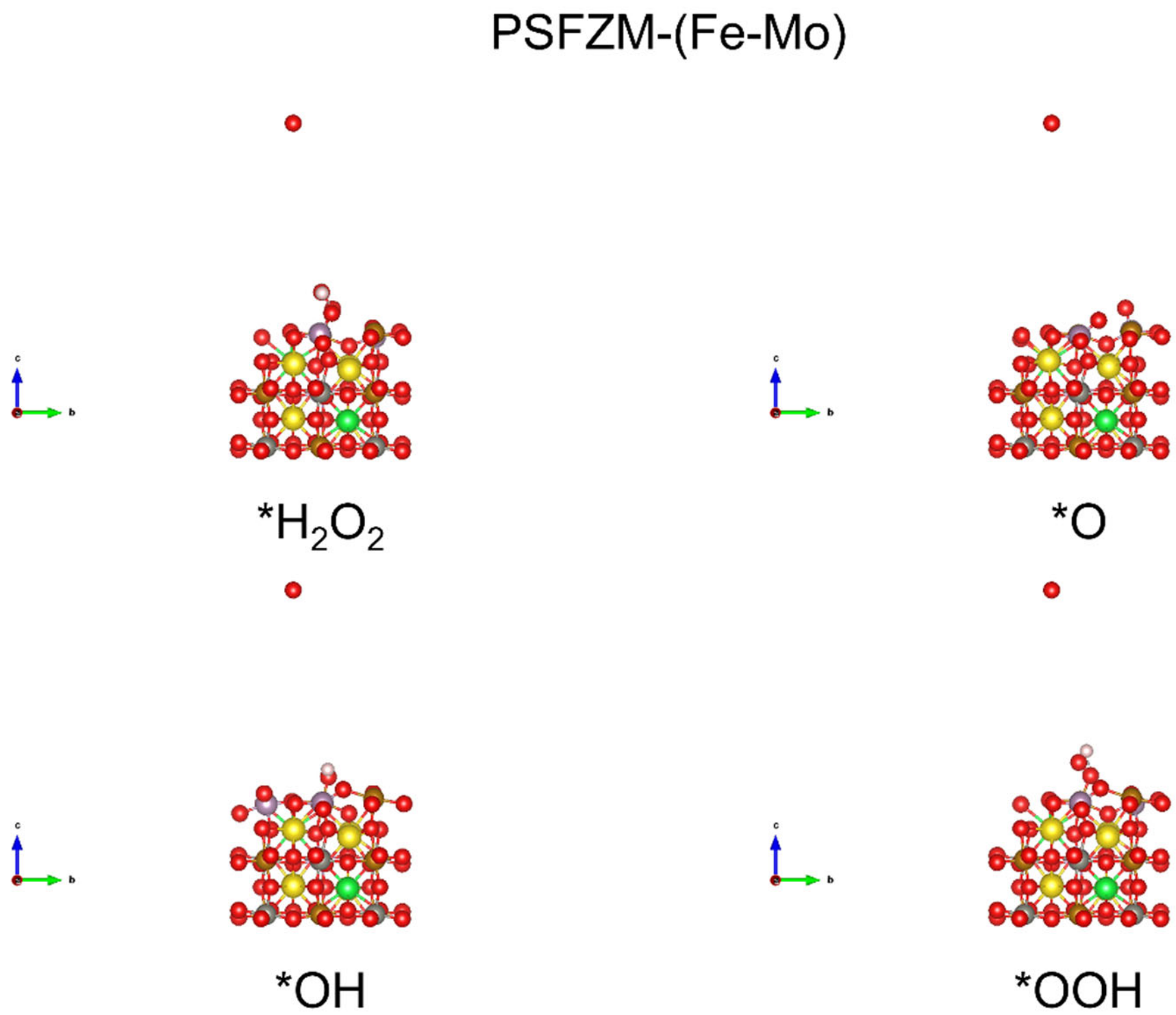

Supplementary Fig. 59 Side-view structure of the adsorbed intermediates *H_2O_2, *O, *OH, and *OOH on the PSFZM- (Fe-Mo) site. Colors represent: Pr (yellow), Sr (green), Fe (brown), Zn (grey), Mo (pink), O (red), H (white).

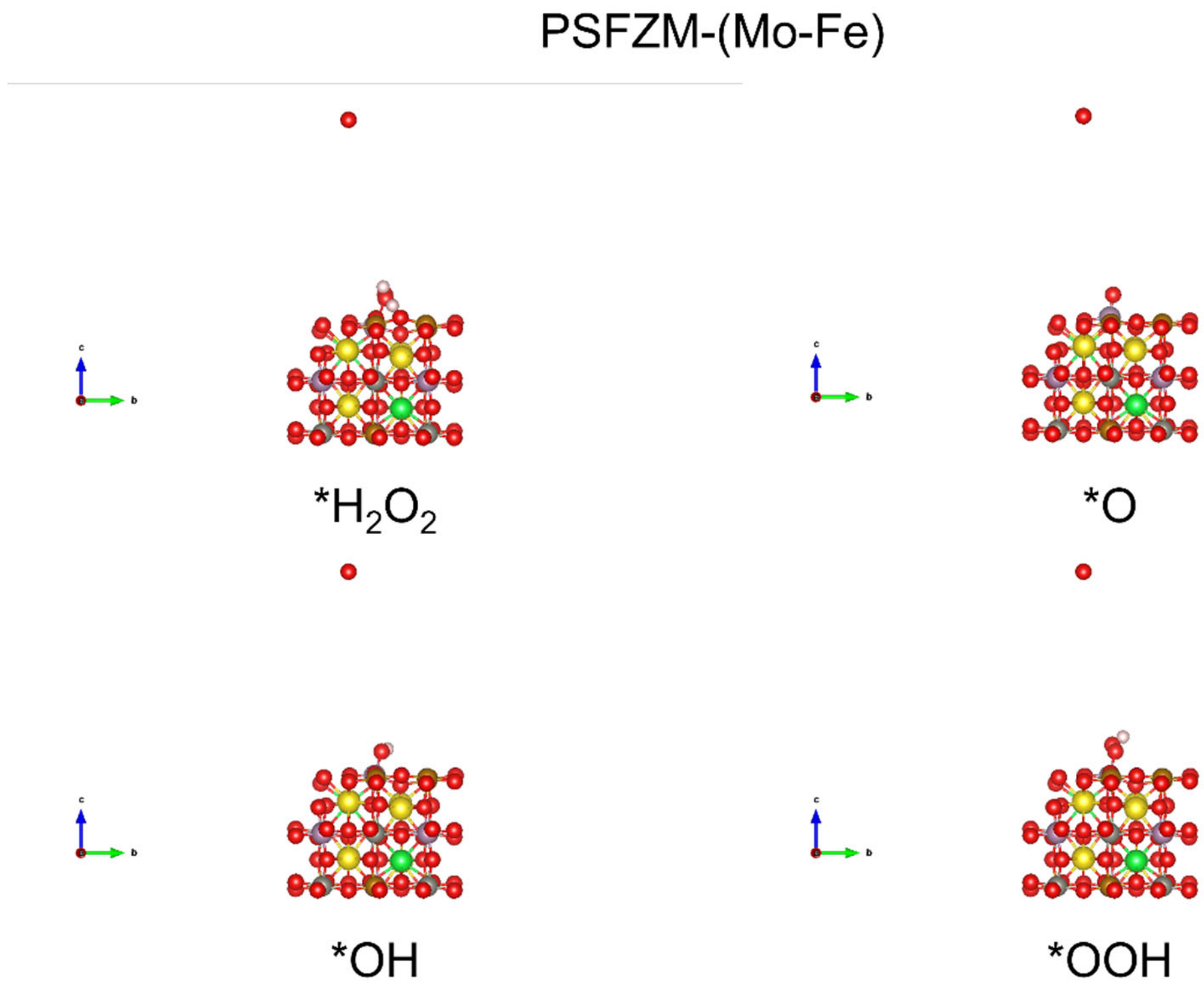

Supplementary Fig. 60 Side-view structure of the adsorbed intermediates *H_2O_2, *O, *OH, and *OOH on the PSFZM- (Mo-Fe) site. Colors represent: Pr (yellow), Sr (green), Fe (brown), Zn (grey), Mo (pink), O (red), H (white).

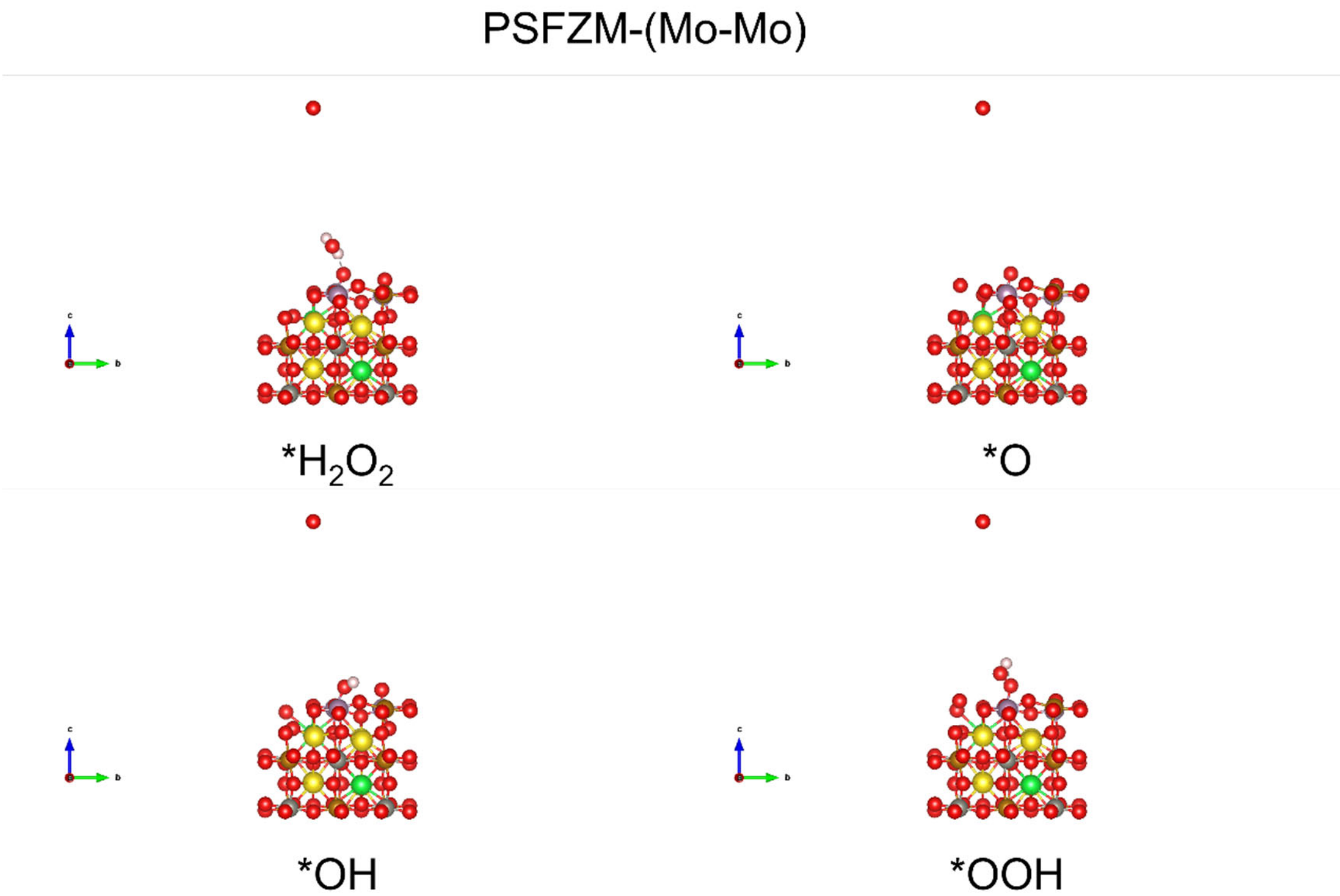

Supplementary Fig. 61 Side-view structure of the adsorbed intermediates *H_2O_2, *O, *OH, and *OOH on the PSFZM- (Mo-Mo) site. Colors represent: Pr (yellow), Sr (green), Fe (brown), Zn (grey), Mo (pink), O (red), H (white).

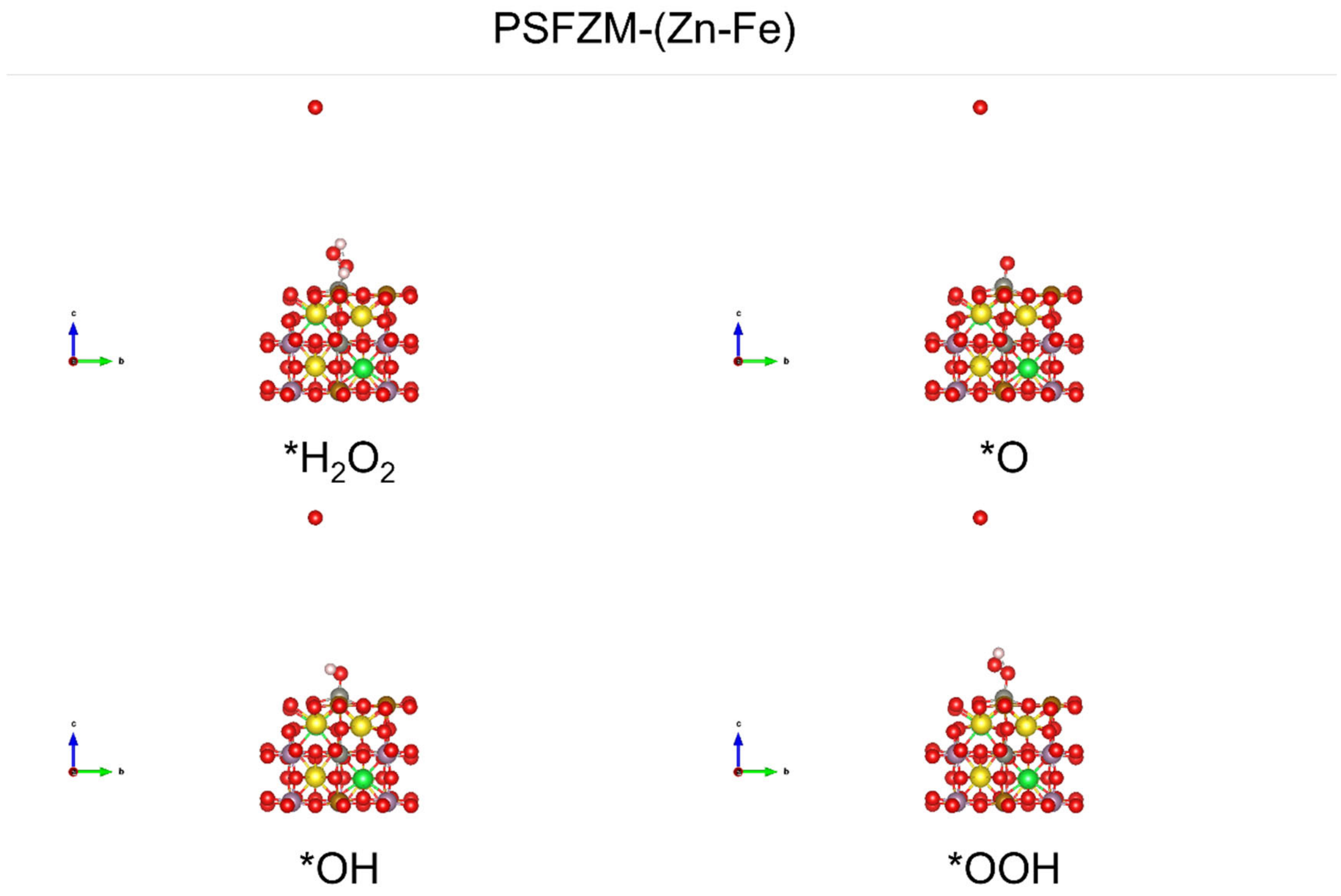

Supplementary Fig. 62 Side-view structure of the adsorbed intermediates *H_2O_2, *O, *OH, and *OOH on the PSFZM- (Zn-Fe) site. Colors represent: Pr (yellow), Sr (green), Fe (brown), Zn (grey), Mo (pink), O (red), H (white).

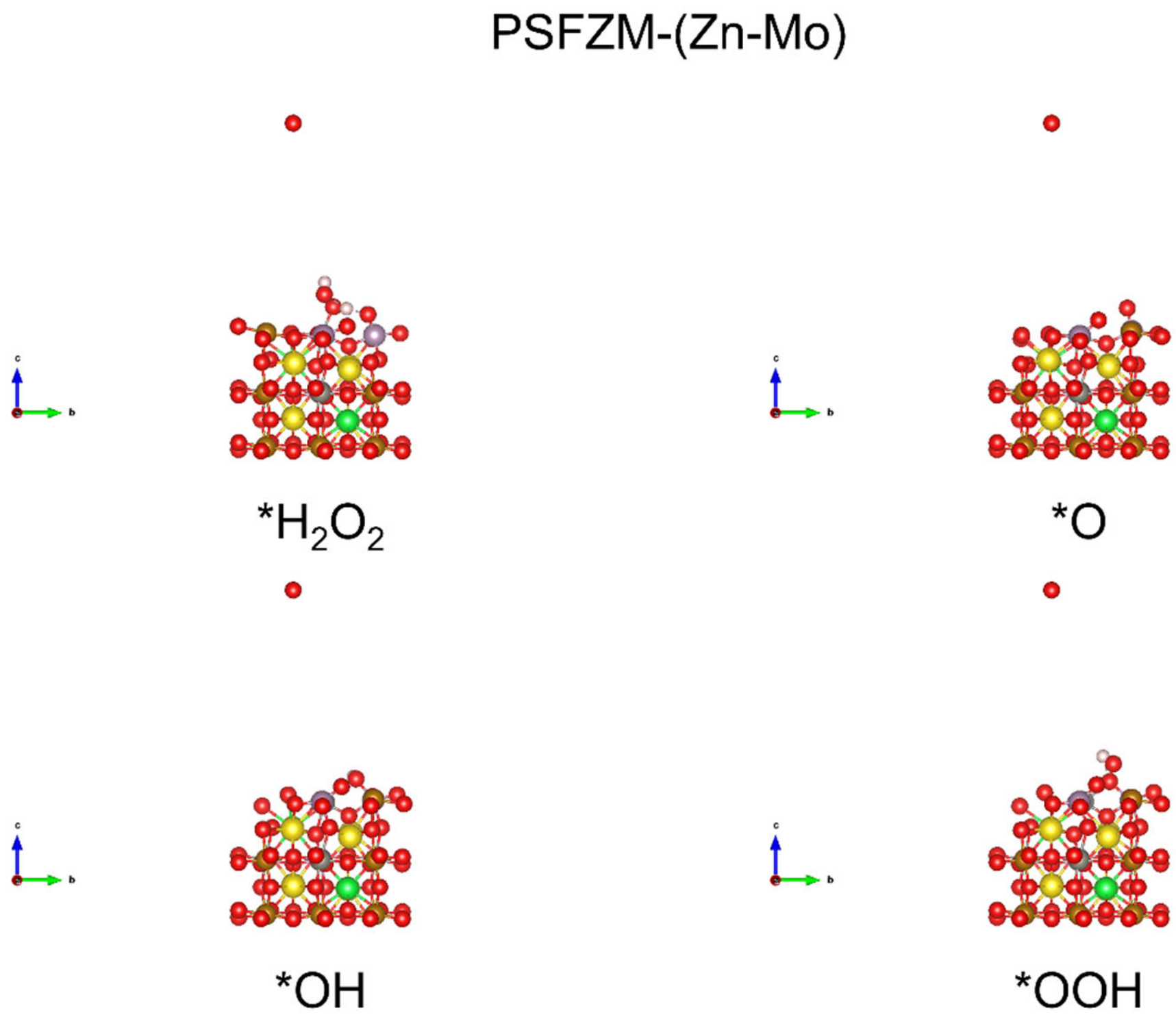

Supplementary Fig. 63 Side-view structure of the adsorbed intermediates *H_2O_2, *O, *OH, and *OOH on the PSFZM- (Zn-Mo) site. Colors represent: Pr (yellow), Sr (green), Fe (brown), Zn (grey), Mo (pink), O (red), H (white).

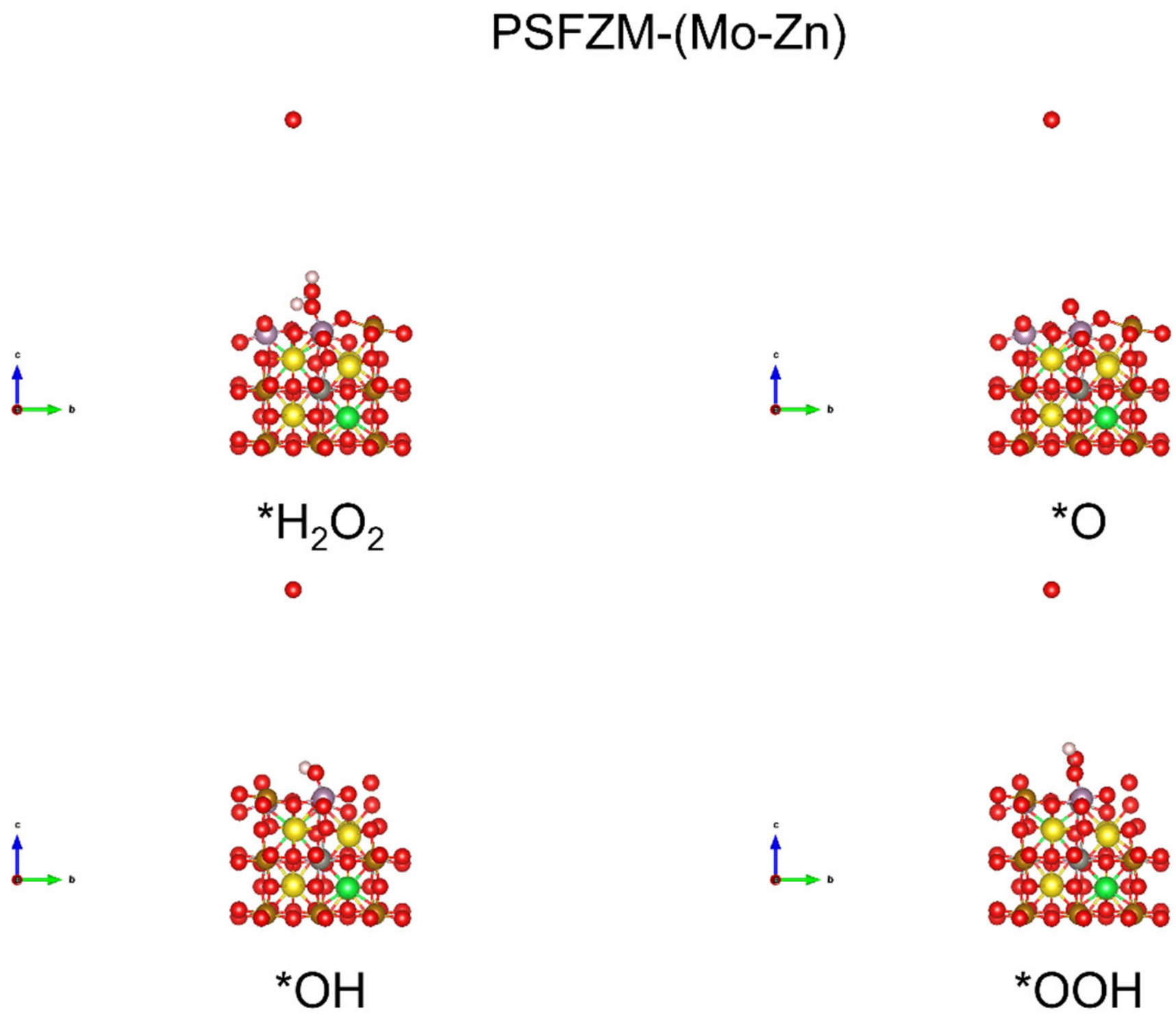

Supplementary Fig. 64 Side-view structure of the adsorbed intermediates *H_2O_2, *O, *OH, and *OOH on the PSFZM- (Mo-Zn) site. Colors represent: Pr (yellow), Sr (green), Fe (brown), Zn (grey), Mo (pink), O (red), H (white).

PSFZM-(Zn-Zn)

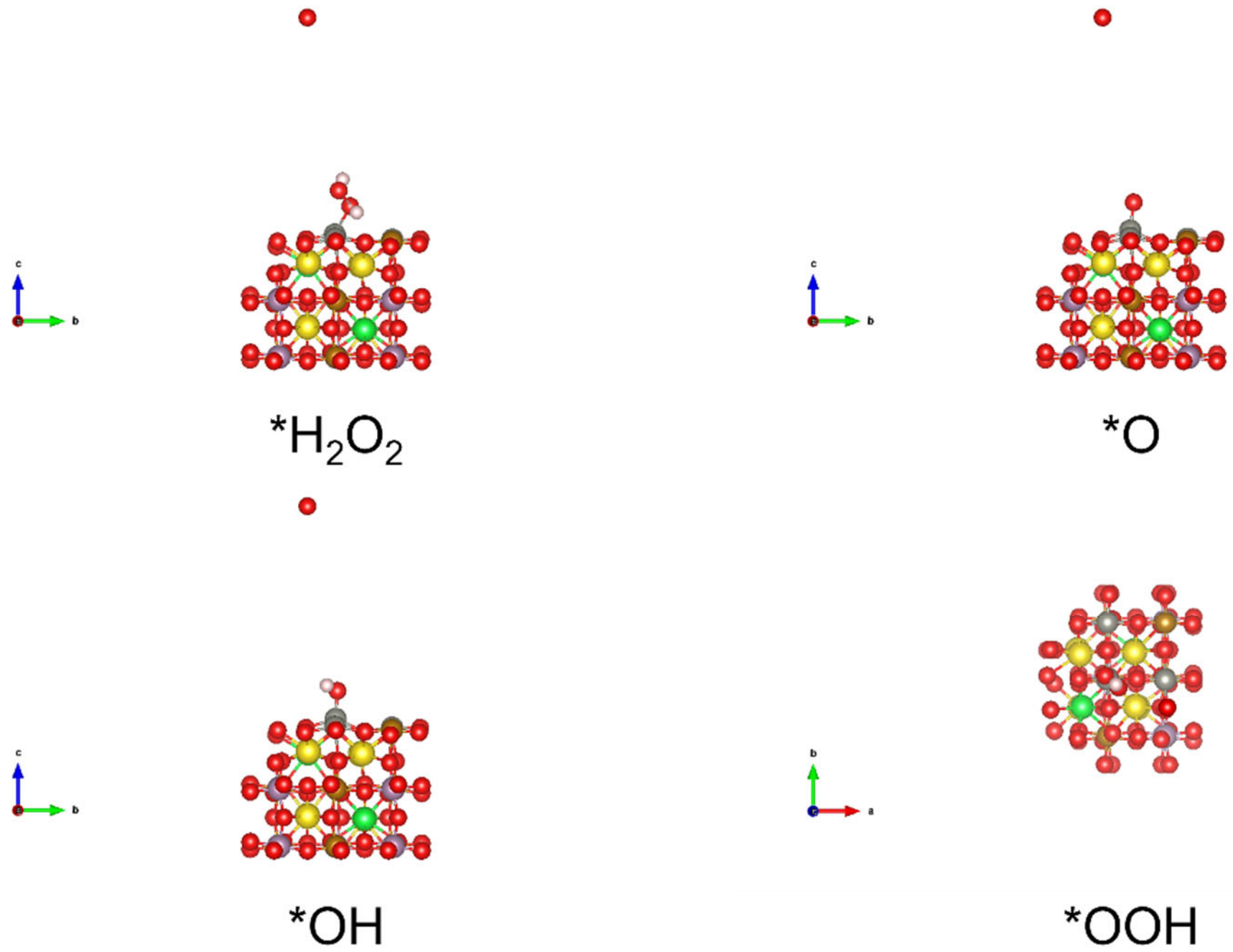

Supplementary Fig. 65 Side-view structure of the adsorbed intermediates *H_2O_2, *O, *OH, and *OOH on the PSFZM- (Zn-Zn) site. Colors represent: Pr (yellow), Sr (green), Fe (brown), Zn (grey), Mo (pink), O (red), H (white).

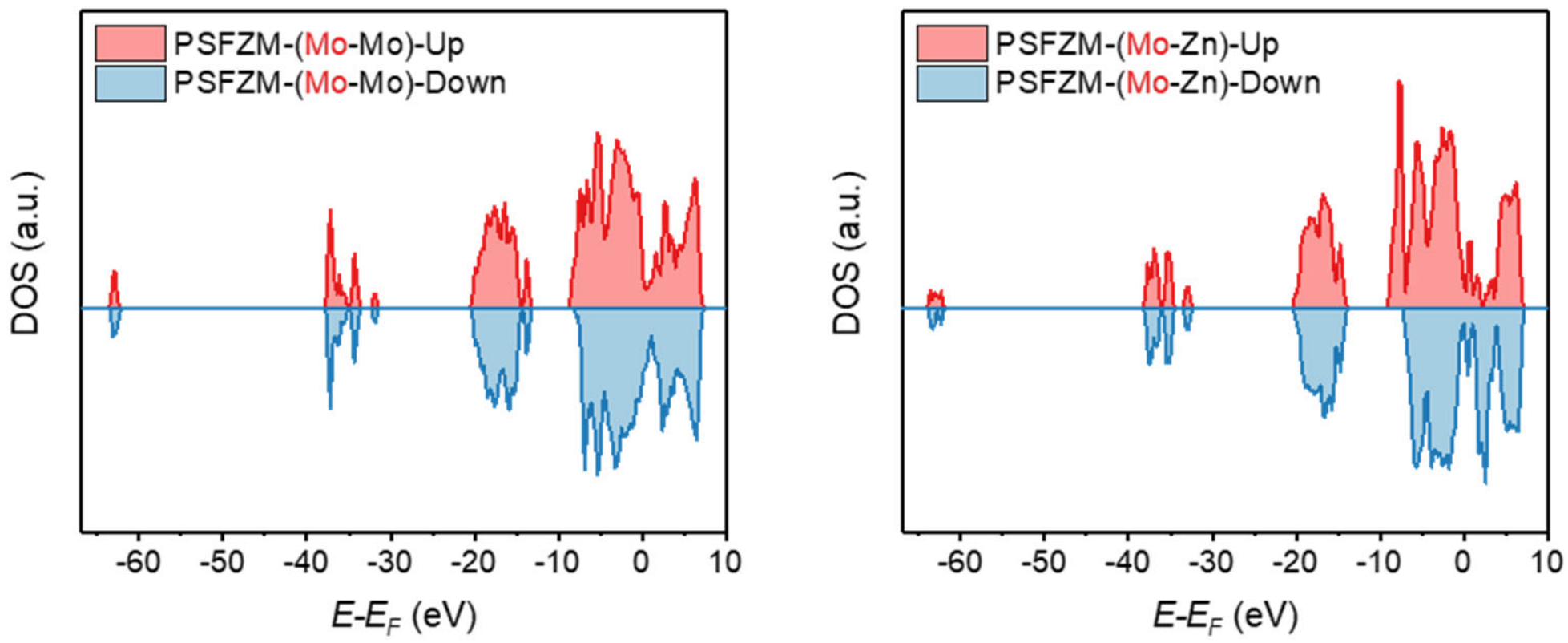

Supplementary Fig. 66 DOS analysis for PSFZM-(Mo-Mo) site and PSFZM-(Mo-Zn) site after H_2O_2 adsorption. Source data for Supplementary Fig. 66 are provided as a Source Data file.

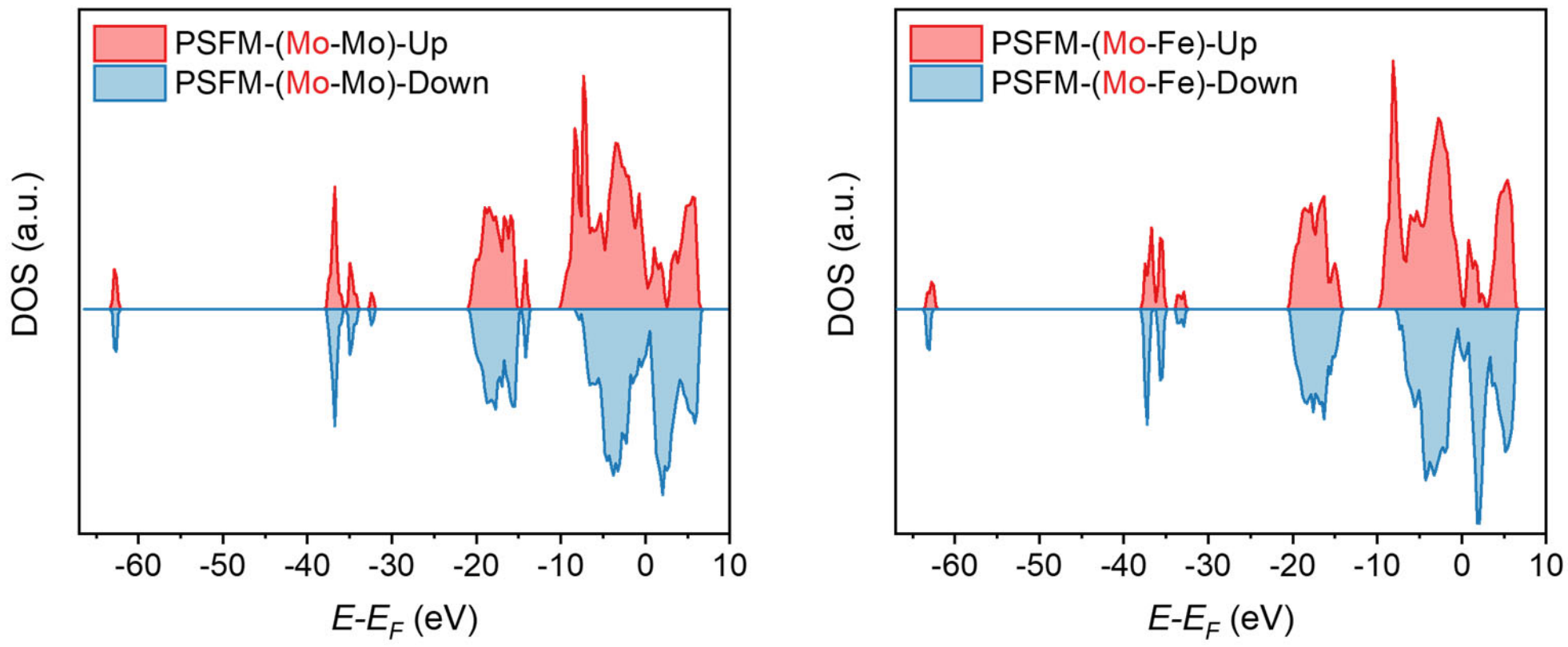

Supplementary Fig. 67 DOS analysis for PSFM-(Mo-Mo) and PSFM-(Mo-Fe) site after H_2O_2 adsorption. Source data for Supplementary Fig. 67 are provided as a Source Data file.

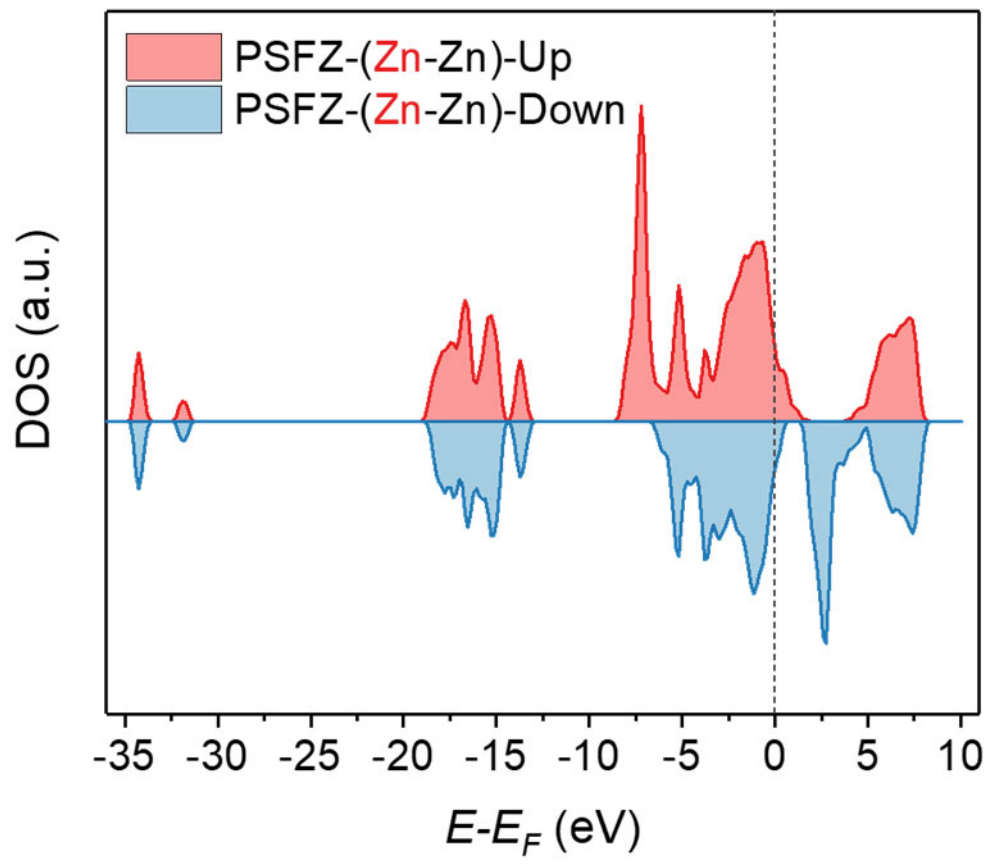

Supplementary Fig. 68 DOS analysis for PSFZ-(Zn-Zn) site after H_2O_2 adsorption. Source data for Supplementary Fig. 68 are provided as a Source Data file.

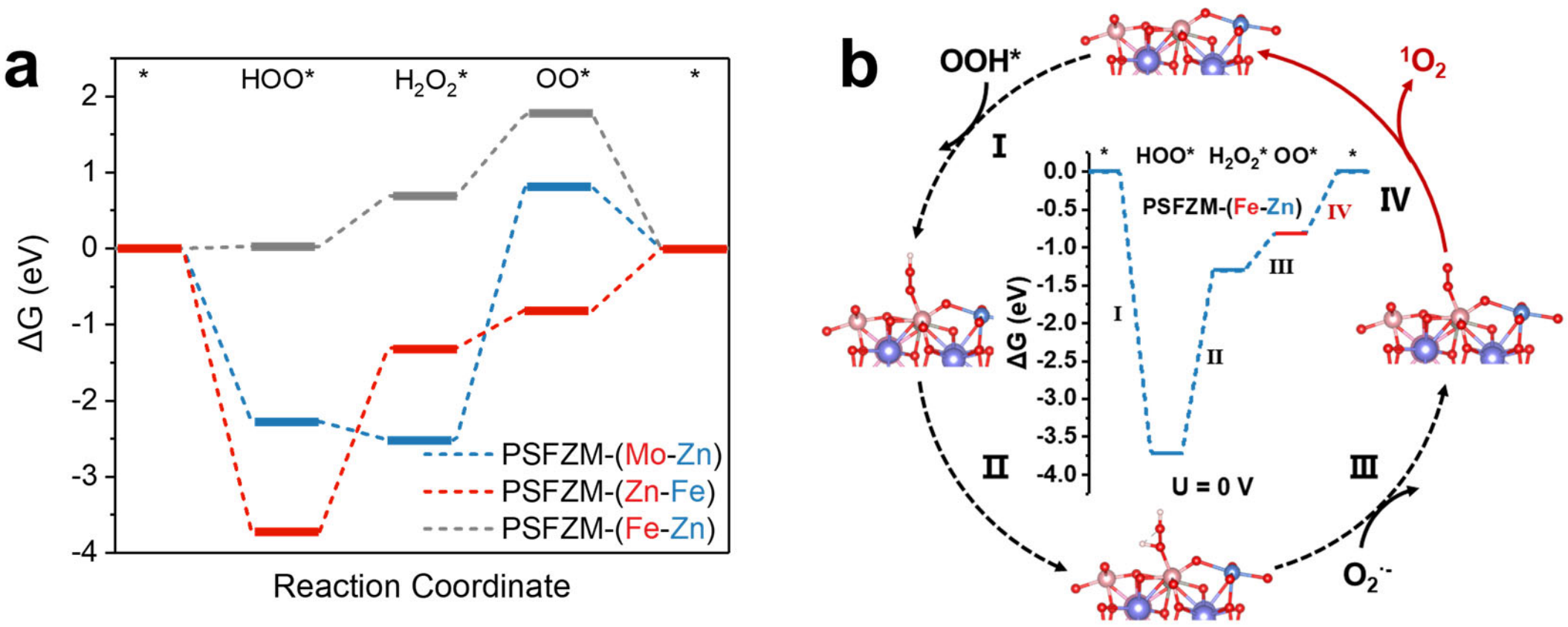

Supplementary Fig. 69 a Gibbs free energy of the whole reaction pathway for the generation of $^1O_2$ via three-electron ORR for all samples. **b** Intermediate structures of oxidation $*O_2$ to $^1O_2$ over PSFZM and the corresponding free energy diagrams. Colors represent: Pr (grey), Sr (yellow), Fe (purple), Zn (blue), Mo (pink), O (red) , H (white). Source data for Supplementary Fig. 69 are provided as a Source Data file.

Given the experimental detection of $^1O_2$ via ESR, we also explored its formation mechanism. DFT results indicate that the conversion of $*H_2O_2$ to *OO is the rate-determining step for $^1O_2$ generation. Notably, the Zn-Fe site exhibits the lowest ΔG for this step (**Supplementary Fig. 69a**), with Fe enhancing *OOH adsorption and destabilizing the O-H bond to promote *OO formation (**Supplementary Fig. 69b**). This underscores the complementary roles of bimetallic Zn centers: Zn-Mo for •OH and Zn-Fe for $^1O_2$, collectively boosting ROS diversity and O_2 utilization.

Thermodynamically downhill adsorption on Zn-Zn and Zn-Mo sites supports efficient intermediate stabilization and •OH formation. Conversely, $^1O_2$ generation occurs preferentially at Zn-Fe sites at Zn-Fe sites, as $^1O_2$ formation is thermodynamically unfavorable at Zn-Mo domains. These results reinforce the selective functionality of the Fe co-catalyst in facilitating alternative ROS pathways.

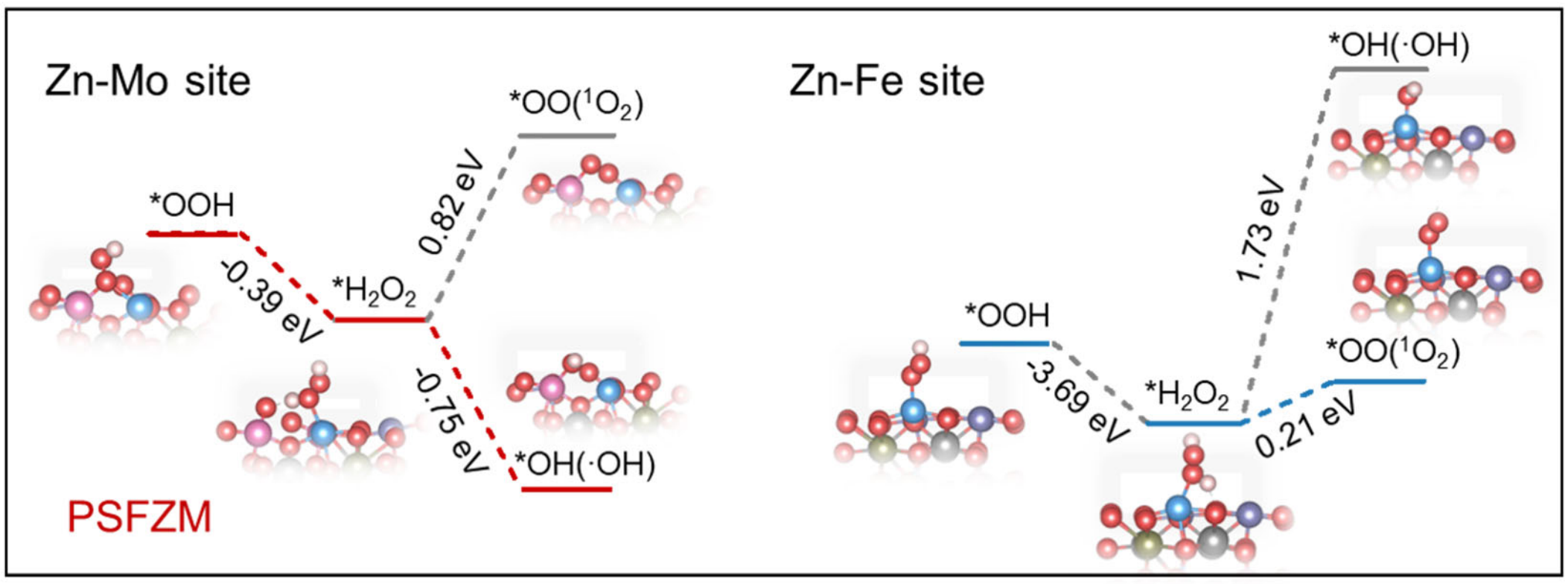

Supplementary Fig. 70 Comparison of free energies of adsorption of intermediate *OOH at all B sites in PSFZM. Comparison of the thermodynamics of $^1O_2$ and •OH generation at Zn-Mo and Zn-Fe sites in PSFZM. Colors represent: Pr (grey), Sr (yellow), Fe (purple), Zn (blue), Mo (pink), O (red) , H (white). Source data for Supplementary Fig. 70 are provided as a Source Data file.

Additionally, Mo incorporation upshifts the Zn d-band center (from -5.693 eV in PSF to -3.028 eV in PSFZM), thereby balancing *OO binding and accelerating ORR kinetics. Based on these results, two synergistic ROS formation pathways are proposed (**Supplementary Fig. 58**):

Pathway I (Zn-Mo site):

$$* + O_2 \rightarrow *O_2 \quad (6)$$

$$*O_2 + (H^+ + e^-) \rightarrow *OOH \quad (7)$$

$$*OOH + (H^+ + e^-) \rightarrow *H_2O_2 \quad (8)$$

$$*H_2O_2 + e^- \rightarrow \bullet OH + OH^- \quad (9)$$

Pathway II (Zn-Fe site):

$$*OOH + \bullet OH \rightarrow {}^1O_2 + H_2O \quad (10)$$

$$*OOH + H_2O_2 \rightarrow \bullet OH + {}^1O_2 + H_2O \quad (11)$$

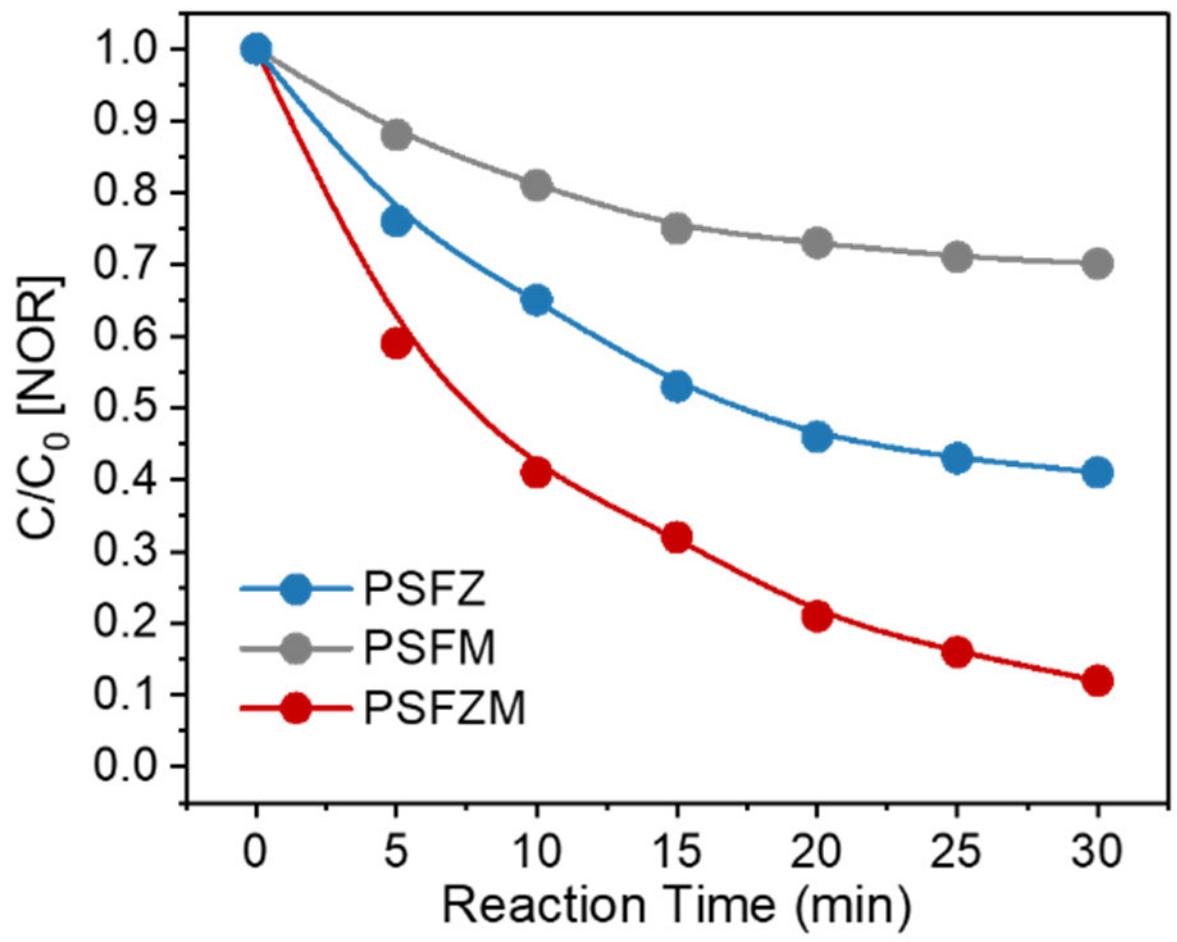

Supplementary Fig. 71 Degradation efficiency of NOR in different reaction systems. Source data for Supplementary Fig. 71 are provided as a Source Data file.

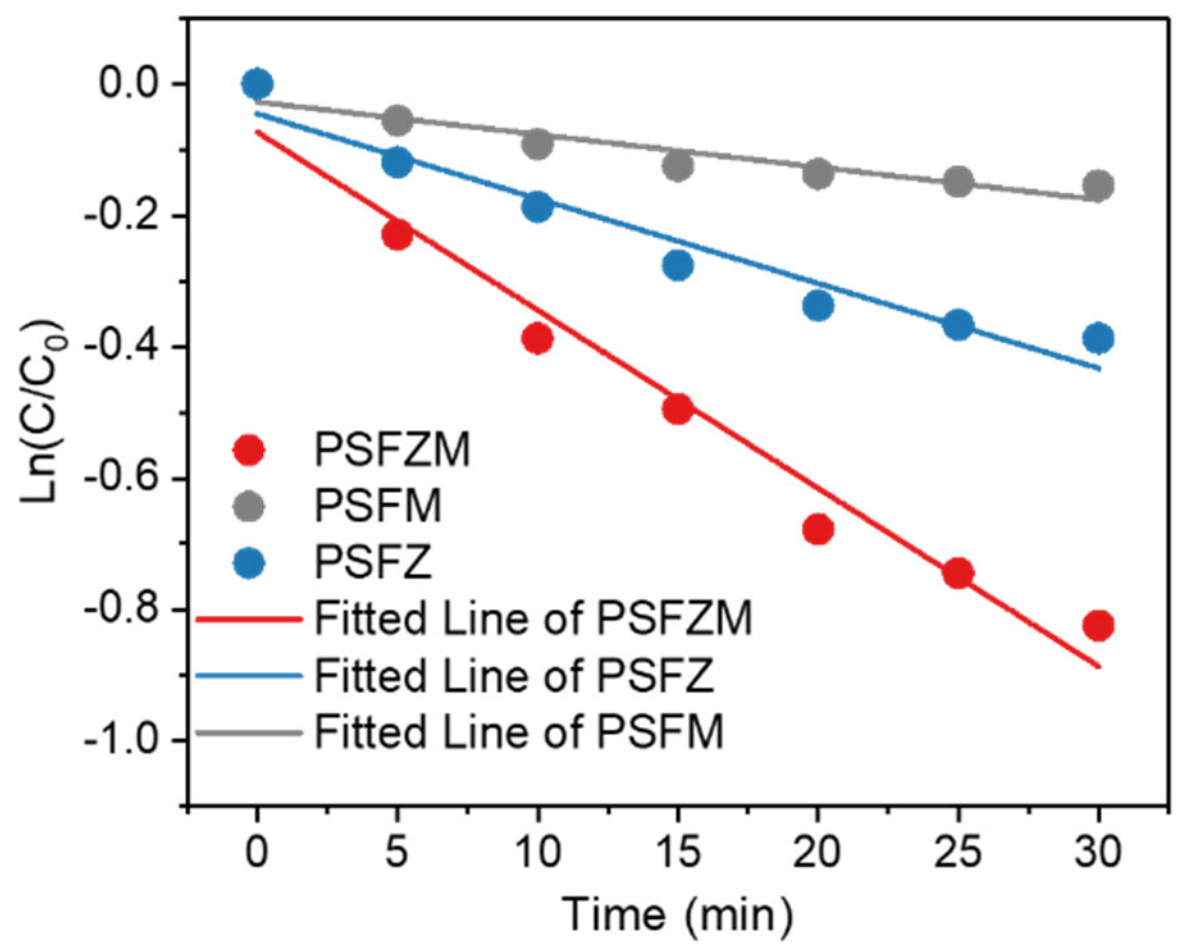

Supplementary Fig. 72 Corresponding first-order kinetics of different catalyst in the degradation system. Source data for Supplementary Fig. 72 are provided as a Source Data file.

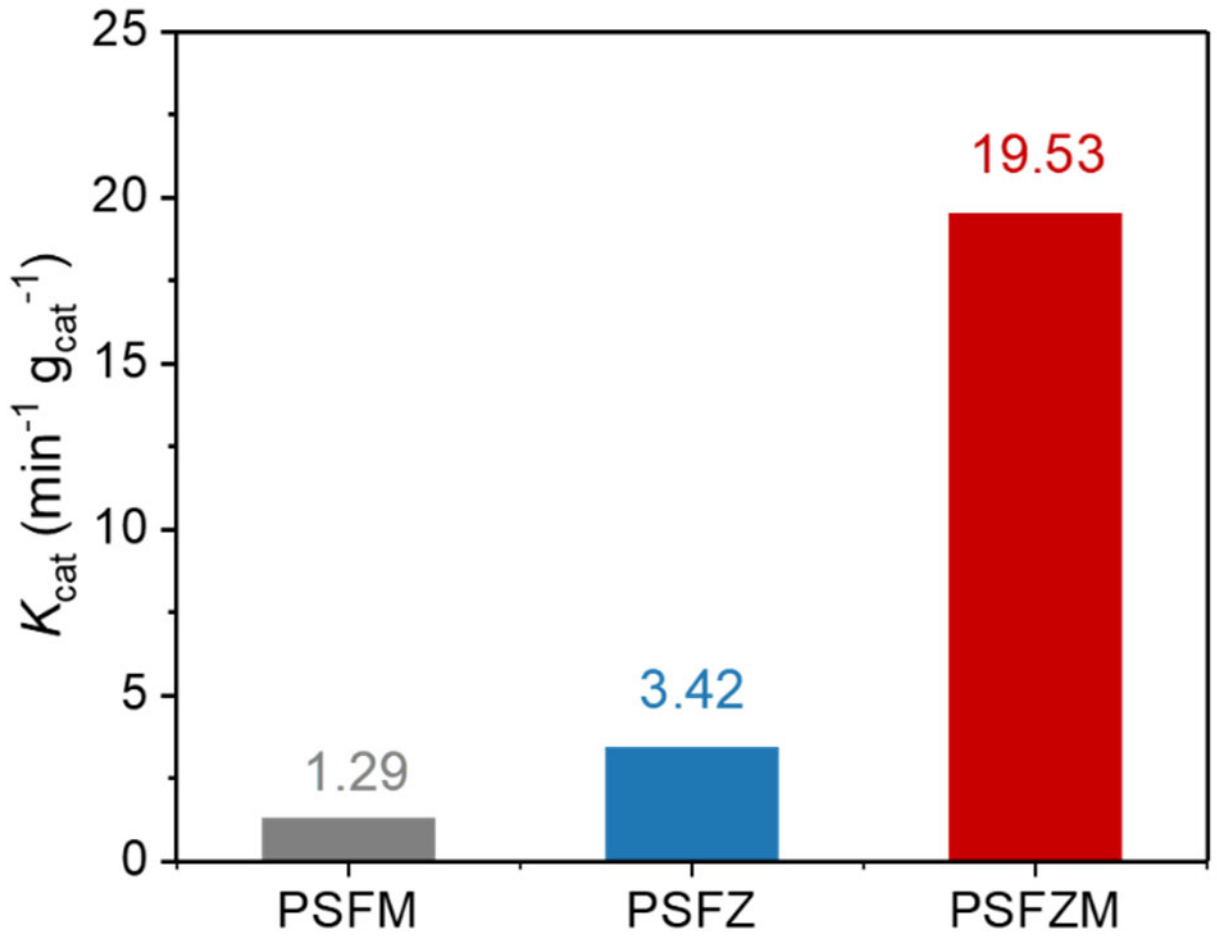

Supplementary Fig. 73 Degradation kinetics of NOR in different reaction systems. Source data for Supplementary Fig. 73 are provided as a Source Data file.

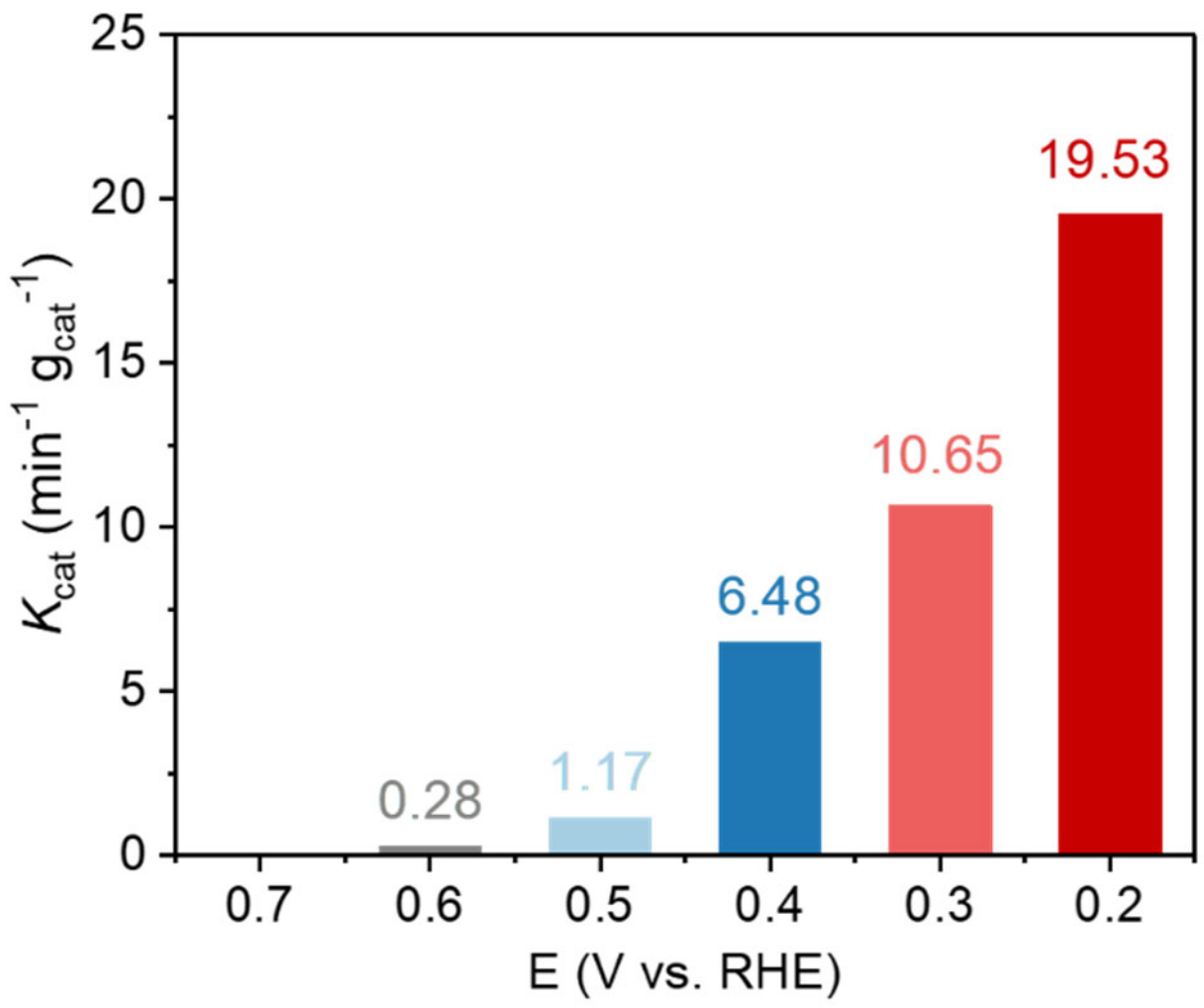

Supplementary Fig. 74 Kinetics of NOR degradation by PSFZM electrode at different voltages. Source data for Supplementary Fig. 74 are provided as a Source Data file.

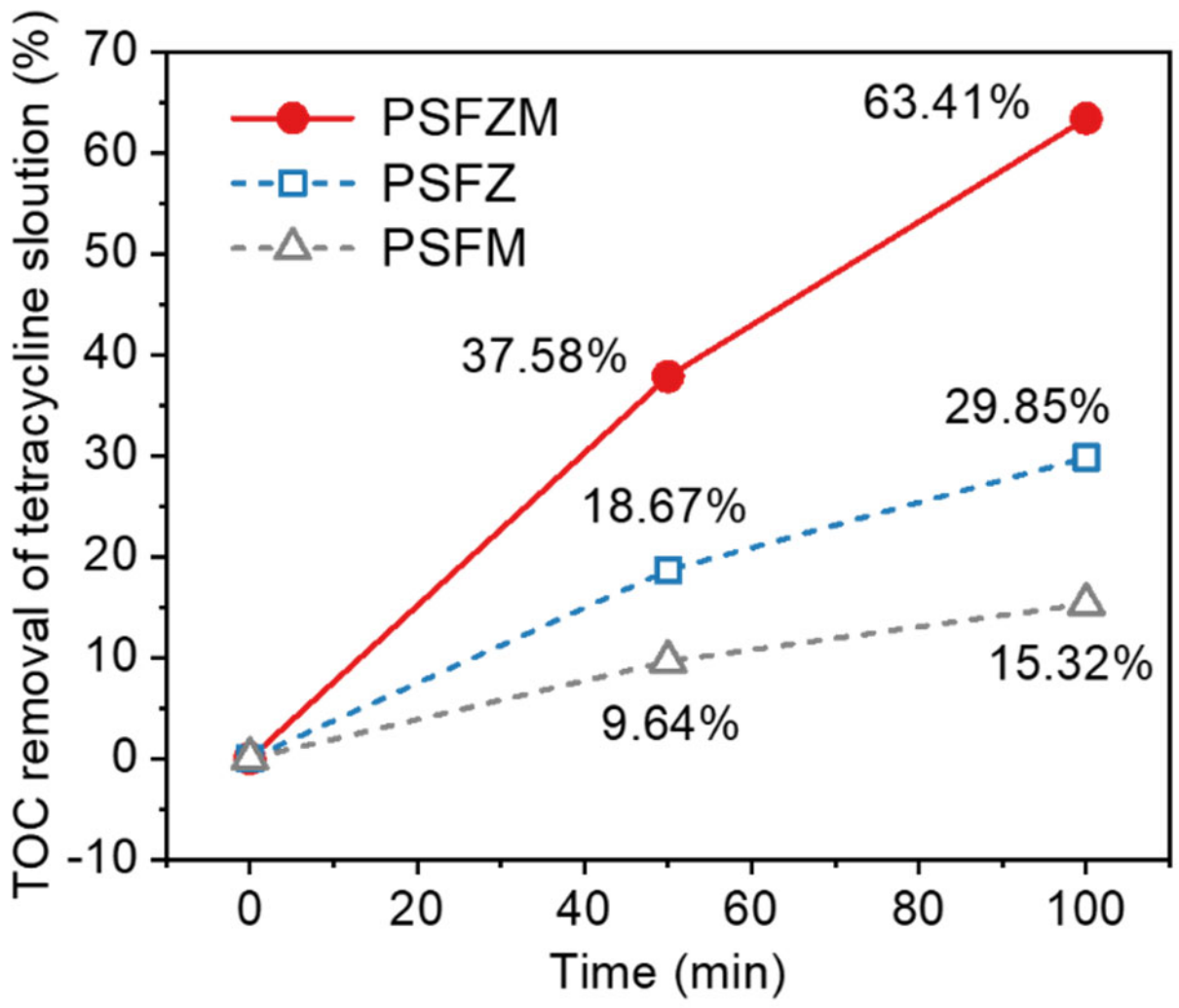

Supplementary Fig. 75 Removal rate of TOC during NOR degradation by PSFZM, PSFZ and PSFM perovskite catalyst (after 100 min). Source data for Supplementary Fig. 75 are provided as a Source Data file.

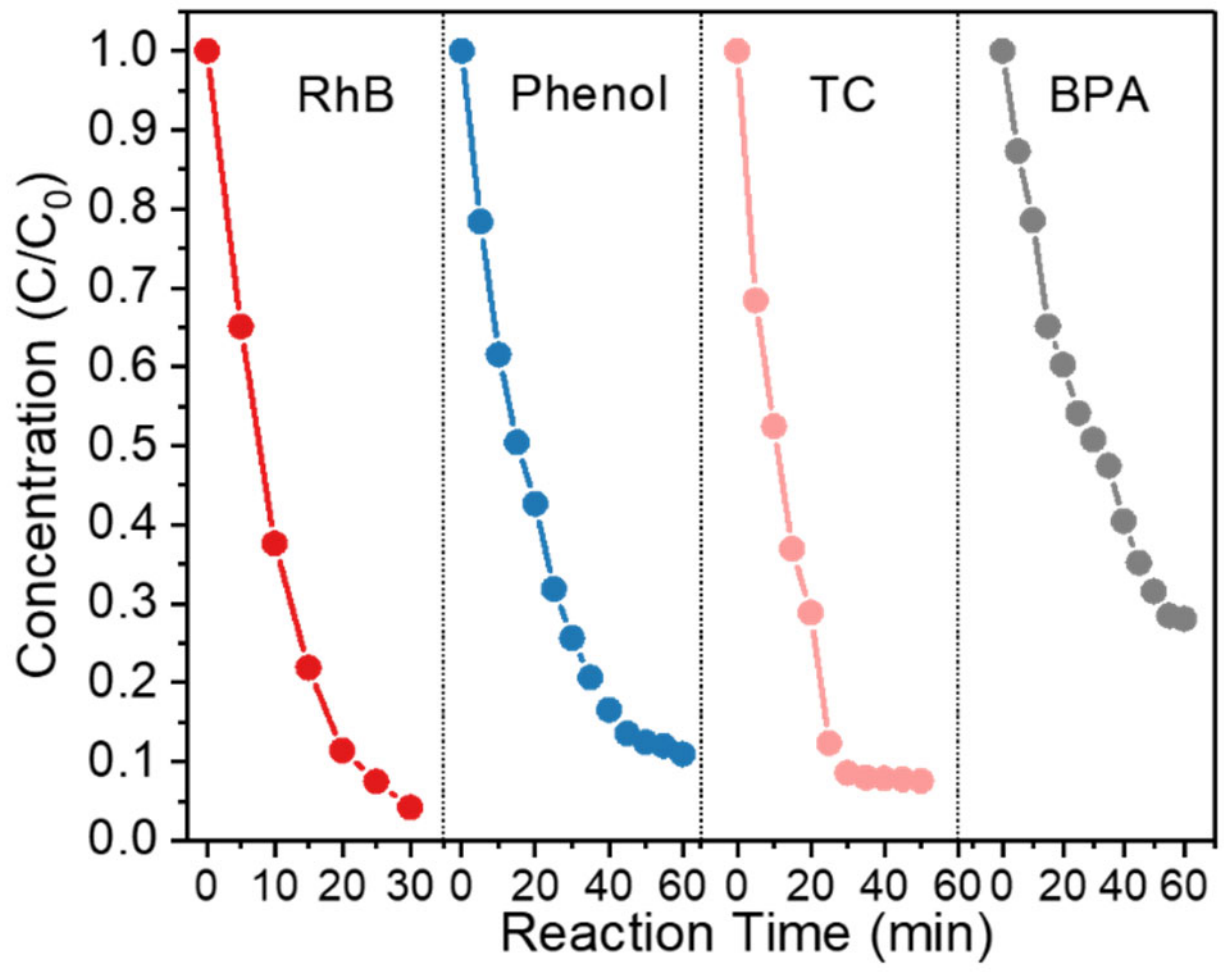

Supplementary Fig. 76 Degradation rate of PSFZM electrode in the presence of different pollutants. Source data for Supplementary Fig. 76 are provided as a Source Data file.

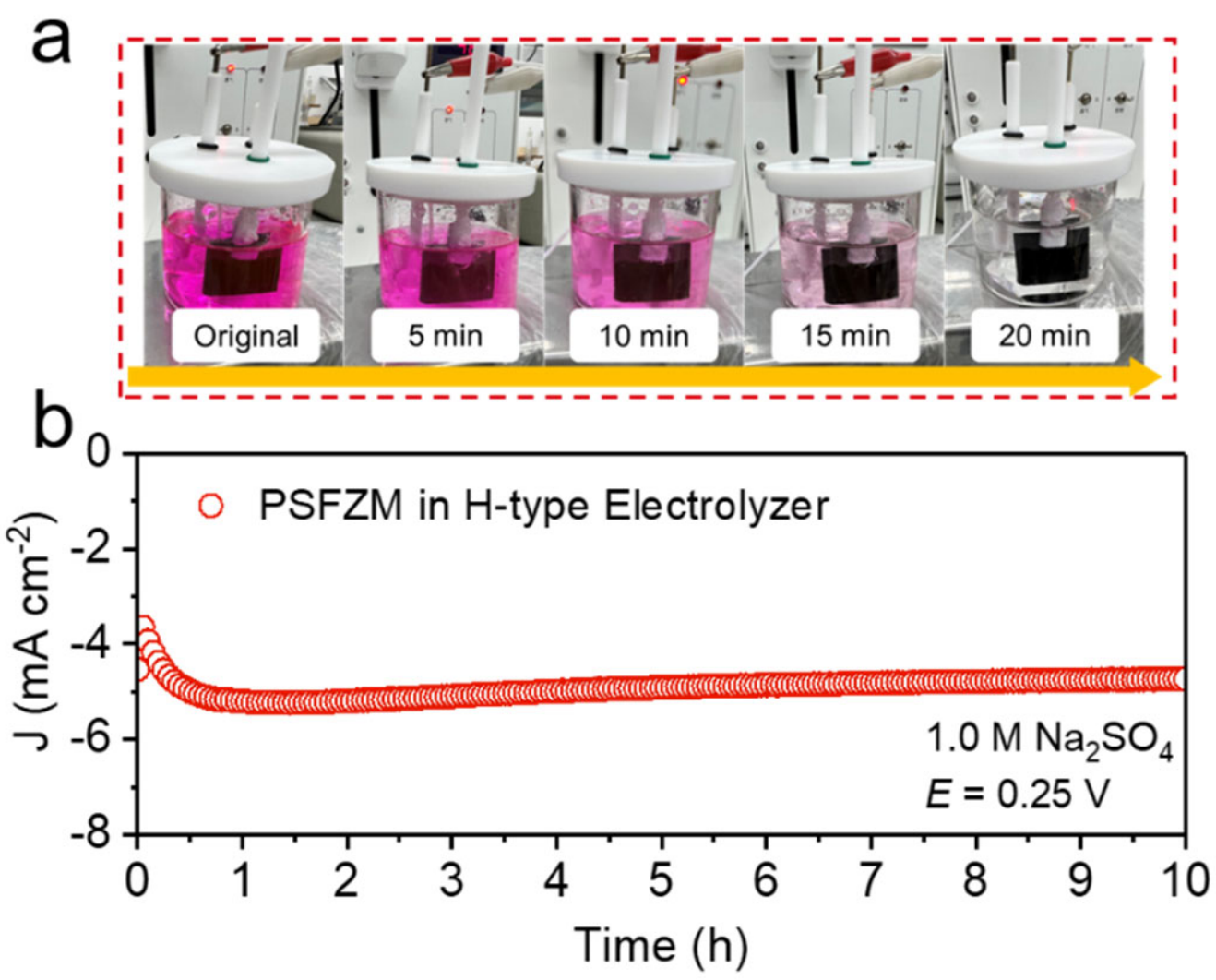

Supplementary Fig. 77 a The PSFZM electrode was used to remove RhB at a voltage of 0.25 V (vs. RHE) and the color change image was recorded within 20 min, and **b** the voltage was maintained for 10 h. Source data for Supplementary Fig. 77 are provided as a Source Data file.

The long-term stability of PSFZM was verified in a two-electrode H-type electrolyzer. After 10 hours of continuous operation, the current density decreased by only 4.6% (**Supplementary Fig. 77**), and the NOR degradation efficiency remained above 85.0% across 20 consecutive cycles (**Supplementary Fig. 77**), indicating robust structural stability and sustained ROS generation capability.

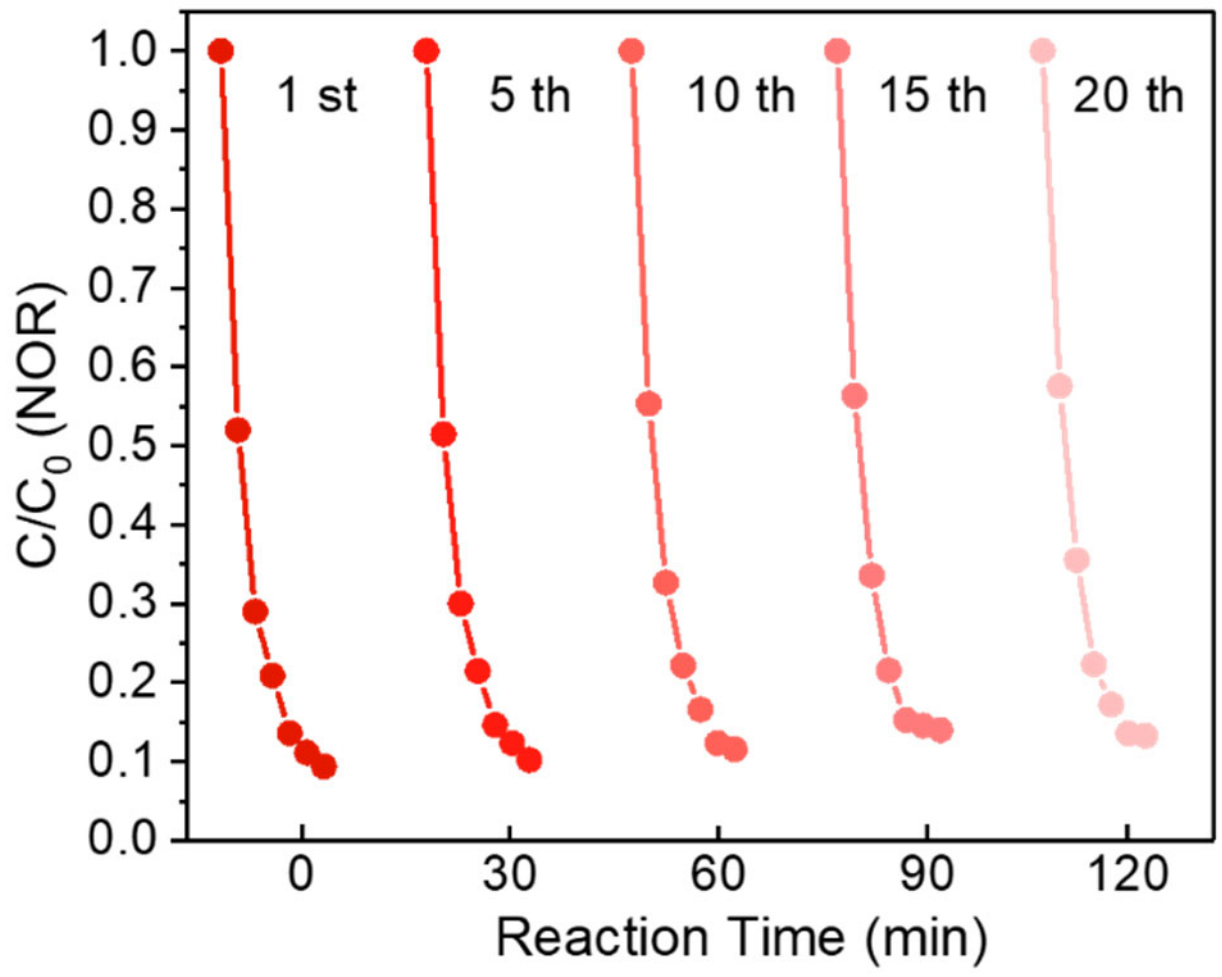

Supplementary Fig. 78 Stability tests of PSFZM samples for treating NOR in solution. Source data for Supplementary Fig. 78 are provided as a Source Data file.

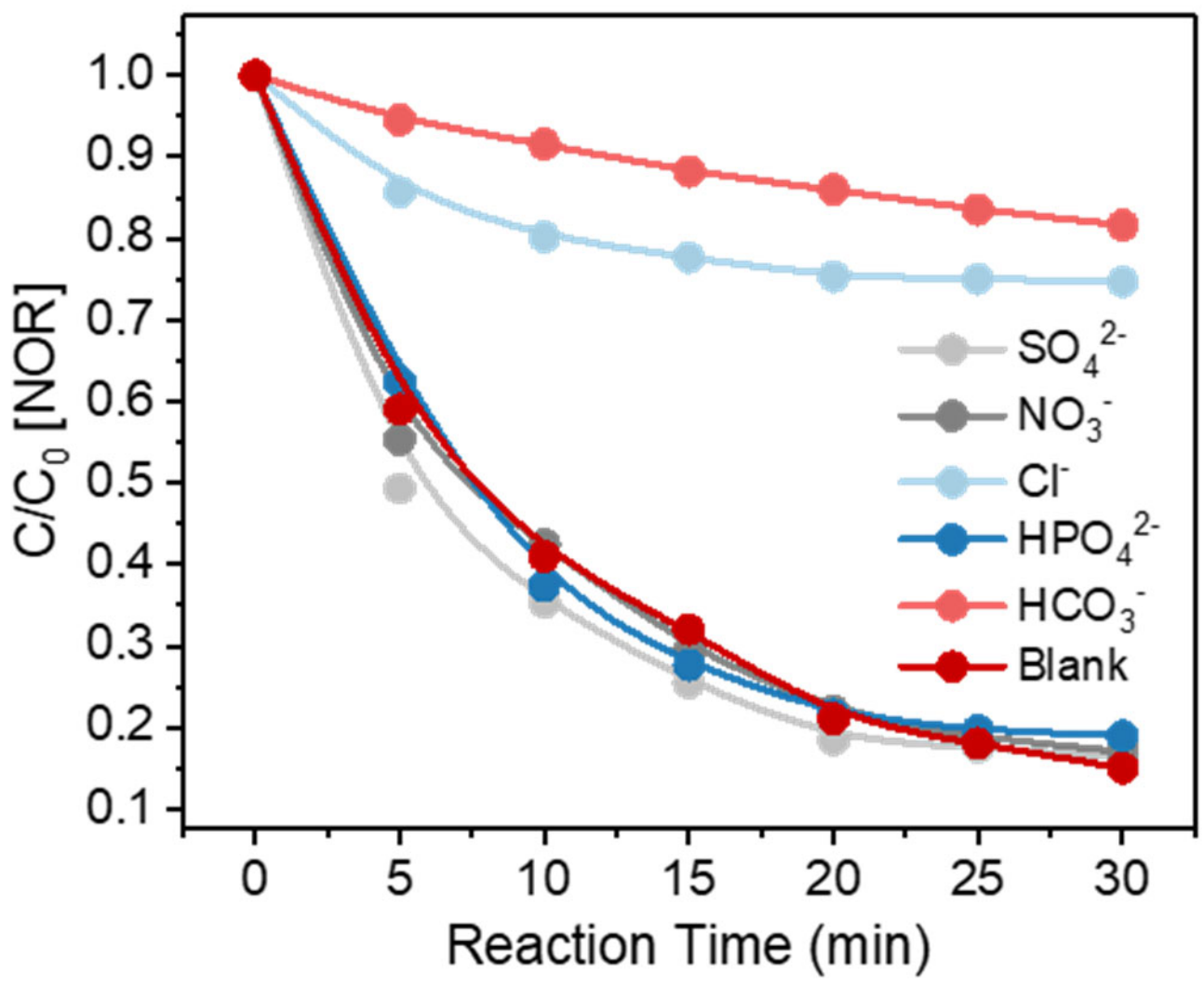

Supplementary Fig. 79 Degradation curves of NOR by PSFZM electrode under different anion interferences. Source data for Supplementary Fig. 79 are provided as a Source Data file.

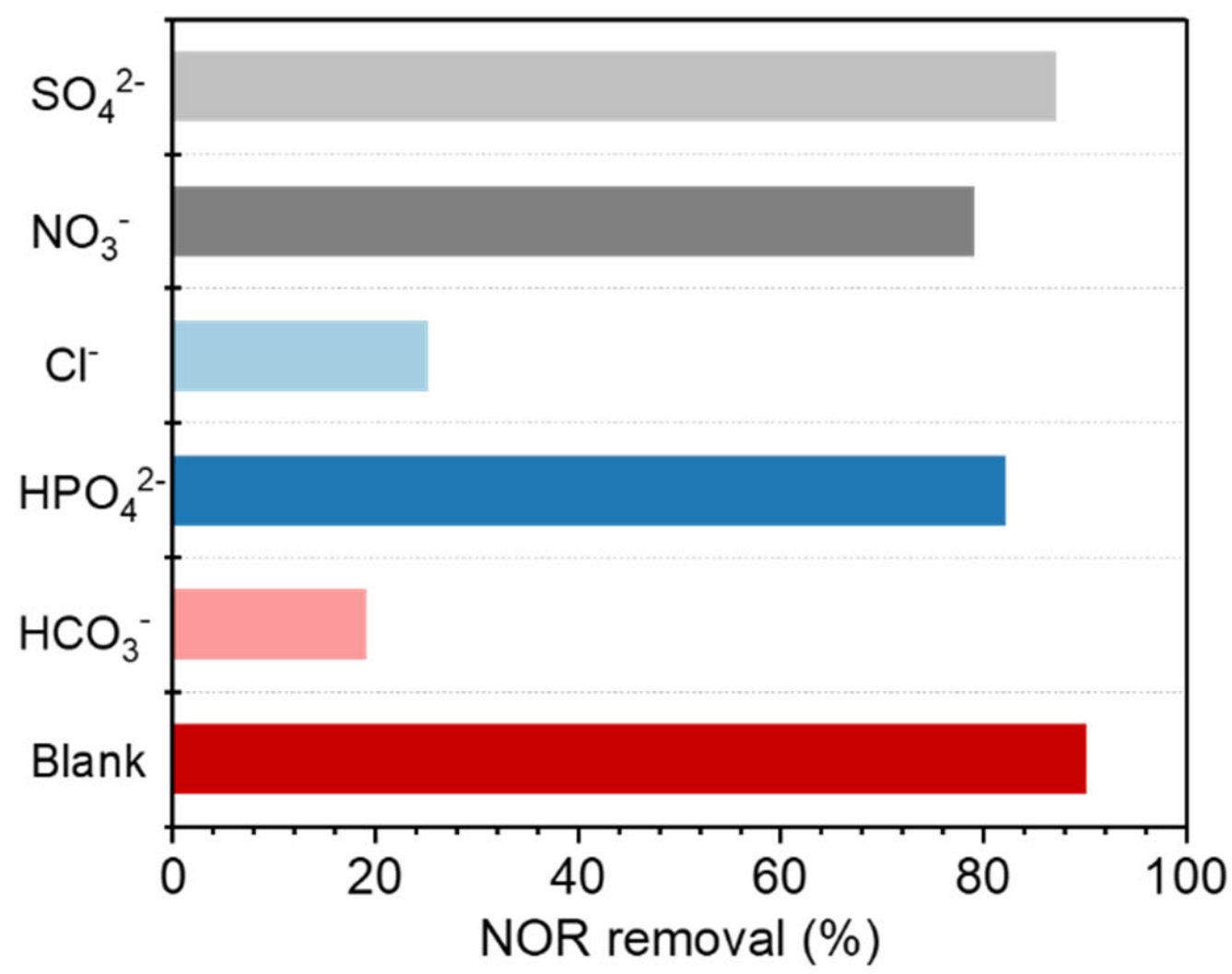

Supplementary Fig. 80 NOR removal rate of PSFZM electrode under different anion interferences. Source data for Supplementary Fig. 80 are provided as a Source Data file.

To simulate realistic water matrices, the effect of common inorganic ions on NOR degradation was evaluated (**Supplementary Fig. 80**). Notably, PO_4^{3-}, known to suppress traditional Fenton reactions, showed negligible effect on NOR degradation in the PSFZM-mediated hetero-electro-Fenton system. Although Cl^- and HCO_3^- generated secondary radicals (Cl•, $CO_3^{•-}$) through scavenging of •OH, the overall degradation efficiency remained high (**Supplementary Fig. 80**), attesting to the resilience of the ROS-driven mechanism.

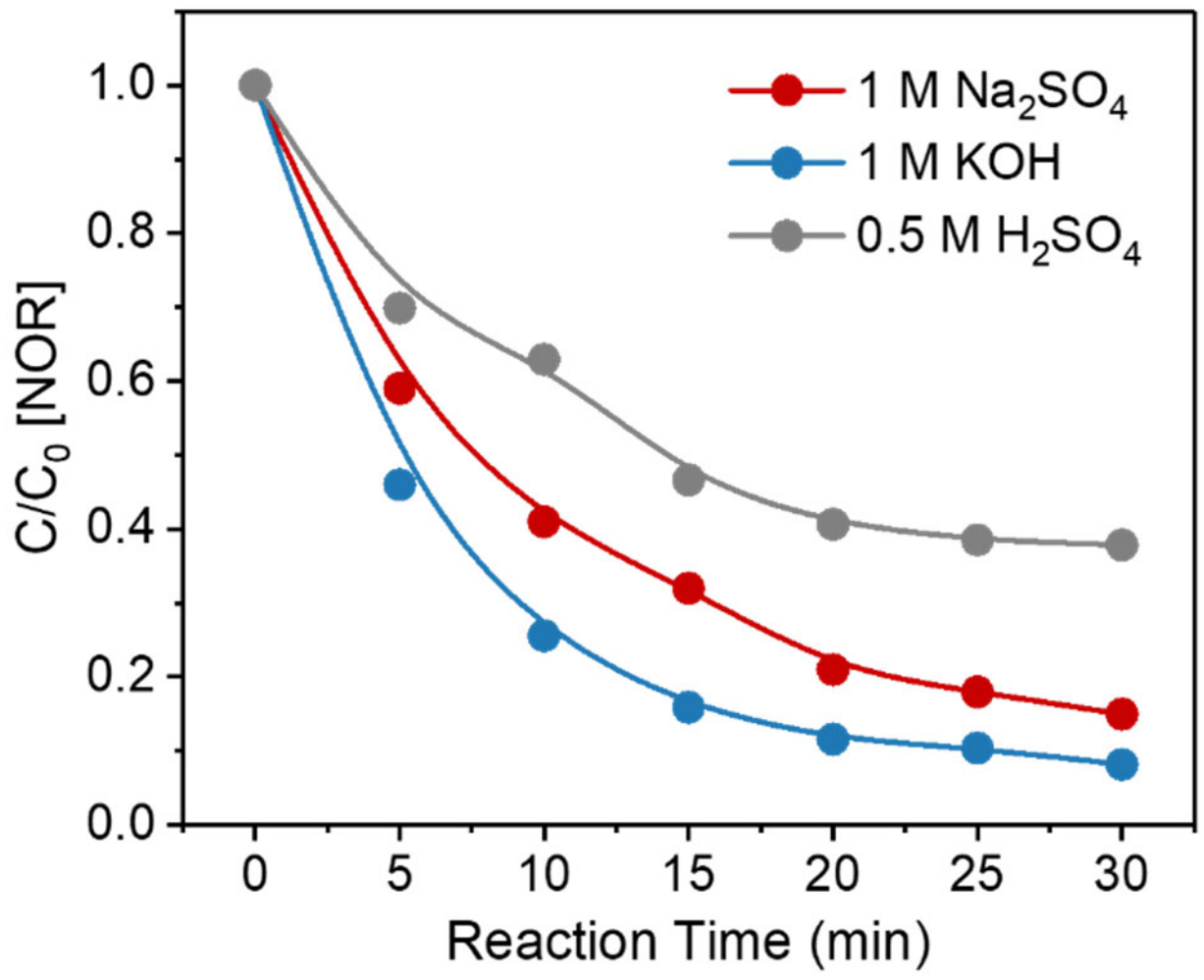

Supplementary Fig. 81 NOR degradation of PSFZM electrode in electrolytes with different pH values. Source data for Supplementary Fig. 81 are provided as a Source Data file.

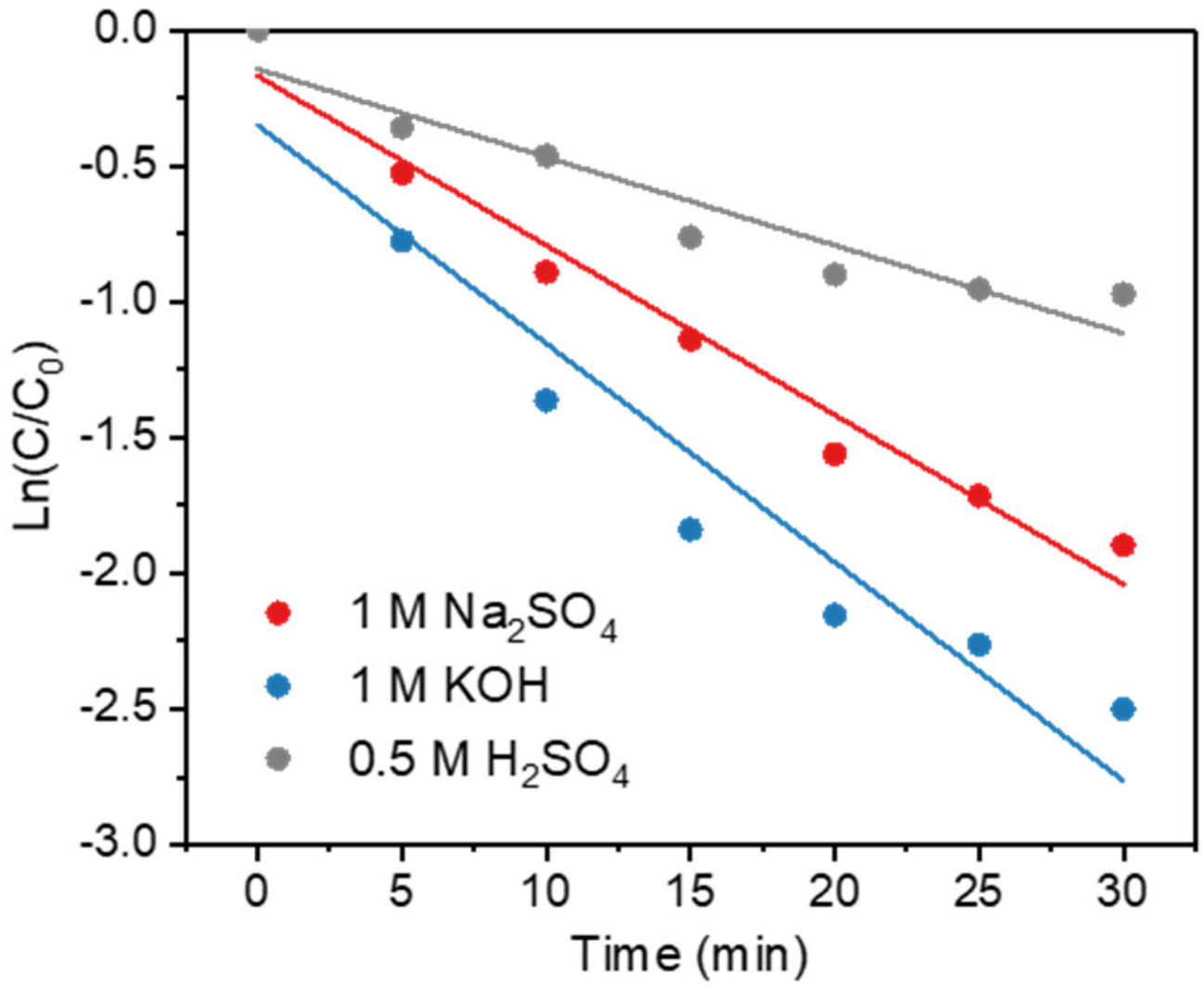

Supplementary Fig. 82 Corresponding first-order kinetics of PSFZM degradation system in different electrolytes. Source data for Supplementary Fig. 82 are provided as a Source Data file.

The PSFZM electrode also demonstrated broad pH adaptability, maintaining high activity across the pH 1-14 range, with optimal degradation (92.7%) under alkaline conditions (**Supplementary Fig. 82**). In contrast, acidic conditions reduced degradation efficiency (57.8%), possibly due to a shift toward the four-electron ORR pathway, which favors direct water formation over ROS generation (**Supplementary Fig. 82**).

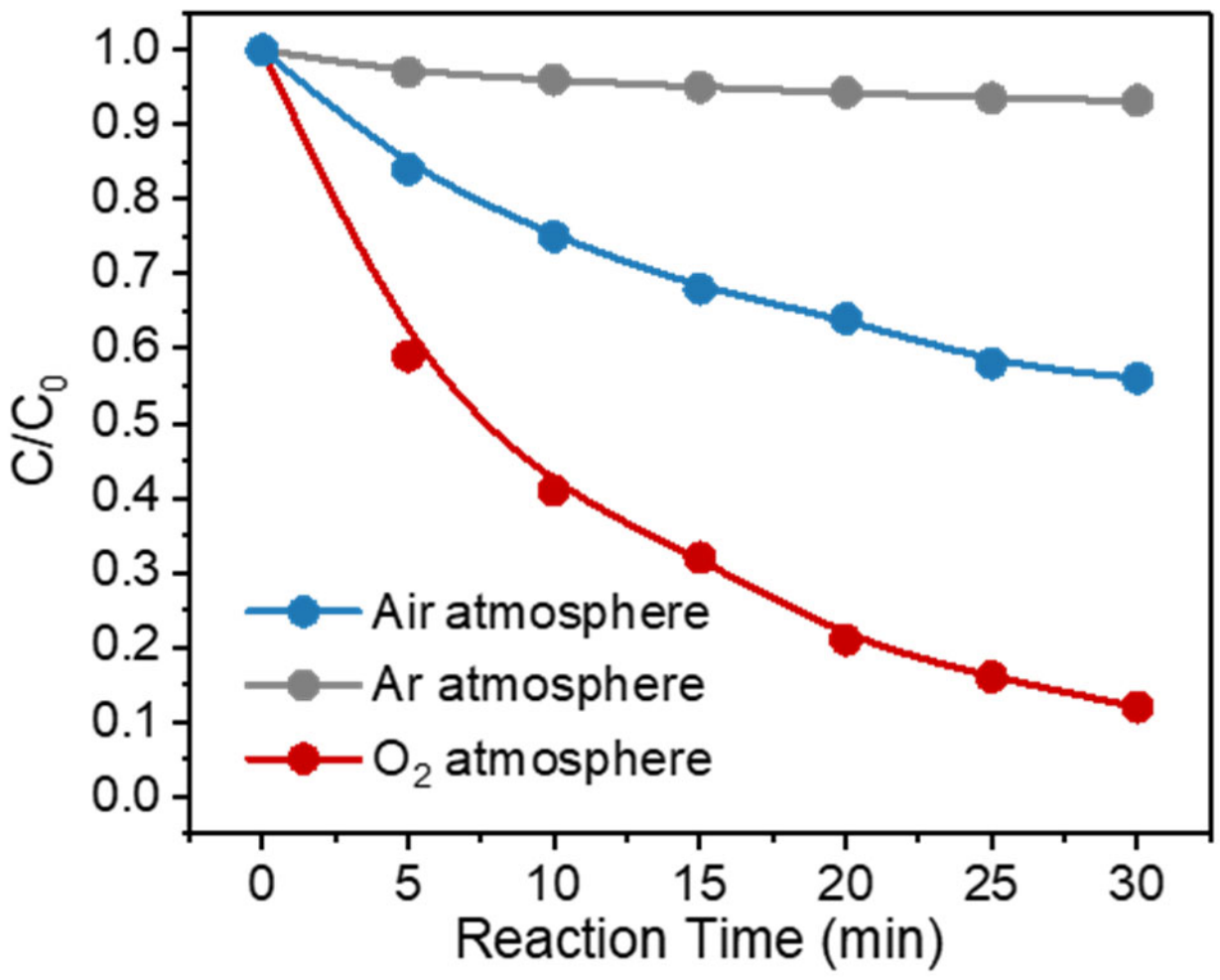

Supplementary Fig. 83 NOR degradation of PSFZM electrode in different saturated atmospheres. Source data for Supplementary Fig. 83 are provided as a Source Data file.

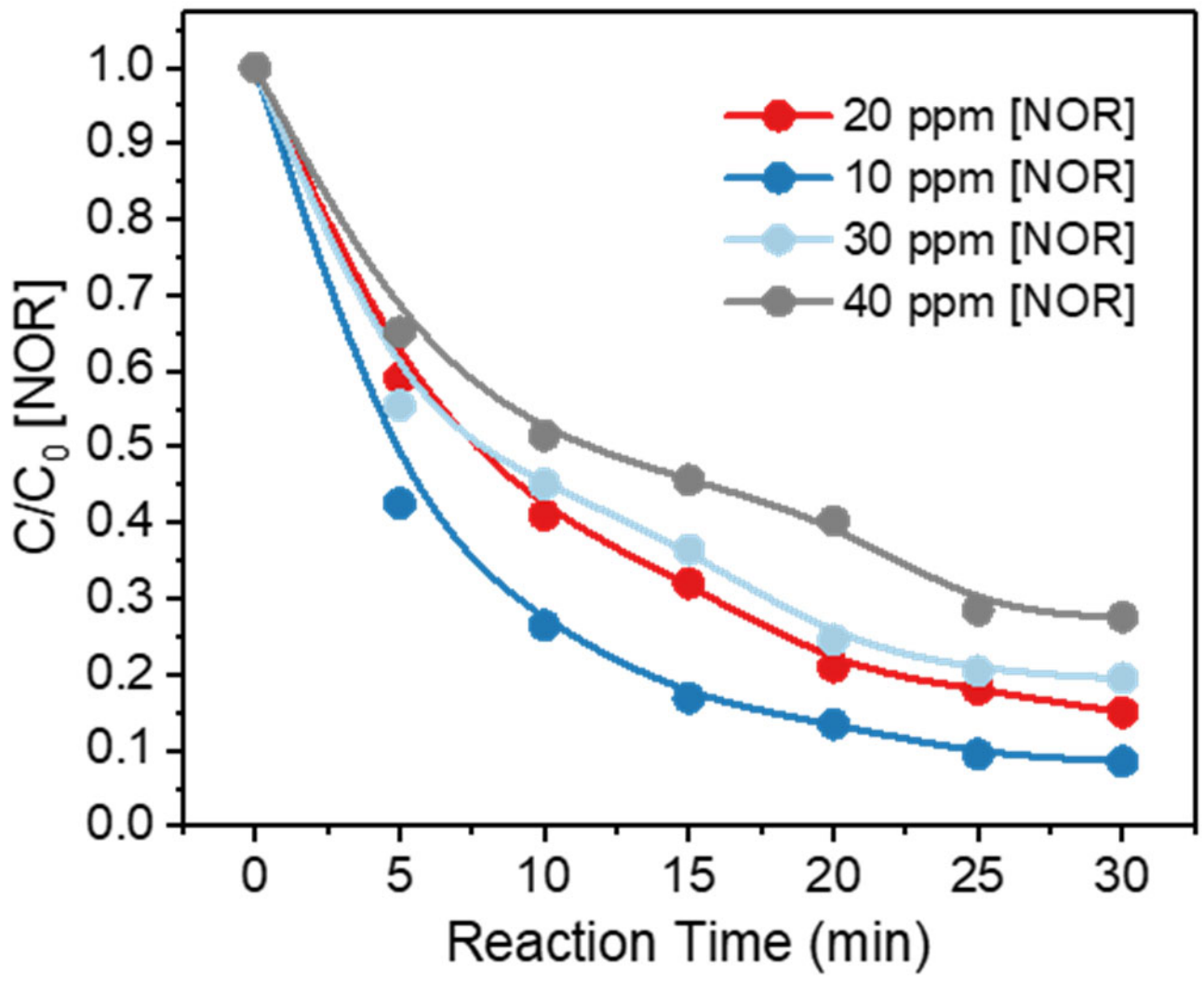

Supplementary Fig. 84 Degradation curves of NOR in PSFZM system in different electrolytes. Source data for Supplementary Fig. 84 are provided as a Source Data file.

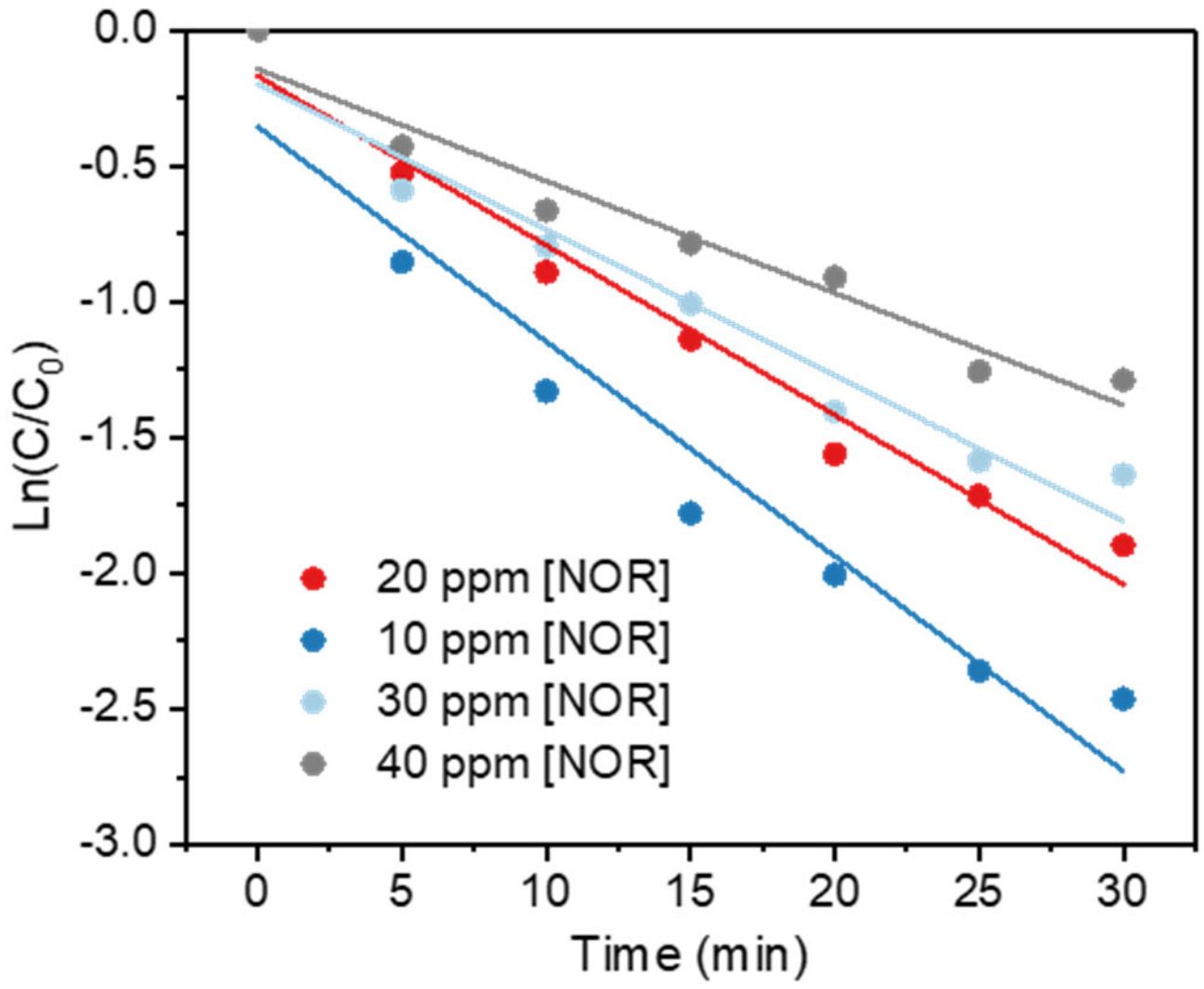

Supplementary Fig. 85 Corresponding first-order kinetics of different catalyst in the degradation system. Source data for Supplementary Fig. 85 are provided as a Source Data file.

Control experiments under O_2-, Ar-, and air-saturated conditions further confirmed that ROS responsible for pollutant degradation originated from cathodic oxygen reduction (**Supplementary Fig. 85**). Furthermore, PSFZM maintained high removal efficiency across varied initial NOR concentrations, demonstrating suitability for handling different contamination loads in practical wastewater (**Supplementary Figs. 84, 85**).

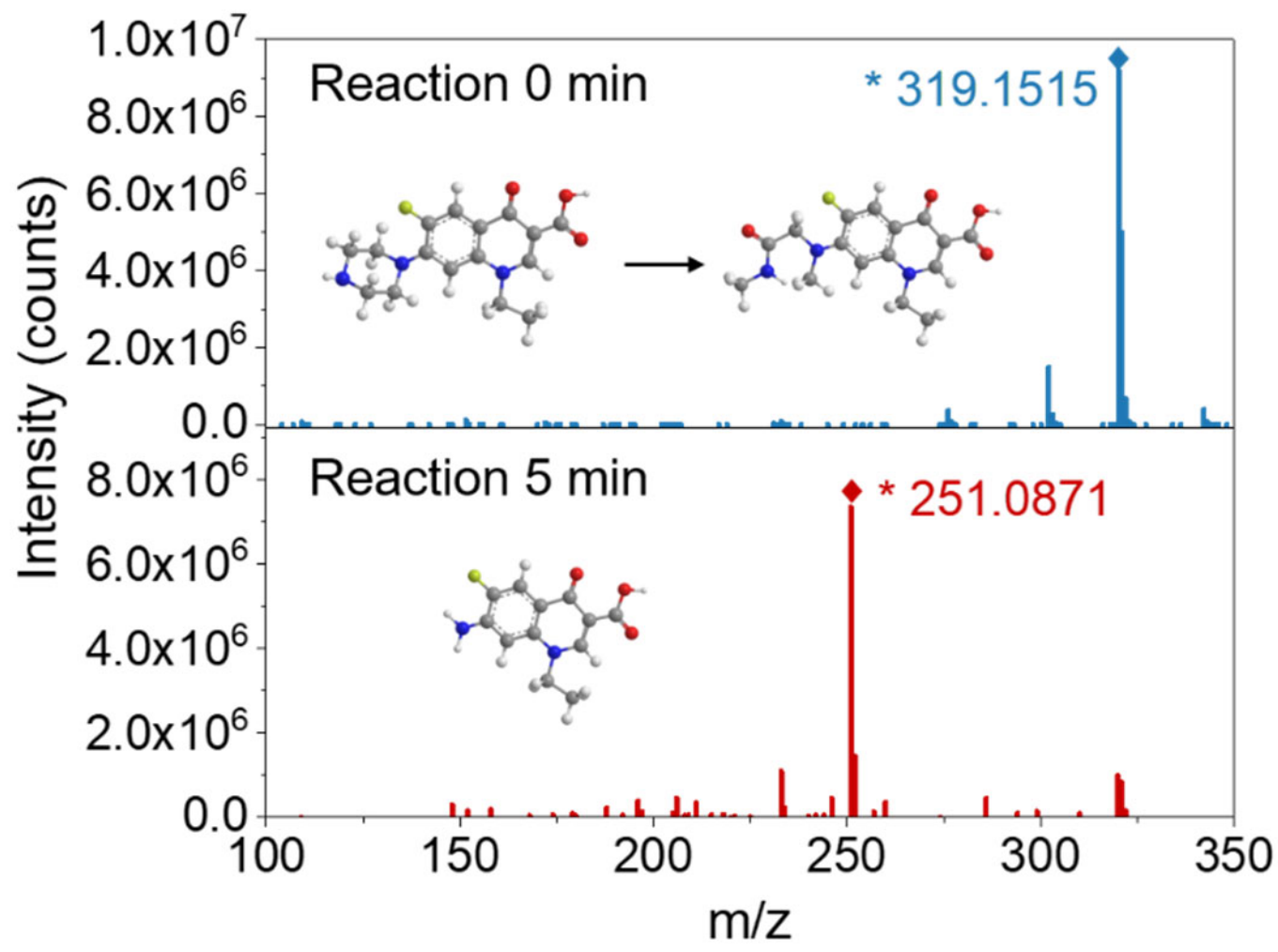

Supplementary Fig. 86 Changes in organic matter content determined by liquid chromatography-mass spectrometry (LC-MS) at the beginning of NOR degradation reaction and 5 min after degradation. Colors represent: C (gray), H (white), O (red), N (blue), F (green). Source data for Supplementary Fig. 86 are provided as a Source Data file. The illustration shows the chemical structural formulas of norfloxacin and the depiperazinyl-ring intermediate.

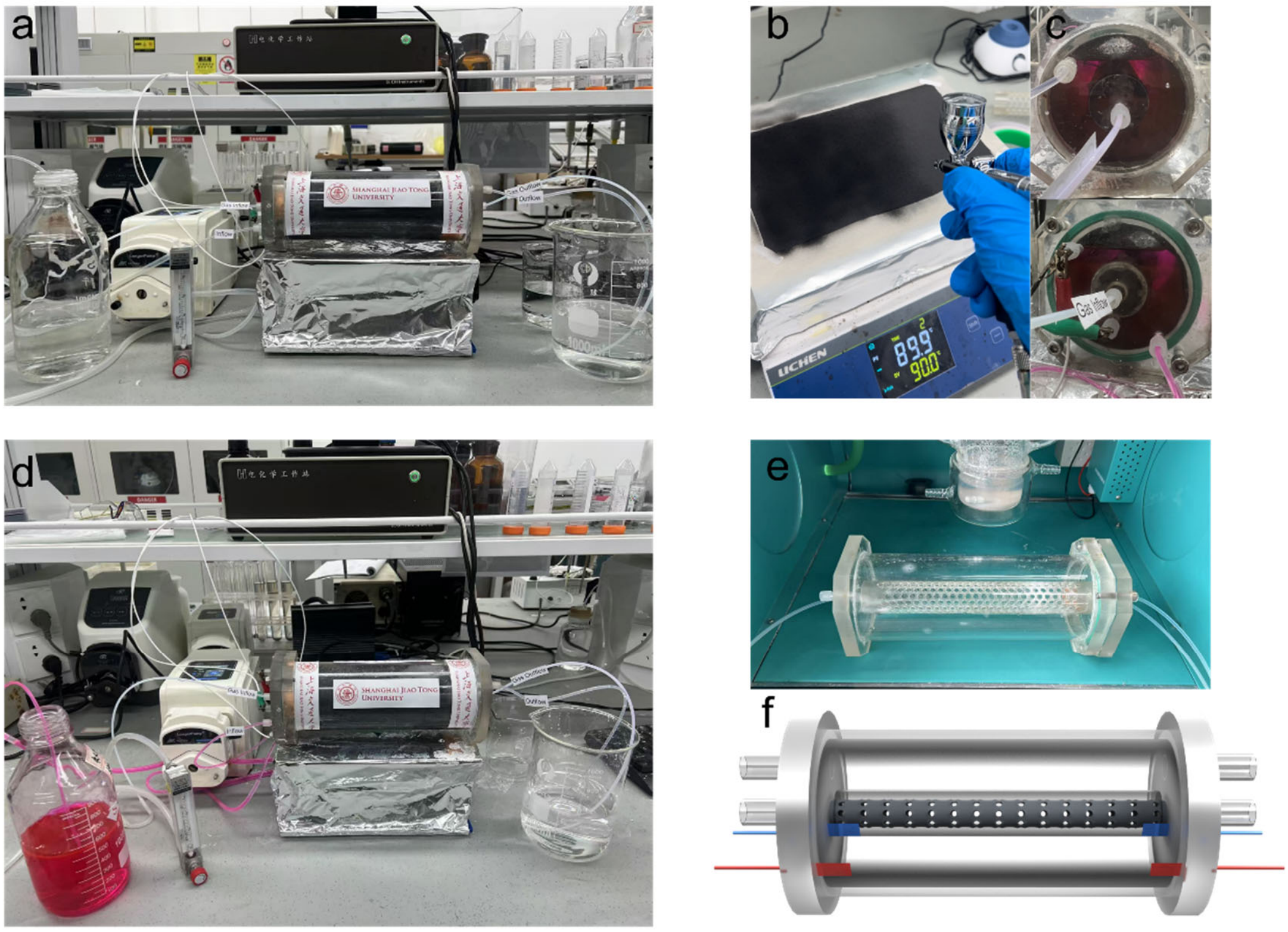

Supplementary Fig. 87 **a** Physical picture of a new membraneless flow electrolyzer with PSFZM as a symmetrical electrode assembly. **b** PSFZM electrode prepared by spraying ink on carbon cloth. **c** Side view of the device in operation. **d** The device degrades RhB to verify the treatment effect of organic matter. **e** Physical perspective picture of the inside of the device. **f** Schematic diagram of the device.

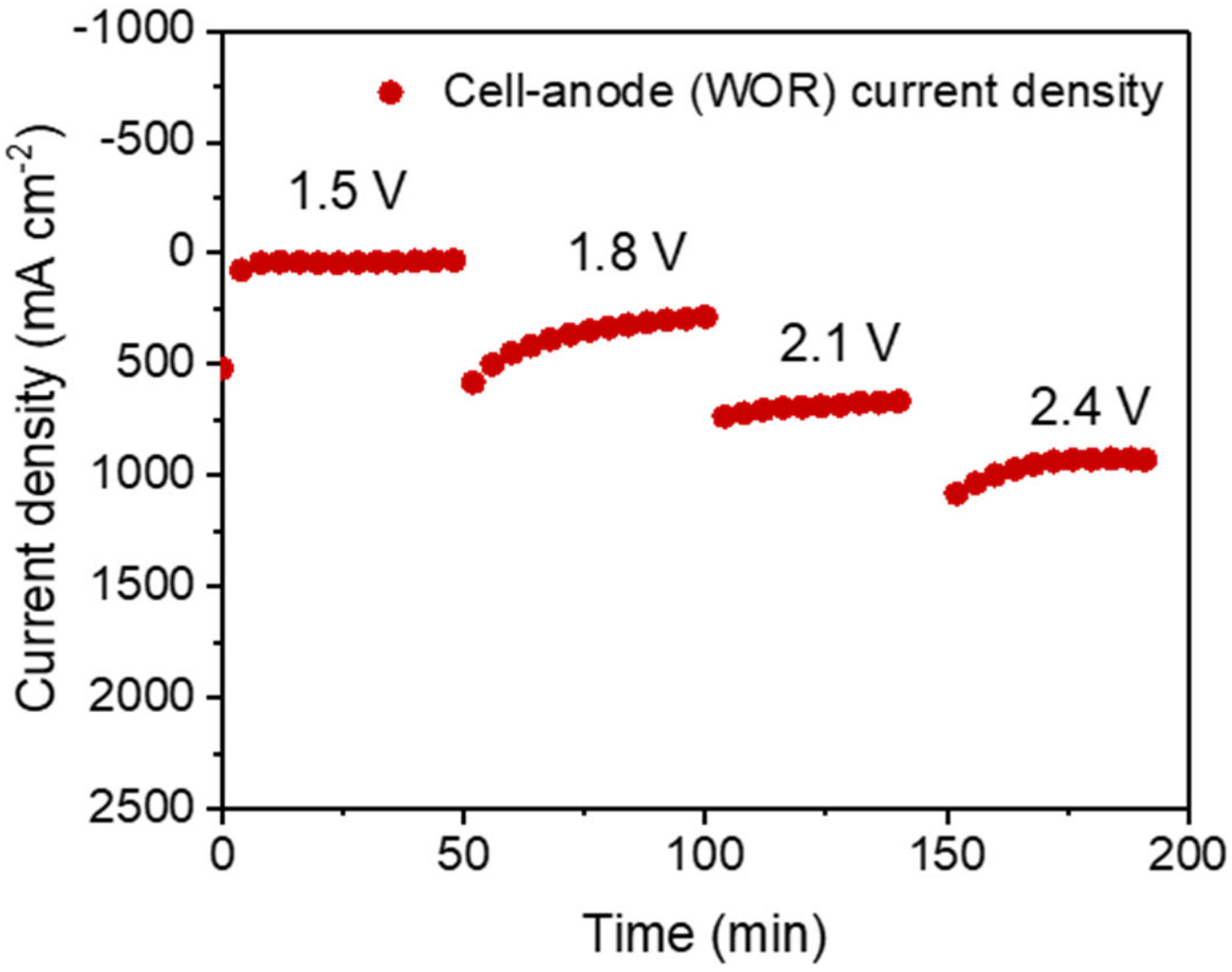

Supplementary Fig. 88 To systematically investigate the e$^-$ WOR selectivity of PSFZM, we performed device tests under chronoamperometric (CA) conditions from 1.5 to 2.4 V. Source data for Supplementary Fig. 88 are provided as a Source Data file.

Note: First, we evaluate the WOR performance of PSFZM. DFT calculations reveal that PSFZM exhibits the lowest Gibbs free energy for the rate-determining step (ΔG_{RDS}) of the electron-driven WOR (**Fig. 6b**). Notably, the $\Delta G_{*OH} > 2.4$ eV suggests weak *OH binding on the catalyst surface, suppressing the evolution of H_2O_2 or O_2 and instead favoring •OH production. Electrochemical measurements under 1.0 M Na_2SO_4 further corroborate these findings, with FE for •OH formation exceeding 50% at >2.0 V and a peak FE_{anode} of 58.9% achieving at ~2.5 V, alongside a high current density of ~200 mA cm^{-2} (**Figs. 6c,d and Supplementary Fig. 88**). Chronoamperometric stability tests further validate the system's scalability and robustness for sustained •OH generation.

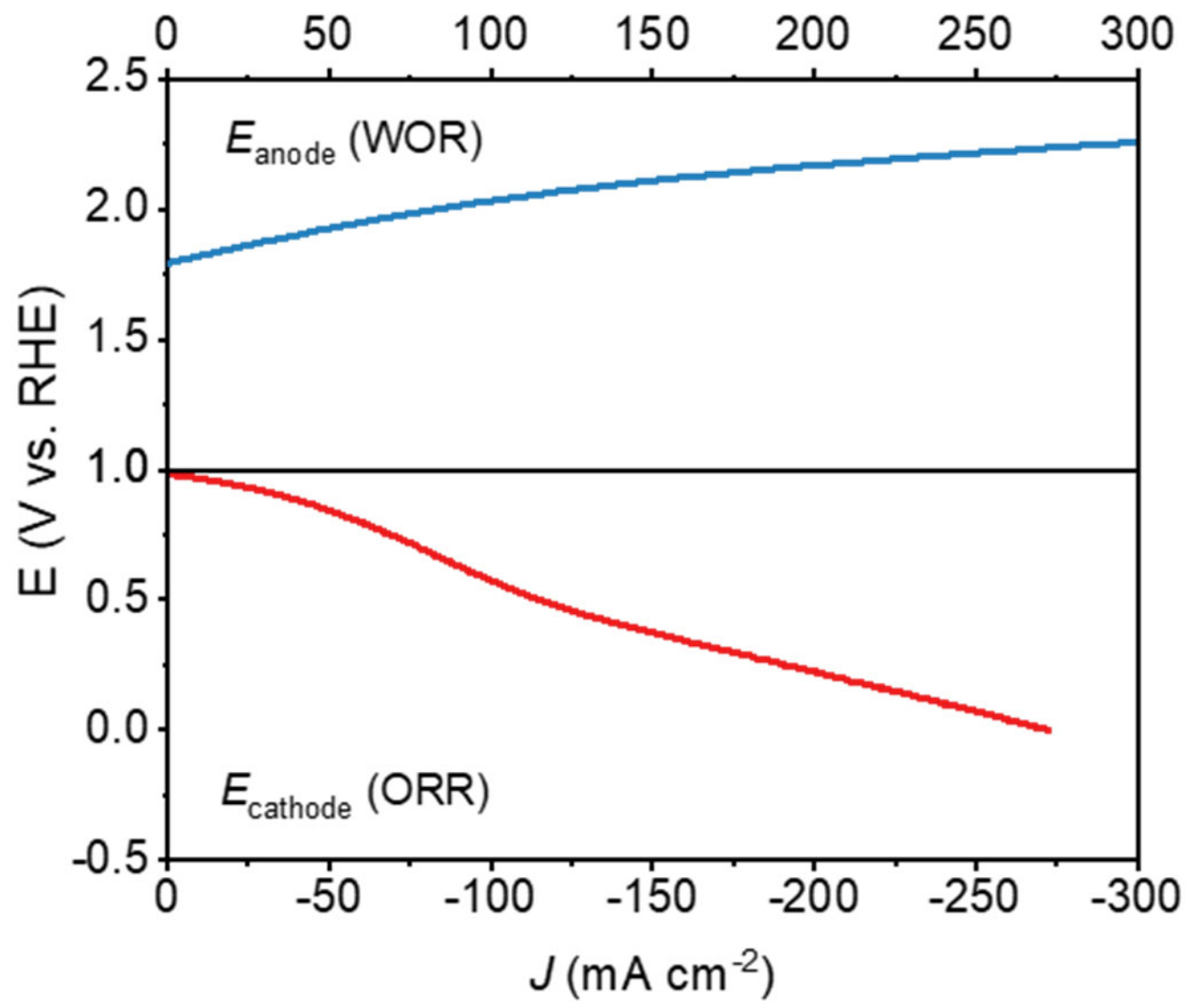

Supplementary Fig. 89 Polarization I-V curves of PSFZM || PSFZM in a flow electrolyzer, tested in 1.0 M Na_2SO_4. Source data for Supplementary Fig. 89 are provided as a Source Data file.

Full-cell polarization curves (**Supplementary Fig. 89**) show a low onset voltage of just 0.77 V and a steep increase in current density at higher voltages, attributed to the synergistic e^- WOR || $3e^-$ ORR catalytic performance of PSFZM.

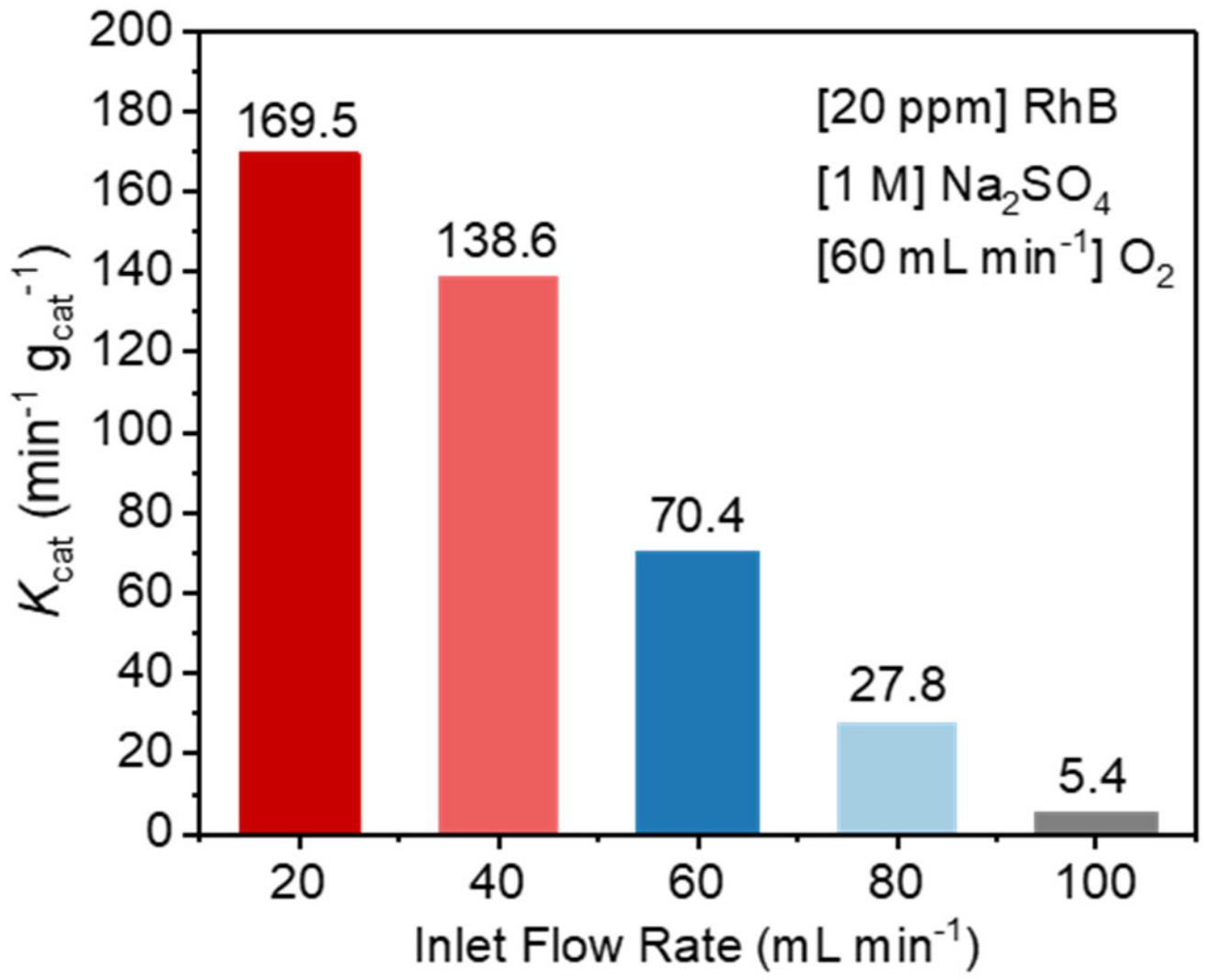

Supplementary Fig. 90 First-order kinetics of ROS generation rate and RhB degradation at inlet water flow rates with different input RhB contaminants. Source data for Supplementary Fig. 90 are provided as a Source Data file.

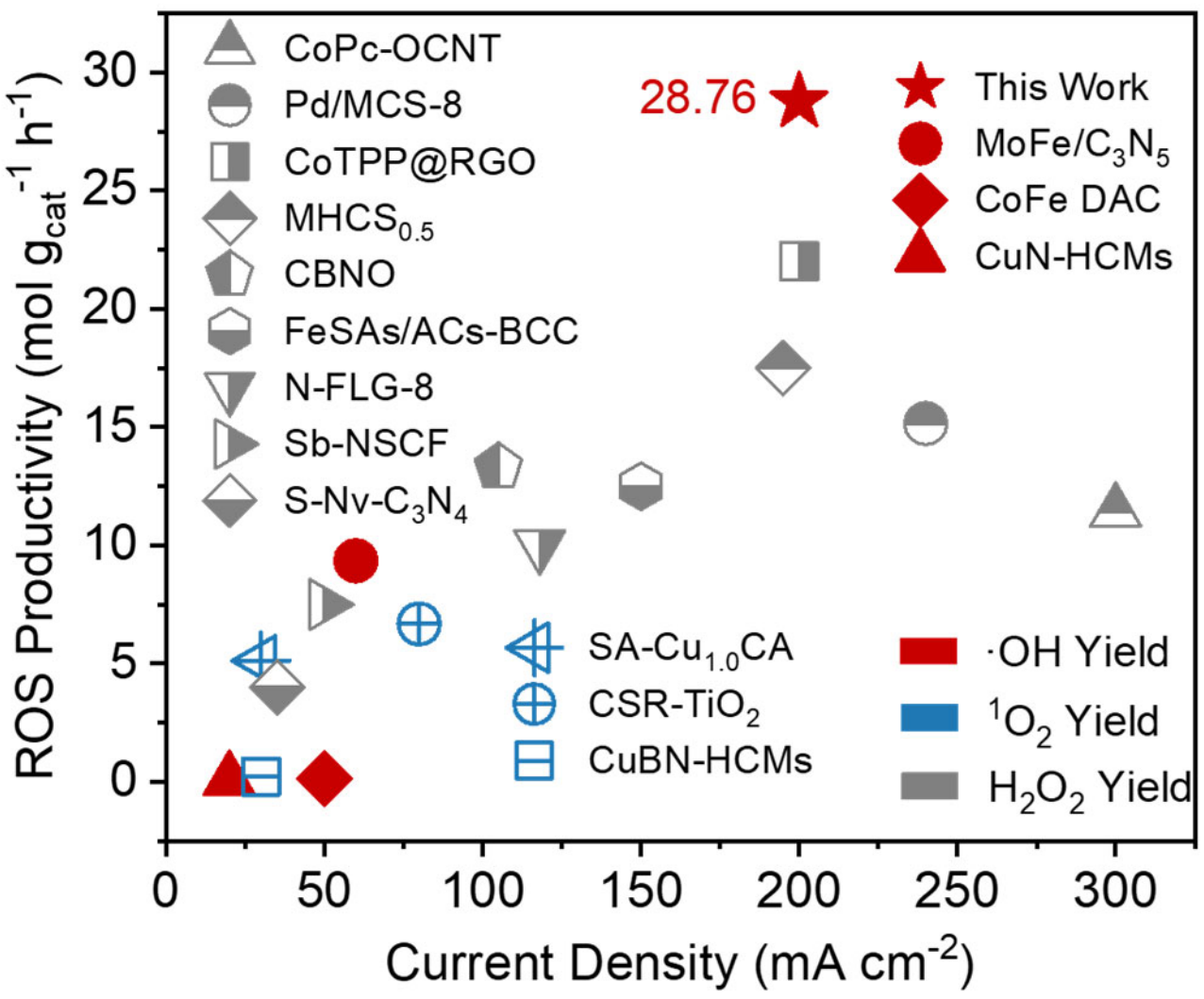

Supplementary Fig. 91 Establish a function diagram of electrolyzer current density and ROS yield for comparison with some advanced catalysts. Source data for Supplementary Fig. 91 are provided as a Source Data file.

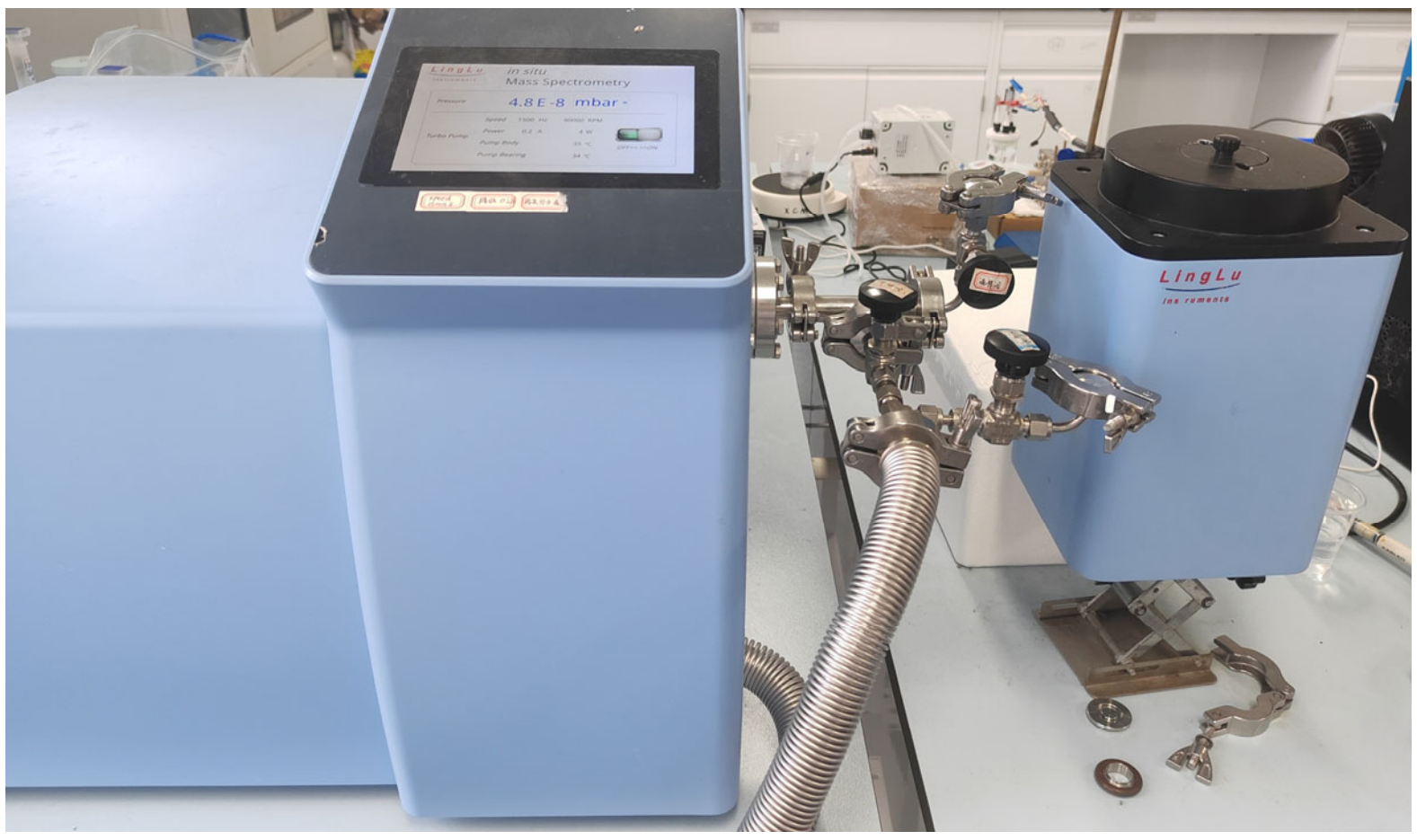

Supplementary Fig. 92 Photograph of the DEMS setup and electrochemical cell.

Supplementary Table 1. Rietveld-refined crystallographic parameters of PSFZ, PSFM and PSFZM.

Parameter	PSFZ	PSFM	PSFZM
**Nominal composition**	$PrSrFe_{0.75}Zn_{0.25}O_{4-\delta}$	$PrSrFe_{0.75}Mo_{0.25}O_{4-\delta}$	$PrSrFe_{0.5}Zn_{0.25}Mo_{0.25}O_{4-\delta}$
**Space group**	I4/mmm	I4/mmm	I4/mmm
**Crystal system**	Tetragonal	Tetragonal	Tetragonal
**a = b / Å**	3.848739	3.855880	3.858459
**c / Å**	12.670967	12.651975	12.673535
**V / Å^3**	187.692	188.107	188.680
**c/a**	3.292	3.281	3.285
**B-site occupation**	$Fe_{0.75}Zn_{0.25}$	$Fe_{0.75}Mo_{0.25}$	$Fe_{0.5}Zn_{0.25}Mo_{0.25}$
**O1 occupancy**	0.772	0.780	0.560
**O2 occupancy**	0.986	0.975	0.976
**B–O$_{eq}$ / Å**	1.924	1.928	1.929
**B–O$_{ap}$ / Å**	1.995	1.990	2.058
**B–O$_{ap}$/B–O$_{eq}$**	1.037	1.032	1.067
**Rwp / %**	6.81	8.77	8.60
**Rp / %**	5.03	5.92	5.92
**RF / %**	4.03	5.72	4.92
**RF^2 / %**	6.70	13.18	8.05

Supplementary Table 2. EXAFS fitting parameters at the Zn *K*-edge for various samples.

Sample	Shell	Bond length (Å)	Coordination Number	σ^2 (Å^2)	E0 shift (eV)	R-factor
**PSFZM**	Zn-O	1.95±0.01	3.7±0.6	0.003±0.002	1.3±2.0	0.014
	Zn-O-Zn	3.28±0.01	3.1±0.4	0.011±0.008		
	Zn-O-M	3.88±0.01	5.0±0.6	0.016±0.010		
**PSFZ**	Zn-O	1.99±0.02	4.3±1.0	0.003±0.001	0.8±2.4	0.005
	Zn-Zn	2.97±0.01	6.0±1.6	0.012±0.004		
	Zn-O-Zn	3.39±0.01	3.0±0.9	0.011±0.004		
	Zn-O-M	3.91±0.03	12.3±0.8	0.007±0.004		

Note: The value of the amplitude reduction factor (S_0^2) was fixed to 0.70; Bond length is the interatomic distance; CN is the coordination number; σ^2 is Debye-Waller factor (a measure of thermal and static disorder in absorber scatter distance); E_0 shift is edge-energy shift (the difference between the zero kinetic energy value of the sample and that of the theoretical model); R factor is used to value the goodness of the fitting.

Supplementary Table 3. Comparison of the performance of current advanced catalysts in O_2 activation systems. (Comparison of activation type, ROS type, ROS yield, degradation of organic matter, and first-order kinetics, etc.)

**Catalyst**	**Type**	**ROS Type**	**Accumulation of ROS**	**Pollutant**	**Rate constant**	**Ref.**
**PSFZM**	Non-Fenton $3e^-$ ORR	•OH	821 μmol h^{-1}	Norfloxacin	0.35 min^{-1}	This work
**CoFe DAC**	Non-Fenton $3e^-$ ORR	•OH	56.8 μmol h^{-1}	Phenol	0.0107 min^{-1}	1
**FeCo-ZIF**	Non-Fenton $3e^-$ ORR	•OH	~100 μmol h^{-1}	Sulfamethoxazole	0.02–0.03 min^{-1}	2
**Ni@N-CNT**	Non-Fenton $3e^-$ ORR	•OH	~100 μmol h^{-1}	Bisphenol A	0.12 min^{-1}	3
**$Cu/CoSe_2/C$**	Non-Fenton $3e^-$ ORR	•OH	100 μmol h^{-1}	Ciprofloxacin	0.06 min^{-1}	4
**FeCo@C**	Non-Fenton $3e^-$ ORR	•OH	/	Ciprofloxacin	1.44 min^{-1}	5
**CuBN-HCMs**	Non-Fenton $3e^-$ ORR	$^1O_2$	337.1 μmol h^{-1}	Phenol	0.073 min^{-1}	6
**CuN_4**	Non-Fenton $3e^-$ ORR	$^1O_2$	36 μmol h^{-1}	Natural organic	/	7
**Mn/Fe@PC-CP**	$2e^-$ ORR (Fe^{2+}/Fe^{3+})	H_2O_2	6.7 μmol h^{-1}	Triclosan	0.062 min^{-1}	8
**Cu/In_2Se_3**	$2e^-$ ORR (Fe^{2+}/Fe^{3+})	H_2O_2	12.2 μmol h^{-1}	Ciprofloxacin	/	9
**$MoFe/C_3N_5$**	Fenton-like $3e^-$ ORR	H_2O_2	32 μmol h^{-1}	Ibuprofen	0.03 min^{-1}	10
**FeNC@C**	Fenton-like $3e^-$ ORR	H_2O_2	200 μmol h^{-1}	2-Chlorophenol	0.07 min^{-1}	11

Supplementary Table 4. EXAFS fitting parameters at the Mo *K*-edge for various samples.

Sample	Shell	Bond length (Å)	Coordination Number	σ^2 (Å^2)	E0 shift(eV)	R-factor
**PSFZM**	Mo-O	1.77±0.01	4.0±0.2	0.001±0.001	6.7±0.9	0.012
	Mo-O-M	3.99±0.01	6.8±0.8	0.012±0.004		
**PSFM**	Mo-O	1.77±0.01	3.9±0.3	0.001±0.001	6.4±1.0	0.014
	Mo-O-M	3.98±0.02	5.6±0.6	0.011±0.004		

Note: The value of the amplitude reduction factor (S_0^2) was fixed to 0.95; Bond length is the interatomic distance; CN is the coordination number; σ^2 is Debye-Waller factor (a measure of thermal and static disorder in absorber scatter distance); E_0 shift is edge-energy shift (the difference between the zero kinetic energy value of the sample and that of the theoretical model); R factor is used to value the goodness of the fitting.

Supplementary Table 5. EXAFS fitting parameters at the Fe *K*-edge for various samples.

Sample	Shell	Bond length (Å)	Coordination Number	σ^2 (Å^2)	E0 shift (eV)	R-factor
**PSFZM**	Fe-O	1.95±0.02	4.2±0.8	0.003±0.002	-2.2±0.5	0.014
	Fe-Fe	3.00±0.04	3.8±0.5	0.005±0.002		
	Fe-O	3.44±0.04	3.7±0.9	0.012±0.006		
	Fe-O-M	3.80±0.04	6.2±1.0	0.010±0.006		
**PSFZ**	Fe-O	1.93±0.02	4.1±0.8	0.002±0.002	-3.2±2.4	0.008
	Fe-Fe	2.99±0.04	4.0±0.3	0.004±0.002		
	Fe-O	3.45±0.03	3.9±0.4	0.007±0.006		
	Fe-O-M	3.79±0.02	8.2±1.0	0.004±0.001		
**PSFM**	Fe-O	1.94±0.01	4.4±0.7	0.002±0.002	-3.1±1.9	0.006
	Fe-Fe	3.02±0.04	3.5±0.7	0.014±0.006		
	Fe-O	3.48±0.02	4.1±0.4	0.009±0.007		
	Fe-O-M	3.82±0.02	5.6±1.4	0.004±0.001		

Note: The value of the amplitude reduction factor (S_0^2) was fixed to 0.72; Bond length is the interatomic distance; CN is the coordination number; σ^2 is Debye-Waller factor (a measure of thermal and static disorder in absorber scatter distance); E_0 shift is edge-energy shift (the difference between the zero kinetic energy value of the sample and that of the theoretical model); R factor is used to value the goodness of the fitting.

Supplementary Table 6. The O_2 activation system was compared with the H_2O_2 activation system and the PMS activation system from five dimensions: MA, SA, MRR, TOC and RMI.

	**O_2 activation**	**PMS activation**	**H_2O_2 activation**
**Mass activity (MA) (g min^{-1} g^{-1})**	13.33	0.95	3.68
**Surface activity (SA) (g min^{-1} m^{-2})**	0.0667	0.00212	0.0245
**Metal retention rate (MRR) (%)**	99.96	89.56	85.65
**Total organic carbon (TOC) (%)**	63.41	70.64	59.63
**Raw material input (RMI) (mg)**	0	20	20

Supplementary Table 7. Comparison of ROS generation rates of different generation systems was determined.

Catalysts	Type	ROS generation rate ($\mu mol \cdot L^{-1} \cdot min^{-1}$)	Ref.
**PSFZM**	O_2 activation	228.06 (821 $\mu mol\ h^{-1}$)	This work
**CSR-TiO_2**	O_2 activation	148.26	12
**g-C_3N_4-CoWO**	H_2O_2 activation	3.3	13
**Mo-LDHs**	H_2O_2 activation	5.7	14
**CuCo LDH/BC**	H_2O_2 activation	6.9	15
**45Fe-POM/CNNS-$N_{vac.}$**	H_2O_2 activation	9.2	16
**Fe(III)/$CaSO_3$**	PMS activation	14.8	17
**DSLB-800**	PMS activation	19.5	18
**FeN_4/B**	PMS activation	227	19
**β-MnO_2**	PMS activation	29.5	20
**Cu-SA/Ti_3C_2Tx**	PMS activation	30.2	21
**$BiOIO_3$/γ-FeOOH**	O_2 activation	0.635	22
**Bi/Bi_2WO_{6-x}**	O_2 activation	3.6	23
**CMCN**	O_2 activation	17.0	24
**Trimethoprim**	O_2 activation	13.5	25
**iron phosphide**	O_2 activation	20.9	26
**SA-$Cu_{1.0}$CA**	O_2 activation	65.3	6

Note: To facilitate direct comparison with other systems, the units of yield have been converted.

References

1. Qin, X. *et al.* Highly Efficient Hydroxyl Radicals Production Boosted by the Atomically Dispersed Fe and Co Sites for Heterogeneous Electro-Fenton Oxidation. *Environ. Sci. Technol.* **57**, 2907–2917 (2023).
2. Cheng, S. et al. FeCo-ZIF derived carbon-encapsulated metal alloy as efficient cathode material for heterogeneous electro-Fenton reaction in 3-electron ORR pathway: Enhanced performance in alkaline condition. *Sep. Purif. Technol.* **325**, 124545 (2023).
3. Li, M. et al. Electrocatalytic transformation of oxygen to hydroxyl radicals via three-electron pathway using nitrogen-doped carbon nanotube-encapsulated nickel nanocatalysts for effective organic decontamination. *J. Hazard. Mater.* **452**, 131352 (2023).
4. Johnson, G. A. et al. Merged magnetic resonance and light sheet microscopy of the whole mouse brain. *Proc. Natl. Acad. Sci.* **120**, 2218617120 (2023).
5. Xiao, F. *et al.* Selective Electrocatalytic Reduction of Oxygen to Hydroxyl Radicals via 3-Electron Pathway with FeCo Alloy Encapsulated Carbon Aerogel for Fast and Complete Removing Pollutants. *Angew. Chemie Int. Ed.* **60**, 10375–10383 (2021).
6. Feng, Z. *et al.* New Insights into Selective Singlet Oxygen Production via the Typical Electroactivation of Oxygen for Water Decontamination. *Environ. Sci. Technol.* **57**, 17123–17131 (2023).
7. Zhu, G., Fan, X., Yu, Y., Liu, Y. & Quan, X. Regulating the Electronic Structure of Cu Single-Atom Catalysts toward Enhanced Electro-Fenton Degradation of Organic Contaminants via $^1O_2$ and •OH. *Environ. Sci. Technol.* **58**, 19545–19554 (2024).
8. Liu, Y. *et al.* Electronic Metal-Support Interactions Boost *OOH Intermediate Generation in Cu/In_2Se_3 for Electrochemical H_2O_2 Production. *Angew. Chemie* **136**, 202319470 (2024).
9. Dong, C. *et al.* Dual-functional single-atomic Mo/Fe clusters–decorated C_3N_5 via three electron-pathway in oxygen reduction reaction for tandemly removing

contaminants from water. *Proc. Natl. Acad. Sci.* **120**, 2305883120 (2023).

10. Hu, J. *et al.* Duet Fe_3C and FeN_x Sites for H_2O_2 Generation and Activation toward Enhanced Electro-Fenton Performance in Wastewater Treatment. *Environ. Sci. Technol.* **55**, 1260–1269 (2021).
11. Zhao Y., et al. α-Fe_2O_3 as a versatile and efficient oxygen atom transfer catalyst in combination with H_2O as the oxygen source. *Nat. Catal.* **4**, 684-691 (2021).
12. Wang, R. *et al.* Compressive-strained rutile TiO_2 enables O_2 mono-hydrogenation for singlet oxygen electrosynthesis. *Nat. Synth.* **4**, 754-764 (2025) .
13. Zhao S., Zhao X. Insights into the role of singlet oxygen in the photocatalytic hydrogen peroxide production over polyoxometalates-derived metal oxides incorporated into graphitic carbon nitride framework. *Appl. Catal., B* **250**, 408-418 (2019).
14. Sels B. F., De Vos D. E., Jacobs P. A. Kinetics of the oxygenation of unsaturated organics with singlet oxygen generated from H_2O_2 by a heterogeneous molybdenum catalyst. *J. Am. Chem. Soc.* **76**, 6916-6926 (2007).
15. Li L., et al. Enhancing hydrogen peroxide activation of CuCo layered double hydroxide by compositing with biochar: Performance and mechanism. *Sci. Total Environ.* **828**, 154188 (2022).
16. Jiang J., et al. Photo-Fenton degradation of emerging pollutants over Fe-POM nanoparticle/porous and ultrathin g-C_3N_4 nanosheet with rich nitrogen defect: Degradation mechanism, pathways, and products toxicity assessment. *Appl. Catal., B* **278**, 119349 (2020).
17. Zhou Z., et al. Insights into the removal of organic contaminants by calcium sulfite activation with Fe(III): Performance, kinetics, and mechanisms. *Water Res.* **221**, 118792 (2022).
18. Wang Y., et al. Sulfamethoxazole degradation by regulating active sites on distilled spirits leesderived biochar in a continuous flow fixed bed peroxymonosulfate reactor. *Appl. Catal., B* **310**, 121342 (2022).
19. Zhang, T. *et al.* Cooperative Spin Alignment Enhances Selective Singlet Oxygen

Generation in Wastewater Pre-Oxidation System. *Angew. Chemie Int. Ed.* **65**, e8246739 (2026).

20. Zhu S., Li X., Kang J., Duan X., Wang S. Persulfate activation on crystallographic manganese oxides: mechanism of singlet oxygen evolution for nonradical selective degradation of aqueous contaminants. *Environ. Sci. Technol.* **53**, 307-315 (2018).
21. Jin L., You S., Yao Y., Chen H., Wang Y., Liu Y. An electroactive single-atom copper anchored MXene nanohybrid filter for ultrafast water decontamination. *J. Mater. Chem. A* **9**, 25964-25973 (2021).
22. Xu, J., *et al.* Piezo-catalytic in-site H_2O_2 generation and activation across wide pH range to drive hydroxyl radical-mediated pollutant degradation. *Nat Commun* **16**, 7908 (2025).
23. Huang T., Tian F., Wen Z., Li G., Liang Y., Chen R. Synergistic mediation of metallic bismuth and oxygen vacancy in Bi/Bi_2WO_{6-x} to promote $^1O_2$ production for the photodegradation of bisphenol A and its analogues in water matrix. *J. Hazard. Mater.* **403**, 123661 (2021).
24. Chen, X., *et al.* Ultrafast energy-neutral molecular oxygen activation via atomically-adjacent bimetallic catalytic sites. *Nat Commun* **17**, 975 (2026).
25. Luo X., Zheng Z., Greaves J., Cooper W. J., Song W. Trimethoprim: Kinetic and mechanistic considerations in photochemical environmental fate and AOP treatment. *Water Res.* **46**, 1327-1336 (2012).
26. Zheng N., et al. In-situ production of singlet oxygen by dioxygen activation on iron phosphide for advanced oxidation processes. *Appl. Catal., B* **307**, 121157 (2022).
