# Supplementary material for: Single-atom-engineered perovskite enables near-theoretical-rate hydroxyl radical electrogeneration
Source: Nat Commun. 2026 Jul 24;17:9026. doi: 10.1038/s41467-026-75926-5 (PMC13507266; doi:10.1038/s41467-026-75926-5)
Supplement: Supplementary file 2 [file 41467_2026_75926_MOESM2_ESM.pdf]

## Description of Additional Supplementary Files

**File Name:** Supplementary Data 1

**Description:** The atomic coordinates for the optimized computational models used in the electronic structure calculations presented in this work.

**File Name:** Supplementary Data 2

**Description:** The DFT calculation model files (cif) for the catalysts and key intermediates in this work.
