# Supplementary material for: Single-atom-engineered perovskite enables near-theoretical-rate hydroxyl radical electrogeneration
Source: Nat Commun. 2026 Jul 24;17:9026. doi: 10.1038/s41467-026-75926-5 (PMC13507266; doi:10.1038/s41467-026-75926-5)
Supplement: Supplementary file 3 [file 41467_2026_75926_MOESM5_ESM.pdf]

Peer Review File

Single-atom-engineered perovskite enables near-theoretical-rate hydroxyl radical electrogeneration

Corresponding Author: Professor Emiliano Cortes

This file contains all reviewer reports in order by version, followed by all author rebuttals in order by version.

Version 0:

Reviewer comments:

Reviewer #1

(Remarks to the Author)
This manuscript reports a Zn–Mo co-engineered Ruddlesden–Popper perovskite, $Pr_{1.0}Sr_{1.0}Fe_{0.5}Zn_{0.25}Mo_{0.25}O_{4-\delta}$ (PSFZM), for hydroxyl radical electrogeneration through a proposed single-site active-domain mechanism. The authors combine structural characterization, RRDE, operando/intermediate spectroscopy, and DFT calculations, and further extend the catalyst to a membraneless flow electrolyzer. The topic is timely and potentially important for electrochemical advanced oxidation and selective oxygen activation. The concept of coupling intermediate stabilization and O–O bond activation within one lattice-confined active domain is interesting. However, in its current form, I do not believe the manuscript yet meets the standard for publication in Nature Communications. The central claims regarding a direct $3e^-$ ORR pathway to •OH and a single-atom-engineered active domain are not supported by sufficiently compelling evidence. In addition, there is insufficient rigor in radical quantification and Faradaic efficiency analysis, together with multiple inconsistencies in the Methods and Supporting Information. Addressing these issues would substantially improve the overall quality, clarity, and robustness of the manuscript.

1. The manuscript presents the system as bypassing the conventional decoupled $2e^-$ ORR + $1e^-$ H_2O_2 activation pathway. However, the authors' own operando FTIR discussion describes a sequential O_2→*OOH→*H_2O_2→•OH evolution, and the H_2O_2-addition experiment is interpreted to show that surface-adsorbed H_2O_2 is the immediate precursor to •OH. This evidence is more consistent with a confined tandem mechanism within one local domain than with a rigorously direct $3e^-$ pathway. The current wording therefore appears stronger than the data support. The authors should either substantially strengthen the mechanistic evidence or moderate the central claim throughout the manuscript.
2. The XANES/EXAFS results suggest a Zn–O–Mo local coordination environment, and microscopy indicates a porous nanofiber morphology with elemental dispersion. However, the current evidence does not fully justify the strength of the term "single-atom-engineered" or the claim that Zn atoms are periodically enriched within a distorted tetragonal unit cell. More statistically robust atom-resolved STEM analysis, detailed EXAFS fitting parameters with model comparisons, and additional structural characterization (e.g., Rietveld refinement or pair distribution functon(PDF) analysis) are needed. These would help to rigorously establish the proposed local structure and exclude alternative explanations such as nanoscale segregation or short-range disorder.
3. The XAFS analysis is not fully convincing. In particular, the assignment of the PSFZM absorption edge (lines 103–105) is not clearly supported by the presented spectra. Reference 33 does not appear to substantiate the conclusion of Zn–O–Mo coordination, and more direct coordination evidence is required. For the proposed single-site active domain, Mo K-edge EXAFS in R-space should also show features corresponding to Mo–O–Zn coordination. The assignment of Zn–O–Fe coordination appearing beyond ~3 Å is questionable. Furthermore, there are inconsistencies between Supplementary Fig. 7 and Fig. 2i. The authors should carefully re-examine the data, provide a more rigorous analysis of coordination environments, and include detailed fitting models and parameters.
4. In line 121, the RRDE results are described as delivering a current density of 4.2 mA cm^{-2} at 0.07–0.10 V (vs. RHE), approaching the theoretical diffusion-limited current for a $3e^-$ ORR pathway. However, the data at 0.10 V (vs. RHE) are not shown, and the electron transfer number appears to increase in this potential range, possibly entering the HER regime. ESR measurements should also be extended to include data at 0.10 V (vs. RHE) to ensure consistency.
5. The •OH concentrations determined using benzoic acid and coumarin fluorescence methods show significant discrepancies, which require explanation. The method used to calculate oxygen utilization efficiency should be clearly described. More importantly, the quantification of •OH and the derivation of Faradaic efficiency lack sufficient rigor and transparency. Detailed calibration procedures, error analysis, and clear definitions are necessary.
6. The calculated free-energy landscape supports the special role of the Zn–O–Mo motif, and the comparison across local

motifs is valuable. However, the manuscript uses language such as “barrier-free conversion” and describes the rate-determining step as exergonic on PSFZM. Free-energy profiles alone do not establish the absence of kinetic barriers. If the authors wish to make a kinetics-based argument, transition-state calculations (e.g., CI-NEB) are required. At a minimum, the language should be softened and thermodynamic descriptors should not be conflated with reaction barriers.
7. The full cell is constructed using PSFZM as both cathode and anode, and the authors report a peak FE of 64.7% for •OH generation, while the anode alone is also claimed to generate •OH with FE exceeding 50% and peaking at 58.9%. It remains unclear how the individual cathodic and anodic ROS contributions were separated, how crossover or recombination was excluded, and how the full-cell FE was defined relative to the half-cell values. A more rigorous species balance and electrode-resolved analysis are needed.
8. There are serious inconsistencies in the Methods and Supporting Information that undermine confidence in the study. A particularly concerning example is the LC–MS method: Supplementary Note 3 reports a flow rate of 0.25 mL min^{-1} and an injection volume of 5 μL, whereas Supplementary Note 7 reports 20 mL min^{-1} and 20 μL. These cannot both be correct, and 20 mL min^{-1} is implausible for a conventional LC–MS setup. There are additional inconsistencies, including C 1s calibration at 284.7 eV versus 284.8 eV, a very broad calcination temperature range (1000–1100 °C) without clear clarification, and a negative “raw material input” value in Supplementary Table 1. These issues must be corrected thoroughly.
9. The collection efficiency (N) should be experimentally determined and clearly described. The methodology and calibration procedures should be explicitly provided. Relevant literature (e.g., DOI: 10.1002/adma.202521237) offers detailed guidance and should be considered.
10. The manuscript and Supporting Information require careful proofreading for language consistency and technical accuracy. Several non-idiomatic expressions and typographical errors. For example: “Koutechy-Levich” in Supplementary Fig. 14 should be corrected to “Koutecky-Levich.” The heading “Practical Decontamination Performance and System Engineering. Actual Decontamination Performance and System Engineering.” contains an obvious duplication. UV-UV spectrophotometry” should likely be “UV–vis spectrophotometry.” Subscripts and superscripts in the $^1O_2$ quantification equations should be carefully checked.

Reviewer #2

(Remarks to the Author)
Wang et al. address the severe kinetic and thermodynamic limitations associated with the conventional stepwise route, in which H2O2 is first generated via a two-electron oxygen reduction reaction (2e– ORR) and then further activated across different sites to produce •OH. In this manuscript, the authors propose an atomically defined, nanoscale perovskite active domain, Pr1.0Sr1.0Fe0.5Zn0.25Mo0.25O4-δ (PSFZM), that enables the direct, formal conversion of O2 to •OH through a 3e– ORR pathway, with the key feature being the confinement of intermediate stabilization and O–O bond cleavage within the same lattice-localized motif. The catalytic performance reported in the manuscript is also impressive, including a Faradaic efficiency (FE) toward •OH of up to 28.7 % in half-cell tests, an O2-to-ROS conversion efficiency of 37.7 %, and a remarkably high •OH production rate. In addition, the work presents a flow reactor that directly uses gaseous oxygen instead of dissolved oxygen, further demonstrating the practical potential of this system for electrode-level scale-up and application. This is an interesting study with clear timeliness and a notable degree of originality. By constructing a crystalline framework featuring single-site active domains, the authors aim to circumvent the limitations of the currently prevalent tandem pathway. However, the experimental evidence provided by the combination of multiple characterization techniques is still insufficient to offer strong support for the proposed mechanistic role of the active domain, and several details require further clarification or additional evidence. Overall, I believe that after revision, this manuscript could be suitable for publication in Nature Communications.

1.The current experimental results in the manuscript are more indicative of “suppressed H2O2 accumulation and its facile subsequent conversion on PSFZM”, but they do not strictly demonstrate that this process occurs within the same localized active domain, nor do they rigorously exclude short-range desorption/re-adsorption or interfacial secondary activation. The authors are encouraged to provide isotopic or transient spectroscopic evidence to verify that the local conversion rate of *H2O2 is faster than its desorption and diffusion.
2.The manuscript lacks EXAFS-derived coordination information for PSFZM, PSFZ, and PSFM, including detailed metal–O bond lengths. It is recommended to summarize these parameters in a table.
3.The authors report n ≈ 3.0 for PSFZM in the potential range of 0.20–0.60 V and claim proximity to the theoretical diffusion-limited current for the $3e^-$ ORR. However, the Methods section indicates that RRDE data were not iR-compensated, and the expression of FE is itself unclear. The authors should clarify the calibration of collection efficiency, and provide error bars for n and H2O2 selectivity.
4.The manuscript states that 86.7 % of consumed O2 is converted to •OH, whereas the O2-to-ROS conversion efficiency is only 37.7 %. This discrepancy is confusing and requires a clear definition of the denominator used in each metric.
5.The claim of a “near-theoretical-rate” is insufficiently supported. The title directly asserts proximity to the theoretical rate limit, whereas the main text only states that the current at 0.07–0.10 V is “approaching the theoretical diffusion-limitation current for $3e^-$ ORR,” without providing explicit calculations, boundary conditions, or error analysis for the theoretical limit.
6.The performance comparison in the manuscript uses inconsistent normalization standards for statements such as “highest reported” and “nearly 20 times higher than previously reported systems.” Multiple normalization metrics are employed (e.g., 821 μmol h−1, 228.06 μmol L−1 min−1, 28.8 mol g_cat−1 h−1). A unified comparison basis should be adopted in both the main text and Supplementary Information to avoid distortion in cross-literature comparisons.
7.The manuscript first states that XRD confirms the retention of a Ruddlesden–Popper layered structure, but subsequently claims that TEM reveals an inverse perovskite configuration. This appears inconsistent and requires clarification.
8.Although the device-level performance is impressive, the contributions of cathodic $3e^-$ ORR and anodic WOR in the full cell have not been clearly deconvoluted. The authors should quantitatively distinguish the •OH contributions from the

cathode and anode, and clearly define the denominator used in calculating the reported 64.7% FE.
9.In the Methods section, the FE formula for the RRDE analysis is not written in a sufficiently standard form. The authors are advised to recheck the expression and clearly define the charge meaning represented by the numerator and denominator.
10.There is a repeated subsection title in the Results section: “Practical Decontamination Performance and System Engineering. Actual Decontamination Performance and System Engineering.” One of them should be removed.
11.The correspondence between figure panels and the main text is not sufficiently rigorous in several places. For example, in Fig. 4, the panel indices are cross-referenced inconsistently in the main text as 4g, 4i and 4i, 4j. A thorough check of the figure–text correspondence is recommended.
12.There are numbering errors in the caption of Fig. 4. The caption first states “g Mo K-edge ... h Mo K-edge ... k WT-EXAFS ...”, but later also states “g Fe K-edge ... h Fe K-edge ... k WT-EXAFS ...”. The authors should carefully correct these errors.
13.The phrase “classical spinning method” in the caption of Fig. 2 is not sufficiently precise. The Methods section describes the synthesis as electrospinning, whereas the figure caption uses “classical spinning method.” The terminology should be unified as electrospinning.
14.In Section 6 of the manuscript, the expressions “membrane-free cylindrical electrolyzer” and later “membraneless flow-through electrolyzer” are inconsistent. This should be carefully corrected.
15.The phrase “dominating the continuous evolution of O2 at this site” is not accurate in English. What is intended here should be O2 activation/conversion rather than “evolution of O2,” since in electrocatalysis “evolution” is usually understood as O2 evolution.

Reviewer #3

(Remarks to the Author)
This manuscript reports a single-atom engineered perovskite catalyst (PSFZM) that is claimed to promote an efficient formal 3-electron oxygen reduction reaction ($3e^-$ ORR), enabling direct and selective electrogeneration of hydroxyl radicals (•OH). The central design concept is an atomically confined active domain with a Zn-O-Mo structural motif, which is proposed to stabilize key oxygenated intermediates and facilitate O-O bond cleavage, thereby bypassing the kinetic constraints of conventional stepwise pathways. The authors further report high •OH production, favorable oxygen utilization, effective degradation of organic pollutants, and compatibility with a membrane-free flow electrolyzer for sustained water treatment. However, the current evidence base contains multiple critical gaps in mechanistic proof, incomplete electrochemical quantification, and insufficient controls, so the work requires major revision.
1. The absorption spectrum does not present the fitting results; instead, it should be reported in a table including the coordination number, bond length, and correction factors for different samples. Therefore, the Zn-O-Fe and Zn-O-Mo coordination environments inferred from the wavelet transform remain questionable, as atom-level evidence is insufficient. Moreover, in the in situ XPS data, the Mo 3d, Mo K-edge, and Fe K-edge absorption edges indicate that the PSFZ, PSFM, and PSFZM catalysts are essentially indistinguishable. What, then, is the specific role of Fe and Mo? In a comparison between PAFZ and PSFZM, one would expect the Mo effect to be more clearly elucidated.
2. The authors throughout the manuscript assign Zn as the adsorption site and Mo as the polarizing agent that facilitates O-O bond cleavage; however, no direct evidence is provided to support the proposed role of Mo. Moreover, Fe is also shown by the aberration-corrected TEM results to be arranged in an ordered single-atomic configuration, yet its role is not discussed. In contrast, Mo does not appear to form an ordered single-atomic structure. Therefore, the “single-atom engineered” Zn-O-Mo active sites mentioned in the manuscript are ambiguous and require supporting evidence and clarification.
3. In situ FTIR spectroscopy indicates the presence of the *OOH and *H2O2 intermediates; however, it does not directly capture O-O bond cleavage or the formation of •OH. Therefore, the claim of “continuous unit-site conversion” is not substantiated. Moreover, during the in situ reaction, PSFZM and PSFZ exhibit nearly identical adsorption evolution of the intermediates. How, then, can the latter be inferred to continuously accumulate H2O2? As such, the results remain inconclusive.
4. The DFT results support this proposed mechanism; however, the treatment lacks rigor and depth. Only the Gibbs free energy is reported, while key information-such as transition states, energy barriers, reaction kinetics, and potential-dependent free-energy profiles-is missing. A complete transition-state search, kinetic barrier analysis, and a consistent mechanistic interpretation for •OH and 1O2 are therefore required. Moreover, the manuscript claims that PSFZM follows a 3e- pathway rather than the conventional 2e- + 1e- route. While the authors attempt to exclude the 2e- pathway through characterization and mechanistic or DFT analyses, they do not rule out a single-electron pathway; this should be explicitly demonstrated. Otherwise, one can only conclude that the 2e- pathway is unlikely, rather than excluding the conventional 2e- + 1e- mechanism.
5. As stated in Fig. 5c and earlier discussions, PSFZ follows a 2e- pathway, PSFM a 4e- pathway, and PSFZM a 3e- pathway. However, Fig. 5c indicates that PSFM also follows a 3e- pathway, which is inconsistent with both the figure caption and the preceding text. The authors should clarify this discrepancy.
6. As central conclusion of the manuscript, the proposed 3e- oxygen-reduction pathway is not sufficiently supported by rigorous and complete data. The estimated electron-transfer number $n \approx 3.0$ is valid only within a limited potential window, without an n-versus-potential profile over the full potential range. The RRDE analysis does not correct for background currents and capacitive (pseudocapacitive) currents; moreover, the Koutecky-Levich treatment lacks adequate data points to substantiate a stable 3e- reaction mechanism. In addition, the authors have not provided a complete potential-dependent selectivity curve for H2O2.
7. Some figures and tables contain unclear labeling (e.g., the symbol “L” in Fig. 4L is obscured), and the labeling or order of figures is used arbitrarily. The figure sequence should be reorganized to ensure a coherent logical flow. In addition, the performance literature comparisons are selective, relying on comparatively inferior catalysts rather than the most recent reports. Units should be kept consistent throughout. Overall, all figures, data presentations, and scientific terminology must be carefully revised.

Version 1:

Reviewer comments:

Reviewer #1

(Remarks to the Author)
The revised manuscript has well addressed my concerns and comments in the last revision and the paper is now recommended for acceptance in Nat Commun

Reviewer #2

(Remarks to the Author)
The paper has been well improved according to the comments from the reviewers, thus I would recommend accepting it for publication.

Reviewer #3

(Remarks to the Author)
The paper could be accepted.

Open Access This Peer Review File is licensed under a Creative Commons Attribution 4.0 International License, which permits use, sharing, adaptation, distribution and reproduction in any medium or format, as long as you give appropriate credit to the original author(s) and the source, provide a link to the Creative Commons license, and indicate if changes were made.
In cases where reviewers are anonymous, credit should be given to 'Anonymous Referee' and the source.
The images or other third party material in this Peer Review File are included in the article's Creative Commons license, unless indicated otherwise in a credit line to the material. If material is not included in the article's Creative Commons license and your intended use is not permitted by statutory regulation or exceeds the permitted use, you will need to obtain permission directly from the copyright holder.
To view a copy of this license, visit https://creativecommons.org/licenses/by/4.0/

Response to Reviewers (Manuscript ID: NCOMMS-26-025297-T)

"Single-atom-engineered perovskite enables near-theoretical-rate hydroxyl radical electrogeneration"

–

Referee: 1

Comments to the Author

This manuscript reports a Zn–Mo co-engineered Ruddlesden–Popper perovskite, $Pr_{1.0}Sr_{1.0}Fe_{0.5}Zn_{0.25}Mo_{0.25}O_{4-\delta}$ (PSFZM), for hydroxyl radical electrogeneration through a proposed single-site active-domain mechanism. The authors combine structural characterization, RRDE, operando/intermediate spectroscopy, and DFT calculations, and further extend the catalyst to a membraneless flow electrolyzer. The topic is timely and potentially important for electrochemical advanced oxidation and selective oxygen activation. The concept of coupling intermediate stabilization and O-O bond activation within one lattice-confined active domain is interesting. However, in its current form, I do not believe the manuscript yet meets the standard for publication in Nature Communications. The central claims regarding a direct $3e^-$ ORR pathway to •OH and a single-atom-engineered active domain are not supported by sufficiently compelling evidence. In addition, there is insufficient rigor in radical quantification and Faradaic efficiency analysis, together with multiple inconsistencies in the Methods and Supporting Information. Addressing these issues would substantially improve the overall quality, clarity, and robustness of the manuscript.

We sincerely thank the reviewer for the careful evaluation and constructive comments. We appreciate the reviewer's recognition that the topic is timely and significant for electrochemical advanced oxidation and selective oxygen activation. We also fully acknowledge the reviewer's concerns regarding the mechanistic rigor, radical quantification, Faradaic efficiency analysis, and consistency of the manuscript. In response to these

comments, we have substantially revised the manuscript to improve its scientific rigor, clarity, and overall robustness. We believe these substantial revisions have significantly strengthened the manuscript and improved the rigor of the mechanistic interpretation, quantitative analysis, and structural evidence. We sincerely appreciate the reviewer's insightful comments, which greatly helped improve the quality and clarity of this work.

1. The manuscript presents the system as bypassing the conventional decoupled $2e^-$ ORR + $1e^-$ H_2O_2 activation pathway. However, the authors' own operando FTIR discussion describes a sequential O_2→*OOH→*H_2O_2→•OH evolution, and the H_2O_2-addition experiment is interpreted to show that surface-adsorbed H_2O_2 is the immediate precursor to •OH. This evidence is more consistent with a confined tandem mechanism within one local domain than with a rigorously direct $3e^-$ pathway. The current wording therefore appears stronger than the data support. The authors should either substantially strengthen the mechanistic evidence or moderate the central claim throughout the manuscript.

Response: Thank you for your valuable comments. We have carefully checked your concerns. We have carefully reviewed the concerns you raised and have incorporated new evidence to clarify that PSFZM follows a surface-confined, continuous $3e^-$ ORR pathway, rather than a spatially decoupled "$2e^-$ ORR + $1e^-$ H_2O_2 activation" pathway mediated by the desorption and diffusion of H_2O_2.

In our revised description, the formation of *OOH and surface-adsorbed *H_2O_2 is entirely consistent with the proposed mechanism. The key distinction lies in whether *H_2O_2 accumulates/desorbs as a free intermediate—subsequently undergoing activation at different sites—or remains confined within the same local active region and is immediately converted into •OH. To elucidate this point, we have supplemented our findings with new in situ FTIR and isotope-resolved differential electrochemical mass spectrometry (DEMS) evidence.

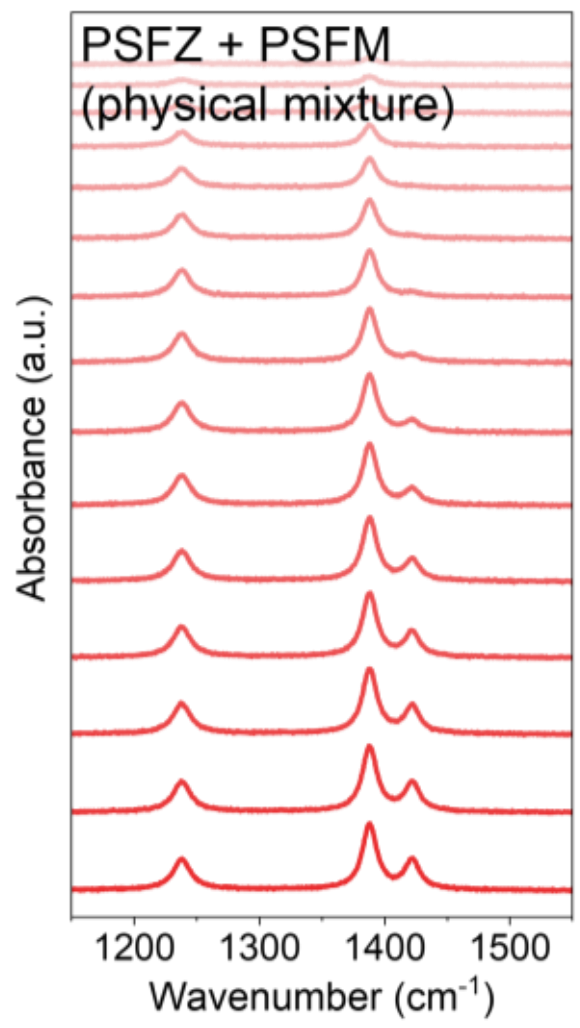

Supplementary Fig. 28 The ORR electrochemical in-situ FTIR spectra of PSFZ + PSFM physical mixture catalyst collected in an incremental voltage configuration.

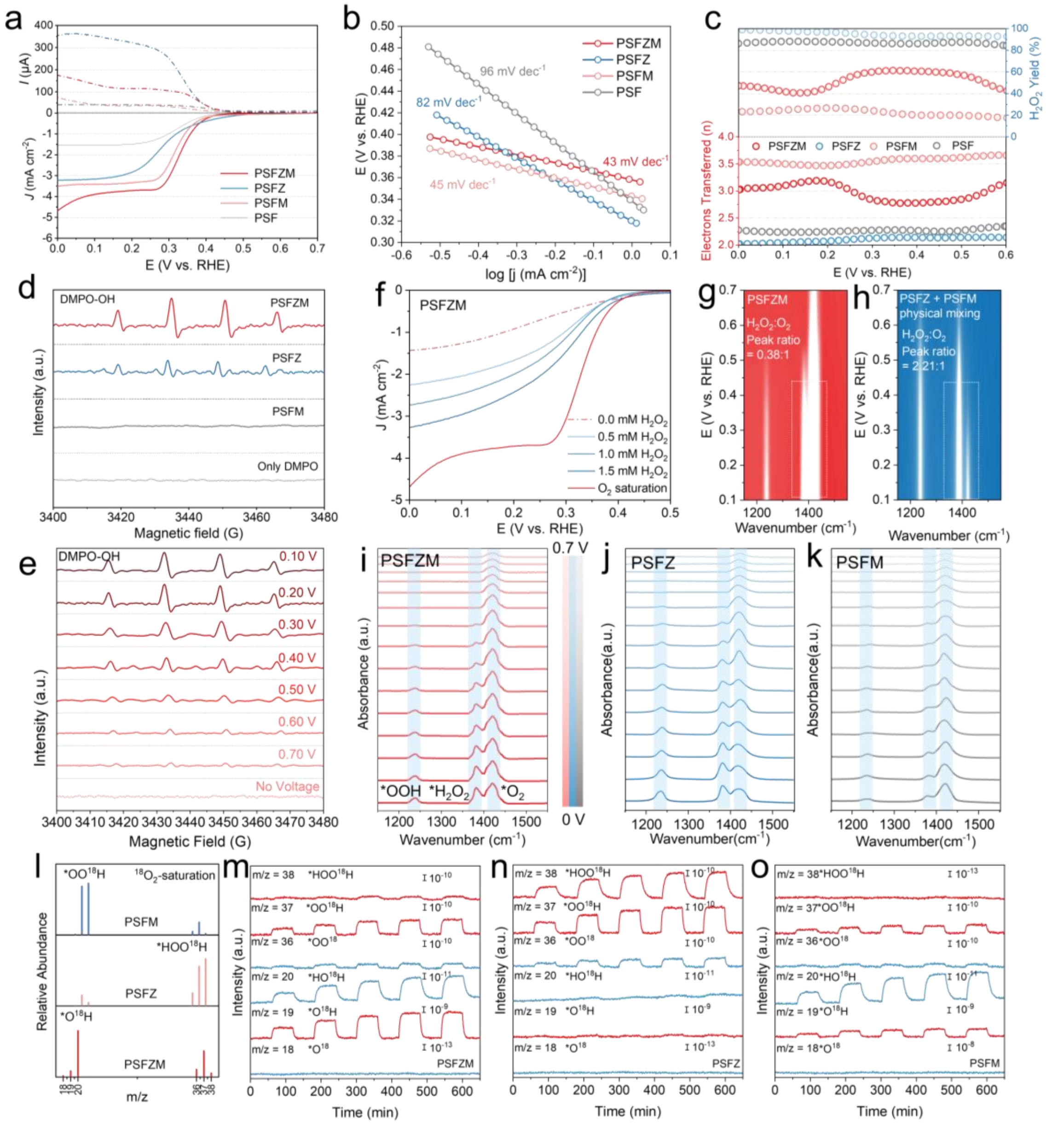

Fig. 3 Qualitative analysis of ORR selectivity and O_2 conversion to •OH. **a** RRDE polarization profiles for oxygen reduction reaction. Note: The solid lines are the disk current density corresponding to oxygen reduction and the dotted lines are the ring current density corresponding to H_2O_2 oxidation. **b** The corresponding Tafel plots. **c** H_2O_2 selectivity and number of transferred electrons (n) calculated from RRDE polarization curves. **d** EPR spectra of DMPO-•OH adducts for samples from different catalyst-catalyzed electro-Fenton reactions. **e** DMPO-•OH adduct EPR spectra of ORR electrochemistry over PSFZM catalyst collected in an incremental voltage configuration. **f** LSV with different concentrations of

H_2O_2 raw materials in the O_2-free configuration and the O_2-filled configuration. Presented are the potential-dependent in situ FTIR contour plots of **g** PSFZM and the **h** PSFZ+PSFM physical mixture, acquired in an O_2-saturated electrolyte. The ORR electrochemical in-situ FTIR spectra of **i** PSFZM, **j** PSFZ, and **k** PSFM catalyst collected in an incremental voltage configuration. **l** Representative mass spectra of PSFZM, PSFZ, and PSFM, recorded under conditions saturated with $^{18}O_2$, reveal distinct ^{18}O-labeled oxygen reduction products. Time-resolved DEMS traces of selected isotope-labeled fragments, including peroxide/hydroperoxide-related channels and hydroxyl/water-related channels, during intermittent electrolysis over **m** PSFZM, **n** PSFZ, and **o** PSFM.

Manuscript Revision:

To further substantiate this mechanism, in-situ Fourier-transform infrared spectroscopy (FT-IR) was used to clarify the evolution of reaction intermediates **(Figs. 3g–3k**). With increasing cathodic bias, the *OOH and *H_2O_2 signals on PSFZ were markedly enhanced, indicating continuous H_2O_2 accumulation without subsequent activation (**Fig. 3j**). By contrast, PSFM exhibited only a weak *OOH signal and almost no detectable H_2O_2 feature, consistent with a four-electron (4e$^-$) ORR pathway (**Fig. 3k**). In sharp contrast, PSFZM displayed an O-O stretching band of adsorbed O_2 at ~1424 cm^{-1}; as the potential shifted negatively, characteristic *OOH-related bands subsequently emerged at ~1241 and ~1389 cm^{-1} (**Fig. 3i**). Notably, the *OOH and *H_2O_2 species reached a dynamic formation–consumption equilibrium near 0.2 V. To more clearly distinguish continuous ORR evolution from spatially separated tandem evolution, PSFZM was compared with a physical mixture of PSFZ and PSFM. For PSFZM, the relative intensity ratio of the *H_2O_2-related band to the O_2/*OOH-related band was only 0.38:1, indicating that peroxide did not accumulate as the dominant surface species (**Fig. 3g**). The nearly six-fold enhancement of the peroxide-related signal in the PSFZ+PSFM physical mixture indicates substantial H_2O_2 accumulation, consistent with a conventional decoupled 2e$^-$ + 1e$^-$ tandem process (**Fig. 3h, Supplementary Fig. 28**). These results strongly support a confined active-domain-mediated continuous conversion pathway of O_2 → *OOH → *H_2O_2 → •OH, in which the B-site Zn centers serve as the key sites for direct O_2 activation during the 3e$^-$ ORR process.

To resolve the operative ORR pathway on PSFZM, we performed isotope-resolved

DEMS in $^{18}O_2$-saturated electrolyte. The relative mass spectral abundance distribution (**Fig. 3l**) shows that PSFM exhibits strong responses only at m/z = 18-20, corresponding to a typical $4e^-$ ORR pathway. In contrast, PSFZ displays synchronized signals at m/z = 37 and 38, while remaining silent at m/z = 19 and 20, indicating the direct desorption of *OOH as H_2O_2. Time-resolved DEMS measurements (**Figs. 3m-o**) further reveal that PSFZM exhibits synchronized and reversible responses across all four channels (m/z = 37, 38, 19, and 20) during each potential step. Compared with PSFZ, the H_2O_2 signal (m/z = 38) is markedly suppressed, whereas the *OH/ H_2O signals (m/z = 19/20) are simultaneously enhanced. In a conventional tandem mechanism involving H_2O_2 desorption–diffusion–re-adsorption, the m/z = 38 signal must precede the m/z = 19/20 signals and exhibit distinct relaxation times. Therefore, the strictly synchronized responses observed for PSFZM can only originate from the continuous reduction of surface-confined *H_2O_2 intermediates at the same active site.

2. The XANES/EXAFS results suggest a Zn–O–Mo local coordination environment, and microscopy indicates a porous nanofiber morphology with elemental dispersion. However, the current evidence does not fully justify the strength of the term "single-atom-engineered" or the claim that Zn atoms are periodically enriched within a distorted tetragonal unit cell. More statistically robust atom-resolved STEM analysis, detailed EXAFS fitting parameters with model comparisons, and additional structural characterization (e.g., Rietveld refinement or pair distribution functon (PDF) analysis) are needed. These would help to rigorously establish the proposed local structure and exclude alternative explanations such as nanoscale segregation or short-range disorder.

Response: Thank you for your valuable comments. We have carefully checked your concerns. We performed statistically robust atom-resolved AC-HAADF-STEM analysis over multiple independent regions. More than 500 B-site atomic columns were analysed to quantify the spatial distribution of abnormal-contrast B-site columns assigned to Zn/Mo-containing local motifs. The statistical analysis shows that this distribution deviates significantly from a random occupation model, as confirmed by the χ^2 test. In addition, the radial distribution function of the abnormal-contrast B-site columns decays toward the random limit at long distances, indicating that the Zn/Mo-related enrichment is local rather than associated with long-range phase separation.

Second, we revised and expanded the EXAFS analysis by including detailed fitting

parameters and model comparisons. In the revised Supplementary Information, we provide the fitting models, coordination numbers, bond lengths, Debye–Waller factors, ΔE_0 values and R factors for the Zn K-edge and Mo K-edge EXAFS spectra. Importantly, fitting of the Zn K-edge requires not only the first-shell Zn–O contribution, but also a higher-shell Zn–O–Mo scattering path to reproduce the experimental R-space features. Control models excluding the Zn–O–Mo contribution, or replacing it solely with Zn–O–Fe/Zn–O–Zn paths, show poorer fitting quality and larger residuals. The Mo K-edge EXAFS analysis provides a consistent local coordination picture, supporting the presence of Mo-containing neighbouring environments around the Zn-centred region. These model-comparison results further strengthen the assignment of the Zn–O–Mo local coordination motif.

We performed Rietveld refinement of the powder X-ray diffraction patterns of PSFZ, PSFM and PSFZM. All three samples can be refined within the tetragonal Ruddlesden–Popper structure with the I4/mmm space group, with no detectable impurity phases. Compared with the singly doped PSFZ and PSFM samples, PSFZM exhibits lattice expansion and more pronounced B–O bond distortion, consistent with the formation of a mixed-metal B-site environment rather than separated oxide phases. The absence of additional diffraction peaks, together with the refined lattice parameters and oxygen-site occupancies, indicates the absence of crystalline Zn-rich or Mo-rich secondary phases.

We further introduced pair distribution function analysis to probe the local structure beyond the average crystallographic model. The PDF results show no additional short-range correlations attributable to ZnO, MoO_x or other segregated nanophases. Instead, the local pair correlations are consistent with a distorted Ruddlesden–Popper framework containing mixed B-site coordination environments. This result supports the EXAFS-derived local Zn–O–Mo structural model and helps exclude nanoscale segregation or amorphous short-range clustering as alternative explanations.

Taken together, the newly added AC-HAADF-STEM statistical analysis, EXAFS model comparison, Rietveld refinement and PDF characterization provide mutually consistent evidence for the proposed Zn–O–Mo local coordination environment in PSFZM, while ruling out major contributions from nanoscale segregation, crystalline impurity phases or simple random short-range disorder.

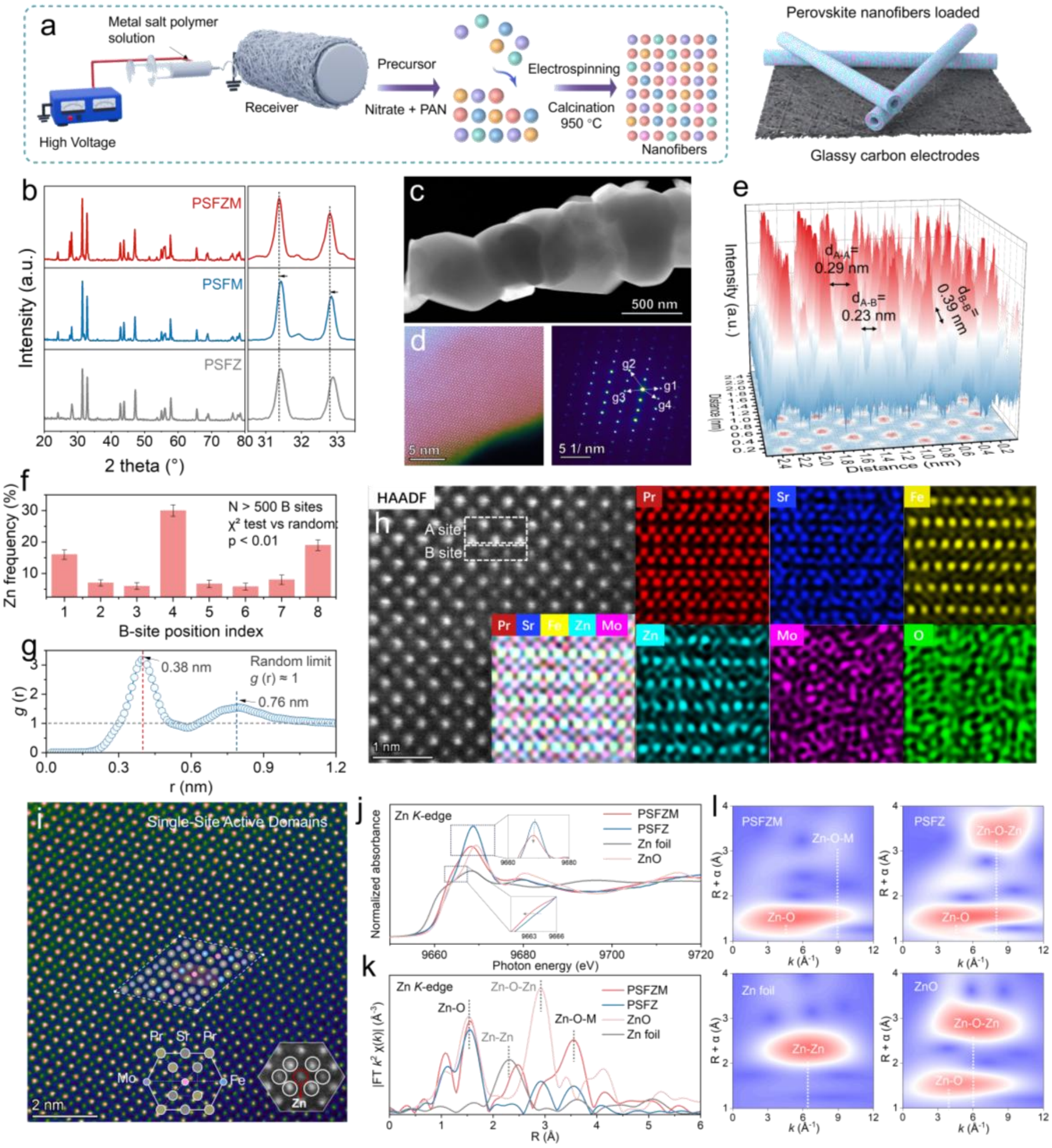

Fig. 2 Atomic structure characterization of the active domains for PSFZM. a Schematic diagram showing the preparation of PSFZM electrodes by the electrospinning method. Colors represent: Pr (red), Sr (purple), Fe (yellow), Zn (green), Mo (blue). **b** XRD patterns of PSFZM, PSFZ and PSFM. **c** SEM image of PSFZM. **d** HAADF-STEM image. The inset shows the corresponding selected area electron diffraction (SAED) pattern of PSFZM. **e** Atomic spacing diagram based on HAADF-STEM. **f** Periodicity analysis of Zn occupation on the B-site sublattice. **g** Radial distribution function g(r) of Zn-rich B-site columns. **h** HAADF-STEM image and the corresponding elemental maps for PSFZM. **i** The elemental intensity maps obtained from HAADF-STEM conversion show that elements with larger ionic radii are A-site elements, while those with smaller ionic radii are B-site elements. **j** Zn K-edge XANES spectra of PSFZM, PSFZ, Zn foil, ZnO. **k** Zn K-edge k^3-weighted FT-EXAFS spectra and **l** WT-EXAFS spectra of PSFZM, PSFZ, Zn foil, ZnO.

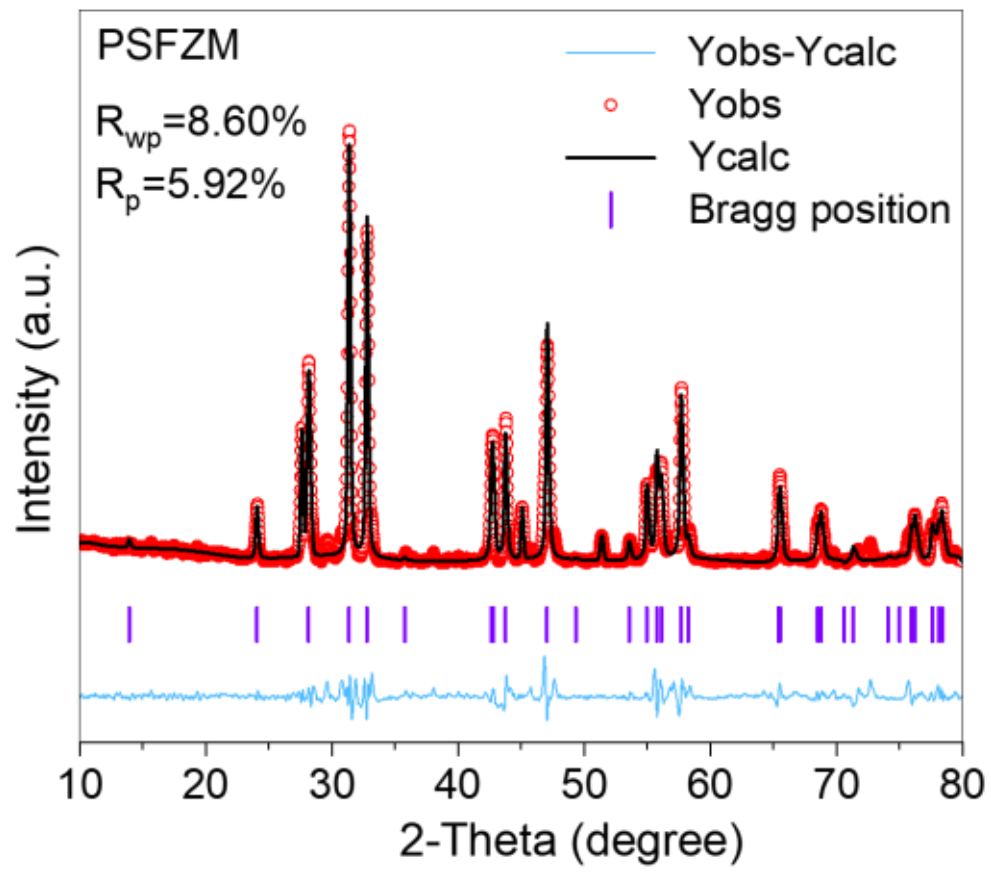

Supplementary Fig. 2. XRD Rietveld refinement of PSFZM.

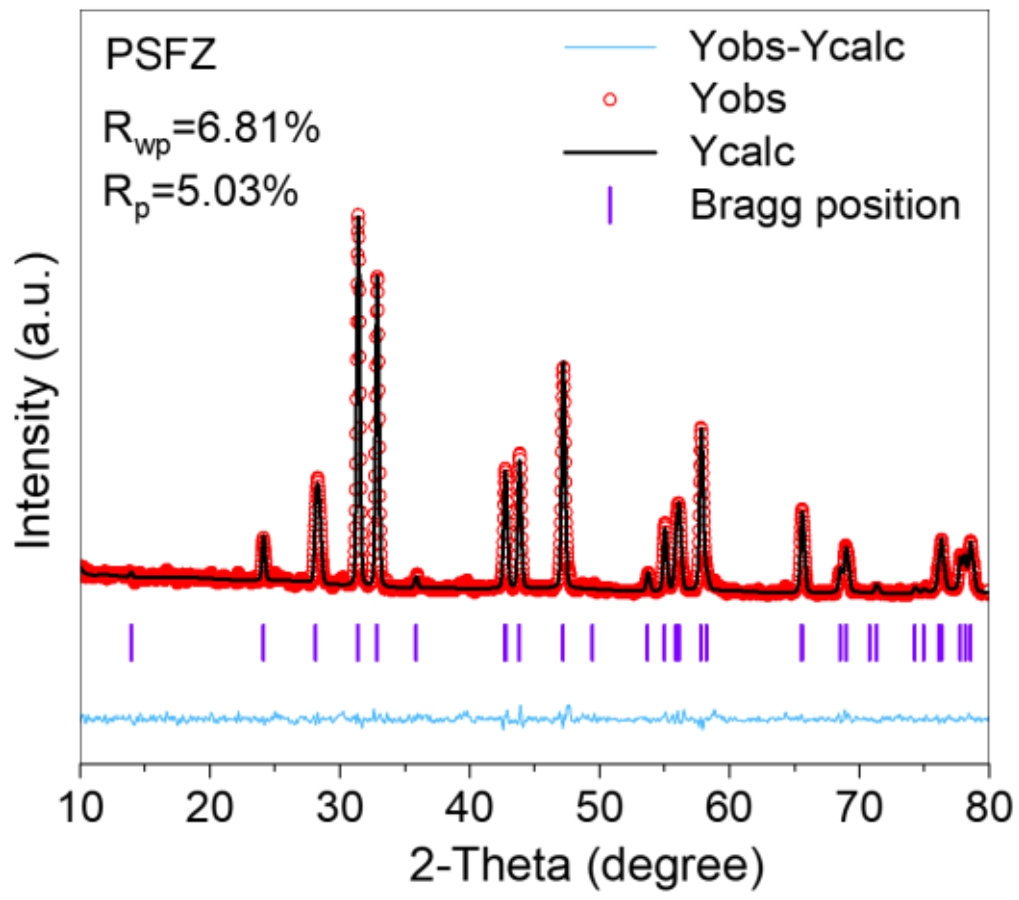

Supplementary Fig. 3. XRD Rietveld refinement of PSFZ.

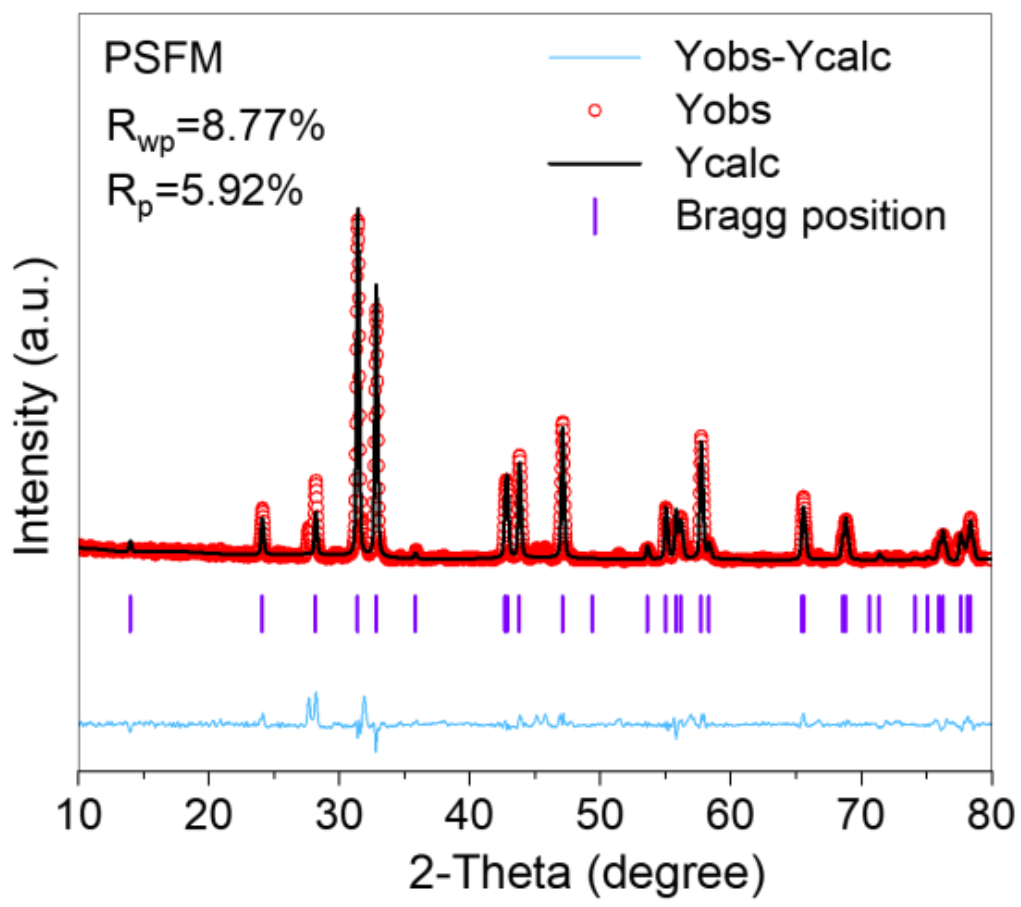

Supplementary Fig. 4. XRD Rietveld refinement of PSFM.

Supplementary Table 1. Rietveld-refined crystallographic parameters of PSFZ, PSFM and PSFZM.

**Parameter**	**PSFZ**	**PSFM**	**PSFZM**
**Nominal composition**	$PrSrFe_{0.75}Zn_{0.25}O_{4-\delta}$	$PrSrFe_{0.75}Mo_{0.25}O_{4-\delta}$	$PrSrFe_{0.5}Zn_{0.25}Mo_{0.25}O_{4-\delta}$
**Space group**	I4/mmm	I4/mmm	I4/mmm
**Crystal system**	Tetragonal	Tetragonal	Tetragonal
**a = b / Å**	3.848739	3.855880	3.858459
**c / Å**	12.670967	12.651975	12.673535
**V / Å^3**	187.692	188.107	188.680
**c/a**	3.292	3.281	3.285
**B-site occupation**	$Fe_{0.75}Zn_{0.25}$	$Fe_{0.75}Mo_{0.25}$	$Fe_{0.5}Zn_{0.25}Mo_{0.25}$
**O1 occupancy**	0.772	0.780	0.560
**O2 occupancy**	0.986	0.975	0.976
**B–O$_{eq}$ / Å**	1.924	1.928	1.929
**B–O$_{ap}$ / Å**	1.995	1.990	2.058
**B–O$_{ap}$/B–O$_{eq}$**	1.037	1.032	1.067
**Rwp / %**	6.81	8.77	8.60
**Rp / %**	5.03	5.92	5.92
**RF / %**	4.03	5.72	4.92
**RF^2 / %**	6.70	13.18	8.05

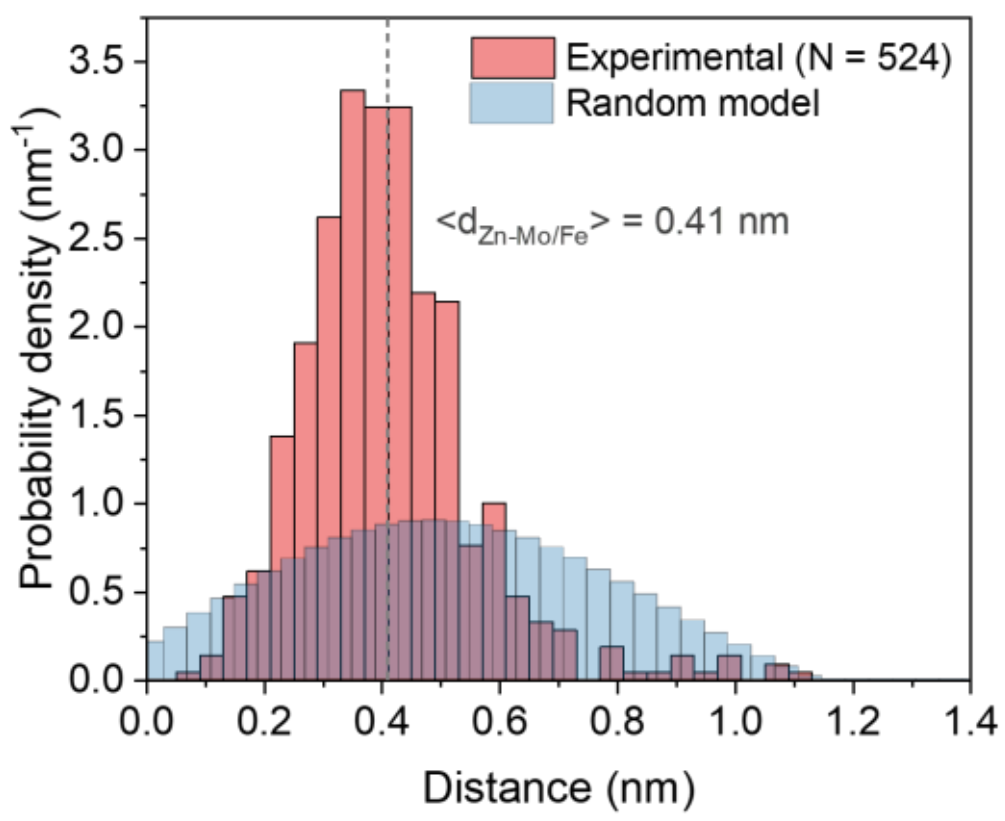

Supplementary Fig. 8 Statistical distribution of Zn–Mo/Fe neighbouring distances in PSFZM.

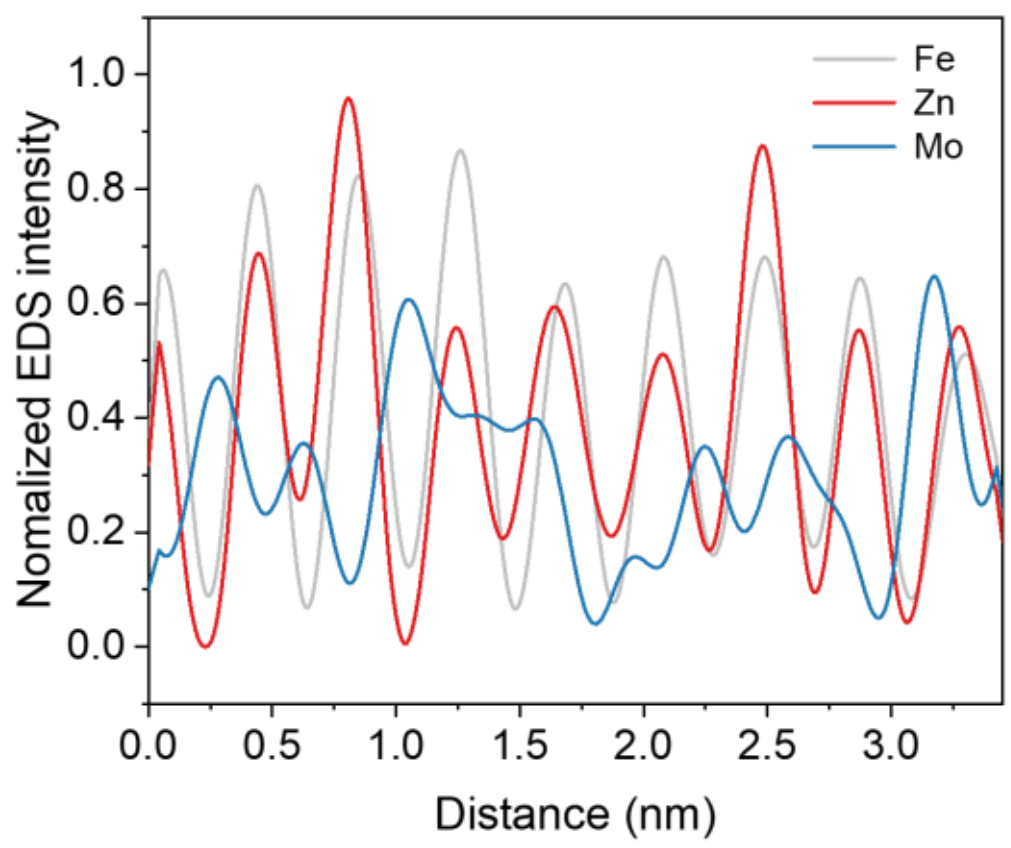

Supplementary Fig. 9 STEM-EDS line-scan analysis of Fe, Zn and Mo distributions in PSFZM.

Manuscript Revision:

Rietveld-refined X-ray Diffraction (XRD) patterns confirm that PSFM, PSFZ, and PSFZM retain the tetragonal Ruddlesden-Popper structure with the I4/mmm space group (**Supplementary Figs. 2-4, Supplementary Table 1**). PSFZM exhibits the largest unit-cell volume and the most pronounced elongation of axial B–O bonds, accompanied by left-shifted diffraction peaks at ~32° and ~34° (**Fig. 2b**). These crystallographic features demonstrate that Zn/Mo co-doping induces lattice expansion and BO_6 octahedral distortion, supporting the formation of mixed-metal B–O active domains distinct from the single-metal local environments. Field-emission scanning electron microscopy (FE-SEM) and high-angle

annular dark-field scanning transmission electron microscopy (HAADF-STEM) images reveal that PSFZM forms a highly crystalline porous nanofiber network structure (**Fig. 2c and Supplementary Figs. 5-7c**). Aberration-corrected high-resolution transmission electron microscopy (AC-HRTEM) images (**Fig. 2d**) together with the atomic spacing map (**Fig. 2e**) further disclose an orthogonally distorted tetragonal lattice within PSFZM. Quantitative statistical analysis of more than 500 B-site atomic columns indicates that the distribution of Zn sites in PSFZM is non-random (**Supplementary Figs. 8 and 9**), showing a significant deviation from the random occupancy model according to the χ^2 test ($p < 0.01$; **Fig. 2f**). The radial distribution function decays toward the random-limit value of $g(r) \approx 1$ (**Fig. 2g**), indicating the presence of a localized, zinc-enriched ordered structure. Atomic-scale elemental mapping and STEM-energy dispersive X-ray spectroscopy (EDS) line-scan analysis further confirms this conclusion, showing that Pr and Sr atoms are uniformly distributed at the A sites, whereas Zn atoms are periodically enriched at the B sites near the center of the distorted tetragonal unit cells (**Figs. 2h, 2i and Supplementary Figs. 7d, 10-11**). Collectively, these results provide compelling evidence for the formation of Zn-centered active-domain units.

3. The XAFS analysis is not fully convincing. In particular, the assignment of the PSFZM absorption edge (lines 103–105) is not clearly supported by the presented spectra. Reference 33 does not appear to substantiate the conclusion of Zn–O–Mo coordination, and more direct coordination evidence is required. For the proposed single-site active domain, Mo K-edge EXAFS in R-space should also show features corresponding to Mo–O–Zn coordination. The assignment of Zn–O–Fe coordination appearing beyond ~3 Å is questionable. Furthermore, there are inconsistencies between Supplementary Fig. 7 and Fig. 2i. The authors should carefully re-examine the data, provide a more rigorous analysis of coordination environments, and include detailed fitting models and parameters.

Response: We thank the reviewer for this careful and important comment. We agree that the original XAFS discussion was not sufficiently rigorous to fully support the proposed Zn–O–Mo local coordination environment. In particular, the previous assignment of the absorption-edge features and the higher-shell EXAFS peaks was not described with sufficient caution, and the citation of Ref. 33 alone could not serve as direct evidence for Zn–O–Mo coordination. We have therefore re-examined the XANES/EXAFS data, revised

the structural assignment, added Mo K-edge evidence, and provided detailed fitting models and parameters in the revised Supplementary Information.

First, we have revised the XANES discussion in lines 103–105. In the original manuscript, the absorption-edge shift of PSFZM was overinterpreted as direct evidence of Zn–O–Mo coordination. We now clarify that the Zn K-edge XANES only indicates a modified electronic structure and local coordination environment around Zn relative to the references, but does not by itself prove a specific Zn–O–Mo linkage. The conclusion of Zn–O–Mo local coordination is now based on the combined EXAFS fitting, wavelet-transform analysis and model comparison, rather than on the XANES edge position alone. We have revised the assignment of the feature beyond ~3 Å in the Zn K-edge EXAFS spectrum. We agree that assigning this region solely to Zn–O–Fe coordination is not sufficiently justified, because higher-shell EXAFS features in this range can contain overlapping contributions from multiple scattering and mixed B-site cation paths. We have therefore removed the overly specific Zn–O–Fe assignment and now describe this feature more cautiously as a higher-shell contribution associated with the mixed B-site coordination environment. The Zn–O–Mo contribution is discussed only when supported by fitting and model-comparison results. Finally, we have carefully checked the inconsistency between Supplementary Fig. 7 and Fig. 2i. The discrepancy originated from differences in data processing and plotting parameters, including the k-range, k-weighting and Fourier-transform window used in the two figures. In the revised manuscript, all EXAFS spectra have been reprocessed using consistent parameters, and the corresponding figures have been replaced. The labels, fitting curves and coordination assignments in Fig. 2i and Supplementary Fig. 7 are now fully consistent.

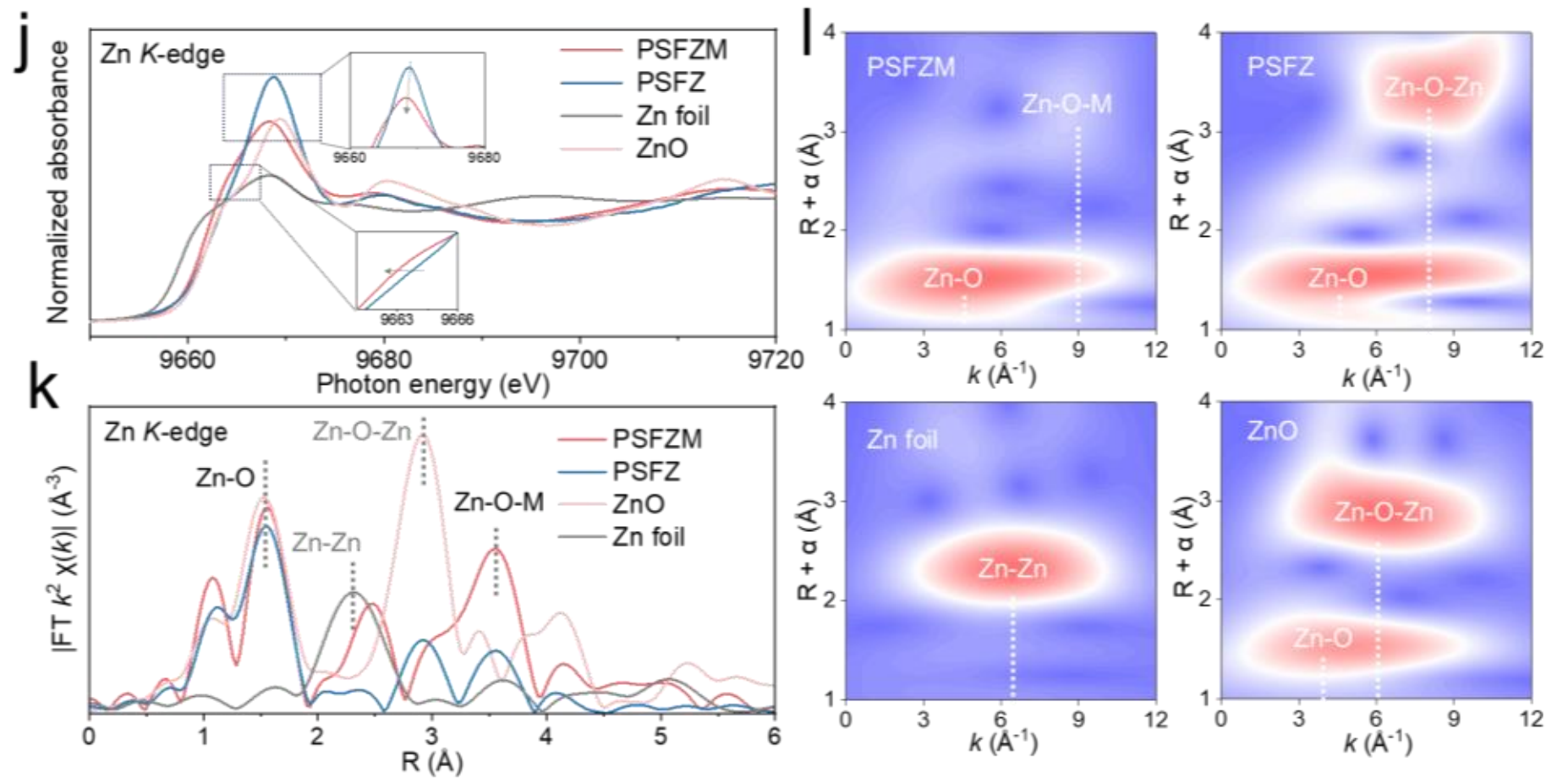

j Zn K-edge XANES spectra of PSFZM, PSFZ, Zn foil, ZnO. **k** Zn K-edge k^3-weighted FT-EXAFS spectra and **l** WT-EXAFS spectra of PSFZM, PSFZ, Zn foil, ZnO.

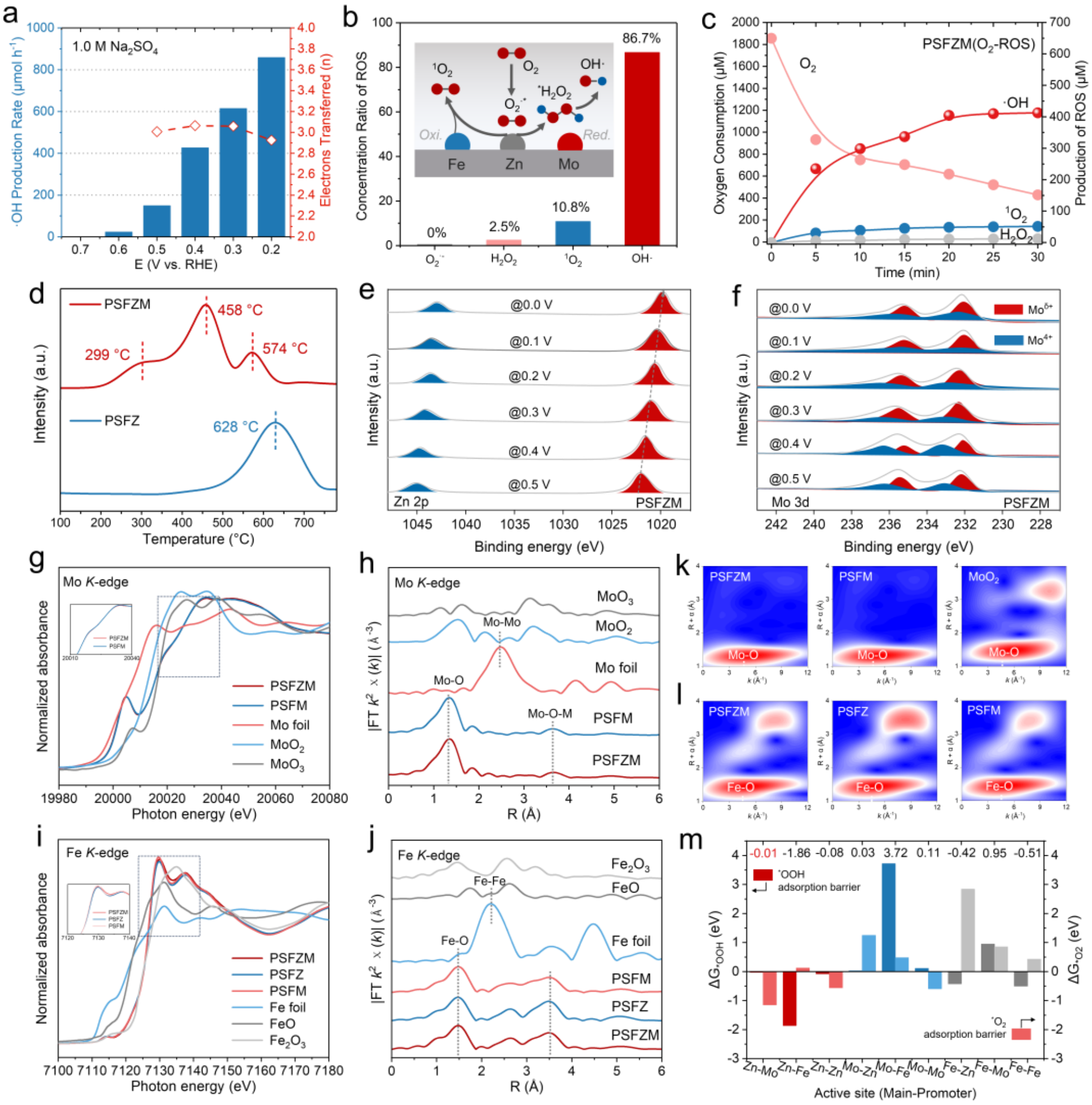

Fig. 4 Mechanism of dual co-catalytic sites promoting ROS formation. a Benzoic acid fluorescence spectrophotometry, used to detect the amount of •OH free radicals generated. •OH yield at different oxygen reduction voltages. **b** The proportion of ROS generated by PSFZM through 3e⁻ ORR, and the inset shows the single-site active domain mechanism. **c** Oxygen utilization during 30-min continuous testing. Quantitative determination of the consumed dissolved O_2 and generated ROS. **d** O_2-TPD profiles of PSFZM and PSFZ. In-situ XPS spectra of **e** Zn 2p and **f** Mo 3d in electrochemistry. **g** Mo K-edge XANES spectra of PSFZM, PSFM, Mo foil, MoO_2 and MoO_3. **h** Mo K-edge k^3-weighted FT-EXAFS spectra. **i** Fe K-edge XANES spectra of PSFZM, PSFZ, PSFM, Fe foil, FeO and Fe_2O_3. **j** Fe K-edge k^3-weighted FT-EXAFS spectra. WT-EXAFS spectra of **k** Mo K-edge and **l** Fe K-edge. **m** Comparison of free energies of adsorption of intermediate *OOH and O_2 at B sites in PSFZM.

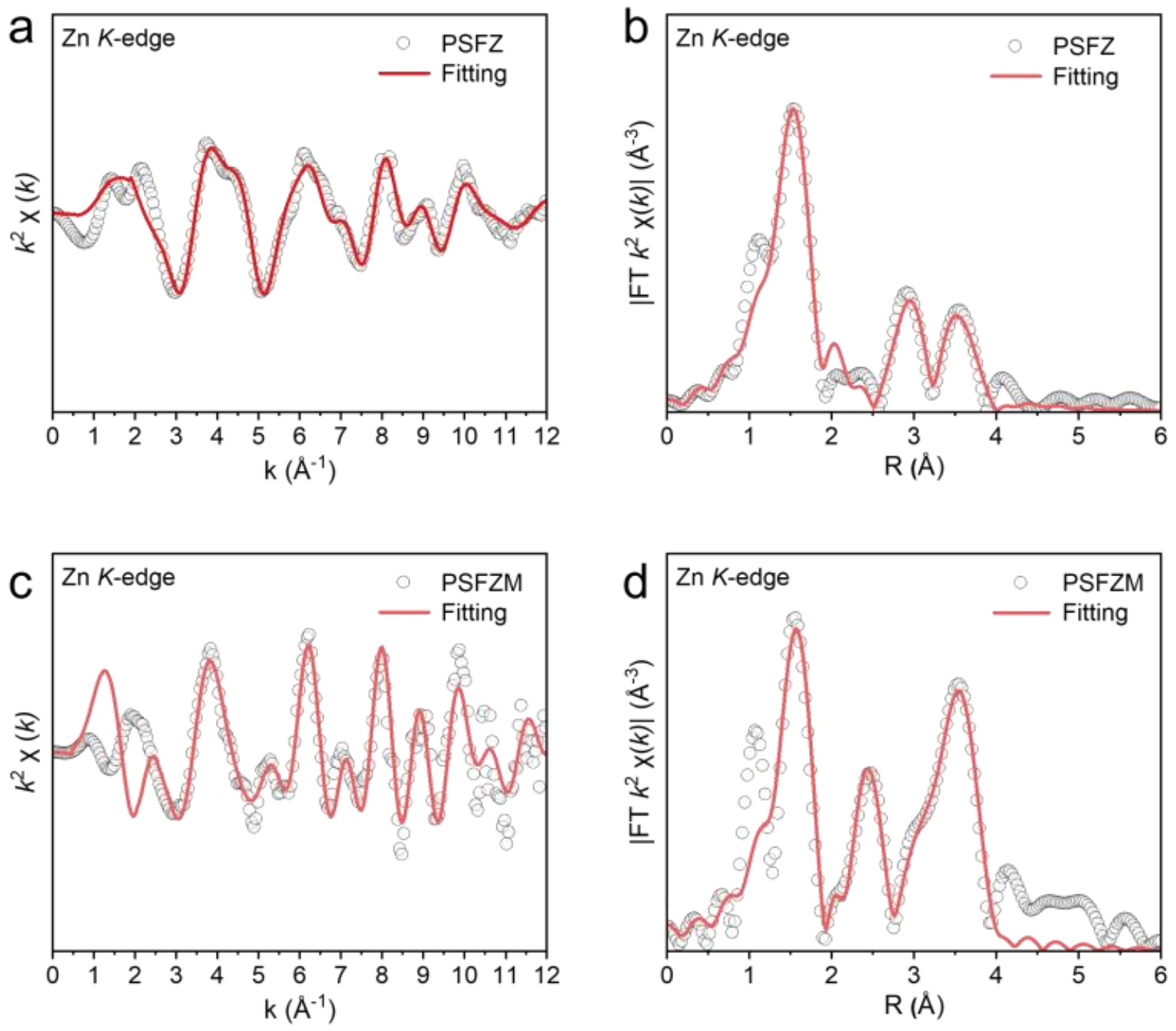

Supplementary Fig. 13 Fitting of the Zn K-edge EXAFS K spectra for **a** PSFZ and **c** PSFZM. Fitting of the Zn K-edge EXAFS R space fitting result for **b** PSFZ and **d** PSFZM.

Supplementary Table 2. EXAFS fitting parameters at the Zn K-edge for various samples.

Sample	Shell	Bond length (Å)	Coordination Number	σ^2 (Å^2)	E0 shift (eV)	R-factor
PSFZM	Zn-O	1.95±0.01	3.7±0.6	0.003±0.002	1.3±2.0	0.014
	Zn-O-Zn	3.28±0.01	3.1±0.4	0.011±0.008		
	Zn-O-M	3.88±0.01	5.0±0.6	0.016±0.010		
PSFZ	Zn-O	1.99±0.02	4.3±1.0	0.003±0.001	0.8±2.4	0.005
	Zn-Zn	2.97±0.01	6.0±1.6	0.012±0.004		
	Zn-O-Zn	3.39±0.01	3.0±0.9	0.011±0.004		
	Zn-O-M	3.91±0.03	12.3±0.8	0.007±0.004		

Note: The value of the amplitude reduction factor (S_0^2) was fixed to 0.70; Bond length is the interatomic distance; CN is the coordination number; σ^2 is Debye-Waller factor (a measure of thermal and static disorder in absorber scatter distance); E_0 shift is edge-energy shift (the difference between the zero kinetic energy value of the sample and that of the theoretical model); R factor is used to value the goodness of the fitting.

Supplementary Table 4. EXAFS fitting parameters at the Mo K-edge for various samples.

Sample	Shell	Bond length (Å)	Coordination Number	σ^2 (Å^2)	E0 shift(eV)	R-factor
PSFZM	Mo-O	1.77±0.01	4.0±0.2	0.001±0.001	6.7±0.9	0.012
	Mo-O-M	3.99±0.01	6.8±0.8	0.012±0.004		
PSFM	Mo-O	1.77±0.01	3.9±0.3	0.001±0.001	6.4±1.0	0.014
	Mo-O-M	3.98±0.02	5.6±0.6	0.011±0.004		

Note: The value of the amplitude reduction factor (S_0^2) was fixed to 0.95; Bond length is the interatomic distance; CN is the coordination number; σ^2 is Debye-Waller factor (a measure of thermal and static disorder in absorber scatter distance); E_0 shift is edge-energy shift (the difference between the zero kinetic energy value of the sample and that of the theoretical model); R factor is used to value the goodness of the fitting.

Supplementary Table 5. EXAFS fitting parameters at the Fe K-edge for various samples.

**Sample**	**Shell**	**Bond length (Å)**	**Coordination Number**	**σ^2 (Å^2)**	**E0 shift (eV)**	**R-factor**
**PSFZM**	Fe-O	1.95±0.02	4.2±0.8	0.003±0.002	-2.2±0.5	0.014
	Fe-Fe	3.00±0.04	3.8±0.5	0.005±0.002		
	Fe-O	3.44±0.04	3.7±0.9	0.012±0.006		
	Fe-O-M	3.80±0.04	6.2±1.0	0.010±0.006		
**PSFZ**	Fe-O	1.93±0.02	4.1±0.8	0.002±0.002	-3.2±2.4	0.008
	Fe-Fe	2.99±0.04	4.0±0.3	0.004±0.002		
	Fe-O	3.45±0.03	3.9±0.4	0.007±0.006		
	Fe-O-M	3.79±0.02	8.2±1.0	0.004±0.001		
**PSFM**	Fe-O	1.94±0.01	4.4±0.7	0.002±0.002	-3.1±1.9	0.006
	Fe-Fe	3.02±0.04	3.5±0.7	0.014±0.006		
	Fe-O	3.48±0.02	4.1±0.4	0.009±0.007		
	Fe-O-M	3.82±0.02	5.6±1.4	0.004±0.001		

Note: The value of the amplitude reduction factor (S_0^2) was fixed to 0.72; Bond length is the interatomic distance; CN is the coordination number; σ^2 is Debye-Waller factor (a measure of thermal and static disorder in absorber scatter distance); E_0 shift is edge-energy shift (the difference between the zero kinetic energy value of the sample and that of the theoretical model); R factor is used to value the goodness of the fitting.

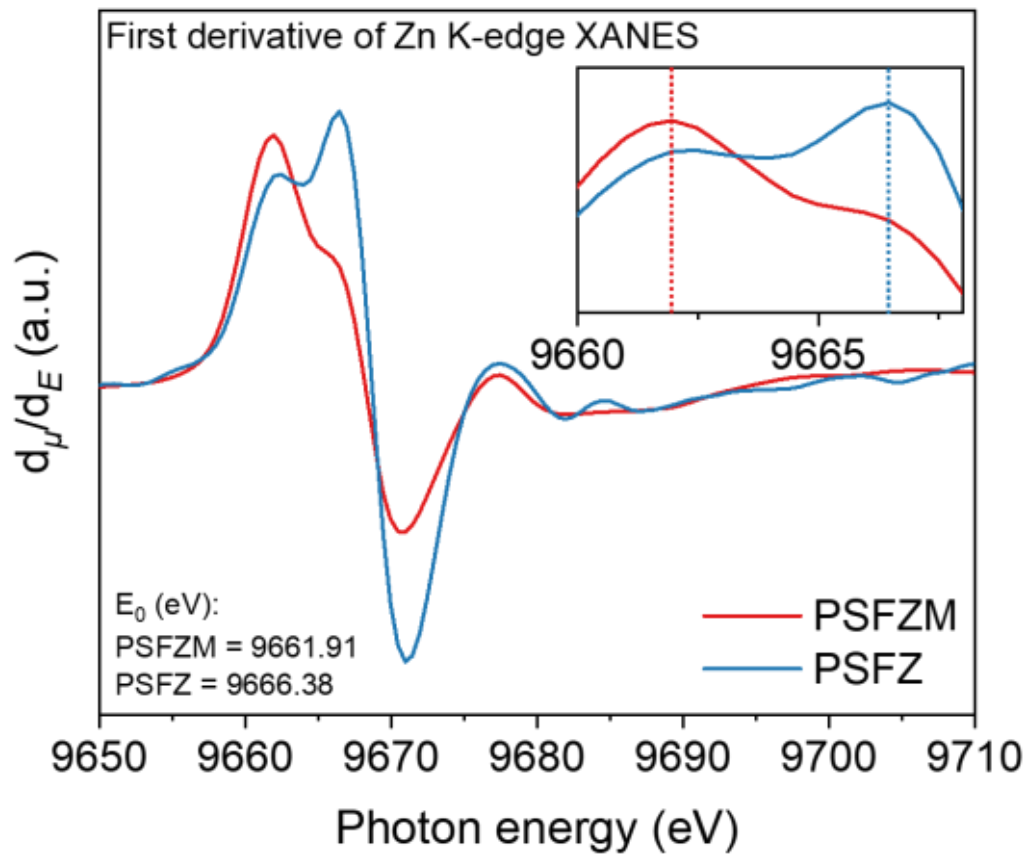

Supplementary Fig. 12 First-derivative Zn K-edge XANES spectra of PSFZM and PSFZ.

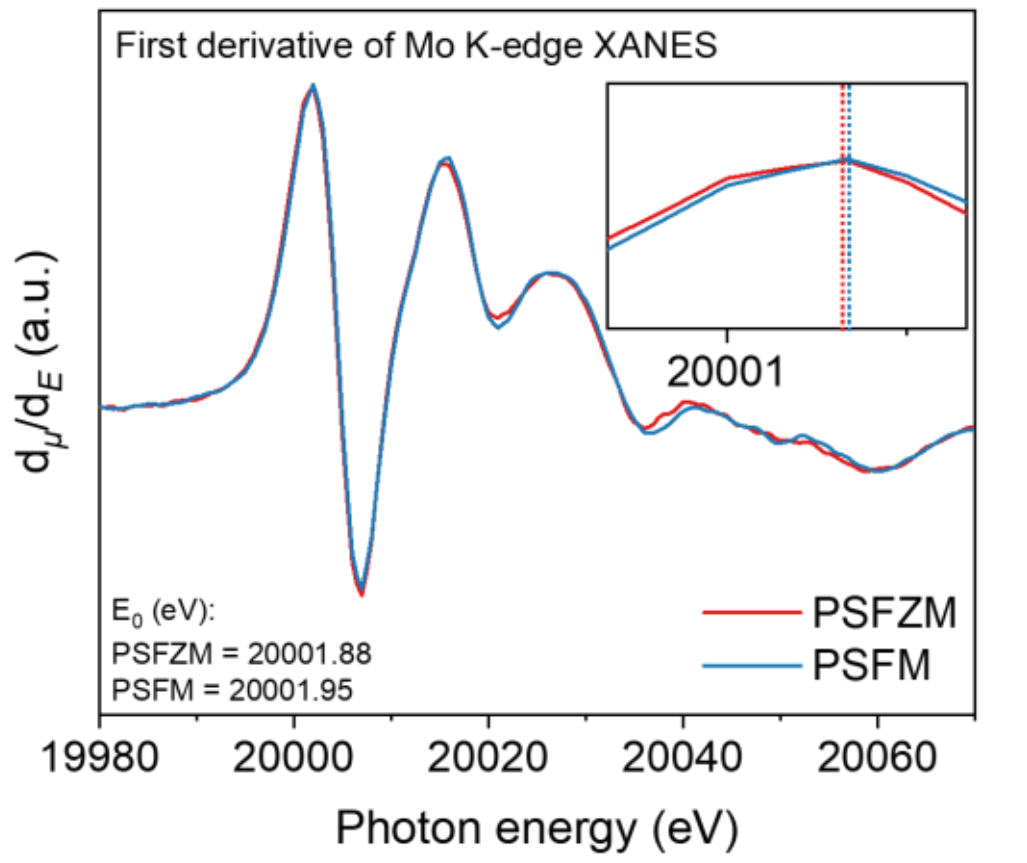

Supplementary Fig. 36 First-derivative Mo K-edge XANES spectra of PSFZM and PSFM.

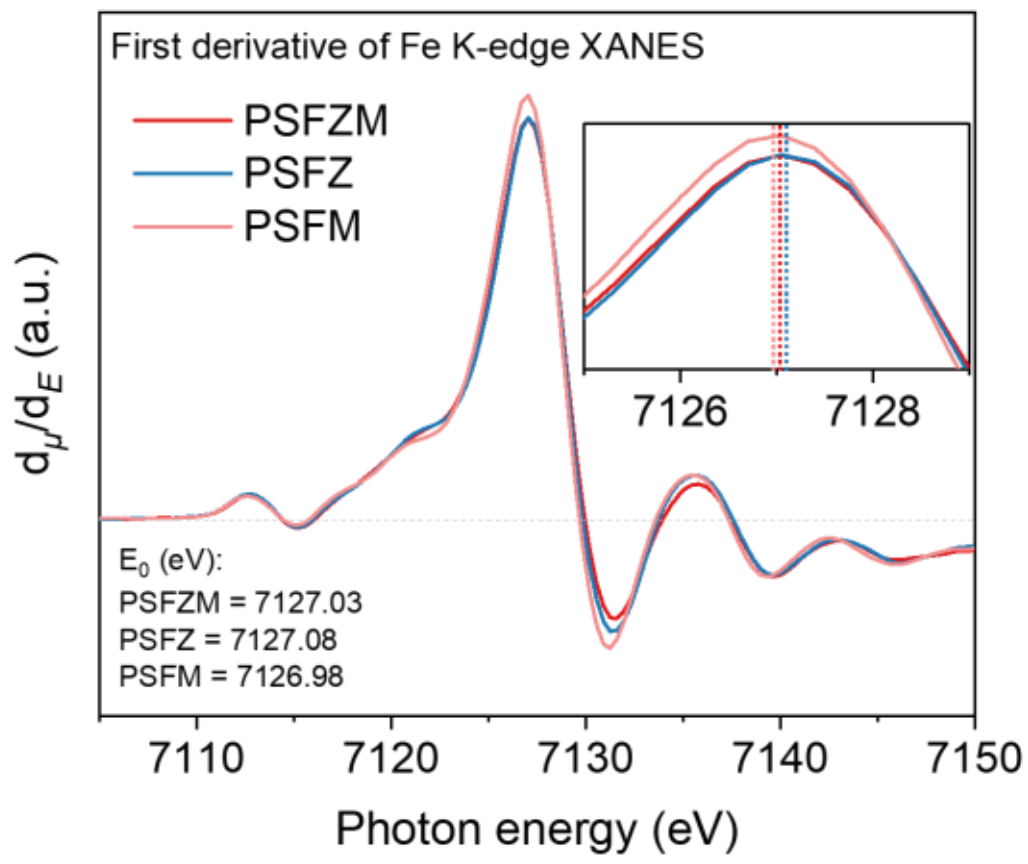

Supplementary Fig. 37 First-derivative Fe K-edge XANES spectra of PSFZM, PSFZ and PSFM.

Manuscript Revision:

The local coordination environment was probed by X-ray absorption fine structure (XAFS) spectroscopy. The Zn K-edge XANES spectrum of PSFZM exhibits an absorption edge positioned between those of Zn foil and ZnO, indicating a reduced Zn valence state ($Zn^{\delta+}$) relative to PSFZ (**Fig. 2j, Supplementary Fig. 12**). Consistently, the Zn–O coordination number decreases from 4.3 ± 1.0 in PSFZ to 3.7 ± 0.6 in PSFZM (**Supplementary Table 2**), corroborating this observation. Fourier-transformed EXAFS (FT-EXAFS) analyses further reveal a pronounced peak at ~2.48 Å in PSFZM, located between the Zn–Zn scattering of Zn foil (2.24 Å) and the Zn–O–Zn scattering of ZnO (2.98 Å) (**Supplementary Fig. 13**). This intermediate position indicates that the Zn-O-Zn motif in PSFZM does not arise from a continuous Zn-O-Zn-O aggregated network, but rather reflects a second-nearest-neighbor geometric correlation between Zn and the surrounding oxygen polyhedra. More importantly, PSFZM features a distinct Zn–O–M coordination shell with a bond length of 3.88 ± 0.01 Å, and the spectrum can be reproduced without invoking any direct Zn-Zn scattering contribution. This scattering path is absent in both PSFZ and the Zn reference samples (**Fig. 2k**), pointing to a stronger Zn-centred local ordering tendency in PSFZM[33]. Wavelet-transformed (WT) EXAFS contour plots (**Fig. 2l**) resolve the scattering contributions in the (k, R) plane and further substantiate this conclusion: PSFZM displays a low-k intensity maximum corresponding to Zn–O backscattering, together with a discrete high-k lobe at k ≈ 9 $Å^{-1}$ that is attributed to the heavier second-shell Zn–O–M path. Combined with the X-ray photoelectron spectroscopy (XPS) results, this direct structural evidence confirms the successful construction of atomic-scale Zn–O–M coupling within the perovskite matrix (**Supplementary Figs. 14-18**). Taken together, these structural analyses verify that periodically modulated active domains have been successfully established in PSFZM.

Combining the Mo K-edge and Fe K-edge XANES spectra (**Figs. 4g-4j and Supplementary Figs 36-39**) with the WT-EXAFS spectra (**Figs. 4k,4l**) revealed that the chemical states and bond lengths of Mo-O and Fe-O in PSFZM are similar to those observed

in PSFZ and PSFM. The bond lengths were determined to be 1.77 ± 0.01 Å for Mo-O and 1.95 ± 0.02 Å for Fe-O, indicating that the chemical states of Mo and Fe are unaffected by the special distorted Ruddlesden-Popper structure (**Supplementary Tables 4, 5, Supplementary Figs 40, 41**). The adsorption characteristics and intermediate evolution processes in PSFZM align with those in PSFZ and PSFM. This further supports the conclusion that Zn is the primary adsorption site in PSFZM, dominating the continuous activation of O_2 at this site.

4. In line 121, the RRDE results are described as delivering a current density of 4.2 mA cm^{-2} at 0.07–0.10 V (vs. RHE), approaching the theoretical diffusion-limited current for a 3e$^-$ ORR pathway. However, the data at 0.10 V (vs. RHE) are not shown, and the electron transfer number appears to increase in this potential range, possibly entering the HER regime. ESR measurements should also be extended to include data at 0.10 V (vs. RHE) to ensure consistency.

Response: We thank the reviewer for this insightful comment. We extended the ESR measurements to 0.10 V versus RHE. The newly added ESR spectra show a clear DMPO–•OH signal at 0.10 V under O_2-saturated conditions. This result confirms that •OH generation remains effective at 0.10 V and is consistent with the 3e$^-$ ORR behaviour derived from RRDE in this potential range.

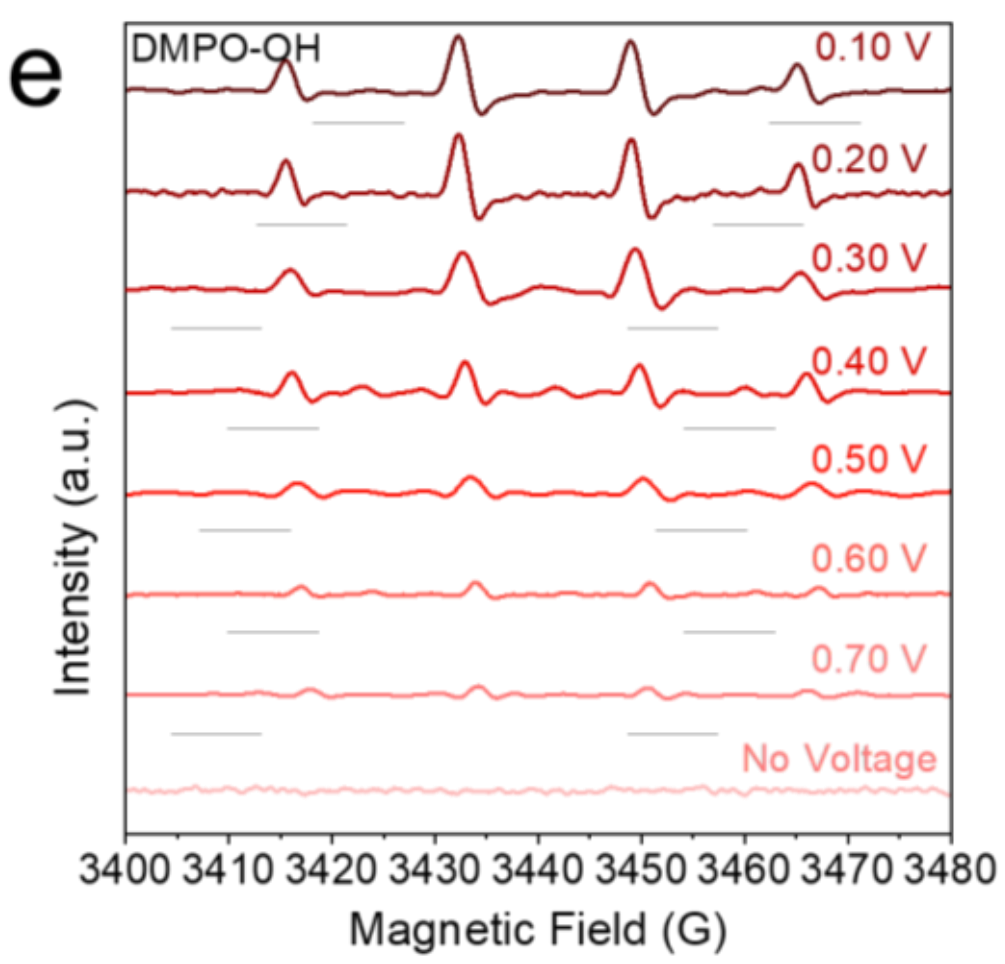

e DMPO-•OH adduct EPR spectra of ORR electrochemistry over PSFZM catalyst collected in an incremental voltage configuration.

Manuscript Revision:

Electron spin resonance (ESR) spectroscopy confirmed that PSFZM predominantly generates •OH during electrocatalysis, with a peak intensity much higher than that of the other samples (**Fig. 3d and Supplementary Fig. 27**). Quasi-operando ESR measurements showed that the signal intensity increased with increasing cathodic bias and reached a maximum at 0.2 V versus RHE (**Fig. 3e**). This directly indicates that •OH originates from the ORR process and is closely correlated with the electrolytic cell voltage.

5. The •OH concentrations determined using benzoic acid and coumarin fluorescence methods show significant discrepancies, which require explanation. The method used to calculate oxygen utilization efficiency should be clearly described. More importantly, the quantification of •OH and the derivation of Faradaic efficiency lack sufficient rigor and transparency. Detailed calibration procedures, error analysis, and clear definitions are necessary.

Response: Thank you for your comments. We have carefully checked your concerns. The discrepancy between the benzoic acid and coumarin fluorescence assays can be attributed to the intrinsic differences between the two probes. Coumarin is relatively hydrophobic and may preferentially probe interfacial or surface-accessible •OH, whereas benzoic acid exists mainly as benzoate under alkaline conditions and is more suitable for detecting solution-accessible •OH. In addition, the two probes differ in their trapping efficiency, fluorescent product yield, adsorption affinity and product stability during electrolysis. Therefore, the absolute •OH concentrations obtained from these two assays are not expected to be identical. In the revised manuscript, we define these values as probe-accessible •OH-equivalent concentrations. The benzoic acid assay was used as the primary quantitative method, while the coumarin assay was used as an independent validation. Importantly, both methods show the same catalyst trend and potential-dependent behaviour, supporting enhanced •OH generation on PSFZM.

We provided detailed calibration procedures and error analyses for the calculation of oxygen utilization efficiency, the quantification of •OH, and the derivation of Faradaic efficiency, and included them in the Methods section.

Manuscript Revision:

Supplementary Note 2. Calibration method for the determination of •OH concentration by fluorescence method of benzoic acid and coumarin.

For BA-based •OH quantification, the electrolyte contained BA under the same pH, ionic strength and electrolysis conditions as those used for ORR measurements. Aliquots were collected at fixed time intervals, filtered, and analysed by steady-state fluorescence spectroscopy. External calibration curves were established using standard hydroxybenzoic acid solutions prepared in the same electrolyte matrix. The fluorescence intensity was corrected by subtracting the blank signal obtained without applied bias or without catalyst. The apparent •OH concentration was obtained from the calibration curve and converted into the amount of •OH according to

$$n_{\cdot\mathrm{OH}} = C_{\cdot\mathrm{OH}}V,$$

where $C_{\cdot\mathrm{OH}}$ is the calibrated apparent •OH-equivalent concentration and V is the electrolyte volume. The •OH production rate was calculated from the slope of $n_{\cdot\mathrm{OH}}$ versus electrolysis time.

For CM-based validation, standard 7-hydroxycoumarin solutions were used to construct calibration curves under the same electrolyte conditions. Because the 7-hydroxycoumarin product can be further oxidized in strongly oxidative systems, CM-derived values were used only as an independent verification of the trend rather than as the primary basis for Faradaic-efficiency calculation.

Supplementary Note 3. Calculation of apparent faraday efficiency.

The apparent Faradaic efficiency for cathodic •OH generation through the formal $3e^-$ ORR pathway was calculated as

$$FE_{\cdot\mathrm{OH}} = \frac{3Fn_{\cdot\mathrm{OH}}}{Q} \times 100\%,$$

where F is the Faraday constant, $n_{\cdot\mathrm{OH}}$ is the amount of •OH determined from the BA calibration, and $Q = \int I\, dt$ is the total cathodic charge passed during electrolysis. For H_2O_2, the corresponding Faradaic efficiency was calculated as

$$FE_{\mathrm{H_2O_2}} = \frac{2Fn_{\mathrm{H_2O_2}}}{Q} \times 100\%.$$

For the paired full-cell test, the reported value is defined as an overall electron-to-•OH generation efficiency rather than a single-electrode Faradaic efficiency, because •OH can be generated through cathodic ORR and anodic water oxidation.

The oxygen-utilization efficiency was calculated from the oxygen balance:

$$\eta_{O_2 \to ROS} = \frac{n_{O_2,ROS}}{n_{O_2,consumed}} \times 100\%.$$

Here, $n_{O2,\ consumed}$ was determined from the decrease in dissolved O_2 after blank correction, and $n_{O2,\ ROS}$ was obtained by summing the O_2-equivalent molar amounts of detected •OH, H_2O_2 and $^1O_2$. The ROS selectivity was calculated as

$$S_i = \frac{n_{O_2,i}}{\sum n_{O_2,i}} \times 100\%.$$

Calibration curves were repeated at least three times. The limit of detection and limit of quantification were calculated as $3\sigma_{\text{blank}}/k$ and $10\sigma_{\text{blank}}/k$,respectively, where k is the slope of the calibration curve. Errors are reported as standard deviations from independent experiments, and propagated uncertainties were used for calculated efficiencies.

6. The calculated free-energy landscape supports the special role of the Zn–O–Mo motif, and the comparison across local motifs is valuable. However, the manuscript uses language such as "barrier-free conversion" and describes the rate-determining step as exergonic on PSFZM. Free-energy profiles alone do not establish the absence of kinetic barriers. If the authors wish to make a kinetics-based argument, transition-state calculations (e.g., CI-NEB) are required. At a minimum, the language should be softened and thermodynamic descriptors should not be conflated with reaction barriers.

Response: We thank the reviewer for this important and constructive comment. We have carefully revised the manuscript to avoid conflating thermodynamic driving force with kinetic barriers. In the revised manuscript, we replaced "barrier-free conversion" with more accurate wording. To provide a kinetics-based assessment, we performed CI-NEB transition-state calculations and potential-dependent calculations for the key elementary steps involved in the proposed ORR-to-•OH pathway. The newly added CI-NEB results show that the Zn–O–Mo motif in PSFZM lowers the activation barrier for peroxide activation/O–O bond cleavage compared with the Zn-only, Mo-only and Fe-centred local environments. Meanwhile, the competing pathway toward complete $4e^-$ reduction remains less favourable on PSFZM. These results support the special role of the Zn–O–Mo motif in promoting the surface-confined consecutive $3e^-$ ORR pathway. We have also revised the discussion of the free-energy landscape. The free-energy calculations are now presented as thermodynamic descriptors to identify favourable intermediate stabilization and the potential-determining

step.

Manuscript Revision:

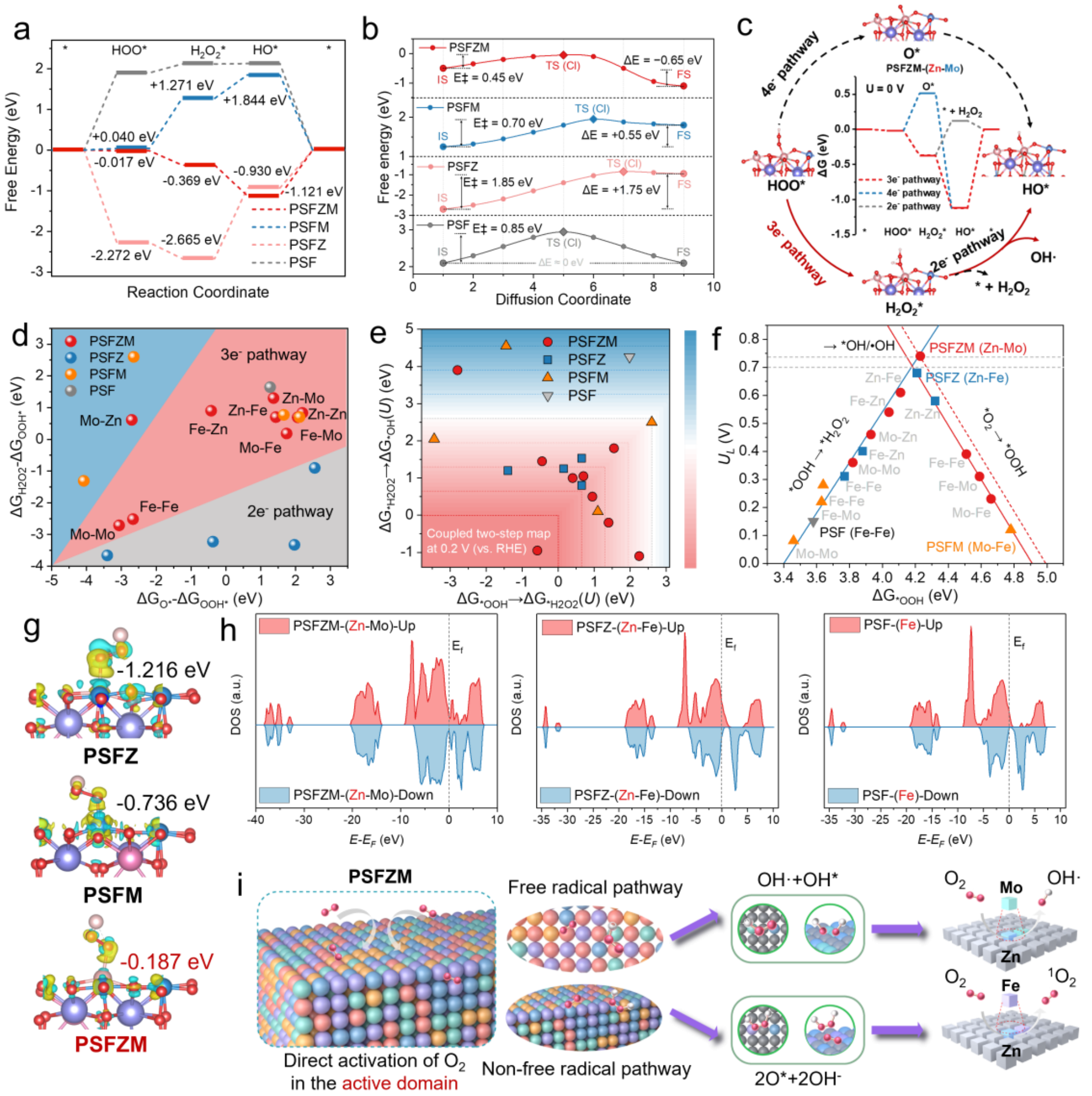

Fig. 5 DFT calculations involving each reaction site. a Gibbs free energy of the whole reaction pathway for the generation of •OH via three-electron ORR for all samples. **b** CI-NEB energy profiles for the rate-determining elementary step on the four candidate catalysts. **c** Structural models and corresponding free energy comparisons of the 3e⁻ ORR and 4e⁻ ORR pathways on the PSFZM catalyst. **d** Energy differences between reaction intermediates at equilibrium potential. The blue, red, and gray regions indicate active sites for the thermodynamically driven 4e⁻, 3e⁻, and 2e⁻ ORR pathways, respectively. **e** Two-step coupled selectivity map at 0.2 V (vs. RHE). **f** Scaling-relation analysis of intermediate adsorption free energies on dual-atom sites. **g** Side view of the charge density difference for H_2O_2 adsorbed on PSFZ/PSFM/PSFZM, the cyan region represents charge depletion, and the

yellow region indicates charge accumulation. **h** Calculated density of states (DOS) analysis for PSF/PSFZ/PSFZM after H_2O_2 adsorption. **i** Schematic diagram of the reaction pathway of O_2 generating ROS through free radical and non-free radical at the active domain of PSFZM. Colors represent: Pr (red), Sr (purple), Fe (yellow), Zn (green), Mo (blue).

Density functional theory (DFT) calculations show that H_2O_2 desorption from the Zn site is an uphill process, that is, a non-spontaneous step with $\Delta G = +0.52$ eV. This feature suppresses H_2O_2 release and thereby enhances the selectivity toward the $3e^-$ ORR pathway. Among all examined structural motifs, the Zn-O-Mo unit in the material constructs a confined microenvironment that optimizes the orientation of reaction intermediates and their desorption kinetics (**Supplementary Figs. 50-65**).

We further performed climbing-image nudged elastic band (CI-NEB) calculations to locate the initial state (IS), transition state (TS), and final state (FS) of each model, thereby evaluating the kinetic accessibility of the key O-O bond activation step. The transition-state profiles show that PSFZM exhibits the lowest energy barrier for converting surface-adsorbed H_2O_2 into •OH-related intermediates. In contrast, PSFZ, PSFM, and PSF encounter higher kinetic barriers or energetically less favorable final states (**Fig. 5b**). This result indicates that the enhanced •OH yield on PSFZM does not arise solely from the thermodynamic stabilization of peroxide species, but also from the simultaneous reduction of the kinetic barrier for intradomain peroxide activation. Meanwhile, the cleavage of $*H_2O_2$, identified as the rate-determining step (RDS), is a downhill process on PSFZM, whereas it is uphill on PSFM (0.75 eV), PSFZ (1.73 eV), and PSF (0.01 eV). Therefore, the unique Zn-O-Mo configuration lowers the critical energy barrier, accelerates electron transfer, and rationalizes the experimentally observed superior •OH generation rate (**Fig. 5c**).

Conventional descriptors, such as ΔG_{*OOH}, often fail to accurately capture the energetics of downstream product formation. Therefore, we introduced $\Delta G_{*H2O2}–\Delta G_{*OOH}$ and $\Delta G_{*O}–\Delta G_{*OOH}$ as more predictive descriptors for reaction selectivity (**Fig. 5d**). Mechanistic mapping shows that the PSFZM catalytic site is located in a specific region that favors the formation of H_2O_2 and •OH while effectively suppressing excessive reduction toward the *O intermediate. A coupled two-step descriptor map at 0.2 V versus RHE further shows that the Zn-O-Mo sites of PSFZM occupy the optimal region where peroxide formation and peroxide activation are balanced (**Fig. 5e**). In contrast, PSFZ stabilizes peroxide intermediates but

lacks sufficient activation capability, leading to H_2O_2 accumulation, while PSFM promotes stronger O–O activation but does not efficiently retain the peroxide intermediate. In **Fig. 5f**, the two volcano branches describe the thermodynamic limitations associated with $*H_2O_2$ and *OOH formation, respectively. The volcano apex approaches the equilibrium potential of the O_2/H_2O_2 couple ($U° = 0.70$ V vs. RHE), defining the minimum theoretical overpotential imposed by the scaling relationship. The Zn–O–Mo motif in PSFZM lies near this optimum and gives the highest limiting potential among all examined motifs ($\Delta G_{*OOH} \approx 4.28$ eV, $U_L \approx$ 0.749 V).This position indicates that the Zn–O–Mo active domain effectively balances *OOH stabilization with $*H_2O_2$ activation, thereby mitigating the conventional scaling constraint and promoting the confined conversion of surface-retained $*H_2O_2$ toward •OH.

7. The full cell is constructed using PSFZM as both cathode and anode, and the authors report a peak FE of 64.7% for •OH generation, while the anode alone is also claimed to generate •OH with FE exceeding 50% and peaking at 58.9%. It remains unclear how the individual cathodic and anodic ROS contributions were separated, how crossover or recombination was excluded, and how the full-cell FE was defined relative to the half-cell values. A more rigorous species balance and electrode-resolved analysis are needed.

Response: Thank you for your comments. We have carefully checked your concerns. We performed electrode-resolved control experiments under otherwise identical electrolyte, current and sampling conditions. The cathodic contribution was evaluated using PSFZM as the cathode with an inert counter electrode, whereas the anodic contribution was quantified using PSFZM as the anode with an inert counter electrode. These measurements allowed us to separately determine the cathodic ORR-derived •OH generation and the anodic water-oxidation-derived •OH generation. The results confirm that both electrodes can contribute to ROS formation, but through different elementary charge-transfer processes.

Second, we added full-cell measurements in a separated configuration with independent catholyte and anolyte sampling. The •OH concentrations in the cathodic and anodic compartments were quantified separately using the same calibrated fluorescence method. The total •OH amount in the full cell was calculated as:

$$n_{\bullet\mathrm{OH,total}} = n_{\bullet\mathrm{OH,cathode}} + n_{\bullet\mathrm{OH,anode}}$$

where $n_{\bullet\mathrm{OH,cathode}}$ and $n_{\bullet\mathrm{OH,anode}}$ were obtained from the catholyte and anolyte, respectively. This electrode-resolved analysis allows the full-cell ROS balance to be

constructed directly rather than inferred from half-cell data.

Third, we clarified the definition of Faradaic efficiency. The cathodic •OH formation is associated with the surface-confined $3e^-$ ORR pathway, whereas anodic •OH generation is treated as a $1e^-$ water/hydroxide oxidation process. Therefore, the apparent cathodic and anodic FEs were calculated as:

$$\mathrm{FE}_{\bullet\mathrm{OH,cathode}} = \frac{3Fn_{\bullet\mathrm{OH,cathode}}}{Q} \times 100\%$$

$$\mathrm{FE}_{\bullet\mathrm{OH,anode}} = \frac{Fn_{\bullet\mathrm{OH,anode}}}{Q} \times 100\%$$

For the full cell, the apparent paired FE for total •OH generation was defined as:

$$\mathrm{FE}_{\bullet\mathrm{OH,full-cell}} = \frac{F(3n_{\bullet\mathrm{OH,cathode}} + n_{\bullet\mathrm{OH,anode}})}{Q} \times 100\%$$

where Q is the total charge passed through the full cell. We now explicitly state that the reported peak value of 64.7% refers to this full-cell apparent paired FE for total probe-accessible •OH, rather than a direct arithmetic comparison with either half-cell FE.

Finally, we revised the discussion of the full-cell results to avoid overinterpretation. The half-cell anodic FE of 58.9% and the full-cell peak FE of 64.7% are now presented as values obtained under different configurations and definitions. The full-cell FE is not treated as a simple sum of independently measured half-cell FEs, because radical recombination, interfacial quenching, mass transport and electrode coupling can all affect the observed ROS yield during paired operation. Instead, the revised manuscript reports the electrode-resolved cathodic and anodic contributions together with the full-cell species balance.

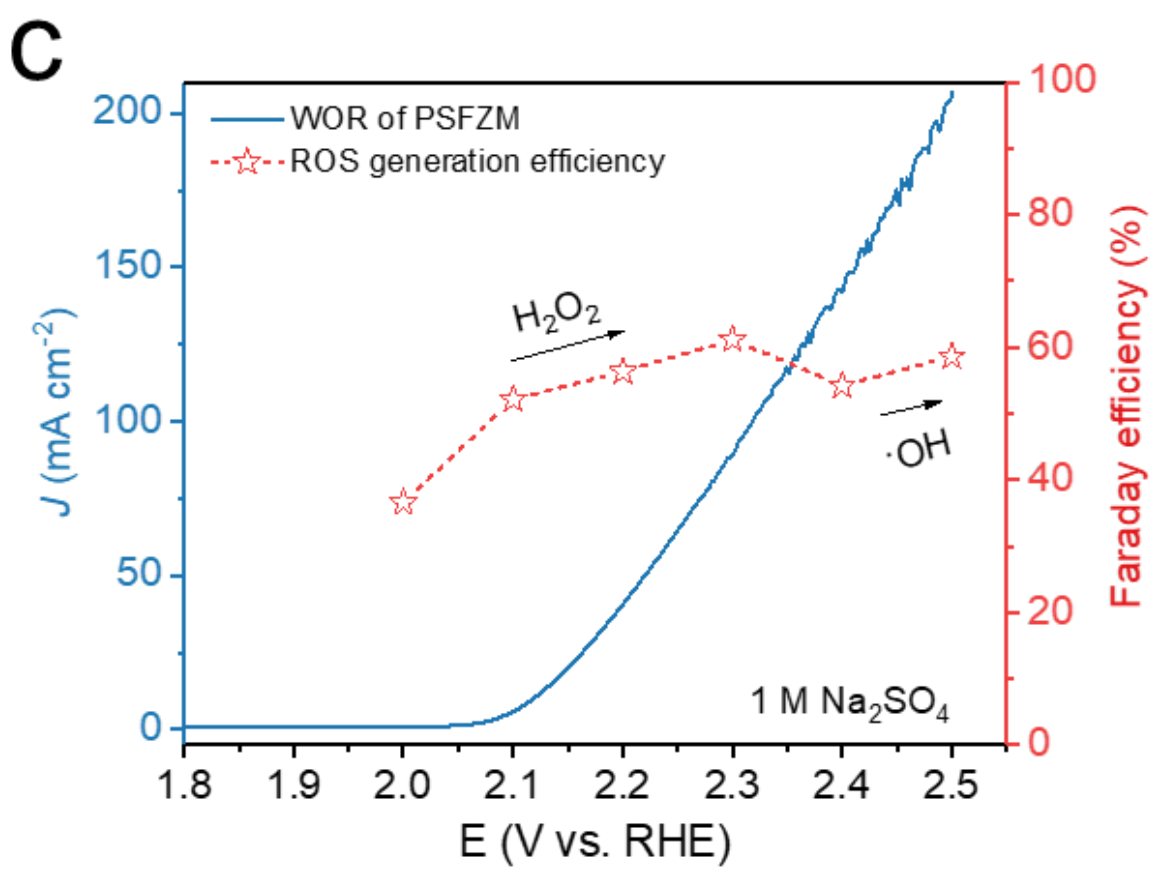

c Polarization I-V curves of PSFZM(WOR) measured in O_2-saturated 1.0 M Na_2SO_4, and

the corresponding FE profiles obtained by the titration method.

Manuscript Revision:

First, we evaluate the WOR performance of PSFZM. DFT calculations reveal that PSFZM exhibits the lowest Gibbs free energy for the rate-determining step (ΔG_{RDS}) of the electron-driven WOR (**Fig. 6b**). Notably, the ΔG_{*OH} > 2.4 eV suggests weak *OH binding on the catalyst surface, suppressing the evolution of H_2O_2 or O_2 and instead favoring •OH production. Electrochemical measurements under 1.0 M Na_2SO_4 further corroborate these findings, with FE for •OH formation exceeding 50% at >2.0 V and a peak FE_{anode} of 58.9% achieving at ~2.5 V, alongside a high current density of ~200 mA cm^{-2} (**Figs. 6c and Supplementary Fig. 88**). Chronoamperometric stability tests further validate the system's scalability and robustness for sustained •OH generation.

In this setup, the $3e^-$ ORR || e^- WOR full-cell system delivered a remarkable peak Faradaic efficiency of 64.7% for •OH generation (**Fig. 6c, d**).

8. There are serious inconsistencies in the Methods and Supporting Information that undermine confidence in the study. A particularly concerning example is the LC–MS method: Supplementary Note 3 reports a flow rate of 0.25 mL min^{-1} and an injection volume of 5 μL, whereas Supplementary Note 7 reports 20 mL min^{-1} and 20 μL. These cannot both be correct, and 20 mL min^{-1} is implausible for a conventional LC–MS setup. There are additional inconsistencies, including C 1s calibration at 284.7 eV versus 284.8 eV, a very broad calcination temperature range (1000–1100 °C) without clear clarification, and a negative "raw material input" value in Supplementary Table 1. These issues must be corrected thoroughly.

Response: We sincerely thank the reviewer for pointing out these inconsistencies. We fully agree that such discrepancies in the Methods and Supplementary Information may affect the reliability and reproducibility of the study. Therefore, we performed a thorough cross-check of the Methods, Supplementary Notes, tables and figure captions, and corrected all inconsistent or erroneous entries in the revised manuscript.

First, we corrected the LC–MS method and unified the LC–MS description across all relevant sections. Second, we revised the XPS calibration description. All XPS spectra were referenced to the adventitious carbon C 1s peak at 284.8 eV. Third, we clarified the exact calcination temperature and duration used for the reported samples: in air at 1000 °C for 24

h. Fourth, we corrected the negative "raw material input" value in Supplementary Table 1. This value resulted from an error in the spreadsheet formula/sign convention during the life-cycle or material-balance calculation. We have rechecked the table, corrected the raw material input value and verified the consistency of all related calculations.

In addition to these specific corrections, we performed a comprehensive consistency check of the Methods and Supplementary Information, including reagent amounts, instrumental parameters, electrochemical conditions, calibration procedures, unit expressions and table annotations. The revised version has been carefully updated to ensure that the experimental procedures are internally consistent and reproducible. We appreciate the reviewer's careful reading, which helped us identify and correct these important methodological inconsistencies.

Manuscript Revision:

Norfloxacin concentration was determined by UV-visible spectrophotometry with a maximum absorption wavelength of λ=278 nm. Liquid chromatography-mass spectrometry (LC-MS, Bruker) was used to detect the intermediate products in the degradation process of norfloxacin, mainly using an SB-C18 reversed-phase column (5 μm×4.6 mm×250 mm), a methanol:water gradient elution mobile phase, an injection volume of 5 μL, a flow rate of 0.25 mL min^{-1}, and an electrospray ionization (ESI) source of positive ion scanning.

All data were normalized relative to the C 1s binding energy of 284.8 eV.

Finally, after calcination in air at 1000 °C for 24 h, $Pr_{1.0}Sr_{1.0}Fe_{0.5}Zn_{0.25}Mo_{0.25}O_{4-\delta}$ catalysts were obtained, including $Pr_{1.0}Sr_{1.0}Fe_{1.0}O_{4-\delta}$ with different precursors, $Pr_{1.0}Sr_{1.0}Fe_{0.75}Zn_{0.25}O_{4-\delta}$ and $Pr_{1.0}Sr_{1.0}Fe_{0.75}Mo_{0.25}O_{4-\delta}$ nanofibers.

Supplementary Table 6. The O_2 activation system was compared with the H_2O_2 activation system and the PMS activation system from five dimensions: MA, SA, MRR, TOC and RMI.

	**O_2 activation**	**PMS activation**	**H_2O_2 activation**
**Mass activity (MA) (g min^{-1} g^{-1})**	13.33	0.95	3.68
**Surface activity (SA) (g min^{-1} m^{-2})**	0.0667	0.00212	0.0245

**Metal retention rate (MRR) (%)**	99.96	89.56	85.65
**Total organic carbon (TOC) (%)**	63.41	70.64	59.63
**Raw material input (RMI) (mg)**	0	20	20

9. The collection efficiency (N) should be experimentally determined and clearly described. The methodology and calibration procedures should be explicitly provided. Relevant literature (e.g., DOI: 10.1002/adma.202521237) offers detailed guidance and should be considered.

Response: Thank you for your comments. We have carefully checked your concerns. In the revised manuscript, we added a dedicated section in the Supporting Information to describe the calibration procedure for N, following the standard RRDE calibration protocol and the methodology suggested by the reviewer, and provided the corresponding experimental results. We also consulted the reference recommended by the reviewer and cited it in the revised manuscript.

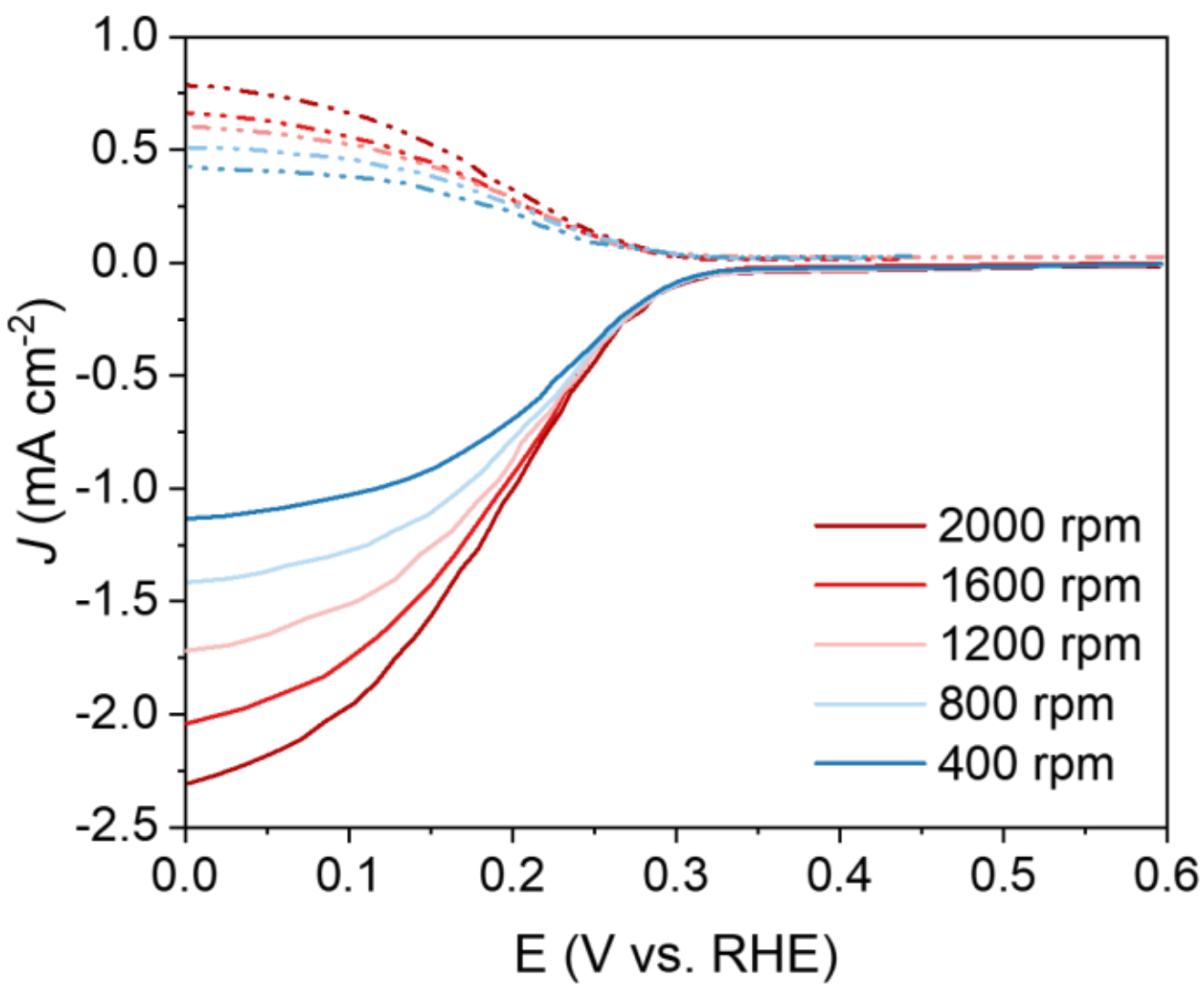

Supplementary Fig. 20 LSV curves in 10 mM $K_3[Fe(CN)_6]$ at different rotation speeds.

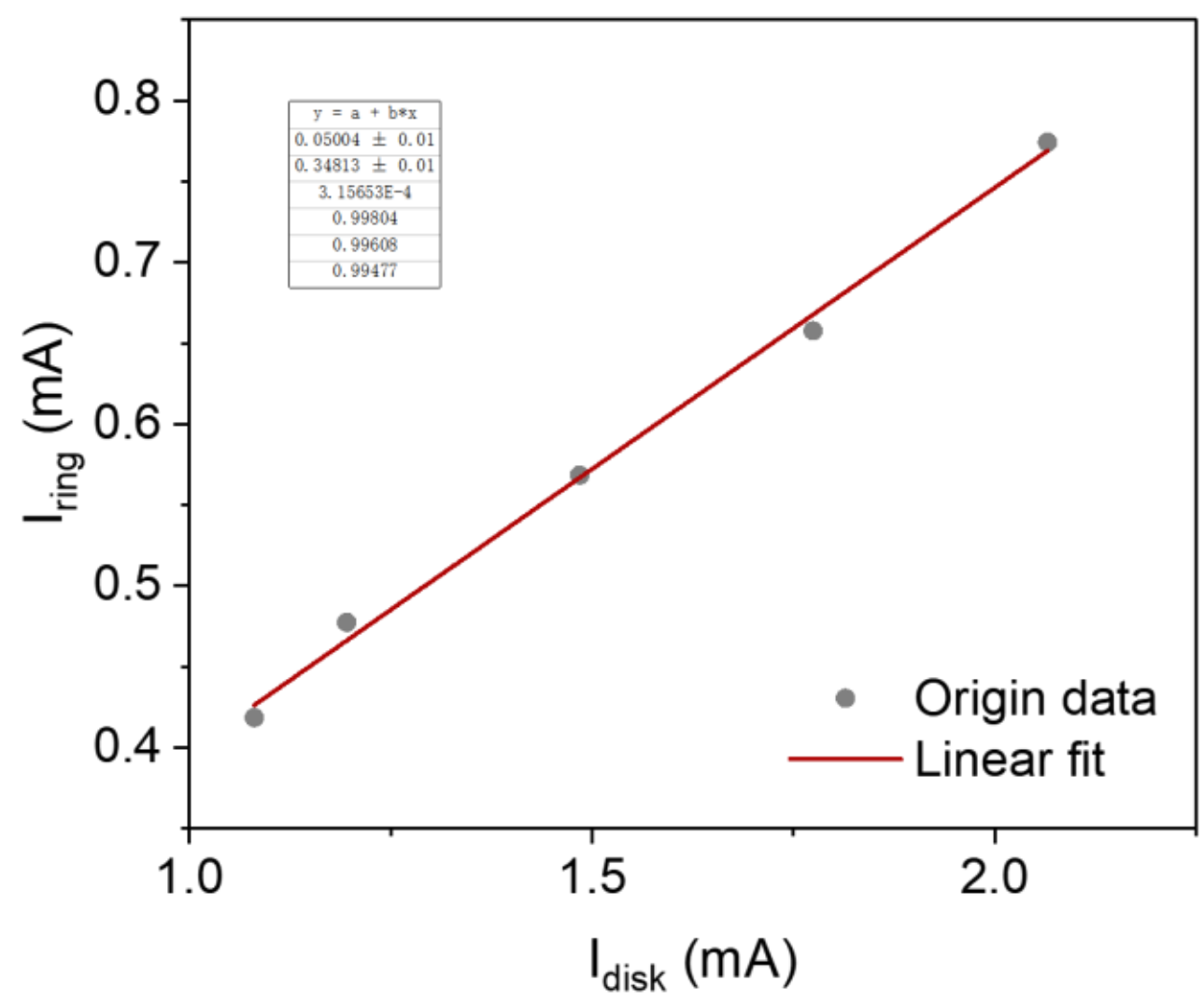

Supplementary Fig. 21 The collection efficiency (N) of RRDE, which was determined to be 34.8%.

Manuscript Revision:

Prior to conducting ORR measurements, the collection efficiency of the rotating ring-disk electrode (RRDE) was experimentally calibrated (collection efficiency of N = 34.8%, **Supplementary Figs. 20, 21**)[34].

34. Li, Y. *et al.* Scalable and Customizable Single-Atom Coatings for pH-Universal H_2O_2 Electrosynthesis. *Adv. Mater.* **38**, e21237 (2026).

Supplementary Note 10. Calibration of the RRDE collection efficiency (N).

The collection efficiency N of the RRDE was experimentally calibrated using the $[Fe(CN)_6]^{3-/4-}$ redox couple in N_2-saturated electrolyte, following the protocol described previously[1]. The calibration electrolyte consisted of 10 mM $K_3[Fe(CN)_6]$ (≥99.0%, Sigma-Aldrich) prepared with ultrapure water (18.2 MΩ·cm, Milli-Q), and was deaerated by bubbling high-purity N_2 (≥99.999%) for at least 30 min prior to and throughout the measurements.

A three-electrode configuration was used, comprising a glassy carbon disk–platinum ring RRDE (disk diameter 4.0 mm; ring inner/outer diameter 5.0/7.0 mm; Pine Research) as the working electrode, a platinum wire as the counter electrode, and a saturated Hg/HgO electrode as the reference. Prior to each measurement, the glassy carbon disk was polished

sequentially with 1.0, 0.3 and 0.05 μm alumina slurries (Buehler) to a mirror finish, followed by ultrasonication in ultrapure water.

Measurements were performed at five rotation rates (ω = 400, 800, 1200, 1600 and 2000 rpm). The electrode was preconditioned with five cyclic voltammetric scans at each rotation rate to ensure a stable interface, and the limiting plateau currents for both the disk and ring were extracted for analysis.

The collection efficiency was calculated as:

$$N = -I_{ring} / I_{disk}$$

where I_{ring} is the limiting oxidation current at the ring and I_{disk} is the limiting reduction current at the disk.

10. The manuscript and Supporting Information require careful proofreading for language consistency and technical accuracy. Several non-idiomatic expressions and typographical errors. For example: "Koutechy-Levich" in Supplementary Fig. 14 should be corrected to "Koutecky-Levich." The heading "Practical Decontamination Performance and System Engineering. Actual Decontamination Performance and System Engineering." contains an obvious duplication. UV-UV spectrophotometry" should likely be "UV–vis spectrophotometry." Subscripts and superscripts in the $^1O_2$ quantification equations should be carefully checked.

Response: We thank the reviewer for carefully pointing out these language and formatting issues. We agree that the manuscript and Supporting Information required further proofreading to improve language consistency, technical accuracy and readability. We have therefore thoroughly checked the revised manuscript, Methods, Supporting Information, figure captions, equations and table annotations, and corrected the typographical and formatting errors.

Specifically, we have corrected "Koutechy–Levich" to "Koutecky–Levich" in Supplementary Fig. 14 and the corresponding caption. We have removed the duplicated heading "Practical Decontamination Performance and System Engineering. Actual Decontamination Performance and System Engineering." and replaced it with the unified heading "Practical Decontamination Performance and System Engineering." We have also corrected "UV–UV spectrophotometry" to "UV–visible spectrophotometry" throughout the Methods and Supporting Information.

In addition, we carefully checked the formatting of chemical formulas, subscripts and superscripts throughout the manuscript. In particular, the equations and descriptions related to $^1O_2$ quantification have been revised to ensure correct notation of $^1O_2$, O_2, H_2O_2, •OH, electron numbers and charge units. We also standardized the formatting of potentials, units, ion formulas, isotope labels and electrochemical terms across the main text and Supporting Information.

Beyond the specific examples mentioned by the reviewer, we performed a full proofreading of the revised manuscript to correct non-idiomatic expressions, typographical errors and inconsistent terminology. We appreciate the reviewer's careful reading, which helped us improve the clarity and technical accuracy of the manuscript.

Manuscript Revision:

Norfloxacin concentration was determined by UV-visible spectrophotometry with a maximum absorption wavelength of λ=278 nm.

and the absorbance is quickly tested with a UV-visible spectrophotometer (UV-6100, Mapada).

The generation rate of $^1O_2$ during electrochemical O_2 activation is determined by FFA ($k_{^1O_2,\ FFA} = 1.2\times10^8\ M^{-1}s^{-1}$). We assume that the physical quenching of $^1O_2$ by FFA is negligible, and the steady-state concentration of $^1O_2$ can be calculated by the following equation:

$$\frac{d[FFA]}{dt} = -k_{O_{2,FFA}}[^1O_2]_{ss}[FFA],$$

$$[^1O_2]_{ss} = \frac{k_{FFA}}{k_{O_{2,FFA}}}.$$

Accordingly, the formation rate of $^1O_2$ in the electrocatalytic oxygen reduction process was calculated by the following equation:

$$R_{1O2,form} = [^1O_2]_0\left(k_d + k_{1O2,FFA}[FFA]\right)$$

where $k_d = 2.7\times10^4\ s^{-1}$ is the physical quenching rate constant by solvent (D_2O/H_2O = 0.95/0.05, $k_{d,\ H2O} = 2.5\times10^5\ s^{-1}$ and $k_{d,\ D2O} = 1.6\times10^4\ s^{-1}$).

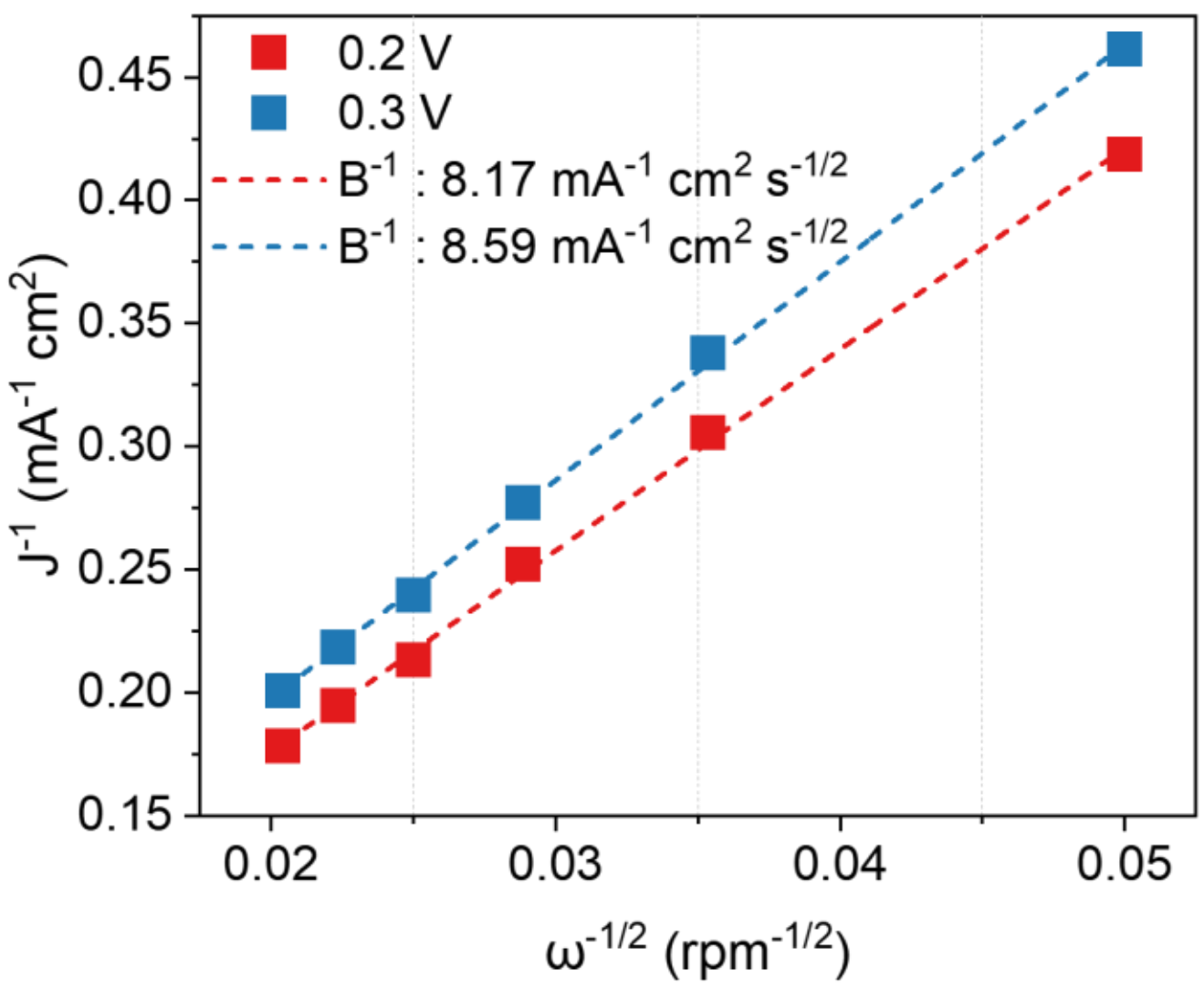

Supplementary Fig. 23 The corresponding Koutecky-Levich slope analysis for PSFZM.

Practical Decontamination Performance and System Engineering.

Referee: 2

Comments to the Author

Wang et al. address the severe kinetic and thermodynamic limitations associated with the conventional stepwise route, in which H_2O_2 is first generated via a two-electron oxygen reduction reaction ($2e^-$ ORR) and then further activated across different sites to produce •OH. In this manuscript, the authors propose an atomically defined, nanoscale perovskite active domain, $Pr_{1.0}Sr_{1.0}Fe_{0.5}Zn_{0.25}Mo_{0.25}O_{4-\delta}$ (PSFZM), that enables the direct, formal conversion of O_2 to •OH through a $3e^-$ ORR pathway, with the key feature being the confinement of intermediate stabilization and O-O bond cleavage within the same lattice-localized motif. The catalytic performance reported in the manuscript is also impressive, including a Faradaic efficiency (FE) toward •OH of up to 28.7 % in half-cell tests, an O_2-to-ROS conversion efficiency of 37.7 %, and a remarkably high •OH production rate. In addition, the work presents a flow reactor that directly uses gaseous oxygen instead of dissolved oxygen, further demonstrating the practical potential of this system for electrode-level scale-up and application. This is an interesting study with clear timeliness and a notable degree of originality. By constructing a crystalline framework featuring single-site active domains, the authors aim to circumvent the limitations of the currently prevalent tandem pathway. However, the experimental evidence provided by the combination of multiple characterization techniques is still insufficient to offer strong support for the proposed mechanistic role of the active domain, and several details require further clarification or additional evidence. Overall, I believe that after revision, this manuscript could be suitable for publication in Nature Communications.

We sincerely thank the reviewer for the positive and constructive evaluation of our manuscript. We are grateful that the reviewer recognizes the timeliness, originality, catalytic performance, and practical potential of our PSFZM system for direct oxygen-to-•OH conversion and flow-reactor applications. Following the reviewer's suggestion, we have carefully revised the manuscript to avoid overstatement and to present the mechanism more conservatively. We believe these revisions substantially improve the clarity, robustness, and mechanistic reliability of the work, and we sincerely appreciate the reviewer's constructive comments, which helped us strengthen the manuscript.
1. The current experimental results in the manuscript are more indicative of "suppressed

H2O2 accumulation and its facile subsequent conversion on PSFZM", but they do not strictly demonstrate that this process occurs within the same localized active domain, nor do they rigorously exclude short-range desorption/re-adsorption or interfacial secondary activation. The authors are encouraged to provide isotopic or transient spectroscopic evidence to verify that the local conversion rate of $*H_2O_2$ is faster than its desorption and diffusion.

Response: We thank the reviewer for this insightful and constructive comment. We agree that suppressed H_2O_2 accumulation alone is not sufficient to rigorously prove that peroxide conversion occurs within the same localized active domain. In the revised manuscript, we therefore added isotope-resolved and transient spectroscopic evidence to examine whether the local conversion of surface $*H_2O_2$ is faster than its desorption, diffusion and possible secondary interfacial activation.

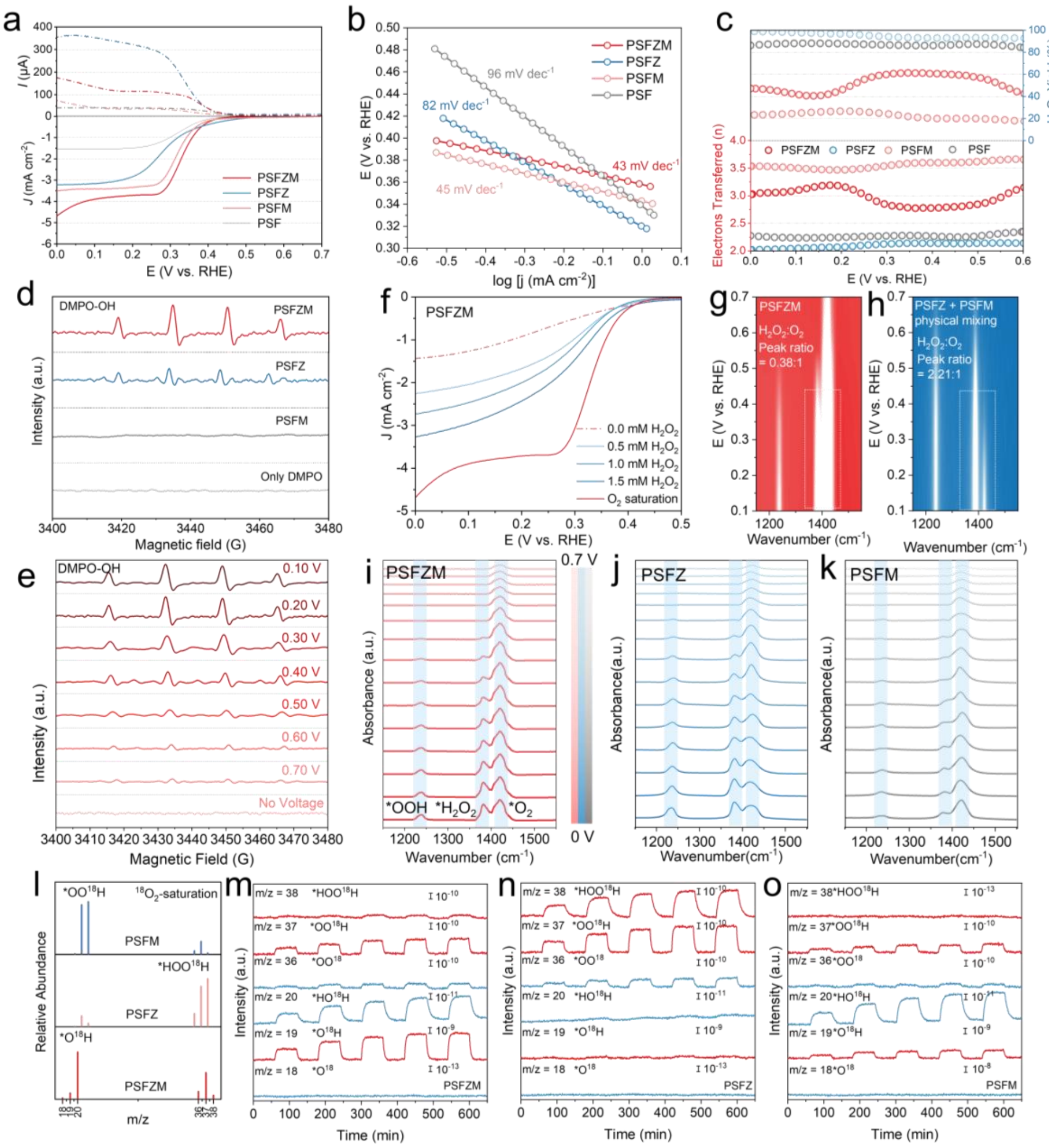

Fig. 3 Qualitative analysis of ORR selectivity and O_2 conversion to •OH. **a** RRDE polarization profiles for oxygen reduction reaction. Note: The solid lines are the disk current density corresponding to oxygen reduction and the dotted lines are the ring current density corresponding to H_2O_2 oxidation. **b** The corresponding Tafel plots. **c** H_2O_2 selectivity and number of transferred electrons (n) calculated from RRDE polarization curves. **d** EPR spectra of DMPO-•OH adducts for samples from different catalyst-catalyzed electro-Fenton reactions. **e** DMPO-•OH adduct EPR spectra of ORR electrochemistry over PSFZM catalyst collected in an incremental voltage configuration. **f** LSV with different concentrations of H_2O_2 raw materials in the O_2-free configuration and the O_2-filled configuration. Presented are the potential-dependent in situ FTIR contour plots of **g** PSFZM and the **h** PSFZ+PSFM physical mixture, acquired in an O_2-saturated electrolyte. The ORR electrochemical in-situ FTIR spectra of **i** PSFZM, **j** PSFZ, and **k** PSFM catalyst collected in an incremental voltage configuration. **l** Representative mass spectra of PSFZM, PSFZ, and PSFM, recorded under conditions saturated with $^{18}O_2$, reveal distinct ^{18}O-labeled oxygen reduction products. Time-resolved DEMS traces of selected isotope-labeled fragments, including peroxide/hydroperoxide-related channels and hydroxyl/water-related channels, during intermittent electrolysis over **m** PSFZM, **n** PSFZ, and **o** PSFM.

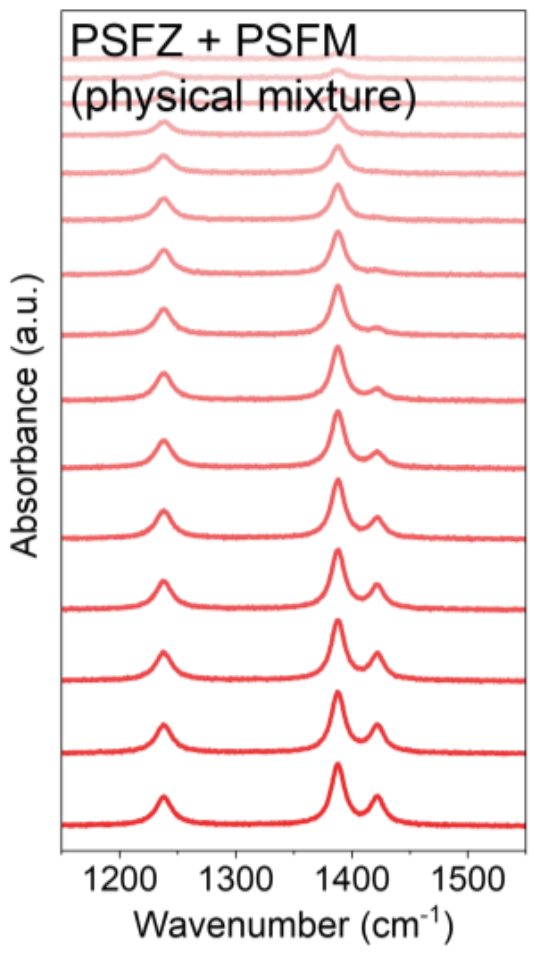

Supplementary Fig. 28 The ORR electrochemical in-situ FTIR spectra of PSFZ + PSFM physical mixture catalyst collected in an incremental voltage configuration.

Manuscript Revision:

To further substantiate this mechanism, in-situ Fourier-transform infrared spectroscopy (FT-IR) was used to clarify the evolution of reaction intermediates (**Figs. 3g–3k**). With

increasing cathodic bias, the *OOH and *H_2O_2 signals on PSFZ were markedly enhanced, indicating continuous H_2O_2 accumulation without subsequent activation (**Fig. 3j**). By contrast, PSFM exhibited only a weak *OOH signal and almost no detectable H_2O_2 feature, consistent with a four-electron (4e⁻) ORR pathway (**Fig. 3k**). In sharp contrast, PSFZM displayed an O-O stretching band of adsorbed O_2 at ~1424 cm^{-1}; as the potential shifted negatively, characteristic *OOH-related bands subsequently emerged at ~1241 and ~1389 cm^{-1} (**Fig. 3i**). Notably, the *OOH and *H_2O_2 species reached a dynamic formation–consumption equilibrium near 0.2 V. To more clearly distinguish continuous ORR evolution from spatially separated tandem evolution, PSFZM was compared with a physical mixture of PSFZ and PSFM. For PSFZM, the relative intensity ratio of the *H_2O_2-related band to the O_2/*OOH-related band was only 0.38:1, indicating that peroxide did not accumulate as the dominant surface species (**Fig. 3g**). The nearly six-fold enhancement of the peroxide-related signal in the PSFZ+PSFM physical mixture indicates substantial H_2O_2 accumulation, consistent with a conventional decoupled $2e^-$ + $1e^-$ tandem process (**Fig. 3h, Supplementary Fig. 28**). These results strongly support a confined active-domain-mediated continuous conversion pathway of O_2 → *OOH → *H_2O_2 → •OH, in which the B-site Zn centers serve as the key sites for direct O_2 activation during the $3e^-$ ORR process.

To resolve the operative ORR pathway on PSFZM, we performed isotope-resolved DEMS in $^{18}O_2$-saturated electrolyte. The relative mass spectral abundance distribution (**Fig. 3l**) shows that PSFM exhibits strong responses only at m/z = 18-20, corresponding to a typical $4e^-$ ORR pathway. In contrast, PSFZ displays synchronized signals at m/z = 37 and 38, while remaining silent at m/z = 19 and 20, indicating the direct desorption of *OOH as H_2O_2. Time-resolved DEMS measurements (**Figs. 3m-o**) further reveal that PSFZM exhibits synchronized and reversible responses across all four channels (m/z = 37, 38, 19, and 20) during each potential step. Compared with PSFZ, the H_2O_2 signal (m/z = 38) is markedly suppressed, whereas the *OH/ H_2O signals (m/z = 19/20) are simultaneously enhanced. In a conventional tandem mechanism involving H_2O_2 desorption–diffusion–re-adsorption, the m/z = 38 signal must precede the m/z = 19/20 signals and exhibit distinct relaxation times. Therefore, the strictly synchronized responses observed for PSFZM can only originate from the continuous reduction of surface-confined *H_2O_2 intermediates at the same active site.

2. The manuscript lacks EXAFS-derived coordination information for PSFZM, PSFZ, and

PSFM, including detailed metal–O bond lengths. It is recommended to summarize these parameters in a table.

Response: We thank the reviewer for this helpful suggestion. In the revised manuscript, we have added a summary table of the EXAFS-derived coordination parameters for PSFZM, PSFZ and PSFM. The table includes the fitted scattering paths, coordination numbers, metal–O bond lengths, Debye–Waller factors, ΔE_0 values and fitting R factors. In particular, the first-shell M–O bond distances obtained from the Zn K-edge, Mo K-edge and Fe K-edge fittings are now explicitly listed and compared among the three samples. These added parameters provide a clearer and more quantitative description of the local coordination environments and support the structural discussion in the revised manuscript.

Supplementary Table 2. EXAFS fitting parameters at the Zn K-edge for various samples.

Sample	Shell	Bond length (Å)	Coordination Number	σ^2 ($Å^2$)	E0 shift (eV)	R-factor
PSFZM	Zn-O	1.95±0.01	3.7±0.6	0.003±0.002	1.3±2.0	0.014
	Zn-O-Zn	3.28±0.01	3.1±0.4	0.011±0.008		
	Zn-O-M	3.88±0.01	5.0±0.6	0.016±0.010		
PSFZ	Zn-O	1.99±0.02	4.3±1.0	0.003±0.001	0.8±2.4	0.005
	Zn-Zn	2.97±0.01	6.0±1.6	0.012±0.004		
	Zn-O-Zn	3.39±0.01	3.0±0.9	0.011±0.004		
	Zn-O-M	3.91±0.03	12.3±0.8	0.007±0.004		

Note: The value of the amplitude reduction factor (S_0^2) was fixed to 0.70; Bond length is the interatomic distance; CN is the coordination number; σ^2 is Debye-Waller factor (a measure of thermal and static disorder in absorber scatter distance); E_0 shift is edge-energy shift (the difference between the zero kinetic energy value of the sample and that of the theoretical model); R factor is used to value the goodness of the fitting.

Supplementary Table 4. EXAFS fitting parameters at the Mo K-edge for various samples.

Sample	Shell	Bond length (Å)	Coordination Number	σ^2 (Å^2)	E0 shift(eV)	R-factor
PSFZM	Mo-O	1.77±0.01	4.0±0.2	0.001±0.001	6.7±0.9	0.012
	Mo-O-M	3.99±0.01	6.8±0.8	0.012±0.004		
PSFM	Mo-O	1.77±0.01	3.9±0.3	0.001±0.001	6.4±1.0	0.014
	Mo-O-M	3.98±0.02	5.6±0.6	0.011±0.004		

Note: The value of the amplitude reduction factor (S_0^2) was fixed to 0.95; Bond length is the interatomic distance; CN is the coordination number; σ^2 is Debye-Waller factor (a measure of thermal and static disorder in absorber scatter distance); E_0 shift is edge-energy shift (the difference between the zero kinetic energy value of the sample and that of the theoretical model); R factor is used to value the goodness of the fitting.

Supplementary Table 5. EXAFS fitting parameters at the Fe K-edge for various samples.

Sample	Shell	Bond length (Å)	Coordination Number	σ^2 (Å^2)	E0 shift (eV)	R-factor
PSFZM	Fe-O	1.95±0.02	4.2±0.8	0.003±0.002	-2.2±0.5	0.014
	Fe-Fe	3.00±0.04	3.8±0.5	0.005±0.002		
	Fe-O	3.44±0.04	3.7±0.9	0.012±0.006		
	Fe-O-M	3.80±0.04	6.2±1.0	0.010±0.006		
PSFZ	Fe-O	1.93±0.02	4.1±0.8	0.002±0.002	-3.2±2.4	0.008
	Fe-Fe	2.99±0.04	4.0±0.3	0.004±0.002		
	Fe-O	3.45±0.03	3.9±0.4	0.007±0.006		
	Fe-O-M	3.79±0.02	8.2±1.0	0.004±0.001		
PSFM	Fe-O	1.94±0.01	4.4±0.7	0.002±0.002	-3.1±1.9	0.006
	Fe-Fe	3.02±0.04	3.5±0.7	0.014±0.006		

	Fe-O	3.48±0.02	4.1±0.4	0.009±0.007		
	Fe-O-M	3.82±0.02	5.6±1.4	0.004±0.001		

Note: The value of the amplitude reduction factor (S_0^2) was fixed to 0.72; Bond length is the interatomic distance; CN is the coordination number; σ^2 is Debye-Waller factor (a measure of thermal and static disorder in absorber scatter distance); E_0 shift is edge-energy shift (the difference between the zero kinetic energy value of the sample and that of the theoretical model); R factor is used to value the goodness of the fitting.

Manuscript Revision:

The local coordination environment was probed by X-ray absorption fine structure (XAFS) spectroscopy. The Zn K-edge XANES spectrum of PSFZM exhibits an absorption edge positioned between those of Zn foil and ZnO, indicating a reduced Zn valence state ($Zn^{\delta+}$) relative to PSFZ (**Fig. 2j, Supplementary Fig. 12**). Consistently, the Zn–O coordination number decreases from 4.3 ± 1.0 in PSFZ to 3.7 ± 0.6 in PSFZM (**Supplementary Table 2**), corroborating this observation. Fourier-transformed EXAFS (FT-EXAFS) analyses further reveal a pronounced peak at ~2.48 Å in PSFZM, located between the Zn–Zn scattering of Zn foil (2.24 Å) and the Zn–O–Zn scattering of ZnO (2.98 Å) (**Supplementary Fig. 13**). This intermediate position indicates that the Zn-O-Zn motif in PSFZM does not arise from a continuous Zn-O-Zn-O aggregated network, but rather reflects a second-nearest-neighbor geometric correlation between Zn and the surrounding oxygen polyhedra. More importantly, PSFZM features a distinct Zn–O–M coordination shell with a bond length of 3.88 ± 0.01 Å, and the spectrum can be reproduced without invoking any direct Zn-Zn scattering contribution. This scattering path is absent in both PSFZ and the Zn reference samples (**Fig. 2k**), pointing to a stronger Zn-centred local ordering tendency in PSFZM[33]. Wavelet-transformed (WT) EXAFS contour plots (**Fig. 2l**) resolve the scattering contributions in the (k, R) plane and further substantiate this conclusion: PSFZM displays a low-k intensity maximum corresponding to Zn–O backscattering, together with a discrete high-k lobe at k ≈ 9 $Å^{-1}$ that is attributed to the heavier second-shell Zn–O–M path. Combined with the X-ray photoelectron spectroscopy (XPS) results, this direct structural evidence confirms the successful construction of atomic-scale Zn–O–M coupling within the perovskite matrix (**Supplementary Figs. 14-18**). Taken together, these structural analyses

verify that periodically modulated active domains have been successfully established in PSFZM.

Combining the Mo K-edge and Fe K-edge XANES spectra (**Figs. 4g-4j and Supplementary Figs 36-39**) with the WT-EXAFS spectra (**Figs. 4k,4l**) revealed that the chemical states and bond lengths of Mo-O and Fe-O in PSFZM are similar to those observed in PSFZ and PSFM. The bond lengths were determined to be 1.77 ± 0.01 Å for Mo-O and 1.95 ± 0.02 Å for Fe-O, indicating that the chemical states of Mo and Fe are unaffected by the special distorted Ruddlesden-Popper structure (**Supplementary Tables 4, 5, Supplementary Figs 40, 41**). The adsorption characteristics and intermediate evolution processes in PSFZM align with those in PSFZ and PSFM. This further supports the conclusion that Zn is the primary adsorption site in PSFZM, dominating the continuous activation of O_2 at this site.

3. The authors report n ≈ 3.0 for PSFZM in the potential range of 0.20–0.60 V and claim proximity to the theoretical diffusion-limited current for the 3e⁻ ORR. However, the Methods section indicates that RRDE data were not iR-compensated, and the expression of FE is itself unclear. The authors should clarify the calibration of collection efficiency, and provide error bars for n and H_2O_2 selectivity.

Response: We thank the reviewer for these valuable comments. In the revised manuscript, we have carefully revised the RRDE data processing, Methods section, and related figures to address these concerns. First, we clarified the iR compensation procedure. The uncompensated solution resistance was determined by electrochemical impedance spectroscopy, and the rotating ring-disk electrode (RRDE) potentials were corrected accordingly. All relevant data have now been replotted using iR-corrected potentials. We have also provided a detailed description of this correction in the Methods section. Second, we added a detailed calibration procedure for the RRDE collection efficiency, which was used for all RRDE calculations. The calibration curve and the corresponding description have been added to the Supplementary Information.

Manuscript Revision:

Supplementary Note 3. Calculation of apparent faraday efficiency.

The apparent Faradaic efficiency for cathodic •OH generation through the formal $3e^-$ ORR pathway was calculated as

$$FE_{\cdot OH} = \frac{3Fn_{\cdot OH}}{Q} \times 100\%,$$

where F is the Faraday constant, $n_{\bullet OH}$ is the amount of •OH determined from the BA calibration, and $Q = \int I\, dt$ is the total cathodic charge passed during electrolysis. For H_2O_2, the corresponding Faradaic efficiency was calculated as

$$FE_{H_2O_2} = \frac{2Fn_{H_2O_2}}{Q} \times 100\%.$$

For the paired full-cell test, the reported value is defined as an overall electron-to-•OH generation efficiency rather than a single-electrode Faradaic efficiency, because •OH can be generated through cathodic ORR and anodic water oxidation.

The oxygen-utilization efficiency was calculated from the oxygen balance:

$$\eta_{O_2 \to ROS} = \frac{n_{O_2,ROS}}{n_{O_2,consumed}} \times 100\%.$$

Here, $n_{O2,\ consumed}$ was determined from the decrease in dissolved O_2 after blank correction, and $n_{O2,\ ROS}$ was obtained by summing the O_2-equivalent molar amounts of detected •OH, H_2O_2 and $^1O_2$. The ROS selectivity was calculated as

$$S_i = \frac{n_{O_2,i}}{\sum n_{O_2,i}} \times 100\%.$$

Calibration curves were repeated at least three times. The limit of detection and limit of quantification were calculated as $3\sigma_{blank}/k$ and $10\sigma_{blank}/k$,respectively, where k is the slope of the calibration curve. Errors are reported as standard deviations from independent experiments, and propagated uncertainties were used for calculated efficiencies.

Supplementary Note 10. Calibration of the RRDE collection efficiency (N).

The collection efficiency N of the RRDE was experimentally calibrated using the $[Fe(CN)_6]^{3-/4-}$ redox couple in N_2-saturated electrolyte, following the protocol described previously[1]. The calibration electrolyte consisted of 10 mM $K_3[Fe(CN)_6]$ (≥99.0%, Sigma-Aldrich) prepared with ultrapure water (18.2 MΩ·cm, Milli-Q), and was deaerated by bubbling high-purity N_2 (≥99.999%) for at least 30 min prior to and throughout the measurements.

A three-electrode configuration was used, comprising a glassy carbon disk–platinum ring RRDE (disk diameter 4.0 mm; ring inner/outer diameter 5.0/7.0 mm; Pine Research) as the working electrode, a platinum wire as the counter electrode, and a saturated Hg/HgO electrode as the reference. Prior to each measurement, the glassy carbon disk was polished

sequentially with 1.0, 0.3 and 0.05 μm alumina slurries (Buehler) to a mirror finish, followed by ultrasonication in ultrapure water.

Measurements were performed at five rotation rates (ω = 400, 800, 1200, 1600 and 2000 rpm). The electrode was preconditioned with five cyclic voltammetric scans at each rotation rate to ensure a stable interface, and the limiting plateau currents for both the disk and ring were extracted for analysis.

The collection efficiency was calculated as:

$$N = -I_{ring} / I_{disk}$$

where I_{ring} is the limiting oxidation current at the ring and I_{disk} is the limiting reduction current at the disk.

4. The manuscript states that 86.7 % of consumed O_2 is converted to •OH, whereas the O_2-to-ROS conversion efficiency is only 37.7 %. This discrepancy is confusing and requires a clear definition of the denominator used in each metric.

Response: We thank the reviewer for pointing out this confusing description. We have revised the manuscript to clearly define the denominator used for each metric. The value of 86.7% refers to the proportion of quantified •OH among the total ROS components, which has now been explicitly clarified in the manuscript. In contrast, the O_2-to-ROS conversion efficiency of 37.7% was calculated using the total supplied/available O_2 as the denominator.

Manuscript Revision:

a comprehensive analysis of ROS distribution through free radical trapping revealed that the proportion of •OH was 86.7 %,

5. The claim of a "near-theoretical-rate" is insufficiently supported. The title directly asserts proximity to the theoretical rate limit, whereas the main text only states that the current at 0.07–0.10 V is "approaching the theoretical diffusion-limitation current for $3e^-$ ORR," without providing explicit calculations, boundary conditions, or error analysis for the theoretical limit.

Response: We appreciate the reviewers' valuable comments. We have further supplemented our analysis with an error assessment based on RRDE measurements, explicitly clarifying that this comparison specifically refers to the mass-transport-limited currents observed under the stated RRDE conditions, rather than universally applicable theoretical kinetic rate limits. These revisions provide a clearer and more rigorous basis for

our assertion. Additionally, we have included calculations for the theoretical $3e^-$ ORR diffusion-limited current in the Supporting Information.

Manuscript Revision:

The theoretical diffusion-limited current density for a formal $3e^-$ ORR process was calculated using the Levich equation:

$$j_L = 0.620 nFD_{O_2}^{2/3} \nu^{-1/6} C_{O_2} \omega^{1/2}$$

where j_L is the diffusion-limited current density, n is the number of electrons transferred per O_2 molecule, F is the Faraday constant, D_{O_2} is the diffusion coefficient of dissolved oxygen, ν is the kinematic viscosity of the electrolyte, C_{O_2} is the dissolved oxygen concentration, and ω is the angular rotation rate. For the RRDE measurement at 1600 rpm,

$$\omega = 2\pi \times \frac{1600}{60} = 167.6 \text{ rad s}^{-1}$$

Using the transport parameters for O_2-saturated 1.0 M Na_2SO_4 under our experimental conditions, the calculated theoretical diffusion-limited current density for a $3e^-$ ORR process is:

$$j_{L,3e^-} = 4.5 \text{ mA cm}^{-2}$$

The measured disk current density of PSFZM at 0.07–0.10 V versus RHE was 4.2 ± 0.2 mA cm^{-2}, corresponding to approximately 93 ± 5 % of the calculated $3e^-$ diffusion-limited current. Therefore, the phrase "approaching the theoretical limit" refers specifically to the calculated Levich mass-transfer limit under the defined RRDE conditions, not to an absolute theoretical limit for •OH generation.

6. The performance comparison in the manuscript uses inconsistent normalization standards for statements such as "highest reported" and "nearly 20 times higher than previously reported systems." Multiple normalization metrics are employed (e.g., 821 μmol h^{-1}, 228.06 μmol L^{-1} min^{-1}, 28.8 mol g cat^{-1} h^{-1}). A unified comparison basis should be adopted in both the main text and Supplementary Information to avoid distortion in cross-literature comparisons.

Response: We appreciate your valuable comments and constructive suggestions. We have carefully addressed your concerns, standardized the units throughout the entire text, and thoroughly revised the manuscript. Within the main text, the generation rate has been standardized to 821 μmol h^{-1}. To facilitate comparisons across different systems—including

those based on PMS and H_2O_2—we converted the rates to μmol L^{-1} min^{-1}, and we have clarified this conversion in the table footnotes.

Manuscript Revision:

As a result, the PSFZM electrode achieves a •OH generation rate as high as 821 μmol h^{-1}, nearly 20 times higher than previously reported systems. Moreover, by integrating this catalyst into a membrane-free flow electrolyzer, we demonstrate its scalability and exceptional performance in a real-world setting, achieving a •OH generation efficiency and rate of 64.7% and 28.8 mol g_{cat}^{-1} h^{-1}, respectively, at a high current density of ~200 mA cm^{-2}.

7. The manuscript first states that XRD confirms the retention of a Ruddlesden–Popper layered structure, but subsequently claims that TEM reveals an inverse perovskite configuration. This appears inconsistent and requires clarification.

Response: We appreciate your valuable comments and constructive suggestions. We have carefully addressed your concerns. We have ruled out the possibility of an anti-perovskite configuration and performed XRD refinement to confirm its Ruddlesden–Popper structure.

Manuscript Revision:

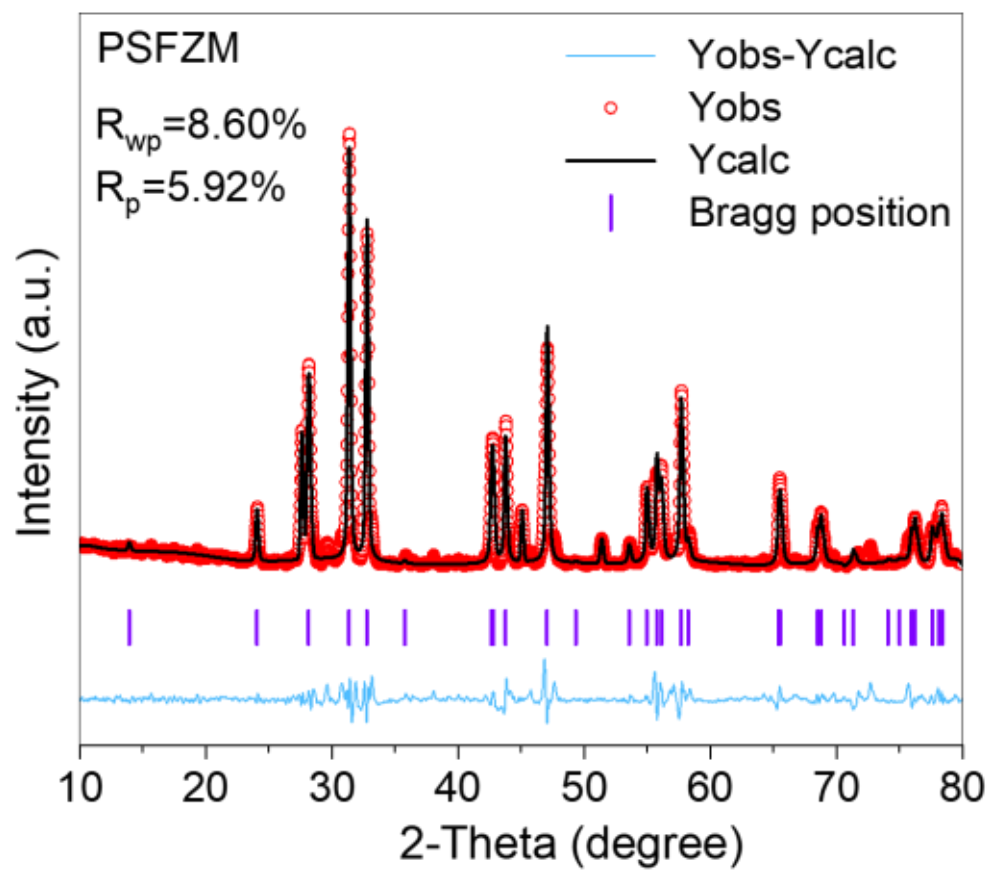

Supplementary Fig. 2. XRD Rietveld refinement of PSFZM.

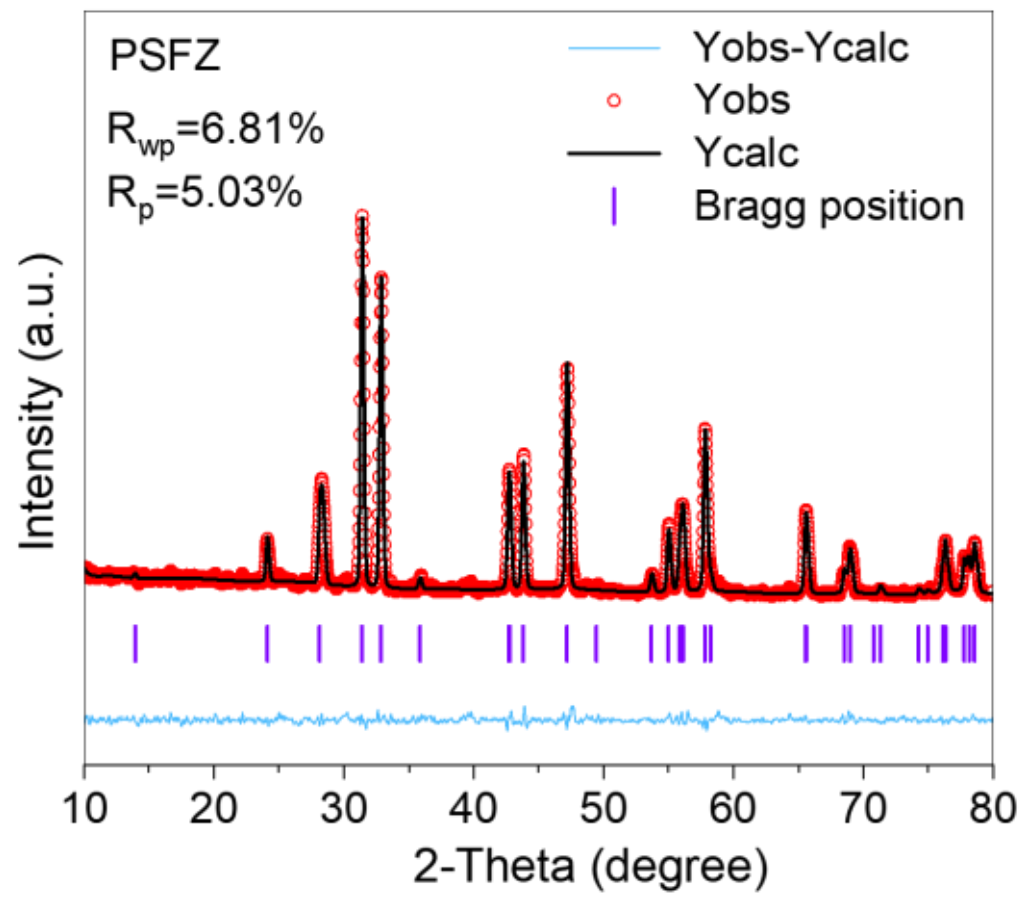

Supplementary Fig. 3. XRD Rietveld refinement of PSFZ.

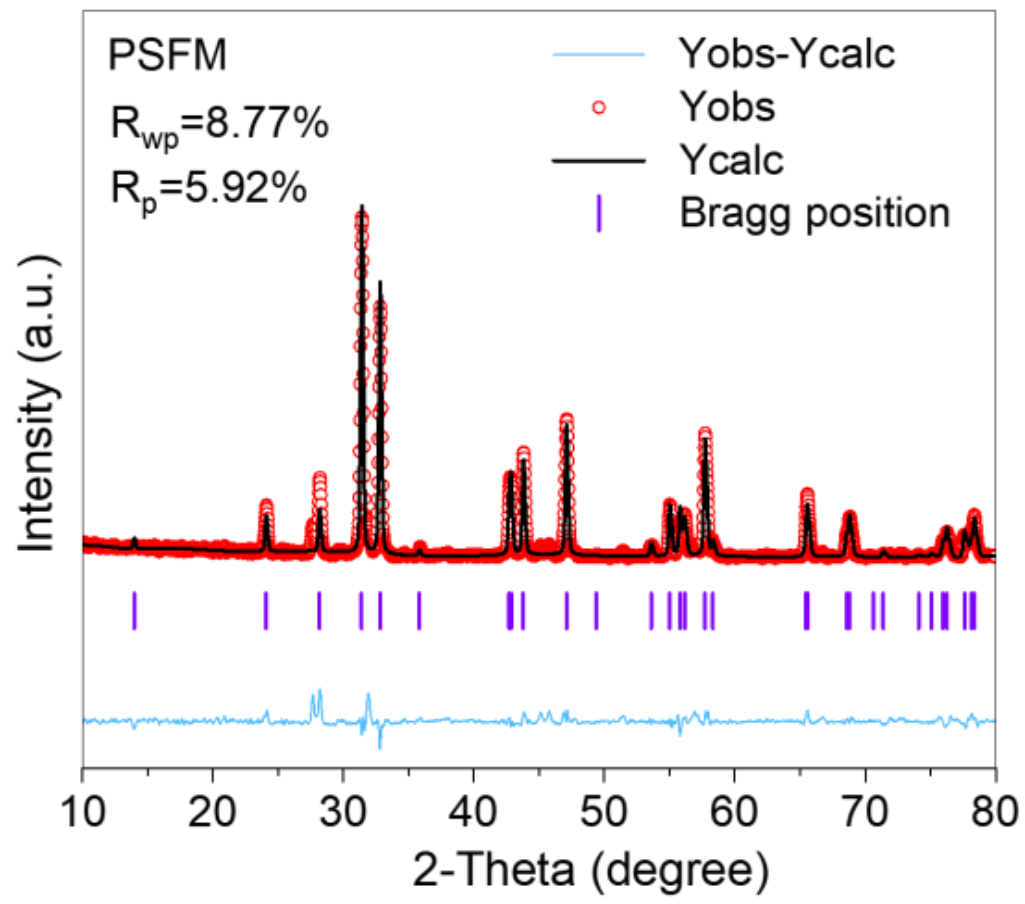

Supplementary Fig. 4. XRD Rietveld refinement of PSFM.

Rietveld-refined X-ray Diffraction (XRD) patterns confirm that PSFM, PSFZ, and PSFZM retain the tetragonal Ruddlesden-Popper structure with the I4/mmm space group (**Supplementary Figs. 2-4, Supplementary Table 1**). PSFZM exhibits the largest unit-cell volume and the most pronounced elongation of axial B–O bonds, accompanied by left-shifted diffraction peaks at ~32° and ~34° (**Fig. 2b**). These crystallographic features demonstrate that Zn/Mo co-doping induces lattice expansion and BO_6 octahedral distortion, supporting the formation of mixed-metal B–O active domains distinct from the single-metal local environments.

8. Although the device-level performance is impressive, the contributions of cathodic 3e⁻ ORR and anodic WOR in the full cell have not been clearly deconvoluted. The authors

should quantitatively distinguish the •OH contributions from the cathode and anode, and clearly define the denominator used in calculating the reported 64.7% FE.

Response: We appreciate your valuable comments and constructive suggestions. We have carefully checked your concerns. We performed electrode-resolved control experiments under otherwise identical electrolyte, current and sampling conditions. The cathodic contribution was evaluated using PSFZM as the cathode with an inert counter electrode, whereas the anodic contribution was quantified using PSFZM as the anode with an inert counter electrode. These measurements allowed us to separately determine the cathodic ORR-derived •OH generation and the anodic water-oxidation-derived •OH generation. The results confirm that both electrodes can contribute to ROS formation, but through different elementary charge-transfer processes.

Second, we added full-cell measurements in a separated configuration with independent catholyte and anolyte sampling. The •OH concentrations in the cathodic and anodic compartments were quantified separately using the same calibrated fluorescence method. The total •OH amount in the full cell was calculated as:

$$n_{\bullet\mathrm{OH,total}} = n_{\bullet\mathrm{OH,cathode}} + n_{\bullet\mathrm{OH,anode}}$$

where $n_{\bullet\mathrm{OH,cathode}}$ and $n_{\bullet\mathrm{OH,anode}}$ were obtained from the catholyte and anolyte, respectively. This electrode-resolved analysis allows the full-cell ROS balance to be constructed directly rather than inferred from half-cell data.

Third, we clarified the definition of Faradaic efficiency. The cathodic •OH formation is associated with the surface-confined 3e^{-} ORR pathway, whereas anodic •OH generation is treated as a 1e^{-} water/hydroxide oxidation process. Therefore, the apparent cathodic and anodic FEs were calculated as:

$$\mathrm{FE}_{\bullet\mathrm{OH,cathode}} = \frac{3Fn_{\bullet\mathrm{OH,cathode}}}{Q} \times 100\%$$

$$\mathrm{FE}_{\bullet\mathrm{OH,anode}} = \frac{Fn_{\bullet\mathrm{OH,anode}}}{Q} \times 100\%$$

For the full cell, the apparent paired FE for total •OH generation was defined as:

$$\mathrm{FE}_{\bullet\mathrm{OH,full-cell}} = \frac{F(3n_{\bullet\mathrm{OH,cathode}} + n_{\bullet\mathrm{OH,anode}})}{Q} \times 100\%$$

where Q is the total charge passed through the full cell. We now explicitly state that the reported peak value of 64.7% refers to this full-cell apparent paired FE for total probe-

accessible •OH, rather than a direct arithmetic comparison with either half-cell FE.

Finally, we revised the discussion of the full-cell results to avoid overinterpretation. The half-cell anodic FE of 58.9% and the full-cell peak FE of 64.7% are now presented as values obtained under different configurations and definitions. The full-cell FE is not treated as a simple sum of independently measured half-cell FEs, because radical recombination, interfacial quenching, mass transport and electrode coupling can all affect the observed ROS yield during paired operation. Instead, the revised manuscript reports the electrode-resolved cathodic and anodic contributions together with the full-cell species balance.

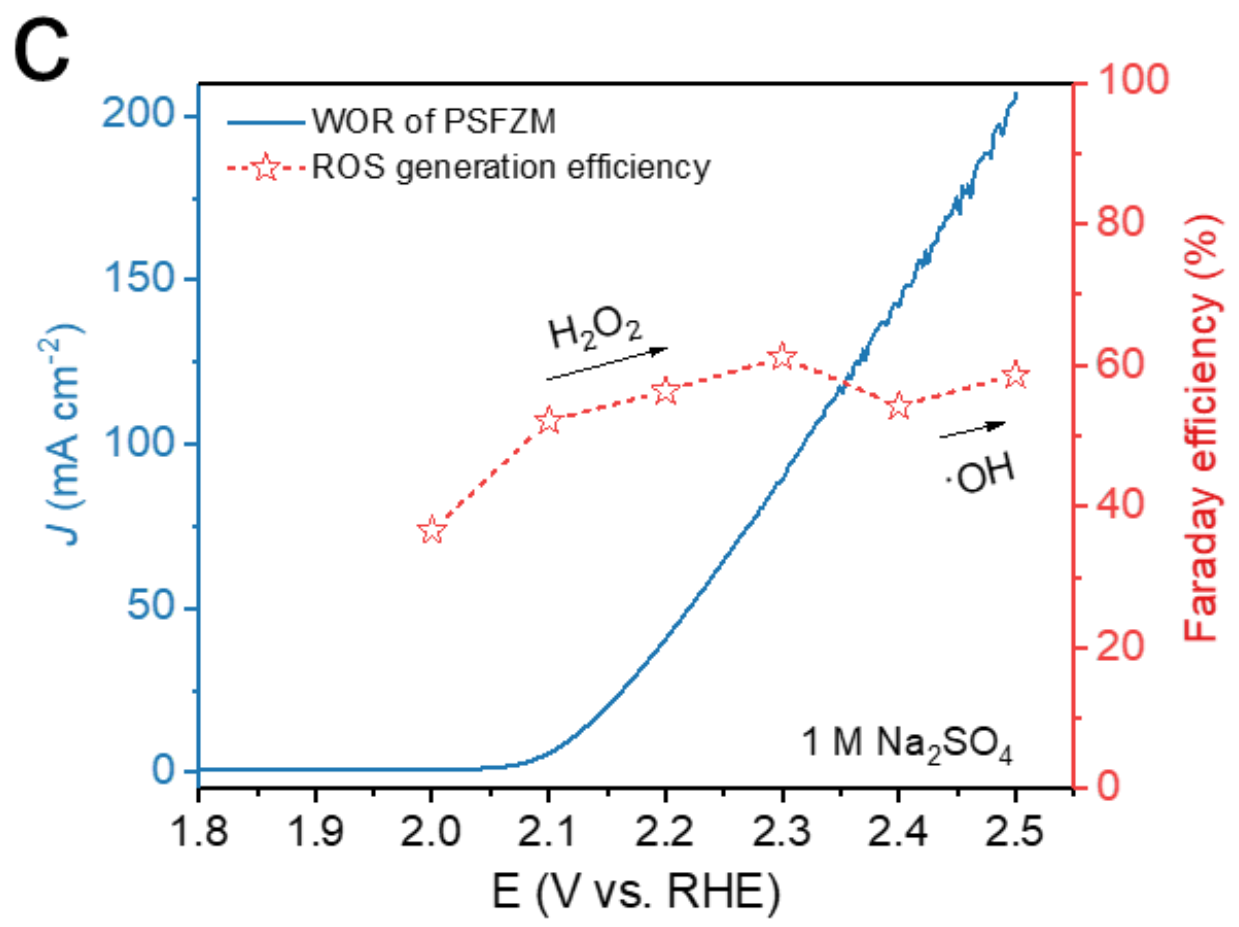

c Polarization I-V curves of PSFZM(WOR) measured in O_2-saturated 1.0 M Na_2SO_4, and the corresponding FE profiles obtained by the titration method.

Manuscript Revision:

First, we evaluate the WOR performance of PSFZM. DFT calculations reveal that PSFZM exhibits the lowest Gibbs free energy for the rate-determining step (ΔG_{RDS}) of the electron-driven WOR (**Fig. 6b**). Notably, the $\Delta G_{*OH} > 2.4$ eV suggests weak *OH binding on the catalyst surface, suppressing the evolution of H_2O_2 or O_2 and instead favoring •OH production. Electrochemical measurements under 1.0 M Na_2SO_4 further corroborate these findings, with FE for •OH formation exceeding 50% at >2.0 V and a peak FE_{anode} of 58.9% achieving at ~2.5 V, alongside a high current density of ~200 mA cm^{-2} (**Figs. 6c and Supplementary Fig. 88**). Chronoamperometric stability tests further validate the system's scalability and robustness for sustained •OH generation.

In this setup, the $3e^-$ ORR || e^- WOR full-cell system delivered a remarkable peak Faradaic

efficiency of 64.7% for •OH generation (**Fig. 6c, d**).

9. In the Methods section, the FE formula for the RRDE analysis is not written in a sufficiently standard form. The authors are advised to recheck the expression and clearly define the charge meaning represented by the numerator and denominator.

Response: We appreciate your valuable comments and constructive suggestions. We agree that the original expression for the Faradaic-efficiency calculation in the RRDE analysis was not sufficiently clear and could cause ambiguity regarding the physical meaning of the numerator and denominator.

In the revised Methods section, we have rewritten the RRDE-related formulae in a standard form and explicitly defined all parameters. Specifically, the ring current was first corrected by the experimentally determined collection efficiency, N, to obtain the amount of peroxide generated at the disk. The Faradaic efficiency/selectivity toward H_2O_2 was then calculated by comparing the charge associated with H_2O_2 oxidation at the ring, corrected by N, with the total cathodic charge passed at the disk. The numerator therefore represents the equivalent charge corresponding to H_2O_2 generated through the two-electron ORR pathway, whereas the denominator represents the total ORR charge consumed at the disk electrode.

Manuscript Revision:

Supplementary Note 3. Calculation of apparent faraday efficiency.

The apparent Faradaic efficiency for cathodic •OH generation through the formal $3e^-$ ORR pathway was calculated as

$$FE_{\cdot OH} = \frac{3Fn_{\cdot OH}}{Q} \times 100\%,$$

where F is the Faraday constant, $n_{\cdot OH}$ is the amount of •OH determined from the BA calibration, and $Q = \int I\, dt$ is the total cathodic charge passed during electrolysis. For H_2O_2, the corresponding Faradaic efficiency was calculated as

$$FE_{H_2O_2} = \frac{2Fn_{H_2O_2}}{Q} \times 100\%.$$

For the paired full-cell test, the reported value is defined as an overall electron-to-•OH generation efficiency rather than a single-electrode Faradaic efficiency, because •OH can be generated through cathodic ORR and anodic water oxidation.

The oxygen-utilization efficiency was calculated from the oxygen balance:

$$\eta_{O_2 \rightarrow ROS} = \frac{n_{O_2,ROS}}{n_{O_2,consumed}} \times 100\%.$$

Here, $n_{O_2,\mathrm{consumed}}$ was determined from the decrease in dissolved O_2 after blank correction, and $n_{O_2,\mathrm{ROS}}$ was obtained by summing the O_2-equivalent molar amounts of detected •OH, H_2O_2 and $^1O_2$. The ROS selectivity was calculated as

$$S_i = \frac{n_{O_2,i}}{\sum n_{O_2,i}} \times 100\%.$$

Calibration curves were repeated at least three times. The limit of detection and limit of quantification were calculated as $3\sigma_{\mathrm{blank}}/k$ and $10\sigma_{\mathrm{blank}}/k$,respectively, where k is the slope of the calibration curve. Errors are reported as standard deviations from independent experiments, and propagated uncertainties were used for calculated efficiencies.

10. There is a repeated subsection title in the Results section: "Practical Decontamination Performance and System Engineering. Actual Decontamination Performance and System Engineering." One of them should be removed.

Response: We appreciate your valuable comments and constructive suggestions. We have carefully checked your concerns. We have removed the subsection headings and retained "Practical Decontamination Performance and System Engineering."

Manuscript Revision:

Practical Decontamination Performance and System Engineering.

11. The correspondence between figure panels and the main text is not sufficiently rigorous in several places. For example, in Fig. 4, the panel indices are cross-referenced inconsistently in the main text as 4g, 4i and 4i, 4j. A thorough check of the figure–text correspondence is recommended.

Response: We appreciate your valuable comments and constructive suggestions. We agree that accurate correspondence between figure panels and the main text is essential for the clarity and rigor of the manuscript.

In the revised manuscript, we have thoroughly checked all figure–text cross-references, with particular attention to Fig. 4. The inconsistent panel citations in the main text, including the incorrect references to Fig. 4g, 4i and Fig. 4i, 4j, have been corrected to match the corresponding figure panels and captions. We have also carefully reviewed all other figures, supplementary figures, tables, and captions throughout the manuscript to ensure that each panel is cited in the correct order and corresponds accurately to the related discussion in the text.

Manuscript Revision:

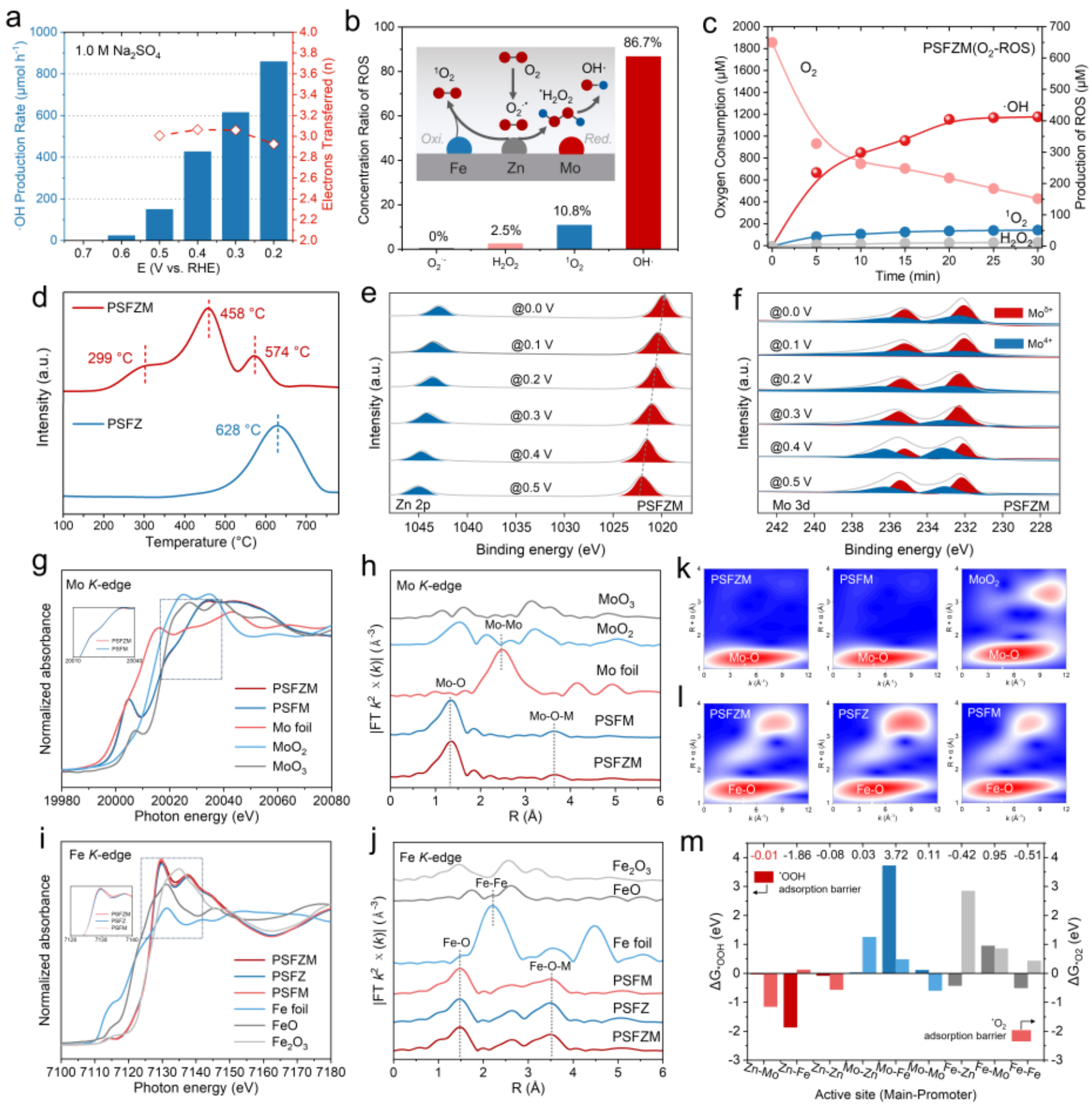

Fig. 4 Mechanism of dual co-catalytic sites promoting ROS formation. a Benzoic acid fluorescence spectrophotometry, used to detect the amount of •OH free radicals generated. •OH yield at different oxygen reduction voltages. **b** The proportion of ROS generated by PSFZM through 3e⁻ ORR, and the inset shows the single-site active domain mechanism. **c** Oxygen utilization during 30-min continuous testing. Quantitative determination of the consumed dissolved O_2 and generated ROS. **d** O_2-TPD profiles of PSFZM and PSFZ. In-situ XPS spectra of **e** Zn 2p and **f** Mo 3d in electrochemistry. **g** Mo K-edge XANES spectra of PSFZM, PSFM, Mo foil, MoO_2 and MoO_3. **h** Mo K-edge k^3-weighted FT-EXAFS spectra. **i** Fe K-edge XANES spectra of PSFZM, PSFZ, PSFM, Fe foil, FeO and Fe_2O_3. **j** Fe K-edge k^3-weighted FT-EXAFS spectra. WT-EXAFS spectra of **k** Mo K-edge and **l** Fe K-edge. **m** Comparison of free energies of adsorption of intermediate *OOH and O_2 at B sites in PSFZM.

12. There are numbering errors in the caption of Fig. 4. The caption first states "g Mo K-

edge ... h Mo K-edge ... k WT-EXAFS ...", but later also states "g Fe K-edge ... h Fe K-edge ... k WT-EXAFS ...". The authors should carefully correct these errors.
**Response**: We appreciate your valuable comments and constructive suggestions. We have carefully checked your concerns. We have conducted a comprehensive review and revision of the captions.   **Manuscript Revision**:   **Fig. 4 Mechanism of dual co-catalytic sites promoting ROS formation. a** Benzoic acid fluorescence spectrophotometry, used to detect the amount of •OH free radicals generated. •OH yield at different oxygen reduction voltages. **b** The proportion of ROS generated by PSFZM through $3e^-$ ORR, and the inset shows the single-site active domain mechanism. **c** Oxygen utilization during 30-min continuous testing. Quantitative determination of the consumed dissolved O_2 and generated ROS. **d** O_2-TPD profiles of PSFZM and PSFZ. In-situ XPS spectra of **e** Zn 2p and **f** Mo 3d in electrochemistry. **g** Mo K-edge XANES spectra of PSFZM, PSFM, Mo foil, MoO_2 and MoO_3. **h** Mo K-edge k^3-weighted FT-EXAFS spectra. **i** Fe K-edge XANES spectra of PSFZM, PSFZ, PSFM, Fe foil, FeO and Fe_2O_3. **j** Fe K-edge k^3-weighted FT-EXAFS spectra. WT-EXAFS spectra of **k** Mo K-edge and **l** Fe K-edge. **m** Comparison of free energies of adsorption of intermediate *OOH and O_2 at B sites in PSFZM.
13. The phrase "classical spinning method" in the caption of Fig. 2 is not sufficiently precise. The Methods section describes the synthesis as electrospinning, whereas the figure caption uses "classical spinning method." The terminology should be unified as electrospinning.
**Response**: We appreciate your valuable comments and constructive suggestions. We have carefully checked your concerns. We have comprehensively revised the description.   **Manuscript Revision**:   **a** Schematic diagram showing the preparation of PSFZM electrodes by the electrospinning method. Colors represent: Pr (red), Sr (purple), Fe (yellow), Zn (green), Mo (blue).
14. In Section 6 of the manuscript, the expressions "membrane-free cylindrical electrolyzer" and later "membraneless flow-through electrolyzer" are inconsistent. This should be carefully corrected.
**Response**: We appreciate your valuable comments and constructive suggestions. We

have carefully checked your concerns. We have comprehensively revised the description.

Manuscript Revision:

The PSFZM electrode achieved an ROS production rate of 28.8 mol g_{cat}^{-1} h^{-1} in a membrane-free cylindrical electrolytic cell—a value ranking among the highest currently reported across all types of electrochemical systems.

15. The phrase "dominating the continuous evolution of O_2 at this site" is not accurate in English. What is intended here should be O_2 activation/conversion rather than "evolution of O_2," since in electrocatalysis "evolution" is usually understood as O_2 evolution.

Response: We appreciate your valuable comments and constructive suggestions. We have carefully checked your concerns. We have comprehensively revised the description.

Manuscript Revision:

Fig 1. Intermediate recognition strategy promotes •OH generation. a The evolution of O_2 activation in a classic multiphase catalytic system. Long pathway resulting from the migration of intermediates from the $Metal_1$ site to the $Metal_2$ site. **b** The evolution of O_2 activation in the single-site active domain catalytic system. The intermediate completes the adsorption, protonation and O–O bond cleavage process in the domain.

Referee: 3

Comments to the Author

This manuscript reports a single-atom engineered perovskite catalyst (PSFZM) that is claimed to promote an efficient formal 3-electron oxygen reduction reaction ($3e^-$ ORR), enabling direct and selective electrogeneration of hydroxyl radicals (•OH). The central design concept is an atomically confined active domain with a Zn-O-Mo structural motif, which is proposed to stabilize key oxygenated intermediates and facilitate O-O bond cleavage, thereby bypassing the kinetic constraints of conventional stepwise pathways. The authors further report high •OH production, favorable oxygen utilization, effective degradation of organic pollutants, and compatibility with a membrane-free flow electrolyzer for sustained water treatment. However, the current evidence base contains multiple critical gaps in mechanistic proof, incomplete electrochemical quantification, and insufficient controls, so the work requires major revision.

We sincerely thank the reviewer for the careful evaluation and constructive comments. We appreciate the reviewer's recognition of the originality and potential significance of our single-atom-engineered PSFZM catalyst, particularly the concept of a lattice-confined Zn–O–Mo active domain for promoting selective oxygen activation and efficient •OH electrogeneration. At the same time, we fully acknowledge the reviewer's concerns regarding mechanistic proof, electrochemical quantification, and the need for additional control experiments. In response, we have comprehensively revised the manuscript to improve the rigor, clarity, and reliability of the work.
1. The absorption spectrum does not present the fitting results; instead, it should be reported in a table including the coordination number, bond length, and correction factors for different samples. Therefore, the Zn-O-Fe and Zn-O-Mo coordination environments inferred from the wavelet transform remain questionable, as atom-level evidence is insufficient. Moreover, in the in situ XPS data, the Mo 3d, Mo K-edge, and Fe K-edge absorption edges indicate that the PSFZ, PSFM, and PSFZM catalysts are essentially indistinguishable. What, then, is the specific role of Fe and Mo? In a comparison between PAFZ and PSFZM, one would expect

the Mo effect to be more clearly elucidated.

Response: We appreciate your valuable comments and constructive suggestions. We agree that the original XAFS analysis was not sufficiently quantitative and that the Zn–O–Fe/Zn–O–Mo assignments should not rely only on wavelet-transform features. In the revised manuscript, we have therefore added detailed EXAFS fitting results for PSFZ, PSFM and PSFZM in a new Supplementary Table, including the fitted scattering paths, coordination numbers, bond lengths, Debye–Waller factors, ΔE_0 correction values and R factors. The corresponding fitting curves in both R space and k space have also been added to the Supporting Information.

We have also revised the discussion of the wavelet-transform analysis. The WT results are now used only as supporting evidence to distinguish different backscattering atoms, whereas the coordination assignments are based on quantitative EXAFS fitting and model comparison. In particular, control fitting models without Zn–O–Mo or Mo–O–Zn paths give poorer agreement with the experimental spectra, supporting the presence of a mixed Zn–O–M local coordination environment in PSFZM. We have removed overly definitive statements that were not directly supported by the fitting results.

Regarding the reviewer's concern that Mo 3d XPS, Mo K-edge and Fe K-edge features appear similar among PSFZ, PSFM and PSFZM, we have clarified that these spectra mainly reflect the average oxidation state and average electronic environment of Mo or Fe. The similar edge positions indicate that no obvious bulk phase segregation or drastic valence change occurs after Zn/Mo incorporation. However, the catalytic difference originates from the local coordination environment rather than a large change in the average oxidation state. This is now explicitly stated in the revised manuscript.

We further clarified the specific roles of Fe and Mo. Fe constitutes the main B-site framework of the Ruddlesden–Popper lattice and helps maintain the electronic/structural environment for the ORR, but Fe-centred motifs alone cannot effectively promote the desired $3e^-$ pathway. Zn preferentially stabilizes the $*OOH/*H_2O_2$ intermediates, whereas the adjacent Mo provides stronger oxophilic interactions and alters the adsorption configuration of $*H_2O_2$ at the Zn site, making the O–O bond more readily polarized and thereby facilitating its subsequent conversion to •OH. The comparison among PSFZ, PSFM and PSFZM supports this cooperative Zn–O–Mo effect: PSFZ favours $*H_2O_2$ accumulation, PSFM tends

toward deeper reduction, whereas PSFZM exhibits suppressed H_2O_2 accumulation and enhanced •OH generation.

In response to the reviewer's suggestion, we have also strengthened the comparison between the Mo-free analogue and PSFZM. The added electrochemical, spectroscopic and DFT/CI-NEB analyses show that introducing Mo into the Zn-containing lattice lowers the barrier for peroxide activation/O–O bond cleavage and shifts the pathway from $*H_2O_2$ accumulation toward surface-confined consecutive $3e^-$ ORR to •OH. These revisions more clearly define the role of Mo and provide a more rigorous structural basis for the proposed Zn–O–Mo active domain.

Manuscript Revision:

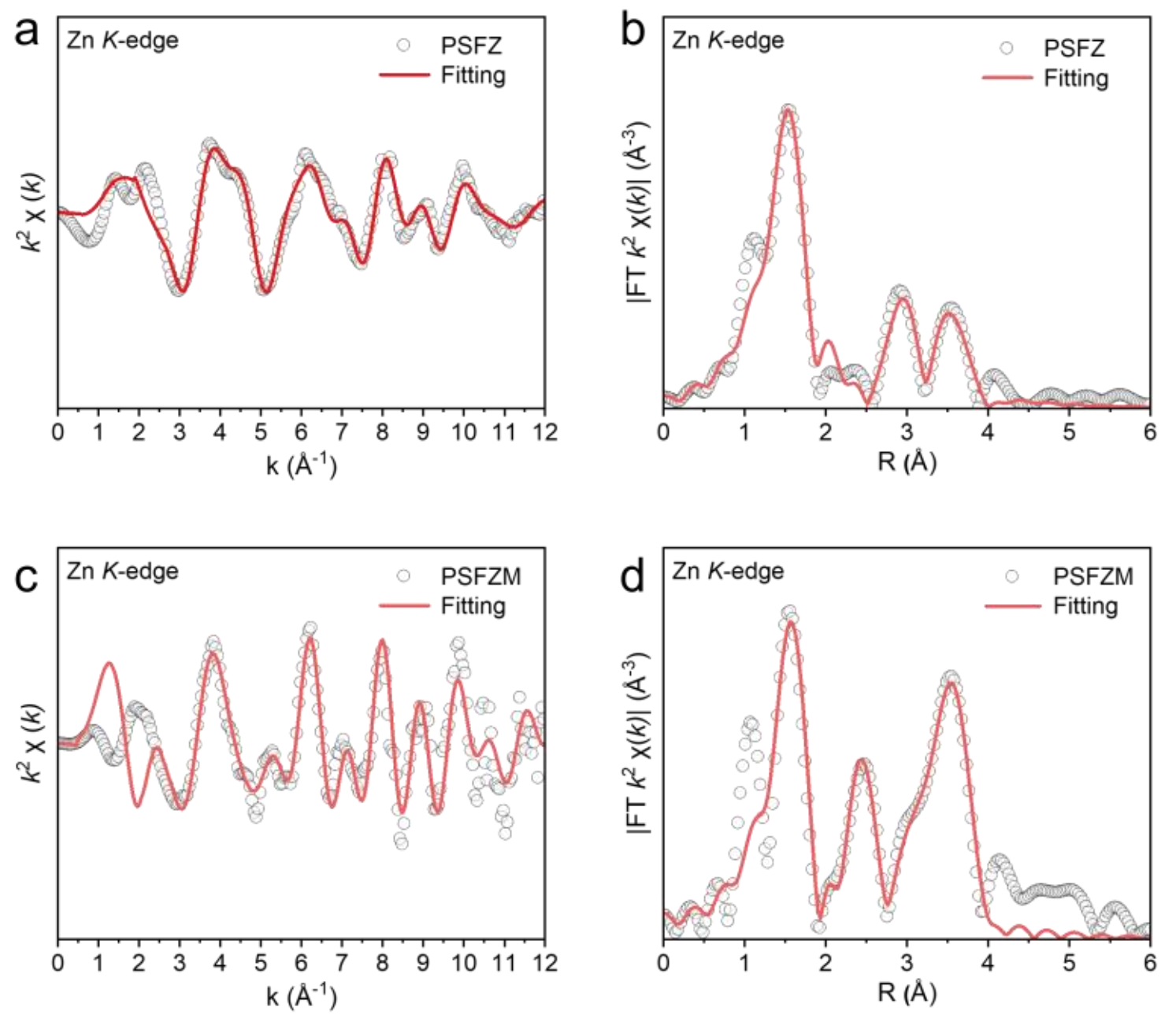

Supplementary Fig. 13 Fitting of the Zn K-edge EXAFS K spectra for **a** PSFZ and **c** PSFZM. Fitting of the Zn K-edge EXAFS R space fitting result for **b** PSFZ and **d** PSFZM.

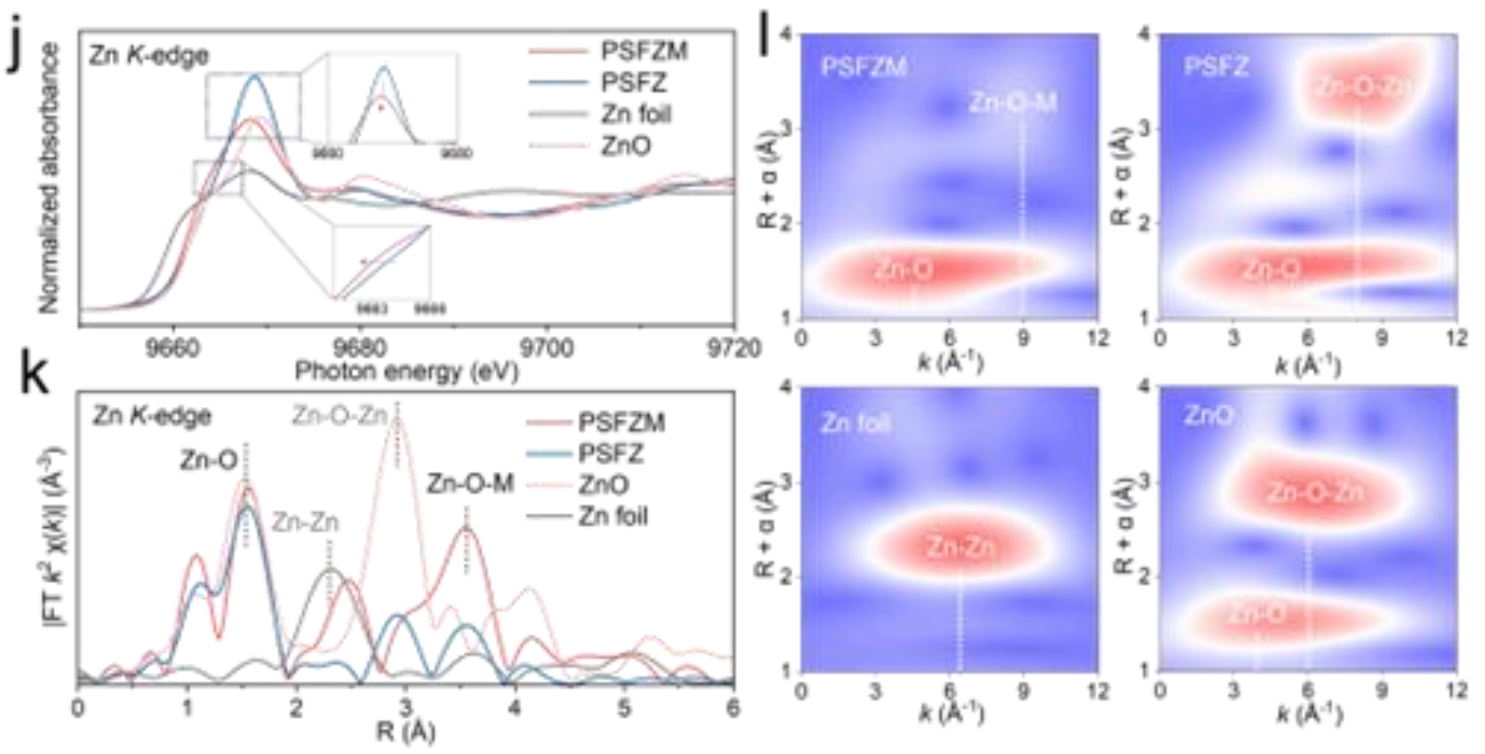

j Zn K-edge XANES spectra of PSFZM, PSFZ, Zn foil, ZnO. **k** Zn K-edge k^3-weighted FT-EXAFS spectra and **l** WT-EXAFS spectra of PSFZM, PSFZ, Zn foil, ZnO.

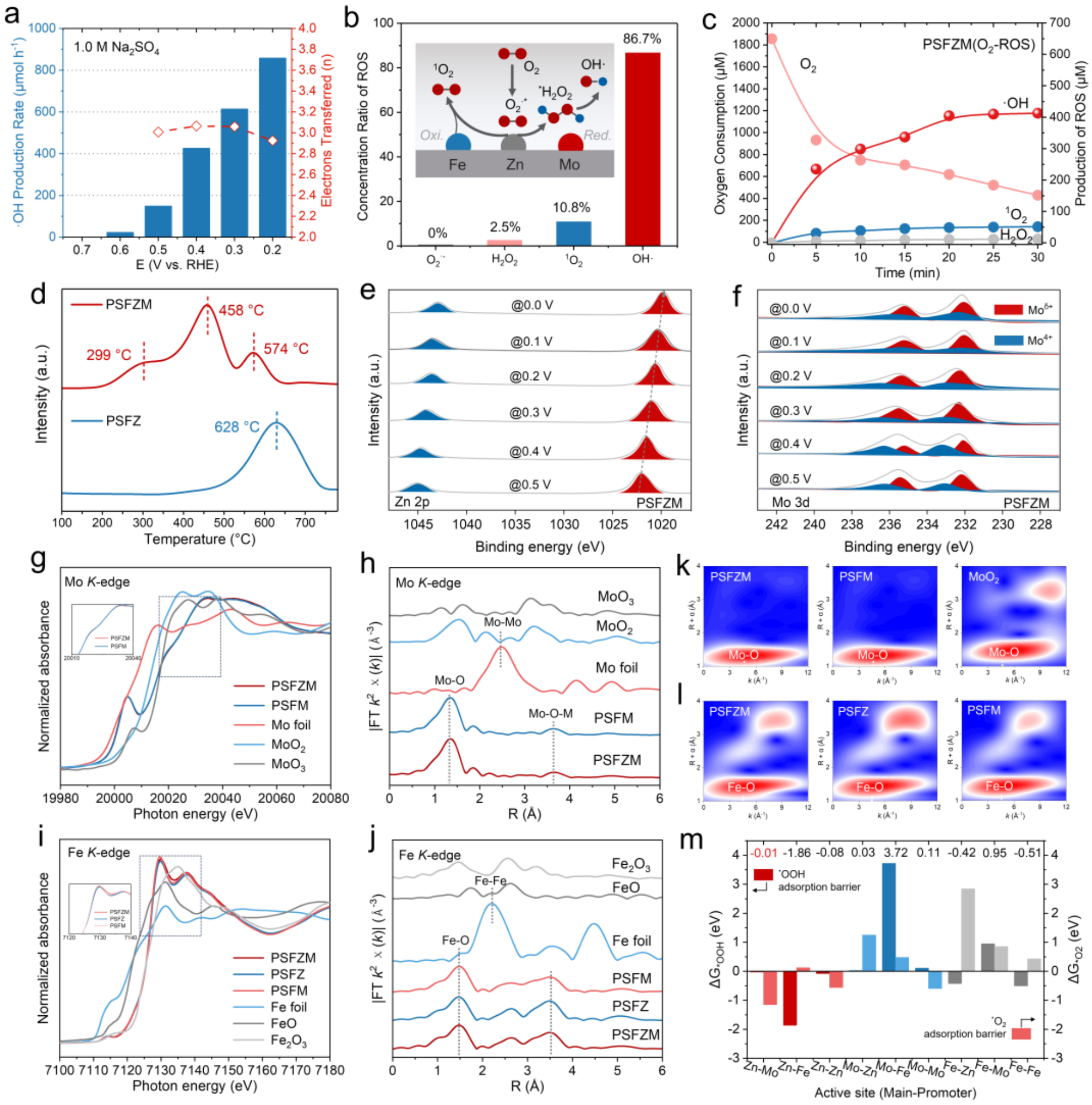

Fig. 4 Mechanism of dual co-catalytic sites promoting ROS formation. a Benzoic acid fluorescence spectrophotometry, used to detect the amount of •OH free radicals generated. •OH yield at different oxygen reduction voltages. **b** The proportion of ROS generated by

PSFZM through 3e⁻ ORR, and the inset shows the single-site active domain mechanism. **c** Oxygen utilization during 30-min continuous testing. Quantitative determination of the consumed dissolved O_2 and generated ROS. **d** O_2-TPD profiles of PSFZM and PSFZ. In-situ XPS spectra of **e** Zn 2p and **f** Mo 3d in electrochemistry. **g** Mo K-edge XANES spectra of PSFZM, PSFM, Mo foil, MoO_2 and MoO_3. **h** Mo K-edge k^3-weighted FT-EXAFS spectra. **i** Fe K-edge XANES spectra of PSFZM, PSFZ, PSFM, Fe foil, FeO and Fe_2O_3. **j** Fe K-edge k^3-weighted FT-EXAFS spectra. WT-EXAFS spectra of **k** Mo K-edge and **l** Fe K-edge. **m** Comparison of free energies of adsorption of intermediate *OOH and O_2 at B sites in PSFZM.

Supplementary Table 2. EXAFS fitting parameters at the Zn K-edge for various samples.

Sample	Shell	Bond length (Å)	Coordination Number	σ^2 (Å^2)	E0 shift (eV)	R-factor
**PSFZM**	Zn-O	1.95±0.01	3.7±0.6	0.003±0.002	1.3±2.0	0.014
	Zn-O-Zn	3.28±0.01	3.1±0.4	0.011±0.008		
	Zn-O-M	3.88±0.01	5.0±0.6	0.016±0.010		
**PSFZ**	Zn-O	1.99±0.02	4.3±1.0	0.003±0.001	0.8±2.4	0.005
	Zn-Zn	2.97±0.01	6.0±1.6	0.012±0.004		
	Zn-O-Zn	3.39±0.01	3.0±0.9	0.011±0.004		
	Zn-O-M	3.91±0.03	12.3±0.8	0.007±0.004		

Note: The value of the amplitude reduction factor (S_0^2) was fixed to 0.70; Bond length is the interatomic distance; CN is the coordination number; σ^2 is Debye-Waller factor (a measure of thermal and static disorder in absorber scatter distance); E_0 shift is edge-energy shift (the difference between the zero kinetic energy value of the sample and that of the theoretical model); R factor is used to value the goodness of the fitting.

Supplementary Table 4. EXAFS fitting parameters at the Mo K-edge for various samples.

Sample	Shell	Bond length (Å)	Coordination Number	σ^2 (Å^2)	E0 shift(eV)	R-factor
PSFZM	Mo-O	1.77±0.01	4.0±0.2	0.001±0.001	6.7±0.9	0.012
	Mo-O-M	3.99±0.01	6.8±0.8	0.012±0.004		
PSFM	Mo-O	1.77±0.01	3.9±0.3	0.001±0.001	6.4±1.0	0.014
	Mo-O-M	3.98±0.02	5.6±0.6	0.011±0.004		

Note: The value of the amplitude reduction factor (S_0^2) was fixed to 0.95; Bond length is the interatomic distance; CN is the coordination number; σ^2 is Debye-Waller factor (a measure of thermal and static disorder in absorber scatter distance); E_0 shift is edge-energy shift (the difference between the zero kinetic energy value of the sample and that of the theoretical model); R factor is used to value the goodness of the fitting.

Supplementary Table 5. EXAFS fitting parameters at the Fe K-edge for various samples.

Sample	Shell	Bond length (Å)	Coordination Number	σ^2 (Å^2)	E0 shift (eV)	R-factor
PSFZM	Fe-O	1.95±0.02	4.2±0.8	0.003±0.002	-2.2±0.5	0.014
	Fe-Fe	3.00±0.04	3.8±0.5	0.005±0.002		
	Fe-O	3.44±0.04	3.7±0.9	0.012±0.006		
	Fe-O-M	3.80±0.04	6.2±1.0	0.010±0.006		
PSFZ	Fe-O	1.93±0.02	4.1±0.8	0.002±0.002	-3.2±2.4	0.008
	Fe-Fe	2.99±0.04	4.0±0.3	0.004±0.002		
	Fe-O	3.45±0.03	3.9±0.4	0.007±0.006		
	Fe-O-M	3.79±0.02	8.2±1.0	0.004±0.001		
PSFM	Fe-O	1.94±0.01	4.4±0.7	0.002±0.002	-3.1±1.9	0.006
	Fe-Fe	3.02±0.04	3.5±0.7	0.014±0.006		

	Fe-O	3.48±0.02	4.1±0.4	0.009±0.007		
	Fe-O-M	3.82±0.02	5.6±1.4	0.004±0.001		

Note: The value of the amplitude reduction factor (S_0^2) was fixed to 0.72; Bond length is the interatomic distance; CN is the coordination number; σ^2 is Debye-Waller factor (a measure of thermal and static disorder in absorber scatter distance); E_0 shift is edge-energy shift (the difference between the zero kinetic energy value of the sample and that of the theoretical model); R factor is used to value the goodness of the fitting.

The local coordination environment was probed by X-ray absorption fine structure (XAFS) spectroscopy. The Zn K-edge XANES spectrum of PSFZM exhibits an absorption edge positioned between those of Zn foil and ZnO, indicating a reduced Zn valence state ($Zn^{\delta+}$) relative to PSFZ (**Fig. 2j, Supplementary Fig. 12**). Consistently, the Zn–O coordination number decreases from 4.3 ± 1.0 in PSFZ to 3.7 ± 0.6 in PSFZM (**Supplementary Table 2**), corroborating this observation. Fourier-transformed EXAFS (FT-EXAFS) analyses further reveal a pronounced peak at ~2.48 Å in PSFZM, located between the Zn–Zn scattering of Zn foil (2.24 Å) and the Zn–O–Zn scattering of ZnO (2.98 Å) (**Supplementary Fig. 13**). This intermediate position indicates that the Zn-O-Zn motif in PSFZM does not arise from a continuous Zn-O-Zn-O aggregated network, but rather reflects a second-nearest-neighbor geometric correlation between Zn and the surrounding oxygen polyhedra. More importantly, PSFZM features a distinct Zn–O–M coordination shell with a bond length of 3.88 ± 0.01 Å, and the spectrum can be reproduced without invoking any direct Zn-Zn scattering contribution. This scattering path is absent in both PSFZ and the Zn reference samples (**Fig. 2k**), pointing to a stronger Zn-centred local ordering tendency in PSFZM[33]. Wavelet-transformed (WT) EXAFS contour plots (**Fig. 2l**) resolve the scattering contributions in the (k, R) plane and further substantiate this conclusion: PSFZM displays a low-k intensity maximum corresponding to Zn–O backscattering, together with a discrete high-k lobe at k ≈ 9 $Å^{-1}$ that is attributed to the heavier second-shell Zn–O–M path. Combined with the X-ray photoelectron spectroscopy (XPS) results, this direct structural evidence confirms the successful construction of atomic-scale Zn–O–M coupling within the perovskite matrix (**Supplementary Figs. 14-18**). Taken together, these structural analyses verify that periodically modulated active domains have been successfully established in

PSFZM.

Combining the Mo K-edge and Fe K-edge XANES spectra (**Figs. 4g-4j and Supplementary Figs 36-39**) with the WT-EXAFS spectra (**Figs. 4k,4l**) revealed that the chemical states and bond lengths of Mo-O and Fe-O in PSFZM are similar to those observed in PSFZ and PSFM. The bond lengths were determined to be 1.77 ± 0.01 Å for Mo-O and 1.95 ± 0.02 Å for Fe-O, indicating that the chemical states of Mo and Fe are unaffected by the special distorted Ruddlesden-Popper structure (**Supplementary Tables 4, 5, Supplementary Figs 40, 41**). The adsorption characteristics and intermediate evolution processes in PSFZM align with those in PSFZ and PSFM. This further supports the conclusion that Zn is the primary adsorption site in PSFZM, dominating the continuous activation of O_2 at this site.

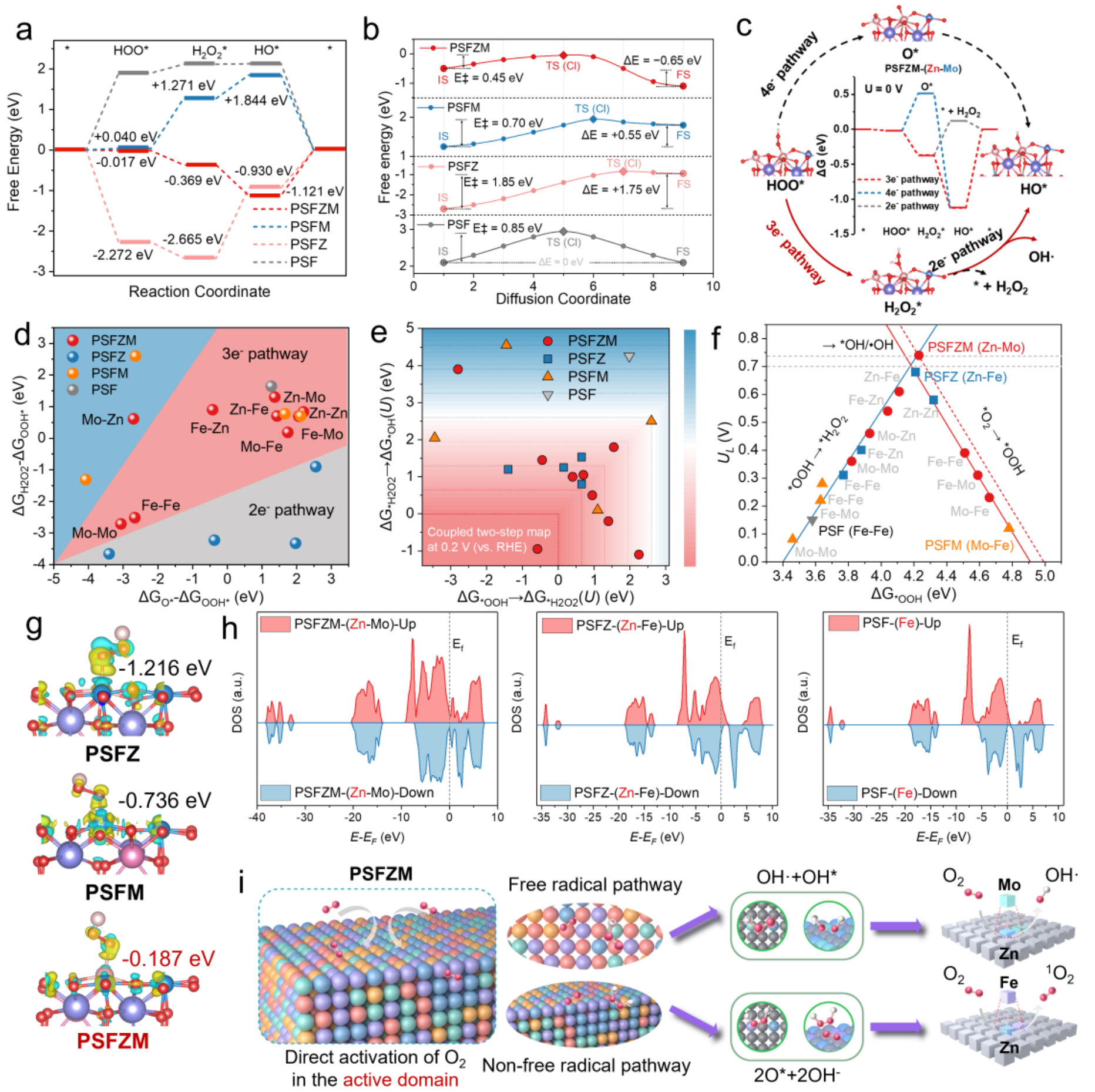

Fig. 5 DFT calculations involving each reaction site. a Gibbs free energy of the whole reaction pathway for the generation of •OH via three-electron ORR for all samples. **b** CI-NEB energy profiles for the rate-determining elementary step on the four candidate catalysts.

c Structural models and corresponding free energy comparisons of the 3e⁻ ORR and 4e⁻ ORR pathways on the PSFZM catalyst. **d** Energy differences between reaction intermediates at equilibrium potential. The blue, red, and gray regions indicate active sites for the thermodynamically driven 4e⁻, 3e⁻, and 2e⁻ ORR pathways, respectively. **e** Two-step coupled selectivity map at 0.2 V (vs. RHE). **f** Scaling-relation analysis of intermediate adsorption free energies on dual-atom sites. **g** Side view of the charge density difference for H_2O_2 adsorbed on PSFZ/PSFM/PSFZM, the cyan region represents charge depletion, and the yellow region indicates charge accumulation. **h** Calculated density of states (DOS) analysis for PSF/PSFZ/PSFZM after H_2O_2 adsorption. **i** Schematic diagram of the reaction pathway of O_2 generating ROS through free radical and non-free radical at the active domain of PSFZM. Colors represent: Pr (red), Sr (purple), Fe (yellow), Zn (green), Mo (blue).

Density functional theory (DFT) calculations show that H_2O_2 desorption from the Zn site is an uphill process, that is, a non-spontaneous step with ΔG = +0.52 eV. This feature suppresses H_2O_2 release and thereby enhances the selectivity toward the 3e⁻ ORR pathway. Among all examined structural motifs, the Zn-O-Mo unit in the material constructs a confined microenvironment that optimizes the orientation of reaction intermediates and their desorption kinetics (**Supplementary Figs. 50-65**).

We further performed climbing-image nudged elastic band (CI-NEB) calculations to locate the initial state (IS), transition state (TS), and final state (FS) of each model, thereby evaluating the kinetic accessibility of the key O-O bond activation step. The transition-state profiles show that PSFZM exhibits the lowest energy barrier for converting surface-adsorbed H_2O_2 into •OH-related intermediates. In contrast, PSFZ, PSFM, and PSF encounter higher kinetic barriers or energetically less favorable final states (**Fig. 5b**). This result indicates that the enhanced •OH yield on PSFZM does not arise solely from the thermodynamic stabilization of peroxide species, but also from the simultaneous reduction of the kinetic barrier for intradomain peroxide activation. Meanwhile, the cleavage of $*H_2O_2$, identified as the rate-determining step (RDS), is a downhill process on PSFZM, whereas it is uphill on PSFM (0.75 eV), PSFZ (1.73 eV), and PSF (0.01 eV). Therefore, the unique Zn-O-Mo configuration lowers the critical energy barrier, accelerates electron transfer, and rationalizes the experimentally observed superior •OH generation rate (**Fig. 5c**).

Conventional descriptors, such as ΔG_{*OOH}, often fail to accurately capture the energetics

of downstream product formation. Therefore, we introduced ΔG_{*H2O2}–ΔG_{*OOH} and ΔG_{*O}–ΔG_{*OOH} as more predictive descriptors for reaction selectivity (**Fig. 5d**). Mechanistic mapping shows that the PSFZM catalytic site is located in a specific region that favors the formation of H_2O_2 and •OH while effectively suppressing excessive reduction toward the *O intermediate. A coupled two-step descriptor map at 0.2 V versus RHE further shows that the Zn-O-Mo sites of PSFZM occupy the optimal region where peroxide formation and peroxide activation are balanced (**Fig. 5e**). In contrast, PSFZ stabilizes peroxide intermediates but lacks sufficient activation capability, leading to H_2O_2 accumulation, while PSFM promotes stronger O–O activation but does not efficiently retain the peroxide intermediate. In **Fig. 5f**, the two volcano branches describe the thermodynamic limitations associated with $*H_2O_2$ and *OOH formation, respectively. The volcano apex approaches the equilibrium potential of the O_2/H_2O_2 couple (U° = 0.70 V vs. RHE), defining the minimum theoretical overpotential imposed by the scaling relationship. The Zn–O–Mo motif in PSFZM lies near this optimum and gives the highest limiting potential among all examined motifs ($\Delta G_{*OOH} \approx 4.28$ eV, $U_L \approx$ 0.749 V).This position indicates that the Zn–O–Mo active domain effectively balances *OOH stabilization with $*H_2O_2$ activation, thereby mitigating the conventional scaling constraint and promoting the confined conversion of surface-retained $*H_2O_2$ toward •OH.

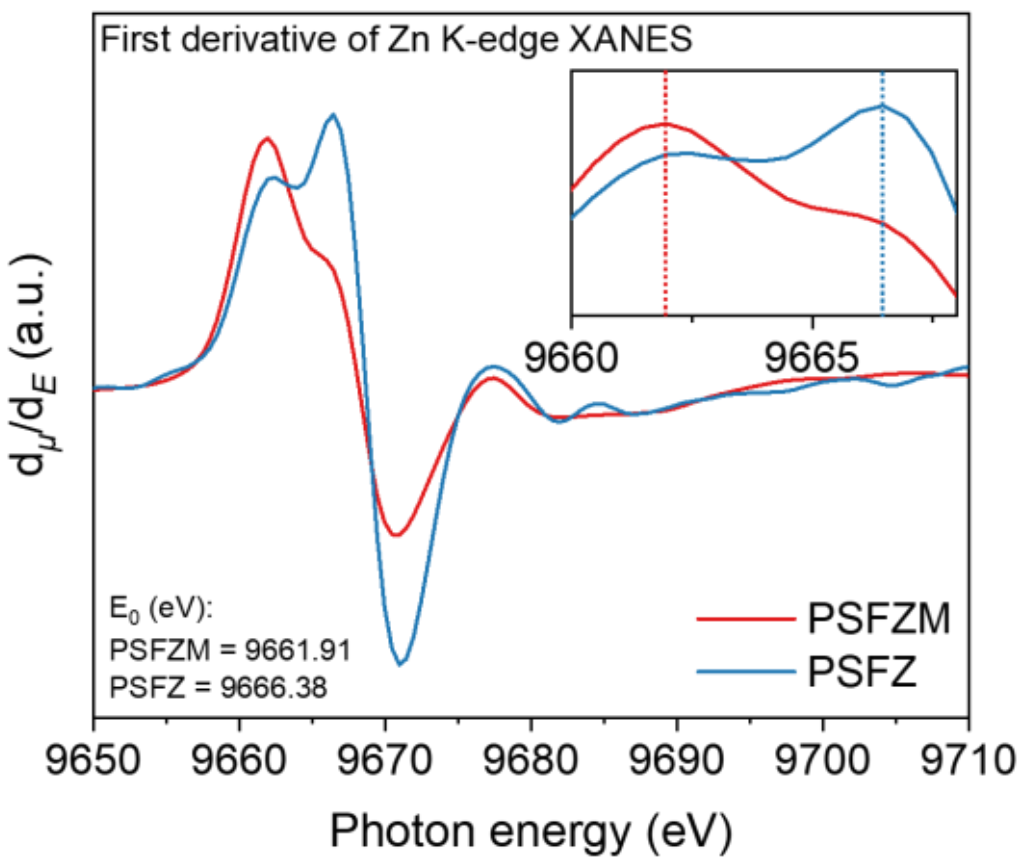

Supplementary Fig. 12 First-derivative Zn K-edge XANES spectra of PSFZM and PSFZ.

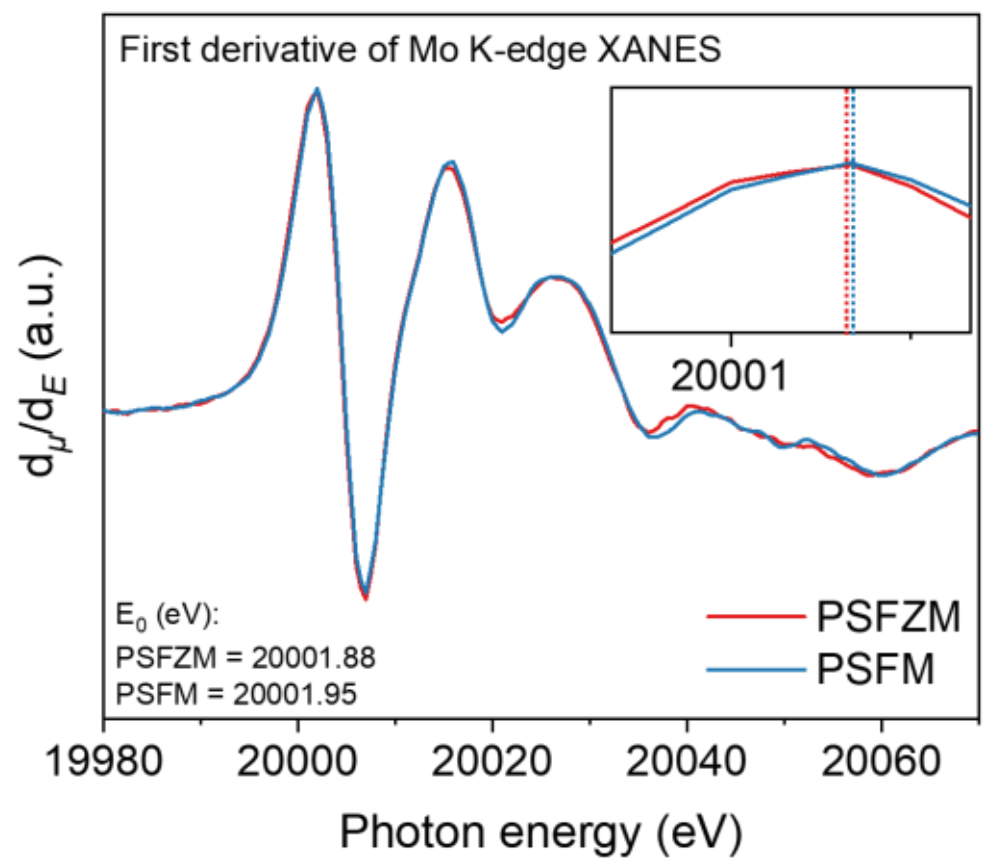

Supplementary Fig. 36 First-derivative Mo K-edge XANES spectra of PSFZM and PSFM.

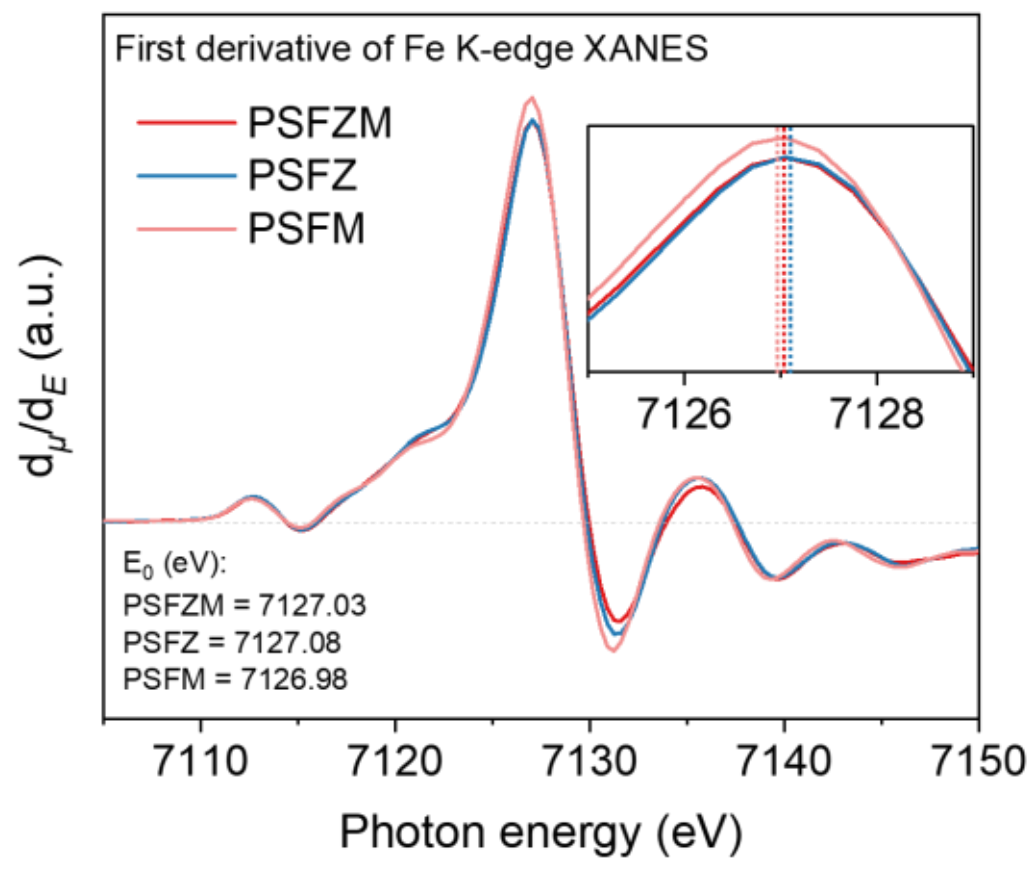

Supplementary Fig. 37 First-derivative Fe K-edge XANES spectra of PSFZM, PSFZ and PSFM.

2. The authors throughout the manuscript assign Zn as the adsorption site and Mo as the polarizing agent that facilitates O-O bond cleavage; however, no direct evidence is provided to support the proposed role of Mo. Moreover, Fe is also shown by the aberration-corrected TEM results to be arranged in an ordered single-atomic configuration, yet its role is not discussed. In contrast, Mo does not appear to form an ordered single-atomic structure. Therefore, the "single-atom engineered" Zn-O-Mo active sites mentioned in the manuscript are ambiguous and require supporting evidence and clarification.

Response: We appreciate your valuable comments and constructive suggestions. We have carefully checked your concerns. We thank the reviewer for this important comment. To demonstrate the periodic formation of the single-atom-engineered active domains, we

performed statistical analysis of aberration-corrected TEM images collected from multiple independent regions. More than 500 B-site atomic columns were analyzed to quantify the spatial distribution of abnormal-contrast B-site columns assigned to Zn/Mo-containing local motifs. The statistical analysis shows that this distribution deviates significantly from a random occupation model, as confirmed by the χ^2 test. In addition, the radial distribution function of the abnormal-contrast B-site columns approaches the random limit at long distances, indicating that the Zn/Mo-related enrichment is local rather than associated with long-range phase separation.

Furthermore, we performed Rietveld refinement of the powder X-ray diffraction patterns of PSFZ, PSFM and PSFZM. Compared with the singly doped PSFZ and PSFM samples, PSFZM exhibits lattice expansion and more pronounced B–O bond distortion, consistent with the formation of a mixed-metal B-site environment rather than separated oxide phases.

To support the proposed role of Mo, we have added and revised several lines of evidence. First, EXAFS fitting and wavelet-transform analysis now provide more quantitative support for a mixed Zn–O–Mo local coordination environment. The fitting parameters, including coordination number, bond length, Debye–Waller factor, ΔE_0 and R factor, are summarized in a new Supplementary Table. Second, the comparison among PSFZ, PSFM and PSFZM demonstrates the functional role of Mo: PSFZ favours H_2O_2 accumulation, PSFM tends toward deeper oxygen reduction, whereas PSFZM suppresses H_2O_2 accumulation and enhances •OH generation. Third, DFT and CI-NEB calculations show that introducing Mo adjacent to Zn changes the adsorption configuration of $*H_2O_2$, strengthens O–O polarization and lowers the kinetic barrier for peroxide activation/O–O bond cleavage compared with Zn-only, Mo-only and Fe-centred motifs.

We also emphasize that the proposed active domain does not require long-range ordered Mo single atoms. Instead, Mo functions as a locally adjacent B-site component within the distorted perovskite lattice. Thus, the key structural feature is not an ordered Mo single-atom array, but the formation of localized Zn–O–Mo coordination motifs that enable cooperative adsorption and activation of peroxide intermediates. We have revised the manuscript accordingly to avoid overstatement and to clearly distinguish ordered Fe-containing lattice columns from locally dispersed Zn/Mo active domains.

Manuscript Revision:

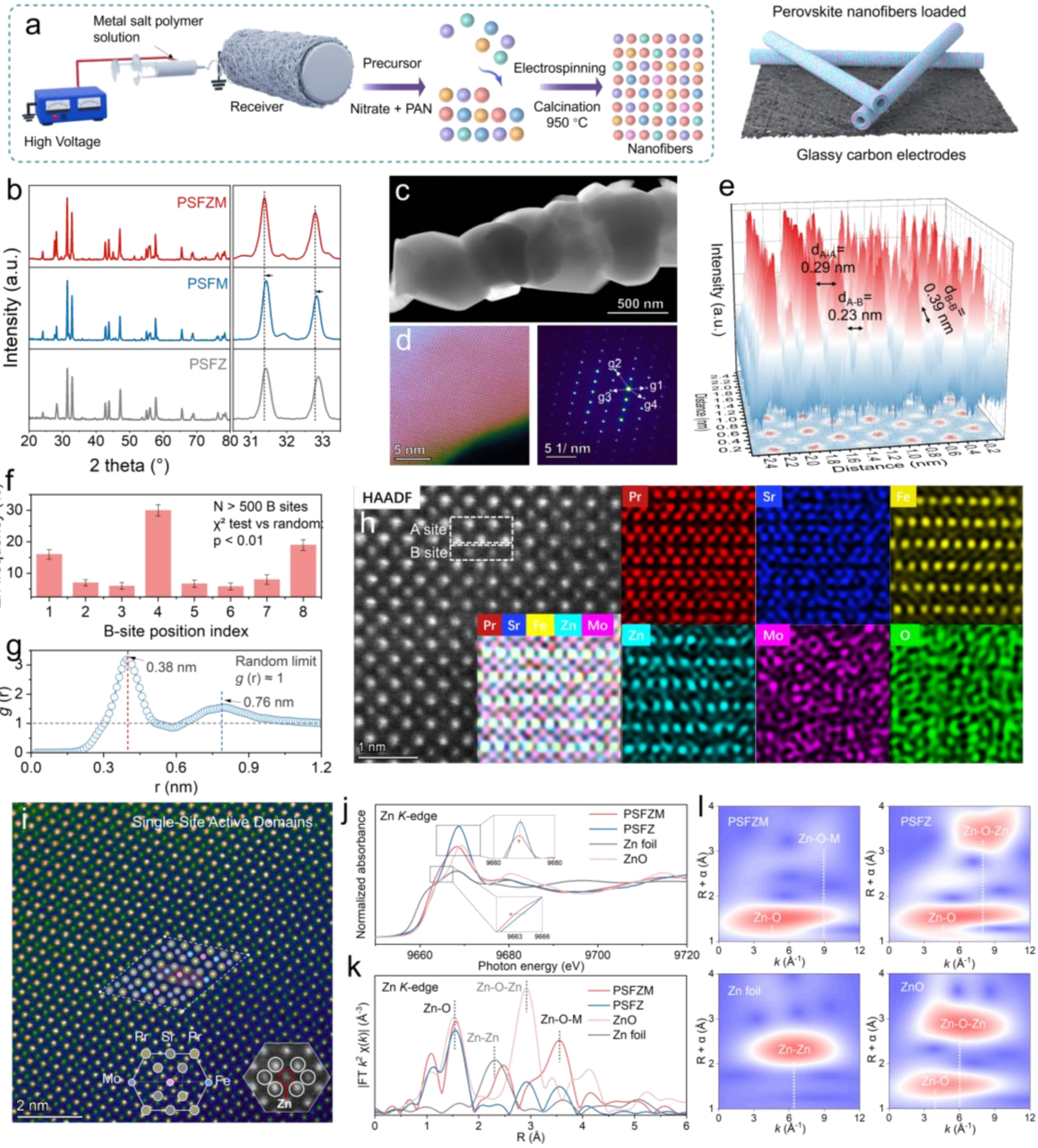

Fig. 2 Atomic structure characterization of the active domains for PSFZM. a Schematic diagram showing the preparation of PSFZM electrodes by the electrospinning method. Colors represent: Pr (red), Sr (purple), Fe (yellow), Zn (green), Mo (blue). **b** XRD patterns of PSFZM, PSFZ and PSFM. **c** SEM image of PSFZM. **d** HAADF-STEM image. The inset shows the corresponding selected area electron diffraction (SAED) pattern of PSFZM. **e** Atomic spacing diagram based on HAADF-STEM. **f** Periodicity analysis of Zn occupation on the B-site sublattice. **g** Radial distribution function g(r) of Zn-rich B-site columns. **h**

HAADF-STEM image and the corresponding elemental maps for PSFZM. **i** The elemental intensity maps obtained from HAADF-STEM conversion show that elements with larger ionic radii are A-site elements, while those with smaller ionic radii are B-site elements. **j** Zn K-edge XANES spectra of PSFZM, PSFZ, Zn foil, ZnO. **k** Zn K-edge k^3-weighted FT-EXAFS spectra and **l** WT-EXAFS spectra of PSFZM, PSFZ, Zn foil, ZnO.

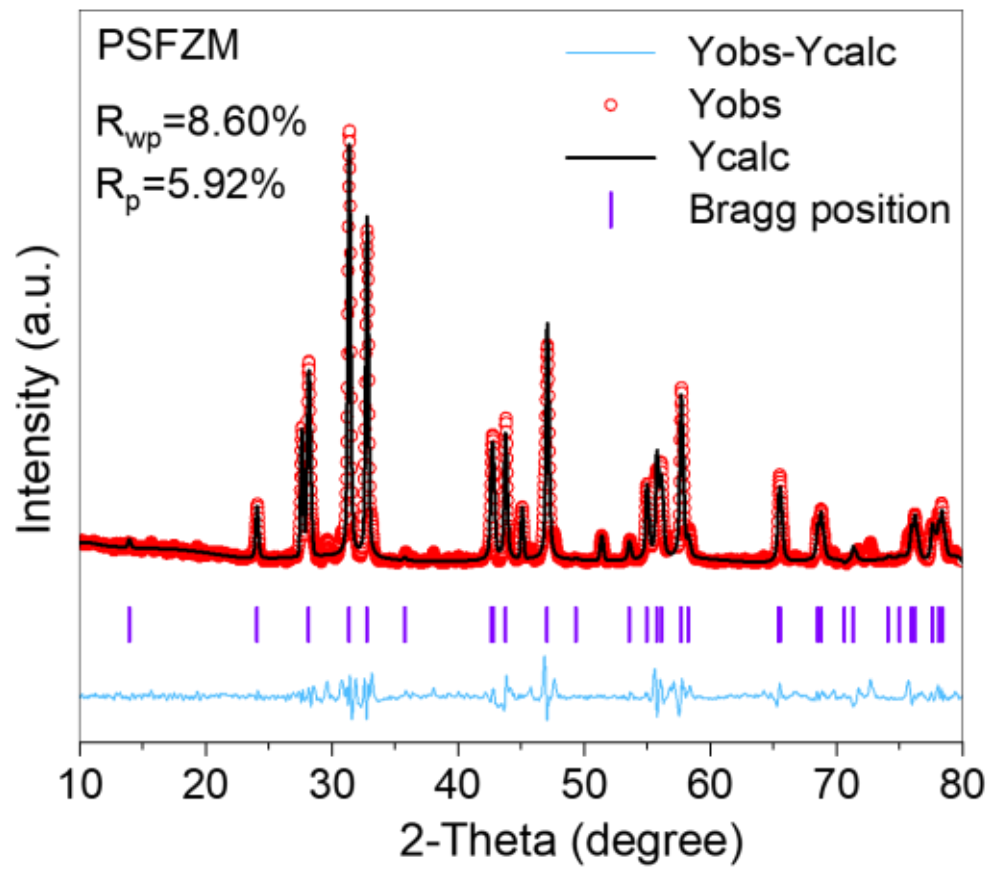

Supplementary Fig. 2. XRD Rietveld refinement of PSFZM.

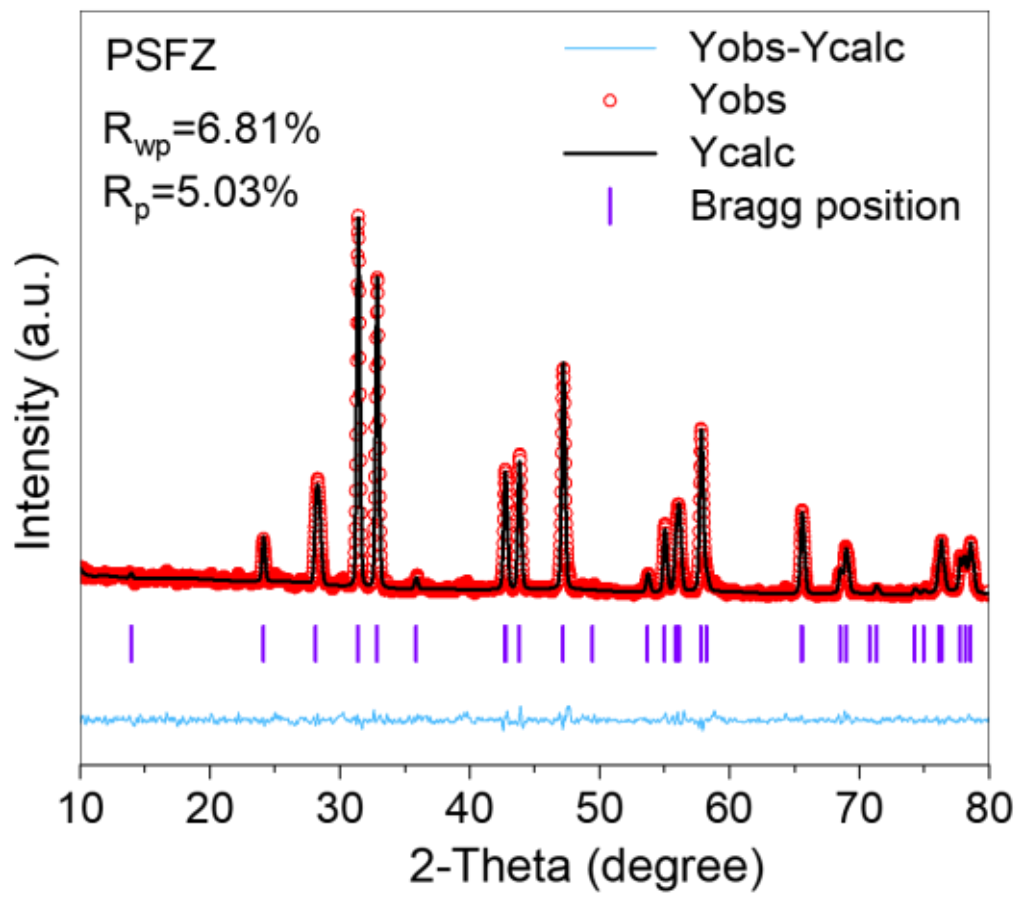

Supplementary Fig. 3. XRD Rietveld refinement of PSFZ.

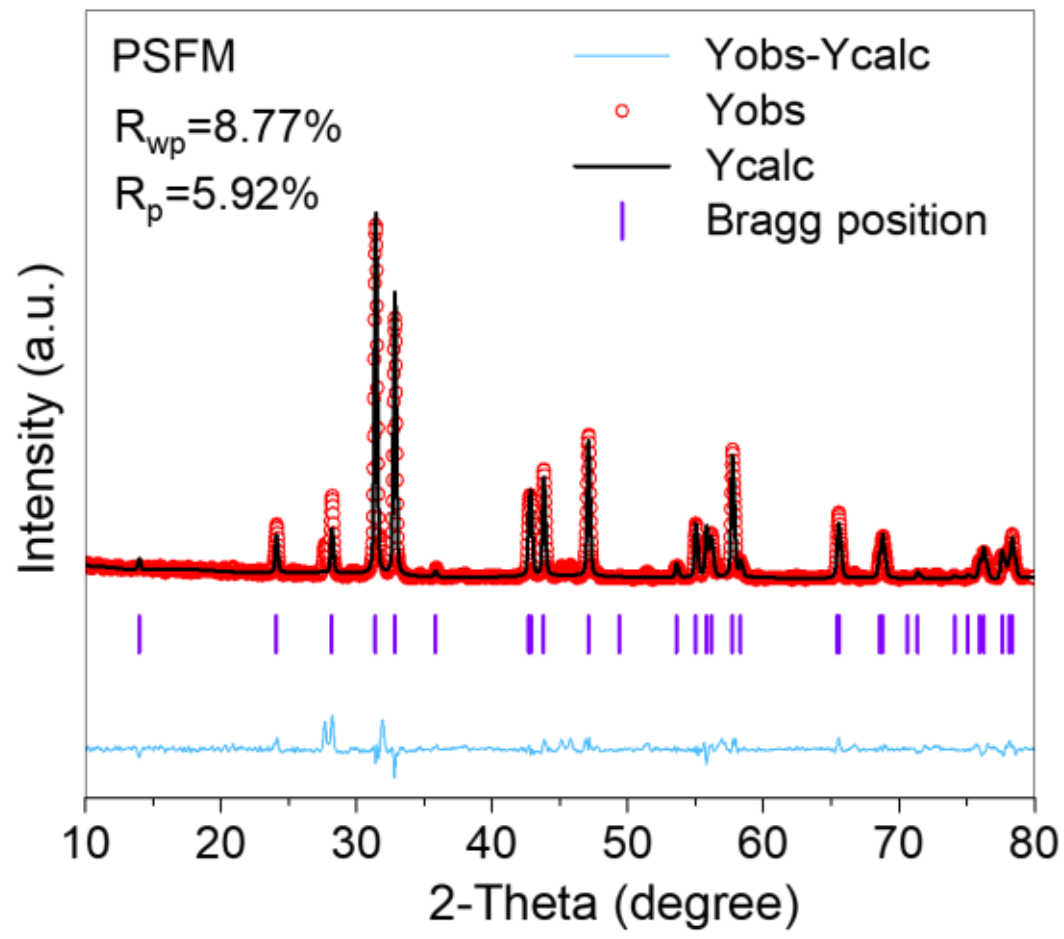

Supplementary Fig. 4. XRD Rietveld refinement of PSFM.

Supplementary Table 1. Rietveld-refined crystallographic parameters of PSFZ, PSFM and PSFZM.

**Parameter**	**PSFZ**	**PSFM**	**PSFZM**
**Nominal composition**	$PrSrFe_{0.75}Zn_{0.25}O_{4-\delta}$	$PrSrFe_{0.75}Mo_{0.25}O_{4-\delta}$	$PrSrFe_{0.5}Zn_{0.25}Mo_{0.25}O_{4-\delta}$
**Space group**	I4/mmm	I4/mmm	I4/mmm
**Crystal system**	Tetragonal	Tetragonal	Tetragonal
**a = b / Å**	3.848739	3.855880	3.858459
**c / Å**	12.670967	12.651975	12.673535
**V / Å^3**	187.692	188.107	188.680
**c/a**	3.292	3.281	3.285
**B-site occupation**	$Fe_{0.75}Zn_{0.25}$	$Fe_{0.75}Mo_{0.25}$	$Fe_{0.5}Zn_{0.25}Mo_{0.25}$
**O1 occupancy**	0.772	0.780	0.560
**O2 occupancy**	0.986	0.975	0.976
**B–O_{eq} / Å**	1.924	1.928	1.929
**B–O_{ap} / Å**	1.995	1.990	2.058
**B–O_{ap}/B–O_{eq}**	1.037	1.032	1.067

**Rwp / %**	6.81	8.77	8.60
**Rp / %**	5.03	5.92	5.92
**RF / %**	4.03	5.72	4.92
**RF^2 / %**	6.70	13.18	8.05

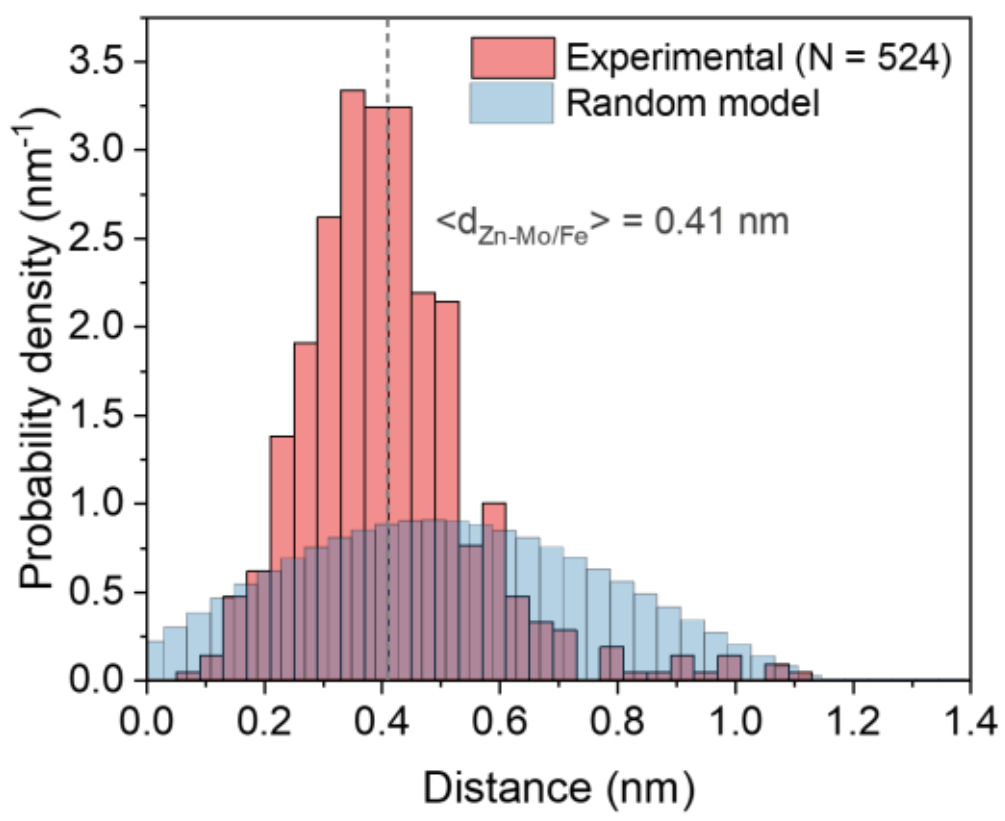

Supplementary Fig. 8 Statistical distribution of Zn–Mo/Fe neighbouring distances in PSFZM.

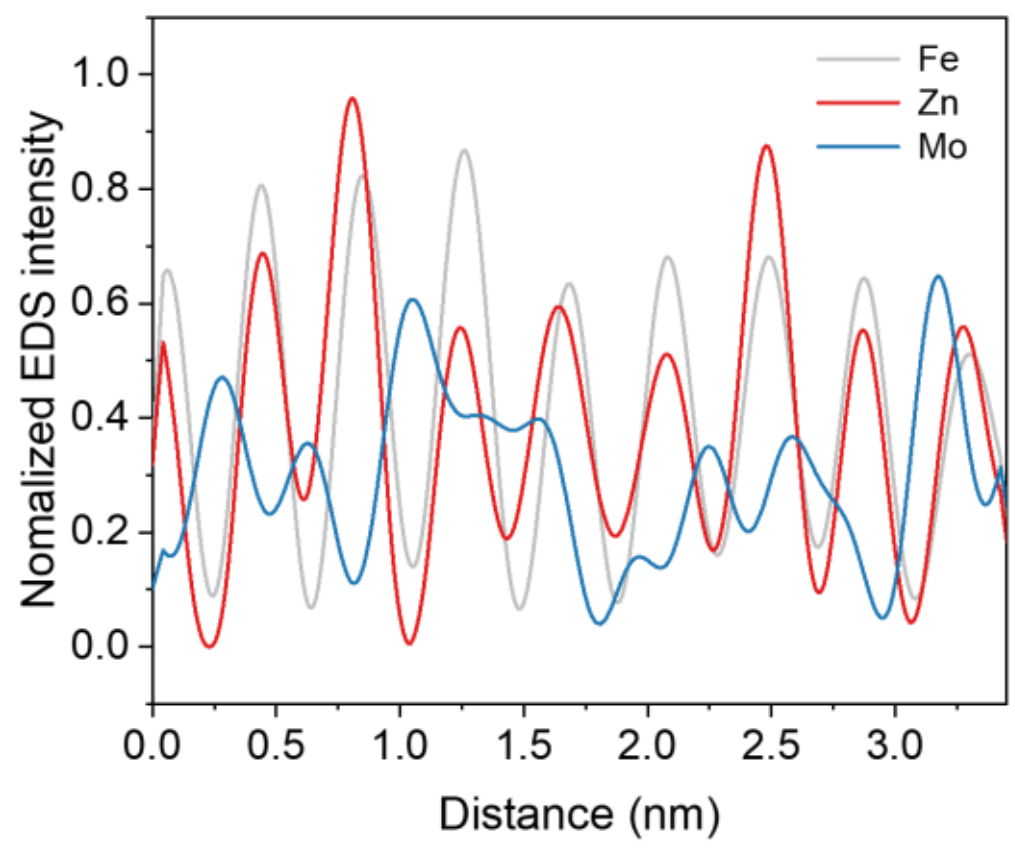

Supplementary Fig. 9 STEM-EDS line-scan analysis of Fe, Zn and Mo distributions in PSFZM.

Rietveld-refined X-ray Diffraction (XRD) patterns confirm that PSFM, PSFZ, and PSFZM retain the tetragonal Ruddlesden-Popper structure with the I4/mmm space group

(**Supplementary Figs. 2-4, Supplementary Table 1**). PSFZM exhibits the largest unit-cell volume and the most pronounced elongation of axial B–O bonds, accompanied by left-shifted diffraction peaks at ~32° and ~34° (**Fig. 2b**). These crystallographic features demonstrate that Zn/Mo co-doping induces lattice expansion and BO_6 octahedral distortion, supporting the formation of mixed-metal B–O active domains distinct from the single-metal local environments. Field-emission scanning electron microscopy (FE-SEM) and high-angle annular dark-field scanning transmission electron microscopy (HAADF-STEM) images reveal that PSFZM forms a highly crystalline porous nanofiber network structure (**Fig. 2c and Supplementary Figs. 5-7c**). Aberration-corrected high-resolution transmission electron microscopy (AC-HRTEM) images (**Fig. 2d**) together with the atomic spacing map (**Fig. 2e**) further disclose an orthogonally distorted tetragonal lattice within PSFZM. Quantitative statistical analysis of more than 500 B-site atomic columns indicates that the distribution of Zn sites in PSFZM is non-random (**Supplementary Figs. 8 and 9**), showing a significant deviation from the random occupancy model according to the χ^2 test ($p < 0.01$; **Fig. 2f**). The radial distribution function decays toward the random-limit value of $g(r) \approx 1$ (**Fig. 2g**), indicating the presence of a localized, zinc-enriched ordered structure. Atomic-scale elemental mapping and STEM-energy dispersive X-ray spectroscopy (EDS) line-scan analysis further confirms this conclusion, showing that Pr and Sr atoms are uniformly distributed at the A sites, whereas Zn atoms are periodically enriched at the B sites near the center of the distorted tetragonal unit cells (**Figs. 2h, 2i and Supplementary Figs. 7d, 10-11**). Collectively, these results provide compelling evidence for the formation of Zn-centered active-domain units.

3. In situ FTIR spectroscopy indicates the presence of the *OOH and $*H_2O_2$ intermediates; however, it does not directly capture O-O bond cleavage or the formation of •OH. Therefore, the claim of "continuous unit-site conversion" is not substantiated. Moreover, during the in-situ reaction, PSFZM and PSFZ exhibit nearly identical adsorption evolution of the intermediates. How, then, can the latter be inferred to continuously accumulate H2O2? As such, the results remain inconclusive.

Response: We appreciate your valuable comments and constructive suggestions. We agree that in situ Fourier Transform Infrared (FTIR) spectroscopy cannot directly capture the cleavage of O-O bonds or the generation of •OH radicals; rather, it can only infer subsequent

processes by identifying changes in the spectroscopic signals of surface oxygen-containing intermediates, specifically *OOH and *H_2O_2. Consequently, we have thoroughly revised the manuscript and incorporated new evidence derived from Differential Electrochemical Mass Spectrometry (DEMS) and FTIR analysis of a physical mixture of PSFZ and PSFM.

To address this concern, we added complementary isotope-resolved and transient spectroscopic evidence to directly track the fate of peroxide intermediates and •OH formation. First, isotope-resolved DEMS was performed under $^{18}O_2$-saturated conditions. PSFZ shows pronounced m/z = 37/38 signals, which are assigned to ^{18}O-labelled peroxide/hydroperoxide species, consistent with the formation and release of H_2O_2 through a dominant $2e^-$ ORR pathway. In contrast, PSFM mainly shows a strong m/z = 20 signal, corresponding to $H_2{}^{18}O$ formation via deeper $4e^-$ reduction. Distinct from both reference catalysts, PSFZM exhibits a pronounced m/z = 19 signal associated with ^{18}OH-related species, whereas the peroxide-related m/z = 37/38 signals remain weak and transient. These results indicate that O_2-derived oxygen is incorporated into hydroxyl radical species on PSFZM without substantial accumulation of peroxide products.

Second, time-resolved DEMS measurements during potential-step electrolysis were added to distinguish surface-confined conversion from a desorption–diffusion–re-adsorption sequence. If •OH formation occurred mainly through desorbed H_2O_2 followed by secondary activation at another interfacial site, the peroxide-related signal would be expected to appear before the hydroxyl-related signal and show a different relaxation behaviour. However, on PSFZM, the m/z = 19/20 and m/z = 37/38 channels show synchronized and reversible responses during each potential step, without an observable peroxide-leading delay. This transient behaviour supports rapid conversion of surface *H_2O_2 before detectable desorption and diffusion.

Third, ESR measurements provide direct evidence for •OH formation. The newly added ESR spectra show clear DMPO–•OH signals on PSFZM under O_2-saturated conditions, including at low potentials where RRDE indicates enhanced $3e^-$ ORR behaviour. In contrast, the signals are negligible under control conditions without O_2. Therefore, while FTIR identifies the *OOH/*H_2O_2 intermediates, ESR and isotope-resolved DEMS provide direct evidence for •OH generation.

To further support this distinction, we introduced a PSFZ + PSFM physical mixture as

a control representing a spatially separated tandem system, in which one component favours H_2O_2 formation and the other promotes subsequent activation. Operando FTIR shows that the physical mixture exhibits much stronger $*H_2O_2$ accumulation than PSFZM. This comparison demonstrates that simply combining H_2O_2-generating and H_2O_2-activating components does not reproduce the peroxide-confined behaviour of PSFZM, supporting the importance of the local Zn-O-Mo active domain.

The revised interpretation is that FTIR confirms the sequential formation of *OOH and $*H_2O_2$ intermediates, while isotope-resolved DEMS, time-resolved DEMS and ESR provide direct evidence for peroxide conversion and •OH formation. Taken together, these results support a surface-confined consecutive $3e^-$ ORR pathway on PSFZM, distinct from the H_2O_2-accumulating $2e^-$ pathway observed on PSFZ.

Manuscript Revision:

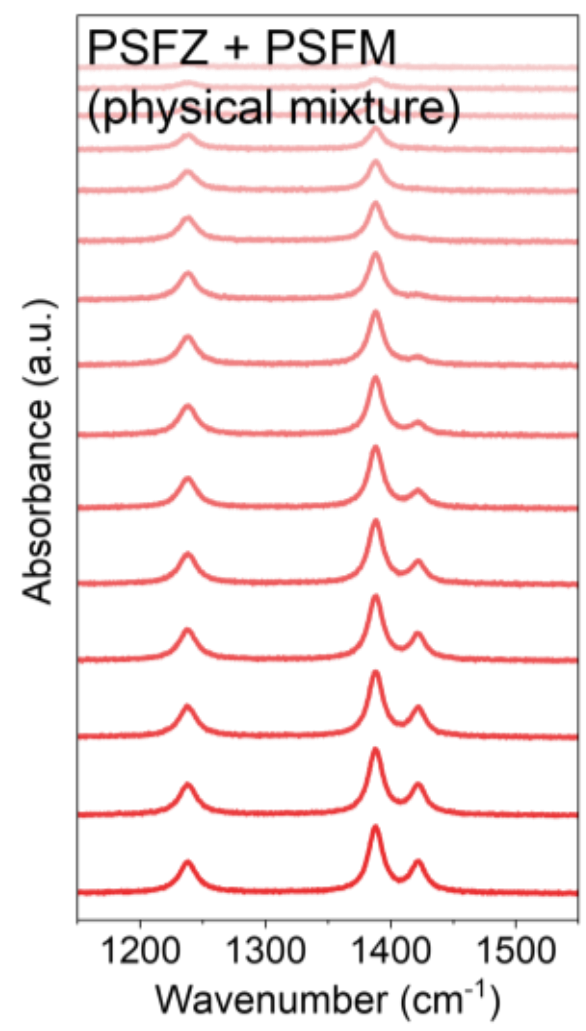

Supplementary Fig. 28 The ORR electrochemical in-situ FTIR spectra of PSFZ + PSFM physical mixture catalyst collected in an incremental voltage configuration.

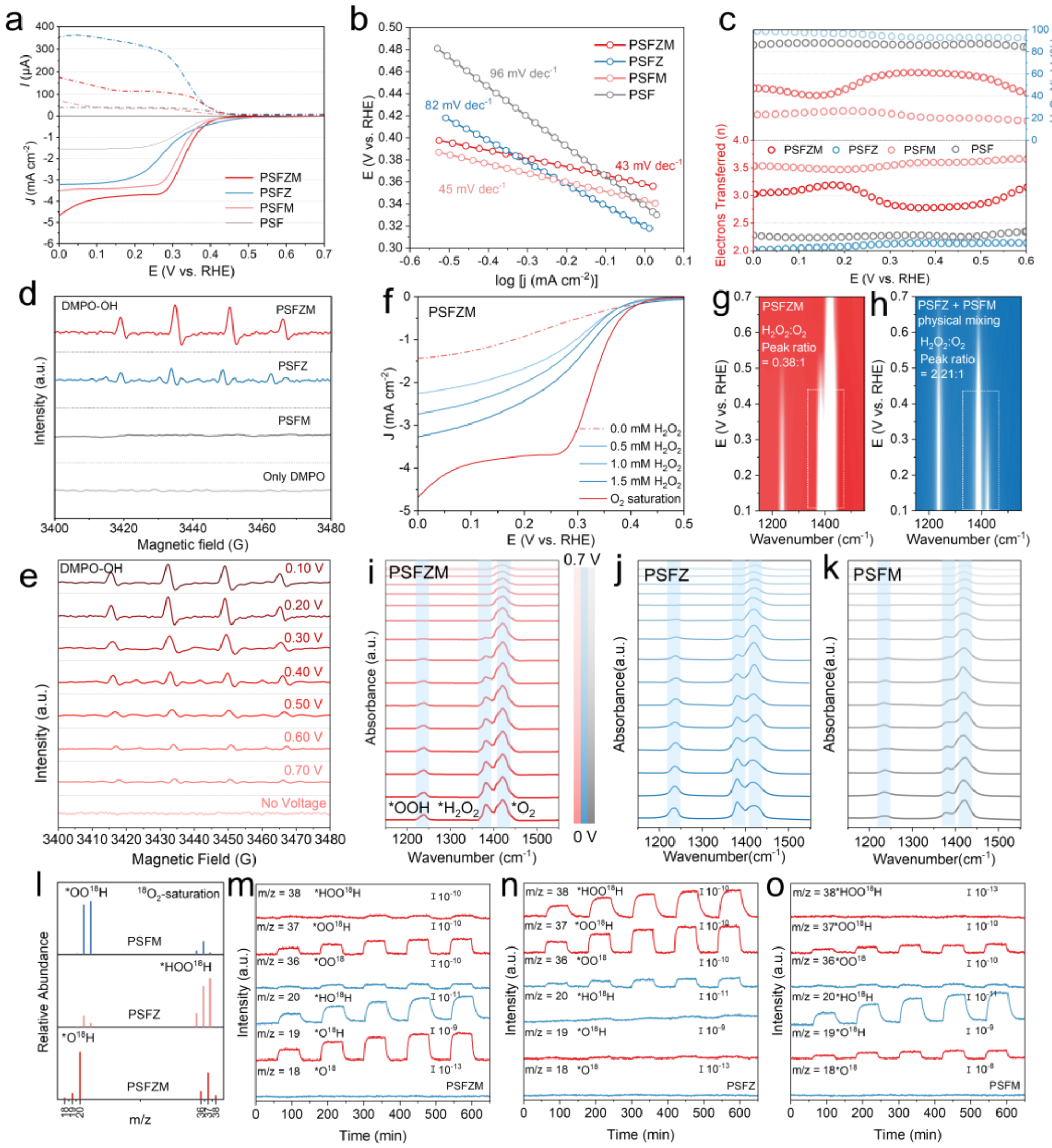

Fig. 3 Qualitative analysis of ORR selectivity and O_2 conversion to •OH. **a** RRDE polarization profiles for oxygen reduction reaction. Note: The solid lines are the disk current density corresponding to oxygen reduction and the dotted lines are the ring current density corresponding to H_2O_2 oxidation. **b** The corresponding Tafel plots. **c** H_2O_2 selectivity and number of transferred electrons (n) calculated from RRDE polarization curves. **d** EPR spectra of DMPO-•OH adducts for samples from different catalyst-catalyzed electro-Fenton reactions. **e** DMPO-•OH adduct EPR spectra of ORR electrochemistry over PSFZM catalyst collected in an incremental voltage configuration. **f** LSV with different concentrations of H_2O_2 raw materials in the O_2-free configuration and the O_2-filled configuration. Presented are the potential-dependent in situ FTIR contour plots of **g** PSFZM and the **h** PSFZ+PSFM

physical mixture, acquired in an O_2-saturated electrolyte. The ORR electrochemical in-situ FTIR spectra of **i** PSFZM, **j** PSFZ, and **k** PSFM catalyst collected in an incremental voltage configuration. **l** Representative mass spectra of PSFZM, PSFZ, and PSFM, recorded under conditions saturated with $^{18}O_2$, reveal distinct ^{18}O-labeled oxygen reduction products. Time-resolved DEMS traces of selected isotope-labeled fragments, including peroxide/hydroperoxide-related channels and hydroxyl/water-related channels, during intermittent electrolysis over **m** PSFZM, **n** PSFZ, and **o** PSFM.

To further substantiate this mechanism, in-situ Fourier-transform infrared spectroscopy (FT-IR) was used to clarify the evolution of reaction intermediates (**Figs. 3g–3k**). With increasing cathodic bias, the *OOH and *H_2O_2 signals on PSFZ were markedly enhanced, indicating continuous H_2O_2 accumulation without subsequent activation (**Fig. 3j**). By contrast, PSFM exhibited only a weak *OOH signal and almost no detectable H_2O_2 feature, consistent with a four-electron ($4e^-$) ORR pathway (**Fig. 3k**). In sharp contrast, PSFZM displayed an O-O stretching band of adsorbed O_2 at ~1424 cm^{-1}; as the potential shifted negatively, characteristic *OOH-related bands subsequently emerged at ~1241 and ~1389 cm^{-1} (**Fig. 3i**). Notably, the *OOH and *H_2O_2 species reached a dynamic formation–consumption equilibrium near 0.2 V. To more clearly distinguish continuous ORR evolution from spatially separated tandem evolution, PSFZM was compared with a physical mixture of PSFZ and PSFM. For PSFZM, the relative intensity ratio of the *H_2O_2-related band to the O_2/*OOH-related band was only 0.38:1, indicating that peroxide did not accumulate as the dominant surface species (**Fig. 3g**). The nearly six-fold enhancement of the peroxide-related signal in the PSFZ+PSFM physical mixture indicates substantial H_2O_2 accumulation, consistent with a conventional decoupled $2e^- + 1e^-$ tandem process (**Fig. 3h, Supplementary Fig. 28**). These results strongly support a confined active-domain-mediated continuous conversion pathway of $O_2 \rightarrow$ *OOH $\rightarrow$ *$H_2O_2 \rightarrow$ •OH, in which the B-site Zn centers serve as the key sites for direct O_2 activation during the $3e^-$ ORR process.

To resolve the operative ORR pathway on PSFZM, we performed isotope-resolved DEMS in $^{18}O_2$-saturated electrolyte. The relative mass spectral abundance distribution (**Fig. 3l**) shows that PSFM exhibits strong responses only at m/z = 18-20, corresponding to a typical $4e^-$ ORR pathway. In contrast, PSFZ displays synchronized signals at m/z = 37 and 38, while remaining silent at m/z = 19 and 20, indicating the direct desorption of *OOH as

H_2O_2. Time-resolved DEMS measurements (**Figs. 3m-o**) further reveal that PSFZM exhibits synchronized and reversible responses across all four channels (m/z = 37, 38, 19, and 20) during each potential step. Compared with PSFZ, the H_2O_2 signal (m/z = 38) is markedly suppressed, whereas the *OH/ H_2O signals (m/z = 19/20) are simultaneously enhanced. In a conventional tandem mechanism involving H_2O_2 desorption–diffusion–re-adsorption, the m/z = 38 signal must precede the m/z = 19/20 signals and exhibit distinct relaxation times. Therefore, the strictly synchronized responses observed for PSFZM can only originate from the continuous reduction of surface-confined *H_2O_2 intermediates at the same active site.

4. The DFT results support this proposed mechanism; however, the treatment lacks rigor and depth. Only the Gibbs free energy is reported, while key information-such as transition states, energy barriers, reaction kinetics, and potential-dependent free-energy profiles-is missing. A complete transition-state search, kinetic barrier analysis, and a consistent mechanistic interpretation for •OH and $^1O_2$ are therefore required. Moreover, the manuscript claims that PSFZM follows a 3e- pathway rather than the conventional $2e^- + 1e^-$ route. While the authors attempt to exclude the 2e- pathway through characterization and mechanistic or DFT analyses, they do not rule out a single-electron pathway; this should be explicitly demonstrated. Otherwise, one can only conclude that the $2e^-$ pathway is unlikely, rather than excluding the conventional $2e^- + 1e^-$ mechanism.

Response: We appreciate your valuable comments and constructive suggestions. We have carefully revised the manuscript and substantially expanded the computational analysis by adding transition-state searches, kinetic barrier calculations, and additional mechanistic comparisons based on potential-dependent free-energy profiles.

First, we performed CI-NEB transition-state calculations for the key elementary steps involved in the proposed *H_2O_2-to-•OH pathway (Fig. 5b). The newly added CI-NEB results show that the Zn–O–Mo motif in PSFZM lowers the kinetic barrier for *H_2O_2 activation/O–O bond cleavage compared with the Zn-only, Mo-only and Fe-centred motifs. In contrast, the competing pathway toward complete $4e^−$ reduction remains less favourable on PSFZM. These results provide a kinetic basis for the proposed surface-confined consecutive $3e^−$ ORR pathway, rather than relying only on thermodynamic free-energy descriptors. Furthermore, we have incorporated potential-dependent free energy profiles to distinguish between thermodynamic driving forces and kinetic barriers. The revised free energy analysis now

enables the identification of potential-determining steps and trends in intermediate stabilization across various applied potentials. Third, we explicitly evaluated the possible $2e^- + 1e^-$ pathway. In this pathway, $*H_2O_2$ would first desorb from the surface, diffuse or re-adsorb, and then undergo secondary activation. Our calculations show that H_2O_2 desorption from the Zn–O–Mo domain is thermodynamically less favourable than its further surface activation, whereas the CI-NEB results show a reduced barrier for in situ O–O bond cleavage of adsorbed $*H_2O_2$. These results support surface-confined conversion of $*H_2O_2$ on PSFZM and are consistent with the isotope-resolved DEMS and operando FTIR results showing suppressed peroxide accumulation.

Manuscript Revision:

Density functional theory (DFT) calculations show that H_2O_2 desorption from the Zn site is an uphill process, that is, a non-spontaneous step with $\Delta G = +0.52$ eV. This feature suppresses H_2O_2 release and thereby enhances the selectivity toward the $3e^-$ ORR pathway. Among all examined structural motifs, the Zn-O-Mo unit in the material constructs a confined microenvironment that optimizes the orientation of reaction intermediates and their desorption kinetics (**Supplementary Figs. 50-65**).

We further performed climbing-image nudged elastic band (CI-NEB) calculations to locate the initial state (IS), transition state (TS), and final state (FS) of each model, thereby evaluating the kinetic accessibility of the key O-O bond activation step. The transition-state profiles show that PSFZM exhibits the lowest energy barrier for converting surface-adsorbed H_2O_2 into •OH-related intermediates. In contrast, PSFZ, PSFM, and PSF encounter higher kinetic barriers or energetically less favorable final states (**Fig. 5b**). This result indicates that the enhanced •OH yield on PSFZM does not arise solely from the thermodynamic stabilization of peroxide species, but also from the simultaneous reduction of the kinetic barrier for intradomain peroxide activation. Meanwhile, the cleavage of $*H_2O_2$, identified as the rate-determining step (RDS), is a downhill process on PSFZM, whereas it is uphill on PSFM (0.75 eV), PSFZ (1.73 eV), and PSF (0.01 eV). Therefore, the unique Zn-O-Mo configuration lowers the critical energy barrier, accelerates electron transfer, and rationalizes the experimentally observed superior •OH generation rate (**Fig. 5c**).

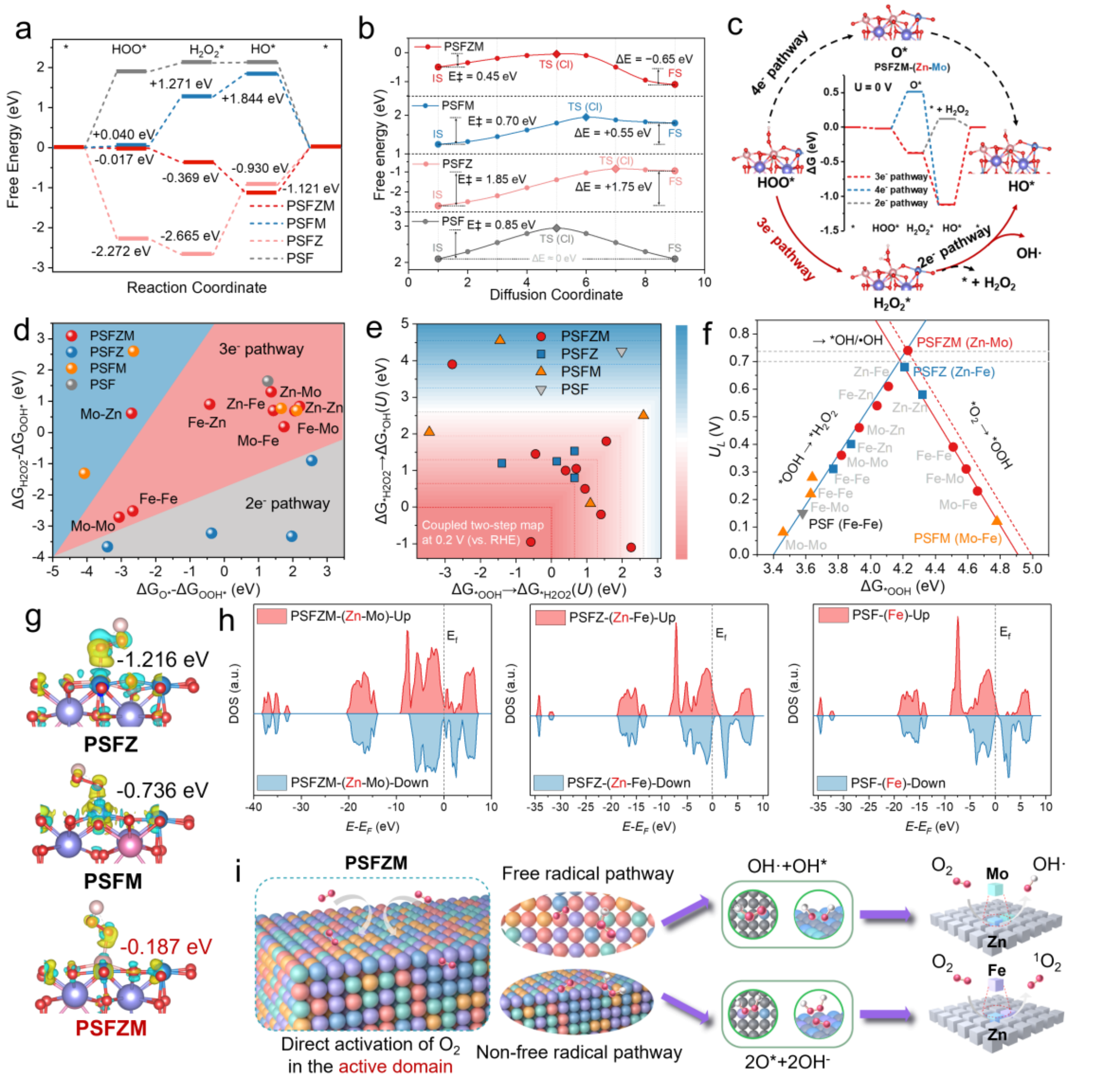

Fig. 5 DFT calculations involving each reaction site. **a** Gibbs free energy of the whole reaction pathway for the generation of •OH via three-electron ORR for all samples. **b** CI-NEB energy profiles for the rate-determining elementary step on the four candidate catalysts. **c** Structural models and corresponding free energy comparisons of the 3e⁻ ORR and 4e⁻ ORR pathways on the PSFZM catalyst. **d** Energy differences between reaction intermediates at equilibrium potential. The blue, red, and gray regions indicate active sites for the thermodynamically driven 4e⁻, 3e⁻, and 2e⁻ ORR pathways, respectively. **e** Two-step coupled selectivity map at 0.2 V (vs. RHE). **f** Scaling-relation analysis of intermediate adsorption free energies on dual-atom sites. **g** Side view of the charge density difference for H_2O_2 adsorbed on PSFZ/PSFM/PSFZM, the cyan region represents charge depletion, and the yellow region indicates charge accumulation. **h** Calculated density of states (DOS) analysis

for PSF/PSFZ/PSFZM after H_2O_2 adsorption. **i** Schematic diagram of the reaction pathway of O_2 generating ROS through free radical and non-free radical at the active domain of PSFZM. Colors represent: Pr (red), Sr (purple), Fe (yellow), Zn (green), Mo (blue).

Conventional descriptors, such as ΔG_{*OOH}, often fail to accurately capture the energetics of downstream product formation. Therefore, we introduced ΔG_{*H2O2}–ΔG_{*OOH} and ΔG_{*O}–ΔG_{*OOH} as more predictive descriptors for reaction selectivity (**Fig. 5d**). Mechanistic mapping shows that the PSFZM catalytic site is located in a specific region that favors the formation of H_2O_2 and •OH while effectively suppressing excessive reduction toward the *O intermediate. A coupled two-step descriptor map at 0.2 V versus RHE further shows that the Zn-O-Mo sites of PSFZM occupy the optimal region where peroxide formation and peroxide activation are balanced (**Fig. 5e**). In contrast, PSFZ stabilizes peroxide intermediates but lacks sufficient activation capability, leading to H_2O_2 accumulation, while PSFM promotes stronger O–O activation but does not efficiently retain the peroxide intermediate. In **Fig. 5f**, the two volcano branches describe the thermodynamic limitations associated with *H_2O_2 and *OOH formation, respectively. The volcano apex approaches the equilibrium potential of the O_2/H_2O_2 couple ($U° = 0.70$ V vs. RHE), defining the minimum theoretical overpotential imposed by the scaling relationship. The Zn–O–Mo motif in PSFZM lies near this optimum and gives the highest limiting potential among all examined motifs ($\Delta G_{*OOH} \approx 4.28$ eV, $U_L \approx 0.749$ V).This position indicates that the Zn–O–Mo active domain effectively balances *OOH stabilization with *H_2O_2 activation, thereby mitigating the conventional scaling constraint and promoting the confined conversion of surface-retained *H_2O_2 toward •OH.

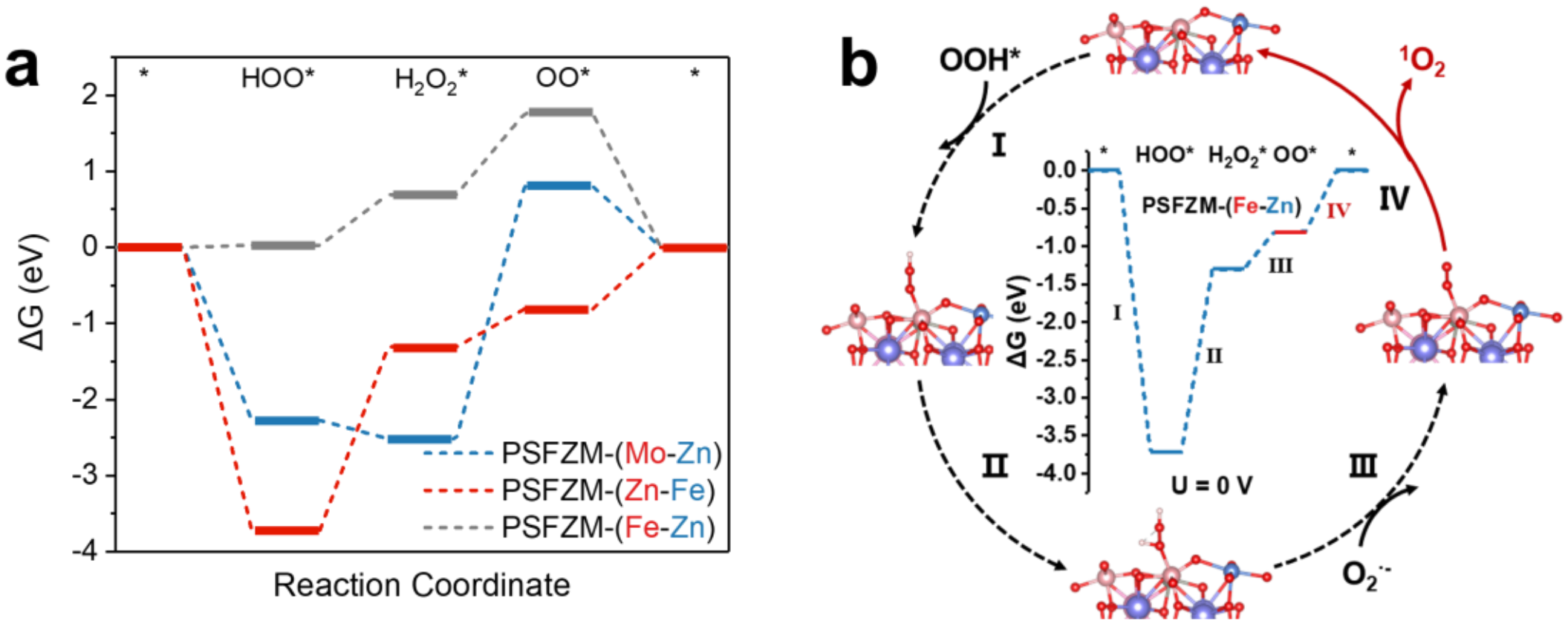

Supplementary Fig. 69 a Gibbs free energy of the whole reaction pathway for the

generation of $^1O_2$ via three-electron ORR for all samples. **b** Intermediate structures of oxidation $*O_2$ to $^1O_2$ over PSFZM and the corresponding free energy diagrams.

Given the experimental detection of $^1O_2$ via ESR, we also explored its formation mechanism. DFT results indicate that the conversion of $*H_2O_2$ to *OO is the rate-determining step for $^1O_2$ generation. Notably, the Zn-Fe site exhibits the lowest ΔG for this step (**Supplementary Fig. 69a**), with Fe enhancing *OOH adsorption and destabilizing the O-H bond to promote *OO formation (**Supplementary Fig. 69b**). This underscores the complementary roles of bimetallic Zn centers: Zn-Mo for •OH and Zn-Fe for $^1O_2$, collectively boosting ROS diversity and O_2 utilization.

Thermodynamically downhill adsorption on Zn-Zn and Zn-Mo sites supports efficient intermediate stabilization and •OH formation. Conversely, $^1O_2$ generation occurs preferentially at Zn-Fe sites at Zn-Fe sites, as $^1O_2$ formation is thermodynamically unfavorable at Zn-Mo domains. These results reinforce the selective functionality of the Fe co-catalyst in facilitating alternative ROS pathways.

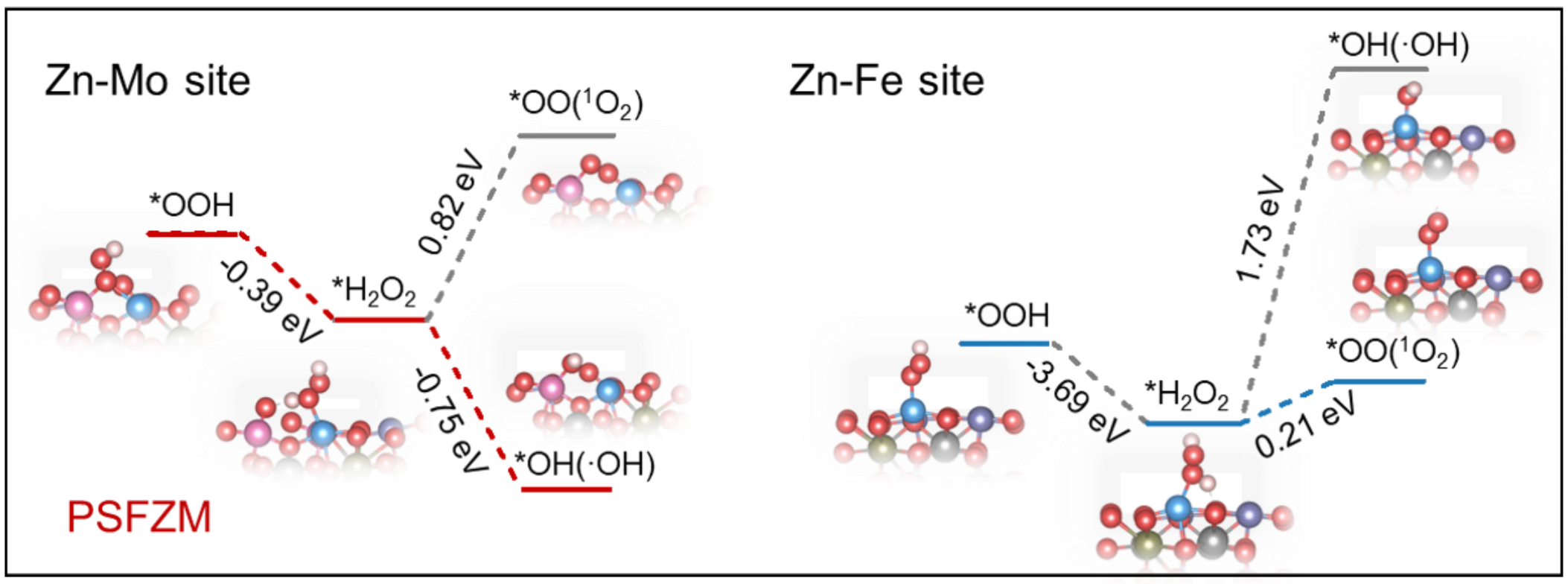

Supplementary Fig. 70 Comparison of free energies of adsorption of intermediate *OOH at all B sites in PSFZM. Comparison of the thermodynamics of $^1O_2$ and •OH generation at Zn-Mo and Zn-Fe sites in PSFZM. Colors represent: Pr (yellow), Sr (gray), Fe (purple), Zn (blue), Mo (pink).

Additionally, Mo incorporation upshifts the Zn d-band center (from -5.693 eV in PSF to -3.028 eV in PSFZM), thereby balancing *OO binding and accelerating ORR kinetics. Based on these results, two synergistic ROS formation pathways are proposed (**Supplementary Fig. 58**):

Pathway I (Zn-Mo site):

$$* + O_2 \rightarrow *O_2 \quad (6)$$

$$*O_2 + (H^+ + e^-) \rightarrow *OOH \quad (7)$$

$$*OOH + (H^+ + e^-) \rightarrow *H_2O_2 \quad (8)$$

$$*H_2O_2 + e^- \rightarrow \bullet OH + OH^- \quad (9)$$

Pathway II (Zn-Fe site):

$$*OOH + \bullet OH \rightarrow {}^1O_2 + H_2O \quad (10)$$

$$*OOH + H_2O_2 \rightarrow \bullet OH + {}^1O_2 + H_2O \quad (11)$$

5. As stated in Fig. 5c and earlier discussions, PSFZ follows a 2e- pathway, PSFM a 4e- pathway, and PSFZM a 3e- pathway. However, Fig. 5c indicates that PSFM also follows a 3e- pathway, which is inconsistent with both the figure caption and the preceding text. The authors should clarify this discrepancy.

Response: We thank the reviewer for pointing out this inconsistency. We agree that the original presentation of Fig. 5c was confusing and inconsistent with both the figure caption and the mechanistic discussion. After carefully rechecking the figure, we found that the indication that PSFM follows a $3e^-$ pathway in Fig. 5c resulted from an incorrect color annotation in the schematic, rather than from an error in the calculations or mechanistic interpretation. Obviously, in the previous version, Zn–Fe or Fe–Zn structural motifs should not appear in PSFM, which contains no Zn.

We have carefully checked the related text, figure caption and graphical labels to eliminate this discrepancy. In the revised manuscript, Fig. 5c and its caption have been corrected to ensure consistency with the experimental and computational results. PSFZ is now clearly assigned to the dominant $2e^-$ ORR pathway with H_2O_2 accumulation, most sites in PSFM are assigned to the dominant $4e^-$ ORR pathway toward H_2O formation, and PSFZM is assigned to the $3e^-$ ORR pathway toward •OH generation.

Manuscript Revision:

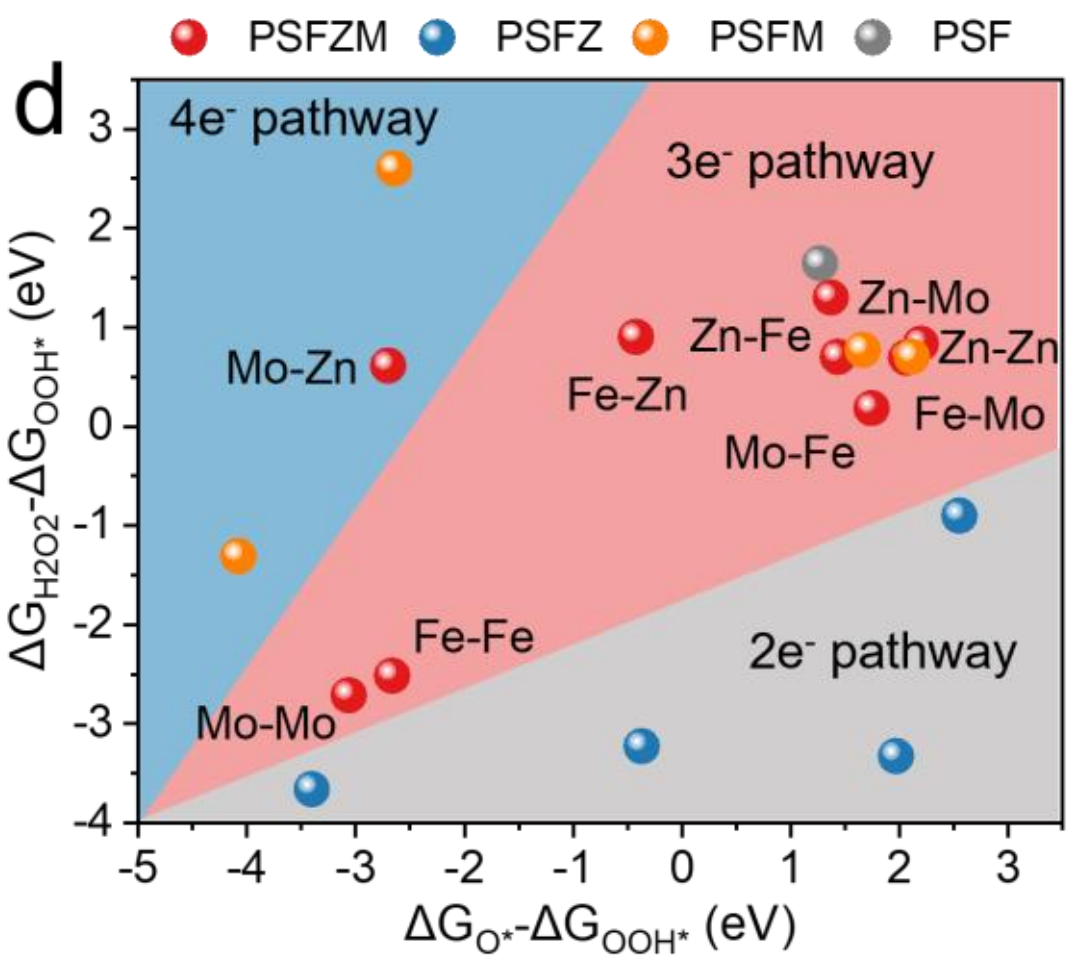

d Energy differences between reaction intermediates at equilibrium potential. The blue, red, and gray regions indicate active sites for the thermodynamically driven $4e^-$, $3e^-$, and $2e^-$ ORR pathways, respectively.

Conventional descriptors, such as ΔG_{*OOH}, often fail to accurately capture the energetics of downstream product formation. Therefore, we introduced ΔG_{*H2O2}–ΔG_{*OOH} and ΔG_{*O}–ΔG_{*OOH} as more predictive descriptors for reaction selectivity (**Fig. 5d**). Mechanistic mapping shows that the PSFZM catalytic site is located in a specific region that favors the formation of H_2O_2 and •OH while effectively suppressing excessive reduction toward the *O intermediate.

6. As central conclusion of the manuscript, the proposed 3e- oxygen-reduction pathway is not sufficiently supported by rigorous and complete data. The estimated electron-transfer number n≈3.0 is valid only within a limited potential window, without an n-versus-potential profile over the full potential range. The RRDE analysis does not correct for background currents and capacitive (pseudocapacitive) currents; moreover, the Koutecky-Levich treatment lacks adequate data points to substantiate a stable 3e- reaction mechanism. In addition, the authors have not provided a complete potential-dependent selectivity curve for H_2O_2.

Response: We sincerely thank the reviewer for the valuable comments and constructive suggestions. We agree with the reviewer that the RRDE analysis and Koutecky–Levich analysis in the original manuscript were incomplete and therefore insufficient to fully support the proposed three-electron oxygen reduction reaction pathway. In the revised manuscript,

we have substantially improved the electrochemical analysis and added more experimental data, thereby providing a more rigorous theoretical basis for the proposed reaction mechanism.

First, we added complete potential-dependent electron-transfer number and H_2O_2 selectivity curves in Fig. 3c, extending the potential window to 0 V (vs. RHE). Before calculating the electron-transfer number (n) and H_2O_2 selectivity, both the disk and ring currents were corrected as described above, and the n-versus-potential profile covering the full measured potential range is now presented. Second, the RRDE data in Fig. 3a have been corrected by subtracting the background current and capacitive/pseudocapacitive current. In addition, we provided the experimentally determined collection efficiency (N) and the corresponding measurement method in Supplementary Figs. 20 and 21 and Supplementary Note 10.

We also added LSV curves collected at higher rotation rates in Supplementary Fig. 22, which were used to recalculate the Koutecky–Levich slopes (Supplementary Fig. 23) and further verify the stability of the three-electron reaction mechanism. In addition, we supplemented the electrochemical data of PSF in Fig. 3a–3c to exclude the contribution of the parent sample to the $3e^-$ ORR pathway.

Manuscript Revision:

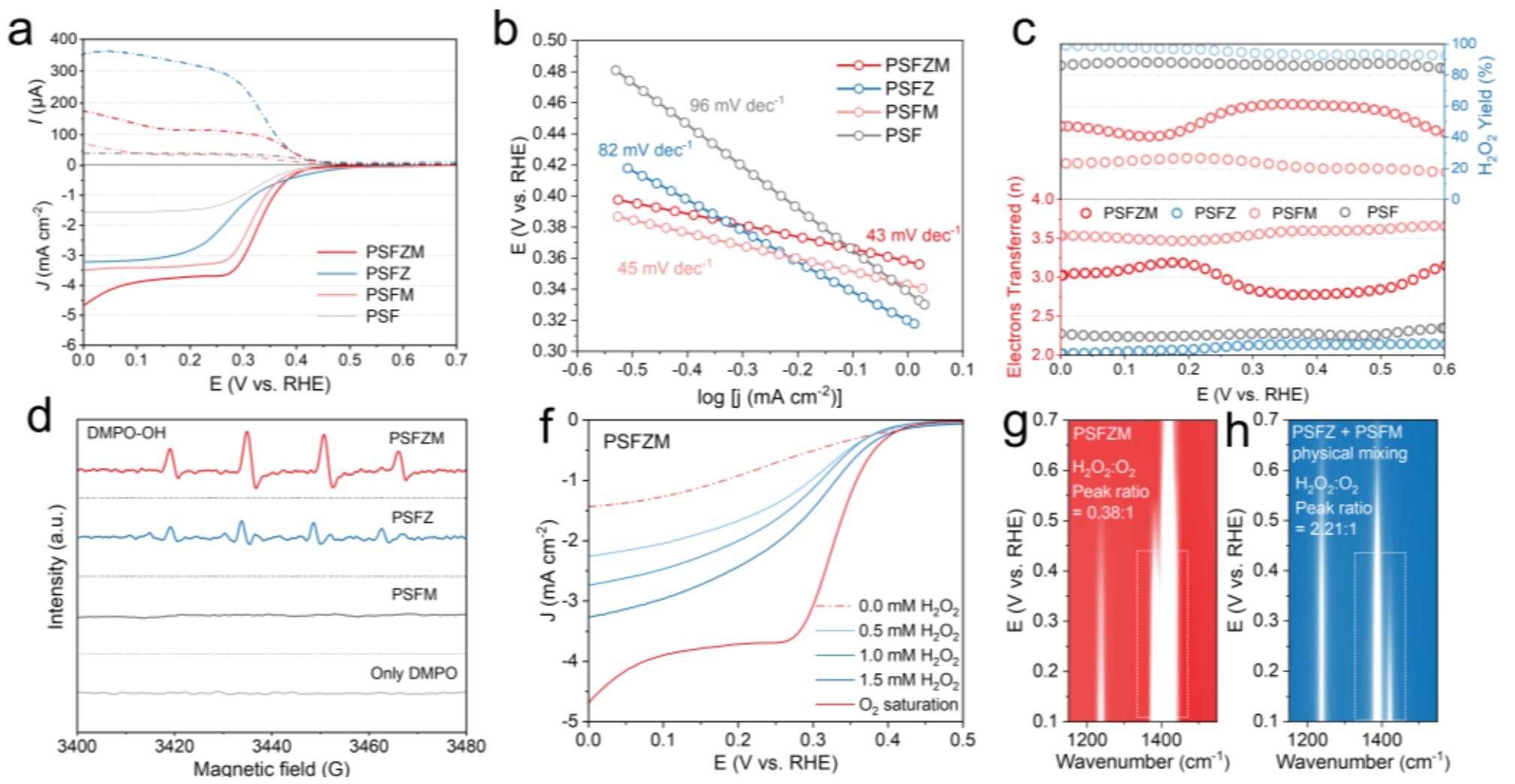

Fig. 3 Qualitative analysis of ORR selectivity and O_2 conversion to •OH. **a** RRDE polarization profiles for oxygen reduction reaction. Note: The solid lines are the disk current density corresponding to oxygen reduction and the dotted lines are the ring current density

corresponding to H_2O_2 oxidation. **b** The corresponding Tafel plots. **c** H_2O_2 selectivity and number of transferred electrons (n) calculated from RRDE polarization curves. **d** EPR spectra of DMPO-•OH adducts for samples from different catalyst-catalyzed electro-Fenton reactions. **e** DMPO-•OH adduct EPR spectra of ORR electrochemistry over PSFZM catalyst collected in an incremental voltage configuration. **f** LSV with different concentrations of H_2O_2 raw materials in the O_2-free configuration and the O_2-filled configuration. Presented are the potential-dependent in situ FTIR contour plots of **g** PSFZM and the **h** PSFZ+PSFM physical mixture, acquired in an O_2-saturated electrolyte.

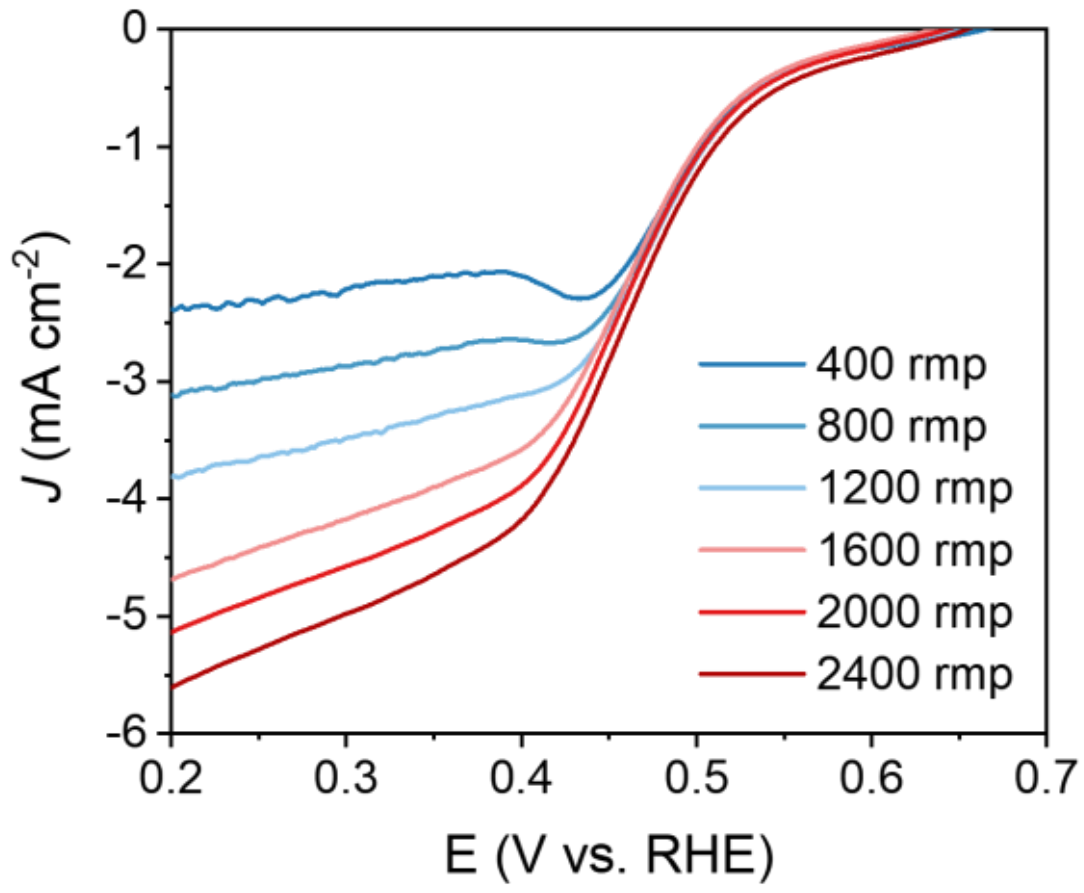

Supplementary Fig. 22 Oxygen reduction reaction profiles at 400 to 2400 rpm in O_2-saturated 1.0 M Na_2SO_4 for PSFZM.

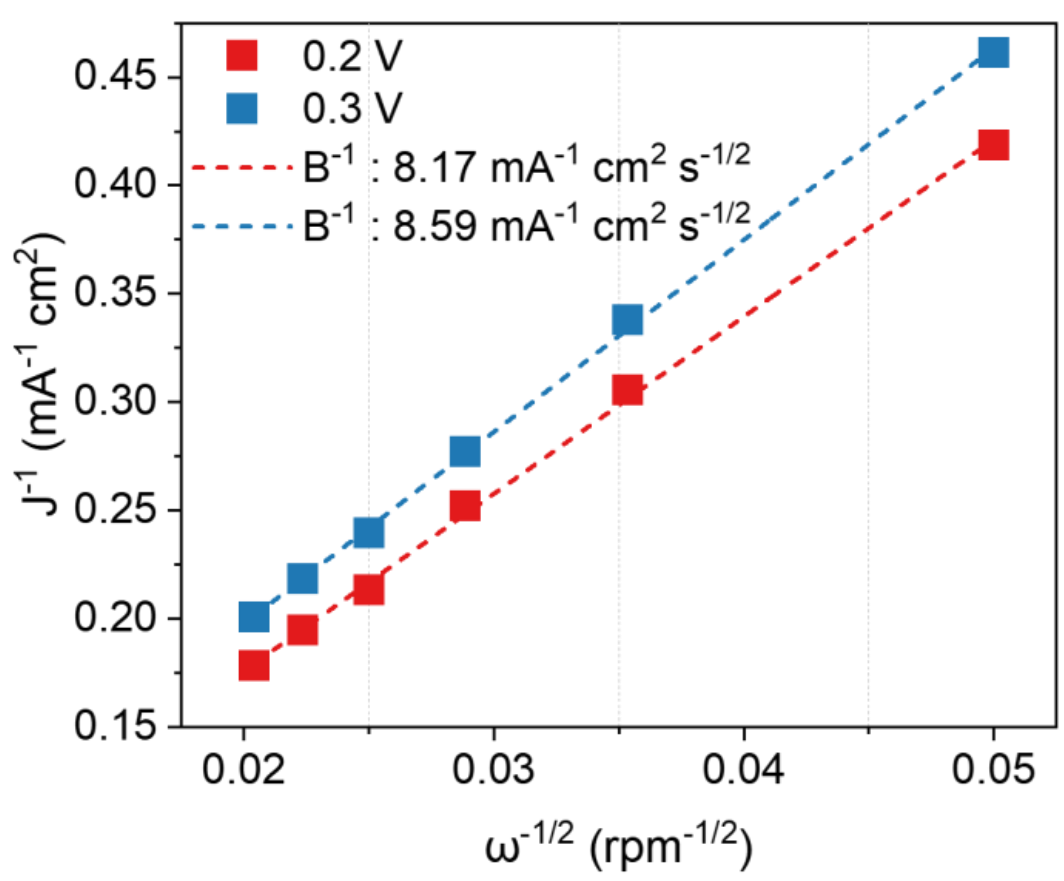

Supplementary Fig. 23 The corresponding Koutecky-Levich slope analysis for PSFZM.

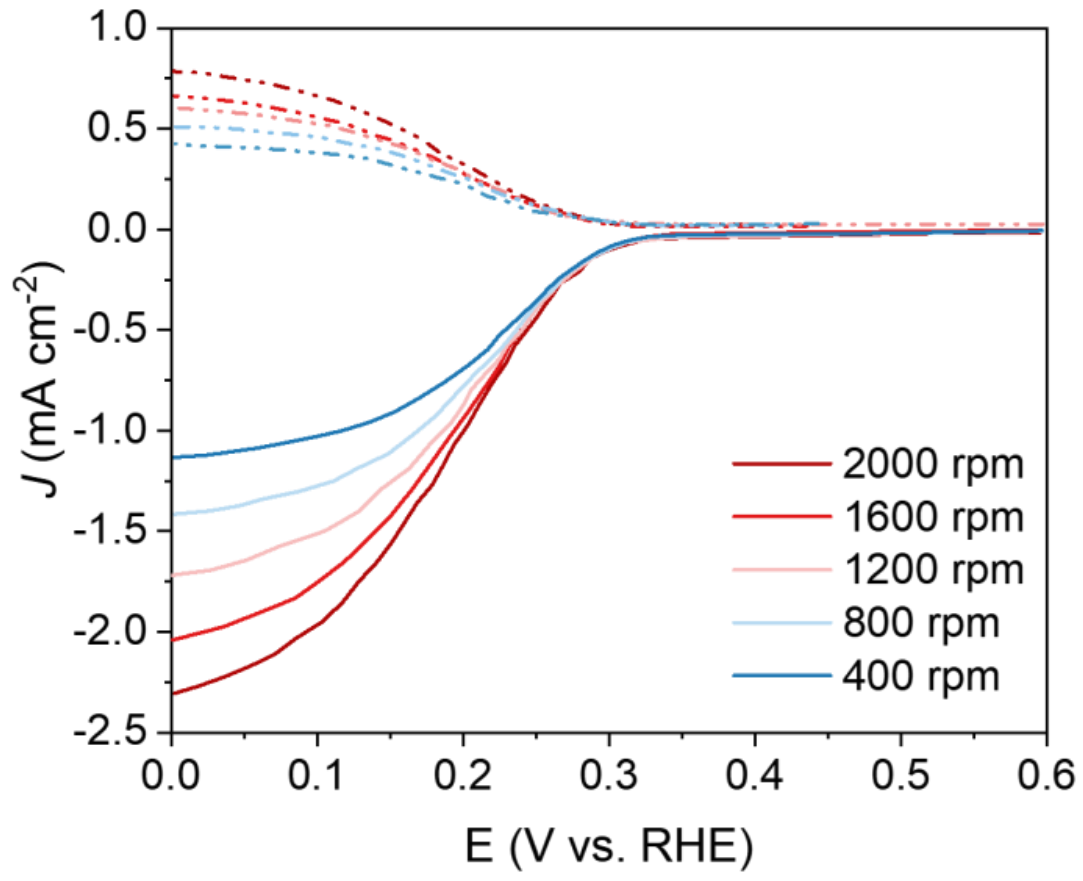

Supplementary Fig. 20 LSV curves in 10 mM $K_3[Fe(CN)_6]$ at different rotation speeds.

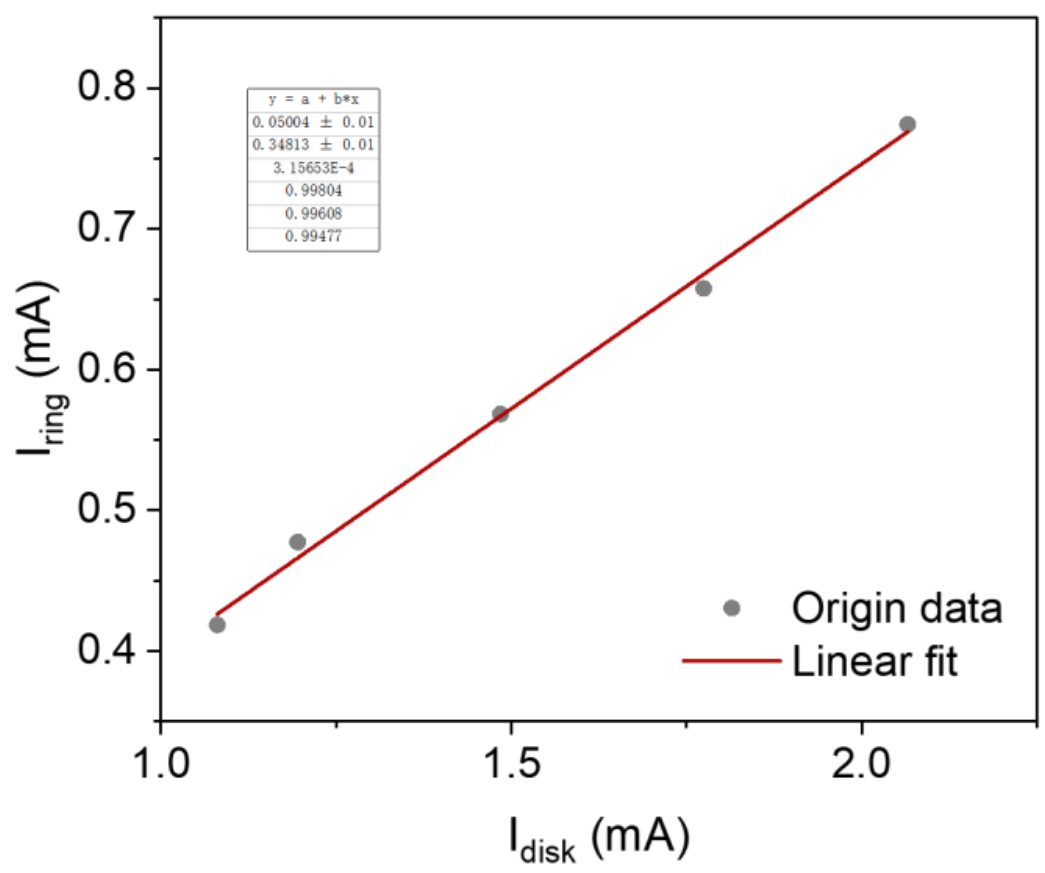

Supplementary Fig. 21 The collection efficiency (N) of RRDE, which was determined to be 34.8%.

Supplementary Note 10. Calibration of the RRDE collection efficiency (N).

The collection efficiency N of the RRDE was experimentally calibrated using the $[Fe(CN)_6]^{3-}/^{4-}$ redox couple in N_2-saturated electrolyte, following the protocol described previously[1]. The calibration electrolyte consisted of 10 mM $K_3[Fe(CN)_6]$ (≥99.0%, Sigma-Aldrich) prepared with ultrapure water (18.2 MΩ·cm, Milli-Q), and was deaerated by bubbling high-purity N_2 (≥99.999%) for at least 30 min prior to and throughout the measurements.

A three-electrode configuration was used, comprising a glassy carbon disk–platinum ring RRDE (disk diameter 4.0 mm; ring inner/outer diameter 5.0/7.0 mm; Pine Research) as the working electrode, a platinum wire as the counter electrode, and a saturated Hg/HgO electrode as the reference. Prior to each measurement, the glassy carbon disk was polished sequentially with 1.0, 0.3 and 0.05 μm alumina slurries (Buehler) to a mirror finish, followed by ultrasonication in ultrapure water.

Measurements were performed at five rotation rates (ω = 400, 800, 1200, 1600 and 2000 rpm). The electrode was preconditioned with five cyclic voltammetric scans at each rotation rate to ensure a stable interface, and the limiting plateau currents for both the disk and ring were extracted for analysis.

The collection efficiency was calculated as:

$$N = -I_{ring} / I_{disk}$$

where I_{ring} is the limiting oxidation current at the ring and I_{disk} is the limiting reduction current at the disk.

7. Some figures and tables contain unclear labeling (e.g., the symbol "L" in Fig. 4L is obscured), and the labeling or order of figures is used arbitrarily. The figure sequence should be reorganized to ensure a coherent logical flow. In addition, the performance literature comparisons are selective, relying on comparatively inferior catalysts rather than the most recent reports. Units should be kept consistent throughout. Overall, all figures, data presentations, and scientific terminology must be carefully revised.

Response: We thank the reviewer for pointing out these important issues regarding figure presentation, figure organization, literature comparison, unit consistency, and scientific terminology. We agree with the reviewer that the clarity and logical coherence of the figures in the original manuscript required improvement.

In the revised manuscript, we have carefully checked and revised all figures, tables, captions, and related descriptions, including the sequence and layout of Fig. 3d–3o and the caption order of Fig. 4, to improve readability and consistency. In addition, we have unified the labeling format throughout the manuscript and now use a consistent lowercase-letter style in both the figures and main text, for example, "Fig. 4l". Second, we removed comparisons with catalysts showing relatively poor performance and added recently published studies in the Supporting Information to provide more robust and up-to-date benchmarking, including

Nat. Commun. 17, 975 (2026), Angew. Chem. Int. Ed. 65, e8246739 (2026), and Nat. Commun. 16, 7908 (2025).

Regarding unit revision throughout the manuscript, we have unified the production rate in the main text as 821 μmol h^{-1}. For comparisons among different systems, including PMS- and H_2O_2-based systems, we converted the rates to μmol L^{-1} min^{-1} and clarified this conversion in the table footnote.

Manuscript Revision:

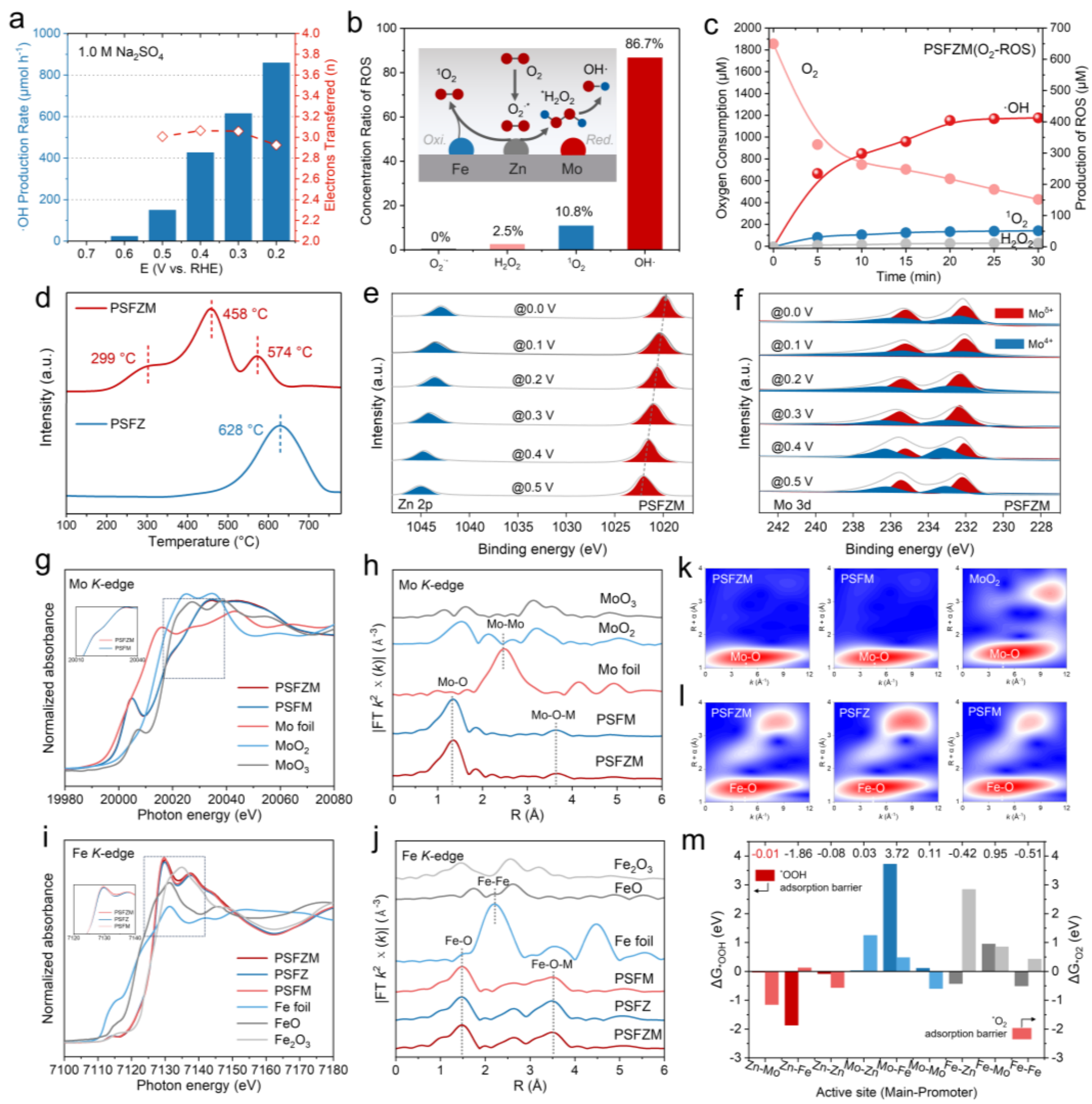

Fig. 4 Mechanism of dual co-catalytic sites promoting ROS formation. a Benzoic acid fluorescence spectrophotometry, used to detect the amount of •OH free radicals generated. •OH yield at different oxygen reduction voltages. **b** The proportion of ROS generated by PSFZM through 3e⁻ ORR, and the inset shows the single-site active domain mechanism. **c**

Oxygen utilization during 30-min continuous testing. Quantitative determination of the consumed dissolved O_2 and generated ROS. **d** O_2-TPD profiles of PSFZM and PSFZ. In-situ XPS spectra of **e** Zn 2p and **f** Mo 3d in electrochemistry. **g** Mo K-edge XANES spectra of PSFZM, PSFM, Mo foil, MoO_2 and MoO_3. **h** Mo K-edge k^3-weighted FT-EXAFS spectra. **i** Fe K-edge XANES spectra of PSFZM, PSFZ, PSFM, Fe foil, FeO and Fe_2O_3. **j** Fe K-edge k^3-weighted FT-EXAFS spectra. WT-EXAFS spectra of **k** Mo K-edge and **l** Fe K-edge. **m** Comparison of free energies of adsorption of intermediate *OOH and O_2 at B sites in PSFZM.

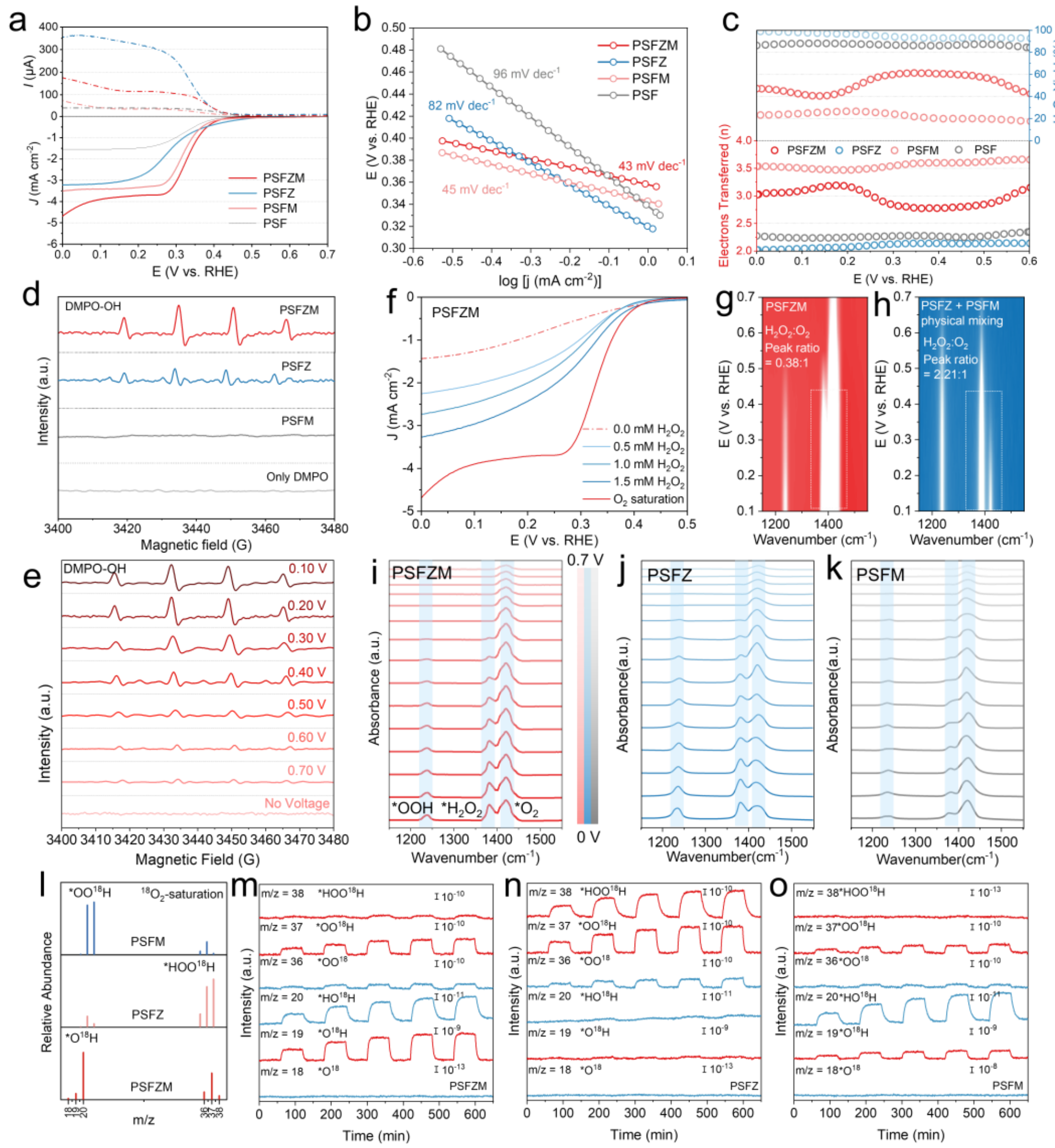

Fig. 3 Qualitative analysis of ORR selectivity and O_2 conversion to •OH. **a** RRDE polarization profiles for oxygen reduction reaction. Note: The solid lines are the disk current

density corresponding to oxygen reduction and the dotted lines are the ring current density corresponding to H_2O_2 oxidation. **b** The corresponding Tafel plots. **c** H_2O_2 selectivity and number of transferred electrons (n) calculated from RRDE polarization curves. **d** EPR spectra of DMPO-•OH adducts for samples from different catalyst-catalyzed electro-Fenton reactions. **e** DMPO-•OH adduct EPR spectra of ORR electrochemistry over PSFZM catalyst collected in an incremental voltage configuration. **f** LSV with different concentrations of H_2O_2 raw materials in the O_2-free configuration and the O_2-filled configuration. Presented are the potential-dependent in situ FTIR contour plots of **g** PSFZM and the **h** PSFZ+PSFM physical mixture, acquired in an O_2-saturated electrolyte. The ORR electrochemical in-situ FTIR spectra of **i** PSFZM, **j** PSFZ, and **k** PSFM catalyst collected in an incremental voltage configuration. **l** Representative mass spectra of PSFZM, PSFZ, and PSFM, recorded under conditions saturated with $^{18}O_2$, reveal distinct ^{18}O-labeled oxygen reduction products. Time-resolved DEMS traces of selected isotope-labeled fragments, including peroxide/hydroperoxide-related channels and hydroxyl/water-related channels, during intermittent electrolysis over **m** PSFZM, **n** PSFZ, and **o** PSFM.

Supplementary Table 7. Comparison of ROS generation rates of different generation systems was determined.

Catalysts	Type	ROS generation rate ($\mu mol \cdot L^{-1} \cdot min^{-1}$)	Ref.
**PSFZM**	O_2 activation	228.06 (821 $\mu mol\ h^{-1}$)	This work
**CSR-TiO$_2$**	O_2 activation	148.26	[18]
**g-C$_3$N$_4$-CoWO**	H_2O_2 activation	3.3	[19]
**Mo-LDHs**	H_2O_2 activation	5.7	[20]
**CuCo LDH/BC**	H_2O_2 activation	6.9	[21]
**45Fe-POM/CNNS-N$_{vac.}$**	H_2O_2 activation	9.2	[22]
**Fe(III)/CaSO$_3$**	PMS activation	14.8	[23]

**DSLB-800**	PMS activation	19.5	[24]
**FeN_4/B**	PMS activation	227	[25]
**β-MnO_2**	PMS activation	29.5	[26]
**Cu-SA/Ti_3C_2Tx**	PMS activation	30.2	[27]
**$BiOIO_3/\gamma$-FeOOH**	O_2 activation	0.635	[28]
**Bi/Bi_2WO_{6-x}**	O_2 activation	3.6	[29]
**CMCN**	O_2 activation	17.0	[30]
**Trimethoprim**	O_2 activation	13.5	[31]
**iron phosphide**	O_2 activation	20.9	[32]
**SA-$Cu_{1.0}$CA**	O_2 activation	65.3	[12]

Note: To facilitate direct comparison with other systems, the units of yield have been converted.

Supplementary References

25. Zhang, T. *et al.* Cooperative Spin Alignment Enhances Selective Singlet Oxygen Generation in Wastewater Pre-Oxidation System. *Angew. Chemie Int. Ed.* **65**, e8246739 (2026).

28. Xu, J., *et al.* Piezo-catalytic in-site H_2O_2 generation and activation across wide pH range to drive hydroxyl radical-mediated pollutant degradation. *Nat Commun* **16**, 7908 (2025).

30. Chen, X., *et al.* Ultrafast energy-neutral molecular oxygen activation via atomically-adjacent bimetallic catalytic sites. *Nat Commun* **17**, 975 (2026).

References

34. Li, Y. *et al.* Scalable and Customizable Single-Atom Coatings for pH-Universal H_2O_2

Electrosynthesis. *Adv. Mater.* **38**, e21237 (2026).
