# Supplementary material for: Single-atom-engineered perovskite enables near-theoretical-rate hydroxyl radical electrogeneration
Source: Nat Commun. 2026 Jul 24;17:9026. doi: 10.1038/s41467-026-75926-5 (PMC13507266; doi:10.1038/s41467-026-75926-5)
Supplement: Supplementary file 4 [file 41467_2026_75926_MOESM3_ESM.docx]

**Supplementary data 1**

The atomic coordination of model in the Manuscript and SI.

**The optimized configurations of PSFZM-Fe-Fe with adsorption *OOH**

psfzm-fe-fe

1.0

7.7817001343	0.0000000000	0.0000000000

0.0000000000	7.7557001114	0.0000000000

0.0000000000	0.0000000000	23.3838996887

O	Pr	Sr	Fe	Zn	Mo	H

34	6	2	6	3	3	1

Direct

0.829559982	0.456699997	0.096019998

0.322510004	0.471210003	0.264319986

0.040819999	0.543299973	0.000520000

0.543850005	0.543299973	0.166899994

0.046510000	0.500829995	0.345389992

0.255259991	0.956700027	0.096120000

0.762229979	0.972199976	0.256430000

0.540970027	0.043299999	0.025240000

0.044009998	0.043299999	0.191630006

0.636200011	0.873139977	0.361710012

0.255259991	0.543299973	0.096120000

0.773479998	0.554340005	0.268409997

0.540970027	0.456699997	0.025240000

0.044009998	0.456699997	0.191630006

0.543309987	0.499269992	0.352340013

0.829559982	0.043299999	0.096019998

0.364459991	0.029740000	0.268040001

0.040819999	0.956700027	0.000520000

0.543850005	0.956700027	0.166899994

0.016130000	0.945680022	0.341329992

0.765829980	0.750000000	0.025760001

0.268869996	0.750000000	0.192139998

0.847850025	0.002520000	0.449979991

0.252350003	0.250000000	0.021299999

0.755379975	0.250000000	0.187690005

0.310649991	0.239989996	0.354750007

0.815959990	0.250000000	0.000000000

0.319000006	0.250000000	0.166390002

0.770659983	0.221120000	0.352070004

0.329439998	0.750000000	0.004460000

0.832480013	0.750000000	0.170849994

0.292829990	0.741710007	0.333840013

0.290730000	0.503180027	0.436019987

0.261310011	0.622950017	0.484310001

0.030670000	0.750000000	0.090999998

0.519909978	0.742709994	0.263080001

0.557730019	0.250000000	0.099950001

0.054159999	0.250000000	0.101149999

0.546540022	0.267820001	0.276809990

0.047470000	0.708570004	0.265630007

0.078599997	0.240879998	0.273119986

0.527090013	0.750000000	0.092200004

0.790899992	0.500000000	0.012880000

0.290899992	0.500000000	0.012880000

0.299580008	0.493589997	0.350600004

0.293929994	0.500000000	0.179269999

0.794110000	0.493120015	0.360020012

0.289229989	0.990360022	0.350939989

0.793929994	0.500000000	0.179269999

0.790899992	0.000000000	0.012880000

0.293929994	0.000000000	0.179269999

0.815890014	0.003240000	0.377570003

0.290899992	0.000000000	0.012880000

0.793929994	0.000000000	0.179269999

0.319889992	0.532079995	0.515370011

**The optimized configurations of PSFZM-Fe-Mo with adsorption *OOH**

psfzm-fe-mo

1.0

7.7817001343	0.0000000000	0.0000000000

0.0000000000	7.7557001114	0.0000000000

0.0000000000	0.0000000000	23.3838996887

O	Pr	Sr	Fe	Zn	Mo	H

34	6	2	6	3	3	1

Direct

0.829559982	0.456699997	0.096019998

0.350879997	0.458050013	0.258020014

0.040819999	0.543299973	0.000520000

0.543850005	0.543299973	0.166899994

0.035100002	0.526859999	0.334509999

0.255259991	0.956700027	0.096120000

0.758870006	0.965550005	0.273820013

0.540970027	0.043299999	0.025240000

0.044009998	0.043299999	0.191630006

0.520950019	0.987269998	0.379740000

0.255259991	0.543299973	0.096120000

0.764789999	0.568949997	0.273950011

0.540970027	0.456699997	0.025240000

0.044009998	0.456699997	0.191630006

0.558990002	0.477319986	0.355870008

0.829559982	0.043299999	0.096019998

0.362219989	0.045820002	0.271169990

0.040819999	0.956700027	0.000520000

0.543850005	0.956700027	0.166899994

0.061299998	0.970889986	0.346230000

0.765829980	0.750000000	0.025760001

0.268869996	0.750000000	0.192139998

0.795970023	0.710879982	0.392679989

0.252350003	0.250000000	0.021299999

0.755379975	0.250000000	0.187690005

0.277289987	0.238079995	0.359039992

0.815959990	0.250000000	0.000000000

0.319000006	0.250000000	0.166390002

0.812049985	0.264129996	0.348280013

0.329439998	0.750000000	0.004460000

0.832480013	0.750000000	0.170849994

0.310420007	0.758310020	0.322510004

0.334050000	0.557869971	0.428810000

0.383500010	0.423929989	0.470779985

0.030670000	0.750000000	0.090999998

0.535960019	0.765730023	0.247970000

0.557730019	0.250000000	0.099950001

0.054159999	0.250000000	0.101149999

0.589129984	0.249909997	0.270220011

0.022919999	0.770770013	0.259099990

0.055879999	0.247759998	0.272049993

0.527090013	0.750000000	0.092200004

0.790899992	0.500000000	0.012880000

0.290899992	0.500000000	0.012880000

0.298570007	0.504630029	0.345959991

0.798200011	0.988390028	0.363700002

0.290899992	0.000000000	0.012880000

0.793929994	0.000000000	0.179269999

0.793929994	0.500000000	0.179269999

0.790899992	0.000000000	0.012880000

0.293929994	0.000000000	0.179269999

0.293929994	0.500000000	0.179269999

0.793730021	0.516700029	0.352189988

0.309949994	0.002930000	0.348089993

0.355910003	0.497180015	0.510510027

**The optimized configurations of PSFZM-Fe-Zn with adsorption *OOH**

psfzm-fe-zn

1.0

7.7817001343	0.0000000000	0.0000000000

0.0000000000	7.7557001114	0.0000000000

0.0000000000	0.0000000000	23.3838996887

O	Pr	Sr	Fe	Mo	Zn	H

34	6	2	6	3	3	1

Direct

0.829559982	0.456699997	0.096019998

0.307550013	0.483200014	0.269030005

0.040819999	0.543299973	0.000520000

0.543850005	0.543299973	0.166899994

0.035969999	0.507340014	0.346100003

0.255259991	0.956700027	0.096120000

0.757319987	0.967710018	0.254669994

0.540970027	0.043299999	0.025240000

0.044009998	0.043299999	0.191630006

0.644339979	0.869170010	0.361669987

0.255259991	0.543299973	0.096120000

0.771600008	0.549409986	0.262210011

0.540970027	0.456699997	0.025240000

0.044009998	0.456699997	0.191630006

0.545499980	0.506479979	0.348749995

0.829559982	0.043299999	0.096019998

0.351980001	0.027389999	0.261220008

0.040819999	0.956700027	0.000520000

0.543850005	0.956700027	0.166899994

0.013620000	0.955049992	0.336560011

0.765829980	0.750000000	0.025760001

0.268869996	0.750000000	0.192139998

0.851459980	0.038929999	0.444840014

0.252350003	0.250000000	0.021299999

0.755379975	0.250000000	0.187690005

0.309879988	0.238739997	0.353289992

0.815959990	0.250000000	0.000000000

0.319000006	0.250000000	0.166390002

0.768580019	0.225620002	0.340460002

0.329439998	0.750000000	0.004460000

0.832480013	0.750000000	0.170849994

0.292329997	0.750230014	0.338569999

0.305330008	0.513530016	0.439770013

0.382330000	0.399760008	0.483649999

0.030670000	0.750000000	0.090999998

0.515519977	0.737559974	0.268059999

0.557730019	0.250000000	0.099950001

0.054159999	0.250000000	0.101149999

0.527310014	0.273220003	0.274879992

0.053950001	0.705009997	0.267289996

0.063720003	0.250120014	0.275570005

0.527090013	0.750000000	0.092200004

0.790899992	0.500000000	0.012880000

0.290899992	0.500000000	0.012880000

0.793929994	0.500000000	0.179269999

0.296119988	0.496069998	0.354490012

0.790899992	0.000000000	0.012880000

0.293929994	0.000000000	0.179269999

0.814599991	0.015920000	0.373299986

0.290899992	0.000000000	0.012880000

0.793929994	0.000000000	0.179269999

0.293929994	0.500000000	0.179269999

0.790459991	0.494729996	0.352589995

0.284539998	0.993730009	0.351069987

0.357910007	0.457789987	0.526269972

**The optimized configurations of PSFZM-Mo-Fe with adsorption *OOH**

psfzm-mo-fe

1.0

7.7817001343	0.0000000000	0.0000000000

0.0000000000	7.7557001114	0.0000000000

0.0000000000	0.0000000000	23.3838996887

O	Pr	Sr	Fe	Mo	Zn	H

34	6	2	6	3	3	1

Direct

0.829559982	0.456699997	0.096019998

0.356279999	0.459910005	0.269190013

0.040819999	0.543299973	0.000520000

0.543850005	0.543299973	0.166899994

0.066140004	0.516629994	0.343019992

0.255259991	0.956700027	0.096120000

0.767059982	0.965690017	0.261839986

0.540970027	0.043299999	0.025240000

0.044009998	0.043299999	0.191630006

0.553669989	0.017000001	0.354970008

0.255259991	0.543299973	0.096120000

0.753960013	0.542620003	0.272529989

0.540970027	0.456699997	0.025240000

0.044009998	0.456699997	0.191630006

0.528249979	0.503329992	0.374320000

0.829559982	0.043299999	0.096019998

0.350600004	0.041949999	0.265610009

0.040819999	0.956700027	0.000520000

0.543850005	0.956700027	0.166899994

0.054979999	0.983770013	0.334320009

0.765829980	0.750000000	0.025760001

0.268869996	0.750000000	0.192139998

0.810509980	0.751309991	0.363819987

0.252350003	0.250000000	0.021299999

0.755379975	0.250000000	0.187690005

0.286060005	0.260360003	0.360210001

0.815959990	0.250000000	0.000000000

0.319000006	0.250000000	0.166390002

0.805360019	0.257869989	0.343769997

0.329439998	0.750000000	0.004460000

0.832480013	0.750000000	0.170849994

0.310279995	0.741609991	0.328839988

0.299490005	0.546310008	0.432080001

0.197710007	0.594280005	0.483119994

0.030670000	0.750000000	0.090999998

0.525749981	0.751659989	0.252279997

0.557730019	0.250000000	0.099950001

0.054159999	0.250000000	0.101149999

0.580200016	0.238800004	0.276969999

0.028620001	0.747250021	0.269369990

0.059340000	0.247240007	0.271880001

0.527090013	0.750000000	0.092200004

0.790899992	0.500000000	0.012880000

0.800509989	0.992060006	0.353439987

0.290899992	0.000000000	0.012880000

0.293929994	0.500000000	0.179269999

0.798179984	0.512350023	0.352880001

0.308490008	0.996580005	0.344090015

0.290899992	0.500000000	0.012880000

0.312079996	0.491880000	0.346399993

0.793929994	0.000000000	0.179269999

0.793929994	0.500000000	0.179269999

0.790899992	0.000000000	0.012880000

0.293929994	0.000000000	0.179269999

0.281210005	0.586690009	0.521529973

**The optimized configurations of PSFZM-Mo-Mo with adsorption *OOH**

psfzm-mo-mo

1.0

7.7817001343	0.0000000000	0.0000000000

0.0000000000	7.7557001114	0.0000000000

0.0000000000	0.0000000000	23.3838996887

O	Pr	Sr	Fe	Zn	Mo	H

34	6	2	6	3	3	1

Direct

0.829559982	0.456699997	0.096019998

0.349700004	0.464639992	0.267329991

0.040819999	0.543299973	0.000520000

0.543850005	0.543299973	0.166899994

0.052970000	0.515079975	0.340849996

0.255259991	0.956700027	0.096120000

0.756680012	0.966600001	0.271990001

0.540970027	0.043299999	0.025240000

0.044009998	0.043299999	0.191630006

0.515169978	0.997539997	0.382380009

0.255259991	0.543299973	0.096120000

0.761349976	0.558669984	0.277240008

0.540970027	0.456699997	0.025240000

0.044009998	0.456699997	0.191630006

0.533439994	0.495440006	0.366290003

0.829559982	0.043299999	0.096019998

0.353040010	0.022530001	0.273369998

0.040819999	0.956700027	0.000520000

0.543850005	0.956700027	0.166899994

0.058649998	0.980859995	0.351449996

0.765829980	0.750000000	0.025760001

0.268869996	0.750000000	0.192139998

0.797450006	0.710030019	0.392749995

0.252350003	0.250000000	0.021299999

0.755379975	0.250000000	0.187690005

0.280710012	0.252110004	0.356979996

0.815959990	0.250000000	0.000000000

0.319000006	0.250000000	0.166390002

0.789030015	0.266589999	0.349750012

0.329439998	0.750000000	0.004460000

0.832480013	0.750000000	0.170849994

0.307999998	0.744239986	0.340209991

0.282429993	0.548179984	0.430999994

0.348280013	0.434980005	0.476999998

0.030670000	0.750000000	0.090999998

0.537079990	0.755410016	0.242599994

0.557730019	0.250000000	0.099950001

0.054159999	0.250000000	0.101149999

0.569180012	0.236900002	0.260129988

0.023750000	0.756460011	0.249939993

0.040619999	0.243599996	0.266550004

0.527090013	0.750000000	0.092200004

0.790899992	0.500000000	0.012880000

0.290899992	0.500000000	0.012880000

0.787710011	0.986819983	0.362199992

0.290899992	0.000000000	0.012880000

0.793929994	0.000000000	0.179269999

0.293929994	0.500000000	0.179269999

0.793929994	0.500000000	0.179269999

0.790899992	0.000000000	0.012880000

0.293929994	0.000000000	0.179269999

0.306430012	0.494459987	0.345539987

0.797940016	0.510930002	0.355309993

0.301770002	0.995270014	0.351630002

0.321319997	0.488869995	0.520049989

**The optimized configurations of PSFZM-Mo-Zn with adsorption *OOH**

psfzm-mo-zn

1.0

7.7817001343	0.0000000000	0.0000000000

0.0000000000	7.7557001114	0.0000000000

0.0000000000	0.0000000000	23.3838996887

O	Pr	Sr	Fe	Mo	Zn

32	6	2	6	3	3

Direct

0.829559982	0.456699997	0.096019998

0.329679996	0.475279987	0.262939990

0.040819999	0.543299973	0.000520000

0.543850005	0.543299973	0.166899994

0.052749999	0.510919988	0.339489996

0.255259991	0.956700027	0.096120000

0.767679989	0.966220021	0.258969992

0.540970027	0.043299999	0.025240000

0.044009998	0.043299999	0.191630006

0.550679982	0.998210013	0.344689995

0.255259991	0.543299973	0.096120000

0.767340004	0.530250013	0.258249998

0.540970027	0.456699997	0.025240000

0.044009998	0.456699997	0.191630006

0.539279997	0.502279997	0.347310007

0.829559982	0.043299999	0.096019998

0.332450002	0.025830001	0.257499993

0.040819999	0.956700027	0.000520000

0.543850005	0.956700027	0.166899994

0.033890001	0.981909990	0.337769985

0.765829980	0.750000000	0.025760001

0.268869996	0.750000000	0.192139998

0.788079977	0.753939986	0.346599996

0.252350003	0.250000000	0.021299999

0.755379975	0.250000000	0.187690005

0.301730007	0.257820010	0.350899994

0.815959990	0.250000000	0.000000000

0.319000006	0.250000000	0.166390002

0.793060005	0.237590000	0.340790004

0.329439998	0.750000000	0.004460000

0.832480013	0.750000000	0.170849994

0.293130010	0.743610024	0.333909988

0.030670000	0.750000000	0.090999998

0.528389990	0.747820020	0.265410006

0.557730019	0.250000000	0.099950001

0.054159999	0.250000000	0.101149999

0.549849987	0.251800001	0.276450008

0.029570000	0.742030025	0.267760009

0.055399999	0.248649999	0.272920012

0.527090013	0.750000000	0.092200004

0.790899992	0.500000000	0.012880000

0.793929994	0.500000000	0.179269999

0.790899992	0.000000000	0.012880000

0.293929994	0.000000000	0.179269999

0.792990029	0.994310021	0.344419986

0.290899992	0.000000000	0.012880000

0.290899992	0.500000000	0.012880000

0.295399994	0.498860002	0.344709992

0.793929994	0.000000000	0.179269999

0.293929994	0.500000000	0.179269999

0.797720015	0.496270001	0.352169991

0.290369987	0.998430014	0.350089997

**The optimized configurations of PSFZM-Zn-Fe with adsorption *OOH**

psfzm-zn-fe

1.0

7.7817001343	0.0000000000	0.0000000000

0.0000000000	7.7557001114	0.0000000000

0.0000000000	0.0000000000	23.3838996887

O	Pr	Sr	Fe	Mo	Zn	H

34	6	2	6	3	3	1

Direct

0.829559982	0.456699997	0.096019998

0.325839996	0.481420010	0.261940002

0.040819999	0.543299973	0.000520000

0.543850005	0.543299973	0.166899994

0.039280001	0.505240023	0.345820010

0.255259991	0.956700027	0.096120000

0.765389979	0.969940007	0.256410003

0.540970027	0.043299999	0.025240000

0.044009998	0.043299999	0.191630006

0.550109982	0.992479980	0.349059999

0.255259991	0.543299973	0.096120000

0.772149980	0.529209971	0.265509993

0.540970027	0.456699997	0.025240000

0.044009998	0.456699997	0.191630006

0.549719989	0.498059988	0.352100015

0.829559982	0.043299999	0.096019998

0.338259995	0.019120000	0.264780015

0.040819999	0.956700027	0.000520000

0.543850005	0.956700027	0.166899994

0.044480000	0.984279990	0.335220009

0.765829980	0.750000000	0.025760001

0.268869996	0.750000000	0.192139998

0.792699993	0.750320017	0.348459989

0.252350003	0.250000000	0.021299999

0.755379975	0.250000000	0.187690005

0.301140010	0.238260001	0.354339987

0.815959990	0.250000000	0.000000000

0.319000006	0.250000000	0.166390002

0.794690013	0.244650006	0.340860009

0.329439998	0.750000000	0.004460000

0.832480013	0.750000000	0.170849994

0.292279989	0.749949992	0.341839999

0.303939998	0.529209971	0.441320002

0.363649994	0.403219998	0.484499991

0.030670000	0.750000000	0.090999998

0.527869999	0.734780014	0.276210010

0.557730019	0.250000000	0.099950001

0.054159999	0.250000000	0.101149999

0.538800001	0.256630003	0.283549994

0.050140001	0.725669980	0.274890006

0.064450003	0.256089985	0.276239991

0.527090013	0.750000000	0.092200004

0.790899992	0.500000000	0.012880000

0.797950029	0.995000005	0.348399997

0.290899992	0.000000000	0.012880000

0.293929994	0.500000000	0.179269999

0.794889987	0.500100017	0.348239988

0.295989990	0.997600019	0.346340001

0.290899992	0.500000000	0.012880000

0.790899992	0.000000000	0.012880000

0.793929994	0.000000000	0.179269999

0.793929994	0.500000000	0.179269999

0.292629987	0.494410008	0.358819991

0.293929994	0.000000000	0.179269999

0.335200012	0.443120003	0.529100001

**The optimized configurations of PSFZM-Zn-Mo with adsorption *OOH**

psfzm-zn-mo

1.0

7.7817001343	0.0000000000	0.0000000000

0.0000000000	7.7557001114	0.0000000000

0.0000000000	0.0000000000	23.3838996887

O	Pr	Sr	Fe	Zn	Mo	H

34	6	2	6	3	3	1

Direct

0.829559982	0.456699997	0.096019998

0.351090014	0.455969989	0.258069992

0.040819999	0.543299973	0.000520000

0.543850005	0.543299973	0.166899994

0.038050000	0.526489973	0.333139986

0.255259991	0.956700027	0.096120000

0.759779990	0.965950012	0.272929996

0.540970027	0.043299999	0.025240000

0.044009998	0.043299999	0.191630006

0.523710012	0.988099992	0.378430009

0.255259991	0.543299973	0.096120000

0.766579986	0.569440007	0.274019986

0.540970027	0.456699997	0.025240000

0.044009998	0.456699997	0.191630006

0.556529999	0.481370002	0.354640007

0.829559982	0.043299999	0.096019998

0.361530006	0.047580000	0.271129996

0.040819999	0.956700027	0.000520000

0.543850005	0.956700027	0.166899994

0.060729999	0.970049977	0.346060008

0.765829980	0.750000000	0.025760001

0.268869996	0.750000000	0.192139998

0.799589992	0.707229972	0.394199997

0.252350003	0.250000000	0.021299999

0.755379975	0.250000000	0.187690005

0.276050001	0.236489996	0.359580010

0.815959990	0.250000000	0.000000000

0.319000006	0.250000000	0.166390002

0.810850024	0.265009999	0.348679990

0.329439998	0.750000000	0.004460000

0.832480013	0.750000000	0.170849994

0.311580002	0.760730028	0.322010010

0.290549994	0.571129978	0.424840003

0.381770015	0.511250019	0.476700008

0.030670000	0.750000000	0.090999998

0.536689997	0.764850020	0.248109996

0.557730019	0.250000000	0.099950001

0.054159999	0.250000000	0.101149999

0.588419974	0.251459986	0.270529985

0.023690000	0.768429995	0.259429991

0.054540001	0.247180000	0.271959990

0.527090013	0.750000000	0.092200004

0.790899992	0.500000000	0.012880000

0.290899992	0.500000000	0.012880000

0.790899992	0.000000000	0.012880000

0.799189985	0.987339973	0.363400012

0.290899992	0.000000000	0.012880000

0.793929994	0.000000000	0.179269999

0.793929994	0.500000000	0.179269999

0.298299998	0.506560028	0.344289988

0.293929994	0.000000000	0.179269999

0.293929994	0.500000000	0.179269999

0.794089973	0.516189992	0.352539986

0.310330003	0.003590000	0.347920001

0.340770006	0.574159980	0.517099977

**The optimized configurations of PSFZM-Zn-Mo with adsorption *H_2_O_2_ before in-situ reconstruction**

1.00000000000000

7.7817001343000003	0.0000000000000000	0.0000000000000000

0.0000000000000000	7.7557001114000004	0.0000000000000000

0.0000000000000000	0.0000000000000000	23.3838996887000015

O	Pr	Sr	Fe	Zn	Mo	H

34	6	2	6	3	3	2

Selective dynamics

Direct

0.8295599819999993 0.4566999970000012 0.0960199980000027	F	F	F

0.3412323338481948 0.4603822202097415 0.2587307561483594	T	T	T

0.0408199990000000 0.5432999730000034 0.0005200000000016	F	F	F

0.5438500050000030 0.5432999730000034 0.1668999939999978	F	F	F

0.0267971995343402 0.5203258307733009 0.3408365398350087	T	T	T

0.2552599910000026 0.9567000269999966 0.0961199999999991	F	F	F

0.7619047783985423 0.9607551983534911 0.2708814323222584	T	T	T

0.5409700270000002 0.0432999989999985 0.0252399999999966	F	F	F

0.0440099979999999 0.0432999989999985 0.1916300059999969	F	F	F

0.3877192102323673 0.9361454116901269 0.4170903029125207	T	T	T

0.2552599910000026 0.5432999730000034 0.0961199999999991	F	F	F

0.7658804974721377 0.5630116157328501 0.2733492290110074	T	T	T

0.5409700270000002 0.4566999970000012 0.0252399999999966	F	F	F

0.0440099979999999 0.4566999970000012 0.1916300059999969	F	F	F

0.5522743852177643 0.4791505942568346 0.3558548903971337	T	T	T

0.8295599819999993 0.0432999989999985 0.0960199980000027	F	F	F

0.3892384203751815 0.0366984389861613 0.2793631554489127	T	T	T

0.0408199990000000 0.9567000269999966 0.0005200000000016	F	F	F

0.5438500050000030 0.9567000269999966 0.1668999939999978	F	F	F

0.0508300321123461 0.9711791611437773 0.3423044868549122	T	T	T

0.7658299799999995 0.7500000000000000 0.0257600010000019	F	F	F

0.2688699959999994 0.7500000000000000 0.1921399980000018	F	F	F

0.7745253793894641 0.7454452379620158 0.3805783804831223	T	T	T

0.2523500029999965 0.2500000000000000 0.0212999990000000	F	F	F

0.7553799749999968 0.2500000000000000 0.1876900050000003	F	F	F

0.2782990371080103 0.2306620413615734 0.3591717791015029	T	T	T

0.8159599900000032 0.2500000000000000 0.0000000000000000	F	F	F

0.3190000060000031 0.2500000000000000 0.1663900020000000	F	F	F

0.7928183203593744 0.2562734888602163 0.3454644722578993	T	T	T

0.3294399979999980 0.7500000000000000 0.0044600000000017	F	F	F

0.8324800130000014 0.7500000000000000 0.1708499940000010	F	F	F

0.3029499263241364 0.7588219358842110 0.3208025080140283	T	T	T

0.2951544189171845 0.6175597705812689 0.4398987716118941	T	T	T

0.4196393950313819 0.5356498038363556 0.4781749998714387	T	T	T

0.0306700000000006 0.7500000000000000 0.0909999980000009	F	F	F

0.5325521632942058 0.7669470379600523 0.2493357638923508	T	T	T

0.5577300189999974 0.2500000000000000 0.0999500010000034	F	F	F

0.0541599989999995 0.2500000000000000 0.1011499990000004	F	F	F

0.5786579410482005 0.2582630304574827 0.2680267059803946	T	T	T

0.0210770185764585 0.7657651646550800 0.2573445036874040	T	T	T

0.0501737851907612 0.2522781712033954 0.2714916976882585	T	T	T

0.5270900129999987 0.7500000000000000 0.0922000039999986	F	F	F

0.7908999919999999 0.5000000000000000 0.0128800000000027	F	F	F

0.2908999919999999 0.5000000000000000 0.0128800000000027	F	F	F

0.7908999919999999 0.0000000000000000 0.0128800000000027	F	F	F

0.7971238138403873 0.0027807849645045 0.3601828217018808	T	T	T

0.2908999919999999 0.0000000000000000 0.0128800000000027	F	F	F

0.7939299940000026 0.0000000000000000 0.1792699989999988	F	F	F

0.7939299940000026 0.5000000000000000 0.1792699989999988	F	F	F

0.2896070069342683 0.5099883429695106 0.3537069282257992	T	T	T

0.2939299940000026 0.0000000000000000 0.1792699989999988	F	F	F

0.2939299940000026 0.5000000000000000 0.1792699989999988	F	F	F

0.7829021750234398 0.5268750018688702 0.3523907737836161	T	T	T

0.2834948314452534 -0.0044341515267401 0.3534751158239235	T	T	T

0.3534786269851617 0.5386296587329861 0.5139781051841090	T	T	T

0.3458012968433611 0.7383803108956849 0.4345917801572730	T	T	T

0.00000000E+00 0.00000000E+00 0.00000000E+00

0.00000000E+00 0.00000000E+00 0.00000000E+00

0.00000000E+00 0.00000000E+00 0.00000000E+00

0.00000000E+00 0.00000000E+00 0.00000000E+00

0.00000000E+00 0.00000000E+00 0.00000000E+00

0.00000000E+00 0.00000000E+00 0.00000000E+00

0.00000000E+00 0.00000000E+00 0.00000000E+00

0.00000000E+00 0.00000000E+00 0.00000000E+00

0.00000000E+00 0.00000000E+00 0.00000000E+00

0.00000000E+00 0.00000000E+00 0.00000000E+00

0.00000000E+00 0.00000000E+00 0.00000000E+00

0.00000000E+00 0.00000000E+00 0.00000000E+00

0.00000000E+00 0.00000000E+00 0.00000000E+00

0.00000000E+00 0.00000000E+00 0.00000000E+00

0.00000000E+00 0.00000000E+00 0.00000000E+00

0.00000000E+00 0.00000000E+00 0.00000000E+00

0.00000000E+00 0.00000000E+00 0.00000000E+00

0.00000000E+00 0.00000000E+00 0.00000000E+00

0.00000000E+00 0.00000000E+00 0.00000000E+00

0.00000000E+00 0.00000000E+00 0.00000000E+00

0.00000000E+00 0.00000000E+00 0.00000000E+00

0.00000000E+00 0.00000000E+00 0.00000000E+00

0.00000000E+00 0.00000000E+00 0.00000000E+00

0.00000000E+00 0.00000000E+00 0.00000000E+00

0.00000000E+00 0.00000000E+00 0.00000000E+00

0.00000000E+00 0.00000000E+00 0.00000000E+00

0.00000000E+00 0.00000000E+00 0.00000000E+00

0.00000000E+00 0.00000000E+00 0.00000000E+00

0.00000000E+00 0.00000000E+00 0.00000000E+00

0.00000000E+00 0.00000000E+00 0.00000000E+00

0.00000000E+00 0.00000000E+00 0.00000000E+00

0.00000000E+00 0.00000000E+00 0.00000000E+00

0.00000000E+00 0.00000000E+00 0.00000000E+00

0.00000000E+00 0.00000000E+00 0.00000000E+00

0.00000000E+00 0.00000000E+00 0.00000000E+00

0.00000000E+00 0.00000000E+00 0.00000000E+00

0.00000000E+00 0.00000000E+00 0.00000000E+00

0.00000000E+00 0.00000000E+00 0.00000000E+00

0.00000000E+00 0.00000000E+00 0.00000000E+00

0.00000000E+00 0.00000000E+00 0.00000000E+00

0.00000000E+00 0.00000000E+00 0.00000000E+00

0.00000000E+00 0.00000000E+00 0.00000000E+00

0.00000000E+00 0.00000000E+00 0.00000000E+00

0.00000000E+00 0.00000000E+00 0.00000000E+00

0.00000000E+00 0.00000000E+00 0.00000000E+00

0.00000000E+00 0.00000000E+00 0.00000000E+00

0.00000000E+00 0.00000000E+00 0.00000000E+00

0.00000000E+00 0.00000000E+00 0.00000000E+00

0.00000000E+00 0.00000000E+00 0.00000000E+00

0.00000000E+00 0.00000000E+00 0.00000000E+00

0.00000000E+00 0.00000000E+00 0.00000000E+00

0.00000000E+00 0.00000000E+00 0.00000000E+00

0.00000000E+00 0.00000000E+00 0.00000000E+00

0.00000000E+00 0.00000000E+00 0.00000000E+00

0.00000000E+00 0.00000000E+00 0.00000000E+00

0.00000000E+00 0.00000000E+00 0.00000000E+00

**The optimized configurations of PSFZM-Zn-Mo with adsorption *H_2_O_2_ after in-situ reconstruction**

1.0

7.7817001343	0.0000000000	0.0000000000

0.0000000000	7.7557001114	0.0000000000

0.0000000000	0.0000000000	23.3838996887

O	Pr	Sr	Fe	Zn	Mo	H

34	6	2	6	3	3	2

Direct

0.829559982	0.456699997	0.096019998

0.351090014	0.455969989	0.258069992

0.040819999	0.543299973	0.000520000

0.543850005	0.543299973	0.166899994

0.038050000	0.526489973	0.333139986

0.255259991	0.956700027	0.096120000

0.759779990	0.965950012	0.272929996

0.540970027	0.043299999	0.025240000

0.044009998	0.043299999	0.191630006

0.523710012	0.988099992	0.378430009

0.255259991	0.543299973	0.096120000

0.766579986	0.569440007	0.274019986

0.540970027	0.456699997	0.025240000

0.044009998	0.456699997	0.191630006

0.556529999	0.481370002	0.354640007

0.829559982	0.043299999	0.096019998

0.361530006	0.047580000	0.271129996

0.040819999	0.956700027	0.000520000

0.543850005	0.956700027	0.166899994

0.060729999	0.970049977	0.346060008

0.765829980	0.750000000	0.025760001

0.268869996	0.750000000	0.192139998

0.799589992	0.707229972	0.394199997

0.252350003	0.250000000	0.021299999

0.755379975	0.250000000	0.187690005

0.276050001	0.236489996	0.359580010

0.815959990	0.250000000	0.000000000

0.319000006	0.250000000	0.166390002

0.810850024	0.265009999	0.348679990

0.329439998	0.750000000	0.004460000

0.832480013	0.750000000	0.170849994

0.311580002	0.760730028	0.322010010

0.290549994	0.571129978	0.424840003

0.381770015	0.511250019	0.476700008

0.030670000	0.750000000	0.090999998

0.536689997	0.764850020	0.248109996

0.557730019	0.250000000	0.099950001

0.054159999	0.250000000	0.101149999

0.588419974	0.251459986	0.270529985

0.023690000	0.768429995	0.259429991

0.054540001	0.247180000	0.271959990

0.527090013	0.750000000	0.092200004

0.790899992	0.500000000	0.012880000

0.290899992	0.500000000	0.012880000

0.790899992	0.000000000	0.012880000

0.799189985	0.987339973	0.363400012

0.290899992	0.000000000	0.012880000

0.793929994	0.000000000	0.179269999

0.793929994	0.500000000	0.179269999

0.298299998	0.506560028	0.344289988

0.293929994	0.000000000	0.179269999

0.293929994	0.500000000	0.179269999

0.794089973	0.516189992	0.352539986

0.310330003	0.003590000	0.347920001

0.340770006	0.574159980	0.517099977

0.364069998	0.685689986	0.439359993

**The optimized configurations of PSFZM-Zn-Zn with adsorption *OOH**

psfzm-zn-zn

1.0

7.7817001343	0.0000000000	0.0000000000

0.0000000000	7.7557001114	0.0000000000

0.0000000000	0.0000000000	23.3838996887

O	Pr	Sr	Fe	Mo	Zn	H

34	6	2	6	3	3	1

Direct

0.829559982	0.456699997	0.096019998

0.324559987	0.481629997	0.261040002

0.040819999	0.543299973	0.000520000

0.543850005	0.543299973	0.166899994

0.044000000	0.499819994	0.348800004

0.255259991	0.956700027	0.096120000

0.762679994	0.969760001	0.254689991

0.540970027	0.043299999	0.025240000

0.044009998	0.043299999	0.191630006

0.561060011	0.990390003	0.348619998

0.255259991	0.543299973	0.096120000

0.768180013	0.527149975	0.259700000

0.540970027	0.456699997	0.025240000

0.044009998	0.456699997	0.191630006

0.548009992	0.498690009	0.353320003

0.829559982	0.043299999	0.096019998

0.338869989	0.023460001	0.258679986

0.040819999	0.956700027	0.000520000

0.543850005	0.956700027	0.166899994

0.033349998	0.979309976	0.334679991

0.765829980	0.750000000	0.025760001

0.268869996	0.750000000	0.192139998

0.792819977	0.754800022	0.345809996

0.252350003	0.250000000	0.021299999

0.755379975	0.250000000	0.187690005

0.306840003	0.240490004	0.355089992

0.815959990	0.250000000	0.000000000

0.319000006	0.250000000	0.166390002

0.798349977	0.229080006	0.341639996

0.329439998	0.750000000	0.004460000

0.832480013	0.750000000	0.170849994

0.290930003	0.745230019	0.344599992

0.301510006	0.516749978	0.447140008

0.378289998	0.416590005	0.494569987

0.030670000	0.750000000	0.090999998

0.528180003	0.732169986	0.280869991

0.557730019	0.250000000	0.099950001

0.054159999	0.250000000	0.101149999

0.538209975	0.256830007	0.287250012

0.050519999	0.716530025	0.279489994

0.070100002	0.253140002	0.279650003

0.527090013	0.750000000	0.092200004

0.790899992	0.500000000	0.012880000

0.793929994	0.500000000	0.179269999

0.293929994	0.000000000	0.179269999

0.798399985	0.991100013	0.348199993

0.290899992	0.000000000	0.012880000

0.293929994	0.500000000	0.179269999

0.290899992	0.500000000	0.012880000

0.790899992	0.000000000	0.012880000

0.793929994	0.000000000	0.179269999

0.294030011	0.493070006	0.364160001

0.797389984	0.491609991	0.357439995

0.292430013	0.993179977	0.354959995

0.354409993	0.472380012	0.537549973

**The optimized configurations of PSFZ-Fe-Fe with adsorption *OOH**

psfz-fe-fe

1.0

7.7817001343	0.0000000000	0.0000000000

0.0000000000	7.7557001114	0.0000000000

0.0000000000	0.0000000000	23.3838996887

O	Pr	Sr	Fe	Zn	H

34	6	2	9	3	1

Direct

0.829559982	0.456699997	0.096019998

0.336659998	0.474009991	0.264140010

0.040819999	0.543299973	0.000520000

0.543850005	0.543299973	0.166899994

0.047499999	0.512799978	0.339859992

0.255259991	0.956700027	0.096120000

0.768800020	0.960739970	0.261299998

0.540970027	0.043299999	0.025240000

0.044009998	0.043299999	0.191630006

0.546549976	0.002390000	0.348179996

0.255259991	0.543299973	0.096120000

0.766979992	0.535440028	0.265729994

0.540970027	0.456699997	0.025240000

0.044009998	0.456699997	0.191630006

0.546670020	0.493900001	0.353230000

0.829559982	0.043299999	0.096019998

0.337769985	0.028550001	0.259550005

0.040819999	0.956700027	0.000520000

0.543850005	0.956700027	0.166899994

0.047920000	0.985400021	0.336809993

0.765829980	0.750000000	0.025760001

0.268869996	0.750000000	0.192139998

0.798460007	0.751810014	0.355859995

0.252350003	0.250000000	0.021299999

0.755379975	0.250000000	0.187690005

0.296959996	0.246130005	0.351080000

0.815959990	0.250000000	0.000000000

0.319000006	0.250000000	0.166390002

0.796419978	0.247370005	0.340959996

0.329439998	0.750000000	0.004460000

0.832480013	0.750000000	0.170849994

0.299879998	0.751119971	0.332300007

0.330390006	0.514649987	0.429509997

0.316749990	0.642539978	0.476269990

0.030670000	0.750000000	0.090999998

0.527610004	0.746609986	0.258309990

0.557730019	0.250000000	0.099950001

0.054159999	0.250000000	0.101149999

0.556079984	0.253419995	0.274789989

0.026960000	0.742449999	0.265040010

0.053789999	0.252570003	0.271169990

0.527090013	0.750000000	0.092200004

0.790899992	0.500000000	0.012880000

0.794200003	0.505370021	0.347479999

0.290899992	0.500000000	0.012880000

0.298729986	0.498640001	0.344799995

0.790899992	0.000000000	0.012880000

0.293929994	0.000000000	0.179269999

0.795570016	0.994790018	0.345220000

0.793929994	0.000000000	0.179269999

0.297529995	0.999419987	0.342610002

0.293929994	0.500000000	0.179269999

0.793929994	0.500000000	0.179269999

0.290899992	0.000000000	0.012880000

0.349940002	0.583199978	0.518029988

**The optimized configurations of PSFZ-Fe-Zn with adsorption *OOH**

psfz-fe-zn

1.0

7.7817001343	0.0000000000	0.0000000000

0.0000000000	7.7557001114	0.0000000000

0.0000000000	0.0000000000	23.3838996887

O	Pr	Sr	Fe	Zn	H

34	6	2	9	3	1

Direct

0.829559982	0.456699997	0.096019998

0.329679996	0.475279987	0.262939990

0.040819999	0.543299973	0.000520000

0.543850005	0.543299973	0.166899994

0.052749999	0.510919988	0.339489996

0.255259991	0.956700027	0.096120000

0.767679989	0.966220021	0.258969992

0.540970027	0.043299999	0.025240000

0.044009998	0.043299999	0.191630006

0.550679982	0.998210013	0.344689995

0.255259991	0.543299973	0.096120000

0.767340004	0.530250013	0.258249998

0.540970027	0.456699997	0.025240000

0.044009998	0.456699997	0.191630006

0.539279997	0.502279997	0.347310007

0.829559982	0.043299999	0.096019998

0.332450002	0.025830001	0.257499993

0.040819999	0.956700027	0.000520000

0.543850005	0.956700027	0.166899994

0.033890001	0.981909990	0.337769985

0.765829980	0.750000000	0.025760001

0.268869996	0.750000000	0.192139998

0.788079977	0.753939986	0.346599996

0.252350003	0.250000000	0.021299999

0.755379975	0.250000000	0.187690005

0.301730007	0.257820010	0.350899994

0.815959990	0.250000000	0.000000000

0.319000006	0.250000000	0.166390002

0.793060005	0.237590000	0.340790004

0.329439998	0.750000000	0.004460000

0.832480013	0.750000000	0.170849994

0.293130010	0.743610024	0.333909988

0.338679999	0.486770004	0.428920001

0.353370011	0.610880017	0.476749986

0.030670000	0.750000000	0.090999998

0.528389990	0.747820020	0.265410006

0.557730019	0.250000000	0.099950001

0.054159999	0.250000000	0.101149999

0.549849987	0.251800001	0.276450008

0.029570000	0.742030025	0.267760009

0.055399999	0.248649999	0.272920012

0.527090013	0.750000000	0.092200004

0.790899992	0.500000000	0.012880000

0.290899992	0.500000000	0.012880000

0.793929994	0.500000000	0.179269999

0.295399994	0.498860002	0.344709992

0.790899992	0.000000000	0.012880000

0.293929994	0.000000000	0.179269999

0.792990029	0.994310021	0.344419986

0.290899992	0.000000000	0.012880000

0.793929994	0.000000000	0.179269999

0.293929994	0.500000000	0.179269999

0.797720015	0.496270001	0.352169991

0.290369987	0.998430014	0.350089997

0.380250007	0.573409975	0.521679997

**The optimized configurations of PSFZ-Zn-Fe with adsorption *OOH**

psfz-zn-fe

1.0

7.7817001343	0.0000000000	0.0000000000

0.0000000000	7.7557001114	0.0000000000

0.0000000000	0.0000000000	23.3838996887

O	Pr	Sr	Fe	Zn	H

34	6	2	9	3	1

Direct

0.829559982	0.456699997	0.096019998

0.332920015	0.475870013	0.263029993

0.040819999	0.543299973	0.000520000

0.543850005	0.543299973	0.166899994

0.033690002	0.513849974	0.339729995

0.255259991	0.956700027	0.096120000

0.769649982	0.958859980	0.261249989

0.540970027	0.043299999	0.025240000

0.044009998	0.043299999	0.191630006

0.545509994	0.996050000	0.347460002

0.255259991	0.543299973	0.096120000

0.767910004	0.536490023	0.260879993

0.540970027	0.456699997	0.025240000

0.044009998	0.456699997	0.191630006

0.554719985	0.499139994	0.348109990

0.829559982	0.043299999	0.096019998

0.334100008	0.027060000	0.259420007

0.040819999	0.956700027	0.000520000

0.543850005	0.956700027	0.166899994

0.044989999	0.986599982	0.337500006

0.765829980	0.750000000	0.025760001

0.268869996	0.750000000	0.192139998

0.795130014	0.748870015	0.354220003

0.252350003	0.250000000	0.021299999

0.755379975	0.250000000	0.187690005

0.300550014	0.234689996	0.351599991

0.815959990	0.250000000	0.000000000

0.319000006	0.250000000	0.166390002

0.793799996	0.248640001	0.339340001

0.329439998	0.750000000	0.004460000

0.832480013	0.750000000	0.170849994

0.292979985	0.758029997	0.333160013

0.326590002	0.502390027	0.433440000

0.312999994	0.620100021	0.483049989

0.030670000	0.750000000	0.090999998

0.523530006	0.743789971	0.262470007

0.557730019	0.250000000	0.099950001

0.054159999	0.250000000	0.101149999

0.546959996	0.255290002	0.275510013

0.032049999	0.743420005	0.266229987

0.059710000	0.253910005	0.272340000

0.527090013	0.750000000	0.092200004

0.790899992	0.500000000	0.012880000

0.793190002	0.502749979	0.346069992

0.290899992	0.500000000	0.012880000

0.793929994	0.500000000	0.179269999

0.790899992	0.000000000	0.012880000

0.293929994	0.000000000	0.179269999

0.794170022	0.995100021	0.343950003

0.793929994	0.000000000	0.179269999

0.295199990	0.998030007	0.343760014

0.293929994	0.500000000	0.179269999

0.292189986	0.498930007	0.350849986

0.290899992	0.000000000	0.012880000

0.347180009	0.566219985	0.525529981

**The optimized configurations of PSFZ-Zn-Zn with adsorption *OOH**

psfz-zn-zn

1.0

7.7817001343	0.0000000000	0.0000000000

0.0000000000	7.7557001114	0.0000000000

0.0000000000	0.0000000000	23.3838996887

O	Pr	Sr	Fe	Zn	H

34	6	2	9	3	1

Direct

0.829559982	0.456699997	0.096019998

0.329149991	0.476260006	0.256449997

0.040819999	0.543299973	0.000520000

0.543850005	0.543299973	0.166899994

0.039960001	0.512860000	0.339920014

0.255259991	0.956700027	0.096120000

0.766189992	0.967610002	0.259380013

0.540970027	0.043299999	0.025240000

0.044009998	0.043299999	0.191630006

0.550059974	0.997770011	0.345959991

0.255259991	0.543299973	0.096120000

0.766579986	0.529349983	0.257669985

0.540970027	0.456699997	0.025240000

0.044009998	0.456699997	0.191630006

0.543079972	0.500899971	0.345039994

0.829559982	0.043299999	0.096019998

0.331299990	0.025280001	0.256900012

0.040819999	0.956700027	0.000520000

0.543850005	0.956700027	0.166899994

0.030260000	0.983829975	0.338910013

0.765829980	0.750000000	0.025760001

0.268869996	0.750000000	0.192139998

0.782859981	0.755999982	0.346340001

0.252350003	0.250000000	0.021299999

0.755379975	0.250000000	0.187690005

0.304630011	0.248089999	0.349059999

0.815959990	0.250000000	0.000000000

0.319000006	0.250000000	0.166390002

0.791230023	0.237029999	0.341870010

0.329439998	0.750000000	0.004460000

0.832480013	0.750000000	0.170849994

0.288190007	0.751129985	0.334140003

0.318379998	0.535220027	0.435020000

0.307520002	0.654320002	0.484340012

0.030670000	0.750000000	0.090999998

0.527849972	0.747179985	0.268640012

0.557730019	0.250000000	0.099950001

0.054159999	0.250000000	0.101149999

0.547540009	0.252779990	0.277449995

0.028650001	0.742519975	0.270000011

0.056150001	0.250779986	0.274450004

0.527090013	0.750000000	0.092200004

0.790899992	0.500000000	0.012880000

0.293929994	0.500000000	0.179269999

0.290899992	0.500000000	0.012880000

0.793929994	0.500000000	0.179269999

0.790899992	0.000000000	0.012880000

0.293929994	0.000000000	0.179269999

0.791180015	0.995570004	0.344880015

0.290899992	0.000000000	0.012880000

0.793929994	0.000000000	0.179269999

0.793730021	0.496850014	0.353469998

0.289310008	0.501770020	0.352939993

0.287999988	0.997780025	0.351749986

0.332199991	0.580579996	0.524179995

**The optimized configurations of PSFM-Fe-Fe with adsorption *OOH**

psfm-fe-fe

1.0

7.7817001343	0.0000000000	0.0000000000

0.0000000000	7.7557001114	0.0000000000

0.0000000000	0.0000000000	23.3838996887

O	Pr	Sr	Fe	Mo	H

34	6	2	9	3	1

Direct

0.829559982	0.456699997	0.096019998

0.336479992	0.473309994	0.255780011

0.040819999	0.543299973	0.000520000

0.543850005	0.543299973	0.166899994

0.041239999	0.526069999	0.334230006

0.255259991	0.956700027	0.096120000

0.765690029	0.957819998	0.260419995

0.540970027	0.043299999	0.025240000

0.044009998	0.043299999	0.191630006

0.546959996	0.021980001	0.351209998

0.255259991	0.543299973	0.096120000

0.764660001	0.536800027	0.254269987

0.540970027	0.456699997	0.025240000

0.044009998	0.456699997	0.191630006

0.553590000	0.474830002	0.349849999

0.829559982	0.043299999	0.096019998

0.342290014	0.028120000	0.258870006

0.040819999	0.956700027	0.000520000

0.543850005	0.956700027	0.166899994

0.046429999	0.977509975	0.336349994

0.765829980	0.750000000	0.025760001

0.268869996	0.750000000	0.192139998

0.770619988	0.747739971	0.350890011

0.252350003	0.250000000	0.021299999

0.755379975	0.250000000	0.187690005

0.282920003	0.251850009	0.350739986

0.815959990	0.250000000	0.000000000

0.319000006	0.250000000	0.166390002

0.808790028	0.255050004	0.336230010

0.329439998	0.750000000	0.004460000

0.832480013	0.750000000	0.170849994

0.312429994	0.748709977	0.332760006

0.337559998	0.522490025	0.432179987

0.342570007	0.669719994	0.472409993

0.030670000	0.750000000	0.090999998

0.531599998	0.769410014	0.267679989

0.557730019	0.250000000	0.099950001

0.054159999	0.250000000	0.101149999

0.558669984	0.235650003	0.282660007

0.025920000	0.763620019	0.272909999

0.057670001	0.238690004	0.277040005

0.527090013	0.750000000	0.092200004

0.790899992	0.500000000	0.012880000

0.790329993	0.503160000	0.348699987

0.290899992	0.500000000	0.012880000

0.298999995	0.497049987	0.348040015

0.790899992	0.000000000	0.012880000

0.293929994	0.000000000	0.179269999

0.793370008	0.995859981	0.349539995

0.793929994	0.000000000	0.179269999

0.299890012	0.001700000	0.348800004

0.293929994	0.500000000	0.179269999

0.793929994	0.500000000	0.179269999

0.290899992	0.000000000	0.012880000

0.369019985	0.614180028	0.515259981

**The optimized configurations of PSFM-Fe-Mo with adsorption *OOH**

psfm-fe-mo

1.0

7.7817001343	0.0000000000	0.0000000000

0.0000000000	7.7557001114	0.0000000000

0.0000000000	0.0000000000	23.3838996887

O	Pr	Sr	Fe	Mo	H

34	6	2	9	3	1

Direct

0.829559982	0.456699997	0.096019998

0.348410010	0.456299990	0.258619994

0.040819999	0.543299973	0.000520000

0.543850005	0.543299973	0.166899994

0.045419998	0.531090021	0.330570012

0.255259991	0.956700027	0.096120000

0.760580003	0.959249973	0.266689986

0.540970027	0.043299999	0.025240000

0.044009998	0.043299999	0.191630006

0.551329970	0.019290000	0.361290008

0.255259991	0.543299973	0.096120000

0.767960012	0.559899986	0.268920004

0.540970027	0.456699997	0.025240000

0.044009998	0.456699997	0.191630006

0.556010008	0.470259994	0.350950003

0.829559982	0.043299999	0.096019998

0.346679986	0.040240001	0.266409993

0.040819999	0.956700027	0.000520000

0.543850005	0.956700027	0.166899994

0.053739998	0.966549993	0.336970001

0.765829980	0.750000000	0.025760001

0.268869996	0.750000000	0.192139998

0.796750009	0.705070019	0.391429991

0.252350003	0.250000000	0.021299999

0.755379975	0.250000000	0.187690005

0.266880006	0.229000002	0.363579988

0.815959990	0.250000000	0.000000000

0.319000006	0.250000000	0.166390002

0.822619975	0.269019991	0.347719997

0.329439998	0.750000000	0.004460000

0.832480013	0.750000000	0.170849994

0.326730013	0.761189997	0.334320009

0.312390000	0.476680011	0.433050007

0.446110010	0.494459987	0.477670014

0.030670000	0.750000000	0.090999998

0.534160018	0.759230018	0.252579987

0.557730019	0.250000000	0.099950001

0.054159999	0.250000000	0.101149999

0.582530022	0.240539998	0.275860012

0.023520000	0.766349971	0.259700000

0.057859998	0.246879995	0.272700012

0.527090013	0.750000000	0.092200004

0.790899992	0.500000000	0.012880000

0.290899992	0.500000000	0.012880000

0.793929994	0.500000000	0.179269999

0.298770010	0.500209987	0.347999990

0.790899992	0.000000000	0.012880000

0.293929994	0.000000000	0.179269999

0.806720018	0.989329994	0.359070003

0.290899992	0.000000000	0.012880000

0.793929994	0.000000000	0.179269999

0.293929994	0.500000000	0.179269999

0.797900021	0.515160024	0.349689990

0.311419994	0.003450000	0.346769989

0.402020007	0.473399997	0.522270024

**The optimized configurations of PSFM-Mo-Fe with adsorption *OOH**

psfm-mo-fe

1.0

7.7817001343	0.0000000000	0.0000000000

0.0000000000	7.7557001114	0.0000000000

0.0000000000	0.0000000000	23.3838996887

O	Pr	Sr	Fe	Mo	H

34	6	2	9	3	1

Direct

0.829559982	0.456699997	0.096019998

0.324180007	0.445820004	0.265159994

0.040819999	0.543299973	0.000520000

0.543850005	0.543299973	0.166899994

0.047640000	0.522930026	0.329609990

0.255259991	0.956700027	0.096120000

0.769580007	0.981100023	0.265210003

0.540970027	0.043299999	0.025240000

0.044009998	0.043299999	0.191630006

0.546069980	0.019120000	0.352200001

0.255259991	0.543299973	0.096120000

0.749040008	0.539279997	0.262620002

0.540970027	0.456699997	0.025240000

0.044009998	0.456699997	0.191630006

0.532280028	0.497610003	0.351570010

0.829559982	0.043299999	0.096019998

0.335880011	0.034850001	0.263040006

0.040819999	0.956700027	0.000520000

0.543850005	0.956700027	0.166899994

0.040360000	0.995119989	0.342359990

0.765829980	0.750000000	0.025760001

0.268869996	0.750000000	0.192139998

0.784009993	0.754769981	0.351520002

0.252350003	0.250000000	0.021299999

0.755379975	0.250000000	0.187690005

0.259510010	0.301360011	0.381480008

0.815959990	0.250000000	0.000000000

0.319000006	0.250000000	0.166390002

0.796660006	0.262459993	0.342869997

0.329439998	0.750000000	0.004460000

0.832480013	0.750000000	0.170849994

0.295480013	0.739880025	0.344689995

0.301510006	0.553110003	0.429300010

0.395280004	0.656499982	0.472380012

0.030670000	0.750000000	0.090999998

0.540730000	0.783420026	0.276430011

0.557730019	0.250000000	0.099950001

0.054159999	0.250000000	0.101149999

0.572749972	0.237130001	0.272579998

0.015510000	0.778599977	0.269899994

0.040070001	0.238230005	0.271840006

0.527090013	0.750000000	0.092200004

0.790899992	0.500000000	0.012880000

0.793280005	0.510399997	0.344020009

0.290899992	0.500000000	0.012880000

0.793929994	0.500000000	0.179269999

0.790899992	0.000000000	0.012880000

0.293929994	0.000000000	0.179269999

0.794120014	0.007330000	0.354070008

0.793929994	0.000000000	0.179269999

0.290879995	0.015680000	0.357609987

0.293929994	0.500000000	0.179269999

0.293760002	0.497449994	0.343640000

0.290899992	0.000000000	0.012880000

0.364459991	0.626209974	0.517629981

**The optimized configurations of PSFM-Mo-Mo with adsorption *OOH**

psfm-mo-mo

1.0

7.7817001343	0.0000000000	0.0000000000

0.0000000000	7.7557001114	0.0000000000

0.0000000000	0.0000000000	23.3838996887

O	Pr	Sr	Fe	Mo	H

34	6	2	9	3	1

Direct

0.829559982	0.456699997	0.096019998

0.353069991	0.462300003	0.263379991

0.040819999	0.543299973	0.000520000

0.543850005	0.543299973	0.166899994

0.058230001	0.524060011	0.338369995

0.255259991	0.956700027	0.096120000

0.760959983	0.960420012	0.268269986

0.540970027	0.043299999	0.025240000

0.044009998	0.043299999	0.191630006

0.531620026	0.012900000	0.371870011

0.255259991	0.543299973	0.096120000

0.768450022	0.549269974	0.268790007

0.540970027	0.456699997	0.025240000

0.044009998	0.456699997	0.191630006

0.548080027	0.479840010	0.357320011

0.829559982	0.043299999	0.096019998

0.353249997	0.027109999	0.266790003

0.040819999	0.956700027	0.000520000

0.543850005	0.956700027	0.166899994

0.066299997	0.972050011	0.344709992

0.765829980	0.750000000	0.025760001

0.268869996	0.750000000	0.192139998

0.794229984	0.721319973	0.381859988

0.252350003	0.250000000	0.021299999

0.755379975	0.250000000	0.187690005

0.280149996	0.252319992	0.354140013

0.815959990	0.250000000	0.000000000

0.319000006	0.250000000	0.166390002

0.812860012	0.263770014	0.346130013

0.329439998	0.750000000	0.004460000

0.832480013	0.750000000	0.170849994

0.326830000	0.744579971	0.338910013

0.324209988	0.501540005	0.431450009

0.316659987	0.623070002	0.480179995

0.030670000	0.750000000	0.090999998

0.534500003	0.751890004	0.246370003

0.557730019	0.250000000	0.099950001

0.054159999	0.250000000	0.101149999

0.576839983	0.240260005	0.268249989

0.023180000	0.757539988	0.254629999

0.055890001	0.247470006	0.267600000

0.527090013	0.750000000	0.092200004

0.790899992	0.500000000	0.012880000

0.293929994	0.500000000	0.179269999

0.290899992	0.500000000	0.012880000

0.793929994	0.500000000	0.179269999

0.790899992	0.000000000	0.012880000

0.293929994	0.000000000	0.179269999

0.799989998	0.989160001	0.360020012

0.290899992	0.000000000	0.012880000

0.793929994	0.000000000	0.179269999

0.800629973	0.510950029	0.350730002

0.308369994	0.496010005	0.343959987

0.309249997	0.998030007	0.347889990

0.336340010	0.528999984	0.515349984

**The optimized configurations of PSF-Fe with adsorption *OOH**

psf-fe

1.0

7.7817001343	0.0000000000	0.0000000000

0.0000000000	7.7557001114	0.0000000000

0.0000000000	0.0000000000	23.3598003387

O	Pr	Sr	Fe	H

32	6	2	12	1

Direct

0.429560006	0.956700027	0.095610000

0.921060026	0.961199999	0.263440013

0.640820026	0.043299999	0.000000000

0.143849999	0.043299999	0.166559994

0.635670006	0.027450001	0.340799987

0.855260015	0.456699997	0.095710002

0.378300011	0.447409987	0.263729990

0.140970007	0.543299973	0.024750000

0.644010007	0.543299973	0.191310003

0.158150002	0.504909992	0.352849990

0.855260015	0.043299999	0.095710002

0.378300011	0.052590001	0.263729990

0.140970007	0.956700027	0.024750000

0.644010007	0.956700027	0.191310003

0.158150002	0.995090008	0.352849990

0.429560006	0.543299973	0.095610000

0.921060026	0.538800001	0.263440013

0.640820026	0.456699997	0.000000000

0.143849999	0.456699997	0.166559994

0.635670006	0.472550005	0.340799987

0.365830004	0.250000000	0.025270000

0.868860006	0.250000000	0.191829994

0.852349997	0.750000000	0.020800000

0.355379999	0.750000000	0.187360004

0.882489979	0.750000000	0.351870000

0.919000030	0.750000000	0.166040003

0.413569987	0.750000000	0.326270014

0.929440022	0.250000000	0.003950000

0.432480007	0.250000000	0.170509994

0.898299992	0.250000000	0.337639987

0.390940011	0.454769999	0.425460011

0.415870011	0.571359992	0.474920005

0.630670011	0.250000000	0.090580001

0.157729998	0.750000000	0.099540003

0.671549976	0.750000000	0.268640012

0.144260004	0.750000000	0.273369998

0.127090007	0.250000000	0.091770001

0.638119996	0.250000000	0.262470007

0.127389997	0.250000000	0.260560006

0.654160023	0.750000000	0.100740001

0.390899986	0.000000000	0.012380000

0.893930018	0.000000000	0.178939998

0.396459997	0.987489998	0.342040002

0.890900016	0.000000000	0.012380000

0.393929988	0.000000000	0.178939998

0.902010024	0.003650000	0.346670002

0.390899986	0.500000000	0.012380000

0.893930018	0.500000000	0.178939998

0.396459997	0.512510002	0.342040002

0.890900016	0.500000000	0.012380000

0.393929988	0.500000000	0.178939998

0.902010024	0.496349990	0.346670002

0.418309987	0.490500003	0.514119983
